# Synthesis of Fluorescent Cyclic Peptides via Gold(I)-Catalyzed Macrocyclization

**DOI:** 10.1021/jacs.3c09261

**Published:** 2023-11-30

**Authors:** Xing-Yu Liu, Wei Cai, Nathan Ronceray, Aleksandra Radenovic, Beat Fierz, Jerome Waser

**Affiliations:** † Laboratory of Catalysis and Organic Synthesis, École Polytechnique Fédérale de Lausanne, EPFL SB ISIC LCSO, 1015 Lausanne, Switzerland; ‡ Laboratory of Biophysical Chemistry of Macromolecules, Institute of Chemical Sciences and Engineering, École Polytechnique Fédérale de Lausanne, EPFL SB ISIC LCBM, 1015 Lausanne, Switzerland; § Laboratory of Nanoscale Biology, School of Engineering, Institute of Bioengineering, EPFL STI IBI LBEN, 1015 Lausanne, Switzerland

## Abstract

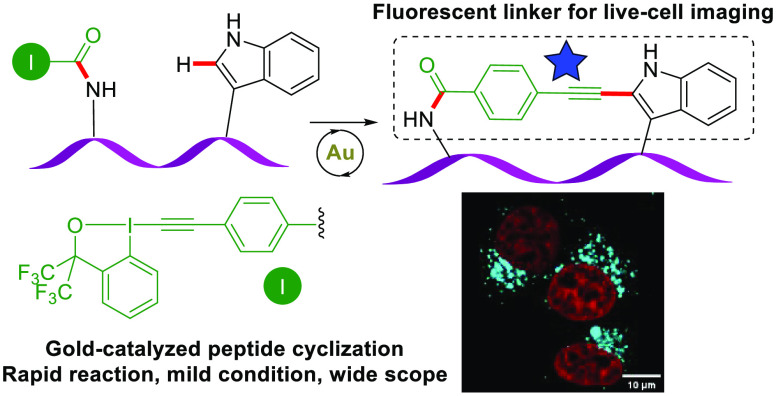

Rapid and efficient cyclization methods that form structurally novel peptidic macrocycles are of high importance for medicinal chemistry. Herein, we report the first gold(I)-catalyzed macrocyclization of peptide-EBXs (ethynylbenziodoxolones) via C_2_-Trp C–H activation. This reaction was carried out in the presence of protecting group free peptide sequences and is enabled by a simple commercial gold catalyst (AuCl·Me_2_S). The method displayed a rapid reaction rate (within 10 min), wide functional group tolerance (27 unprotected peptides were cyclized), and up to 86% isolated yield. The obtained highly conjugated cyclic peptide linker, formed through C–H alkynylation, can be directly applied to live-cell imaging as a fluorescent probe without further attachment of fluorophores.

Modulating protein–protein interactions (PPIs) is a promising strategy for the next generation of therapeutics.^1^ Given that small-molecule drugs (<500 Da) are too small to target the interfaces of PPIs, while larger biologics (>5000 Da) suffer from poor cell permeability and bioavailability, medium-size peptide drugs are highly promising to target PPIs. Compared with their linear counterparts, cyclic peptides show enhanced cell permeability, stability, and affinity toward protein surfaces involved in PPIs.^2^ Therefore, the development of peptide macrocyclization strategies is of great interest.^3^ However, the synthesis of cyclic peptides remains challenging as the favored *trans* geometry of the amide bond disfavors cyclization. As a result, intermolecular cross-coupling is challenging to suppress.

Traditional methods for the synthesis of peptide macrocycles rely mainly on low concentration lactamization and disulfide exchange.^4^ More recently, various transition metal-catalyzed reactions have been developed,^5^ including azide–alkyne cyclization (Cu),^6^ olefin metathesis (Ru),^7^ and cross-coupling (Pd).^8^ To further expand the toolbox with higher structure diversities and atom economy, metal-catalyzed C–H activation has emerged as a powerful tool to construct cyclic peptides.^9^ As shown in Scheme 1a, these transformations proceed through C–H activation, followed by migratory insertion or transmetalation, generating a cyclic metal peptide species. This intermediate undergoes reductive elimination (RE) or β-H elimination, leading to the corresponding cyclic peptides. However, these methods usually require high reaction temperature, long reaction time, strong oxidants, and protecting groups on the side chains.

**Scheme 1:**
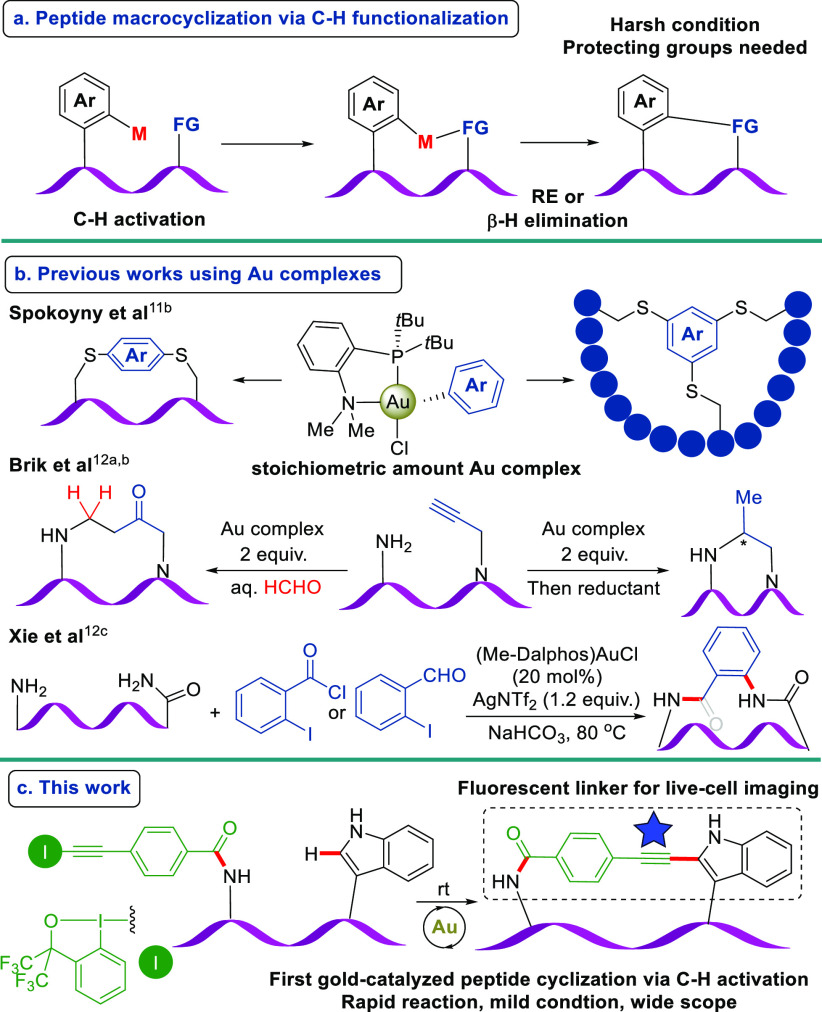
(a) Peptide Macrocyclization via C–H Activation; (b) Previous Works on Peptide Macrocyclization with Gold Complexes; (c) Gold(I)-Catalyzed Macrocyclization of Peptide-EBXs

Gold complexes are known to coordinate chemoselectively to unsaturated bonds and activate them under mild conditions.^10a^ Additionally, their redox chemistry has been increasingly exploited.^10b^ Nevertheless, gold complexes have rarely been used for peptide macrocyclization. Spokoyny and co-workers reported a cysteine S-arylation, enabled by an aminophosphine aryl-gold(III) complex, forming a wide array of stapled peptides or peptide bicycles (Scheme 1b).^11b^ However, the use of a stoichiometric amount of gold complexes was required. Later, two gold(I)-mediated peptide cyclizations via activation of propargylated peptides were disclosed by Brik’s group (Scheme 1b).^12a,12b^ The reaction displayed excellent functional group compatibility, but a stoichiometric amount of gold complex was still required. Very recently, Xie and co-workers^12c^ reported a gold(I)-catalyzed arylation of C-terminal-amidated peptides. By installing an acid chloride or an aldehyde as extra reactive handles on the arene iodide, they achieved head-to-tail cyclization. To the best of our knowledge, there is no report of peptide macrocyclization via C–H activation enabled by a gold catalyst despite their excellent functional group tolerance.

In 2009, our group reported the gold(I)-catalyzed C–H alkynylation of indoles and pyrroles by using hypervalent iodine reagents.^13a,13b^ This reaction can be also realized on other electron-rich aromatic rings including aniline,^13c^ thiophene,^13d^ and furan.^13e,13f^ In 2016, our group^14a^ and Hoeg-Jensen and Skrydstrup’s group^14b^ applied this reaction on peptides for the C_2_-selective ethynylation of Trp with TIPS-EBX (triisopropylsilyl-ethynylbenziodoxolone).

Hoeg-Jensen and co-workers showed that this method was also applicable to modify Trp in the protein apomyoglobin. Based on our recent development of peptide-bound hypervalent iodine reagents (peptide-EBXs, Scheme 2),^15^ we envisioned that this gold(I)-catalyzed C–H alkynylation could be applied on Trp-containing peptides in an intramolecular fashion. Herein, we present the discovery and development of the first gold(I)-catalyzed Trp-C_2_ alkynylation–cyclization of peptides (Scheme 1c).

**Scheme 2:**
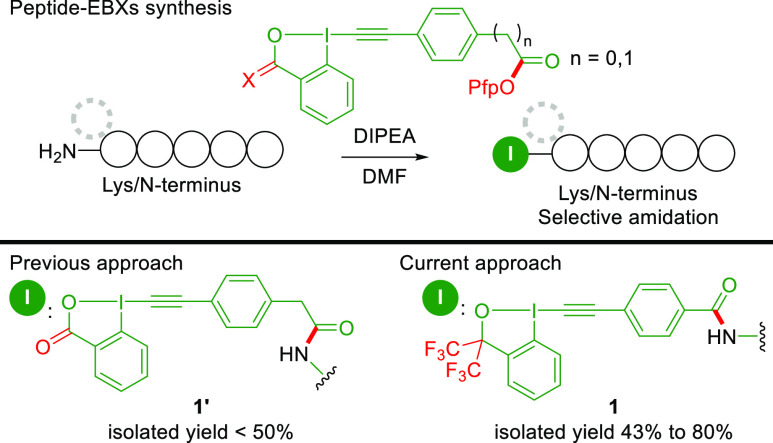
Synthesis of Peptide-EBXs with Bis-CF_3_ Benziodoxole **1** and Benziodoxolone **1′**

In our previous work on peptide-EBXs, we introduced ethynylbenziodoxolones onto peptides.^15^ The amidation between the Lys/N-terminus and an activated ester (OPfp) selectively occurred without touching the other electrophilic sites on the EBX core. The introduced EBXs handle allowed us to conduct peptide modification and macrocyclization through thiol addition and photocatalytic decarboxylative alkynylation. However, the instability of benziodoxolone **1′** led to partial decomposition during purification, and no transition-metal-based macrocyclization was known at this stage. Switching the EBX backbone from benziodoxolone **1′** to bis-CF_3_ benziodoxole **1**(16) enhanced the stability of the peptide-EBXs with higher isolated yields (Scheme 2; see Figure S1 in the Supporting Information (SI) for details). However, compared with benziodoxolones, benziodoxoles displayed inferior reactivity in intermolecular reactions with indoles.^13b^ Therefore, peptide-EBXs **1** may not be reactive enough in the envisaged macrocyclization. Peptide-EBX **1a** (AcKLAFW-OH) was chosen as the model substrate, as a similar sequence showed good cyclization tendency in our previous studies (Table 1).^15^ We first screened different solvents at 5 mM concentration using 100 mol % of the commercially available gold(I) catalyst AuCl·Me_2_S.^14^ Unfortunately, no desired product **2a** was observed in DMF and DMSO (Table 1, entries 1 and 2). To our delight, the Lys-Trp cyclization product was obtained in 64% HPLC-UV yield with 99:1 C2/C4 regioselectivity (see detailed discussion on the structure of the two regioisomers in the SI) by using 2% TFA as EBX activator in MeCN (entry 3).^13d^ Full conversion of **1a** was observed within 10 min without protection from light under air. With the hope that protic solvents would be enough to promote the reaction under less acidic conditions, we further screened methanol and more acidic fluoro-substituted alcohols (entries 4–6). Using MeOH gave comparable results even in the absence of TFA (entry 4). Fluoro-substituted alcohols further significantly enhanced the cyclization efficiency (entries 5 and 6). A 92% HPLC-UV yield was obtained by using HFIP without influencing the regioselectivity (entry 6). The reaction proceeded smoothly with 50 or 10 mol % of gold catalyst without a decrease in the yield (entries 7 and 8). Further lowering the catalyst loading to 1 mol % resulted still in 55% yield of **2a** after 16 h, with 37% yield of recovered **1a** (entry 9). Testing the reaction at a lower (2.5 mM) or higher concentration (10 mM), a drop in yield was observed (entries 10 and 11). We further evaluated different types of metal catalysts (entries 12–14). Simple AuCl also displayed excellent catalytic activity (entry 12). Silver and palladium catalysts showed no desired reactivity (entries 13 and 14). Only decomposition of starting material **1a** was observed. Reagent **1a′**, with a benziodoxolone backbone, was also examined under the same reaction conditions, resulting in a lower yield and regioselectivity (entry 15). As a control, no reaction happened in the absence of the gold catalyst (entry 16). Based on these results, we selected 10 mol % AuCl·Me_2_S and HFIP as solvent as the optimized conditions (entry 8), and the desired product was isolated in 56% isolated yield as a mixture of two regioisomers **2a** and **3a** with the ratio of 97:3 after preparative HPLC purification. An ICP-MS analysis of several isolated peptides showed that the final gold content was lower than 300 ng/mg (>92% gold removal, see Table S2 in the SI).

**Table 1:**
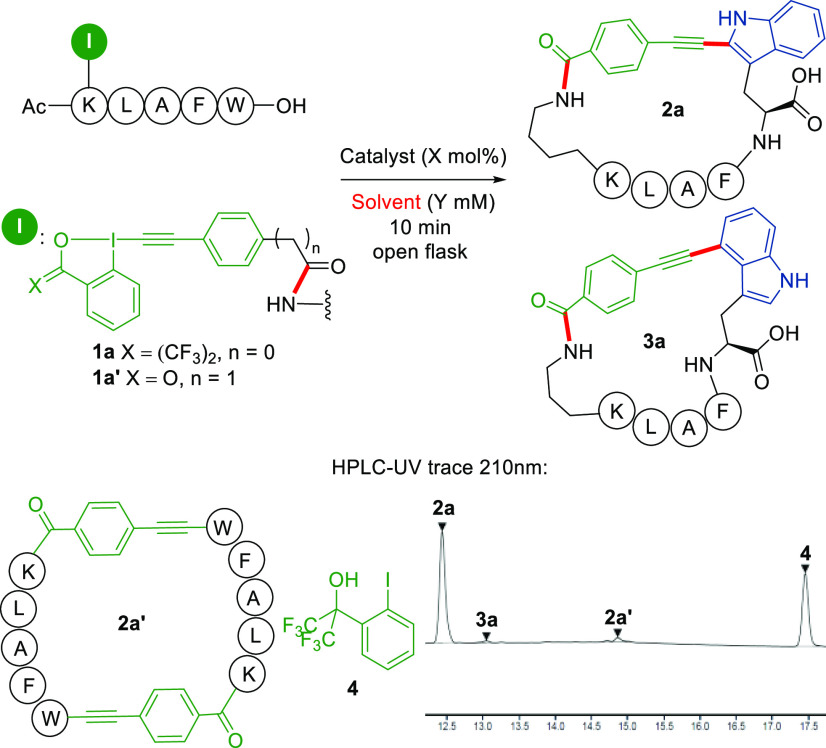
Optimization of the Au-Catalyzed Macrocyclization of Peptide-EBX (**1a**)^a^

entry	catalyst (mol %)	*C* (mM)	solvent	yield^b^
1	AuCl·Me_2_S (100%)	5	DMF	0%
2	AuCl·Me_2_S (100%)	5	DMSO	0%
3	AuCl·Me_2_S (100%)	5	MeCN^c^	64%(99:1)
4	AuCl·Me_2_S (100%)	5	MeOH	69%(95:5)
5	AuCl·Me_2_S (100%)	5	TFE	84%(99:1)
6	AuCl·Me_2_S (100%)	5	HFIP	92%(97:3)
7	AuCl·Me_2_S (50%)	5	HFIP	91%(97:3)
**8**	**AuCl·Me**_**2**_**S (10%)**	**5**	**HFIP**	**90%**(97:3)
9	AuCl·Me_2_S (1%)	5	HFIP	55%^d^(97:3)
10	AuCl·Me_2_S (10%)	2.5	HFIP	64%(97:3)
11	AuCl·Me_2_S (10%)	10	HFIP	66%(97:3)
12	AuCl (100%)	5	HFIP	88%(97:3)
13	AgBF_4_ (100%)	5	HFIP	
14	[Pd]^e^ (100%)	5	HFIP	
15^f^	AuCl·Me_2_S (100%)	5	HFIP	20%(94:6)
16		5	HFIP	

a Conditions: **1a** (1.0 μmol), catalyst (X mol %), solvent (Y mM), 10 min. See Supporting Information for the byproduct analysis.

b HPLC-UV yields are given. The yields were approximated as the ratio of *A*_prod_/*A*_total_ where *A*_prod_ = area in mAU of the product peak and *A*_total_ = area in mAU of all peptides products (product, starting material, and side products if present). The ratio of C2/C4 regioisomers (**2a**:**3a**) is provided in parentheses.

c MeCN mixed with 2% TFA.

d The reaction was run for 16 h; 37% of **1a** was recovered.

e [Pd]: Pd(MeCN)_4_(BF_4_)_2_.

f **1a′** was used instead of **1a**.

After having established the reaction conditions, we investigated the scope of the macrocyclization (Scheme 2). Pentameric peptides AcK-AA-LAFW-OH containing different amino acids, including protected Cys(S-tBu) (**2b**), Asp (**2c**), Glu (**2d**), His (**2e**), Asn (**2f**), Gln (**2g**), Arg (**2h**), Ser (**2i**), and Tyr (**2j**), were examined. The corresponding cyclic peptides were formed in good HPLC conversion (>90%), 25–67% isolated yield, and 87:13–97:3 regioselectivity.

Notably, a terminal alkyne in the uncanonical amino acid propargylic glycine (Pra) can be incorporated into the peptide sequence (**2k**). Unfortunately, only a trace amount of product **2l** was observed in a Met-containing peptide, probably due to coordination of thioether to the gold catalyst. The cyclization of a shorter tripeptide to give compound **2m** is also feasible. This method can be further extended to N-terminal to Trp cyclization. Peptide-EBX sequence **1n** containing the RGD motif, which is responsible for cell adhesion to the extracellular matrix (ECM),^17^ cyclized smoothly to give **2n**. The sequence AFPIPI, which has been shown to have high membrane permeability and oral absorption,^18^ was cyclized efficiently to **2o**. To further highlight the utility of this reaction, several peptide sequences targeting different PPIs were examined. For the DAETGE motif, which has shown good potential to inhibit Keap1–Nrf2 interactions,^19^ the Trp to N-terminus cyclization happened effectively to give cyclic peptides **2p** and **2q**. The cyclic peptide sequence GFFDDLYWFVA has been reported to bind to Lys48-linked Ub chains as a ubiquitination modulator.^12a^ The corresponding linear peptide-EBXs precursor was cyclized smoothly, affording **2r** in 56% yield with excellent regioselectivity. Two MDM2 active peptide sequences each with two Trp residues, allowing either *i*/*i*+3 or *i*/*i*+7 cyclization,^20^ were examined under our conditions. The cyclization reaction mainly occurred in an *i*/*i*+3 manner (**2s**, **2t**), accompanied by minor *i*/*i*+7 products. This methodology can also be applied to cyclization involving N-Me Trp peptides (**2u**–**2w**). In these cases, higher yield and enhanced regioselectivity were observed. These results demonstrated the possibility of using other Trp derivatives for the peptide cyclization. Finally, we attempted the cyclization on the solid phase. Although the hypervalent iodine reagent could be introduced successfully, no desired cyclic peptide was observed after resin cleavage (see Figure S2 for details).

Most peptides do not contain strong fluorophores and therefore cannot be detected easily by fluorescent techniques.^21^ Extra fluorophores must be incorporated onto the peptides. Since the cyclic peptides we obtained contain a highly conjugated aromatic system as the linker, we were wondering if they would display some useful optical properties. Indeed, for peptide **2a**, significant absorption of light from 350 to 400 nm and emission from 400 to 600 nm were observed in DMSO and DMSO/H_2_O (1:4), albeit with lower emission intensity in a DMSO/H_2_O cosolvent system (Scheme 4a). We then wondered whether the peptide macrocycles could be used as fluorescent probes in live-cell imaging. Therefore, we applied our cyclization to several known cell-penetrating peptide sequences (Scheme 4b). PolyTyr^22^ (**2aa**) and polyArg^23a^ (**2ab**–**2ad**) sequences were well-tolerated in the macrocyclization reaction. Importantly, an azide group on the side chain (**2aa**, **2ad**) remained untouched, providing an extra handle for further functionalization (see Figure S3 for the absorption and emission spectra of **2ad**). In addition, the fluorescence decay kinetics of **2ab** were double-exponential with a major lifetime of τ_1_ = 2.12 ± 0.02 ns; see Figures S4–S6).^24^ We then traced the cellular permeabilization of the cyclic peptides using fluorescent microscopy. In order to find out the appropriate concentration for live-cell imaging, an MTT assay (Figure S7) was first conducted to evaluate the cytotoxicity of the cyclic peptides. HeLa cells maintained high cell viability after 16 h of incubation with **2aa**–**2ad** at 10 μM concentration. Therefore, this concentration was used for live-cell imaging. When cells were treated with **2aa**, poor cellular uptake was observed using confocal microscopy (Figure S8), probably due to its poor solubility in cell culture media. To our delight, intracellular fluorescence emission was observed for polyArg cyclic peptides **2ab**–**2ad** under the excitation of a 405 nm laser (Scheme 3c). All peptides exhibited a punctate fluorescence pattern due to enrichment within the endosomal/lysosomal compartments. (See Figure S9 for the co-localization with LysoTracker.^23b,23c^) These results demonstrated the potential use of macrocyclic peptides for imaging studies without further modifications.

**Scheme 3:**
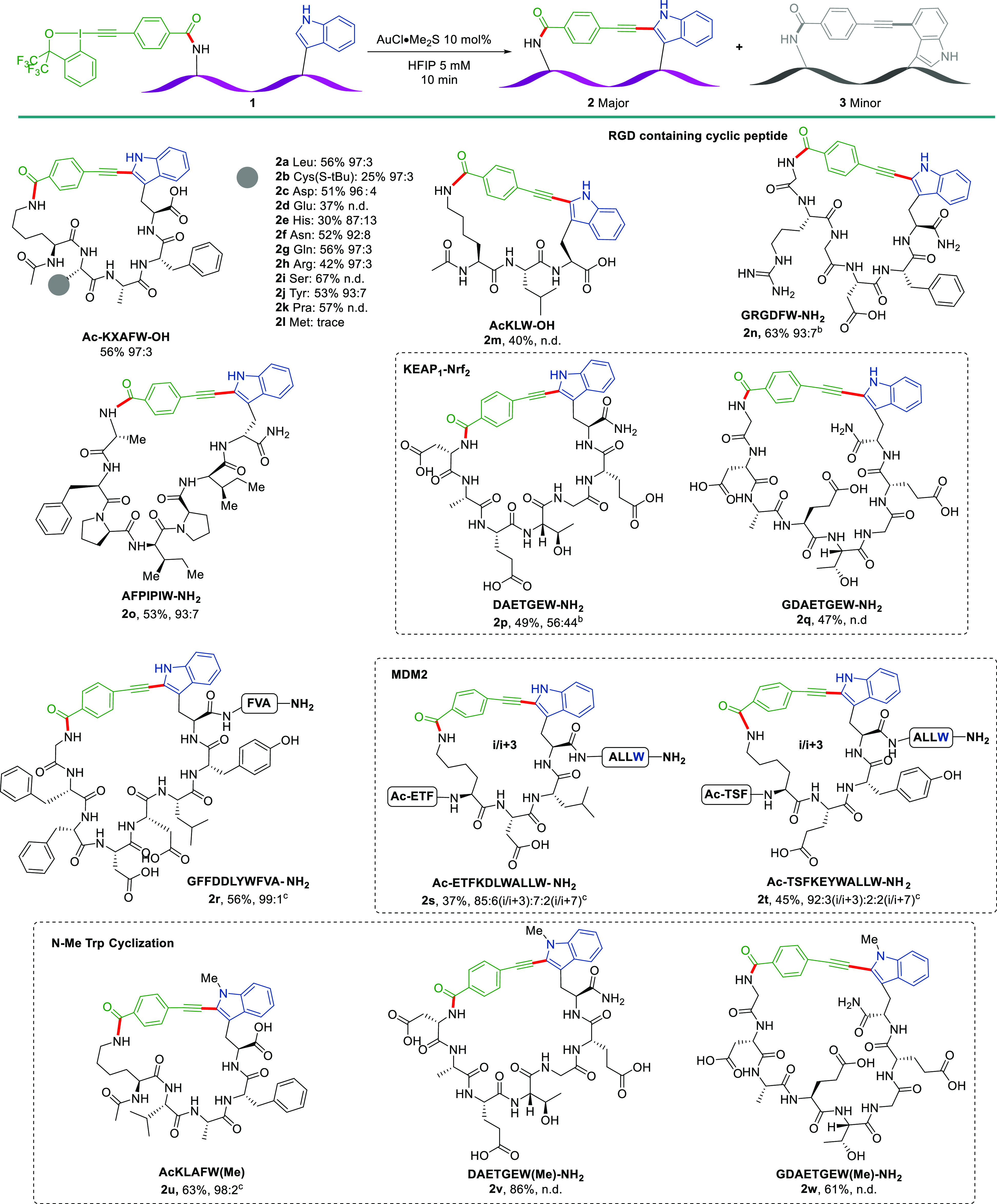
Scope of the Gold(I)-Catalyzed Peptide Macrocyclization The reactions were performed on 0.01 mmol. Isolated yields are given; the ratio of C2 and C4 regioisomers **2**/**3** was determined by HPLC-UV ratio. n.d.: not resolved by HPLC/UV. Full experimental details are provided in the SI. The two regioisomers were isolated separately. Only a single regioisomer was isolated.

**Scheme 4:**
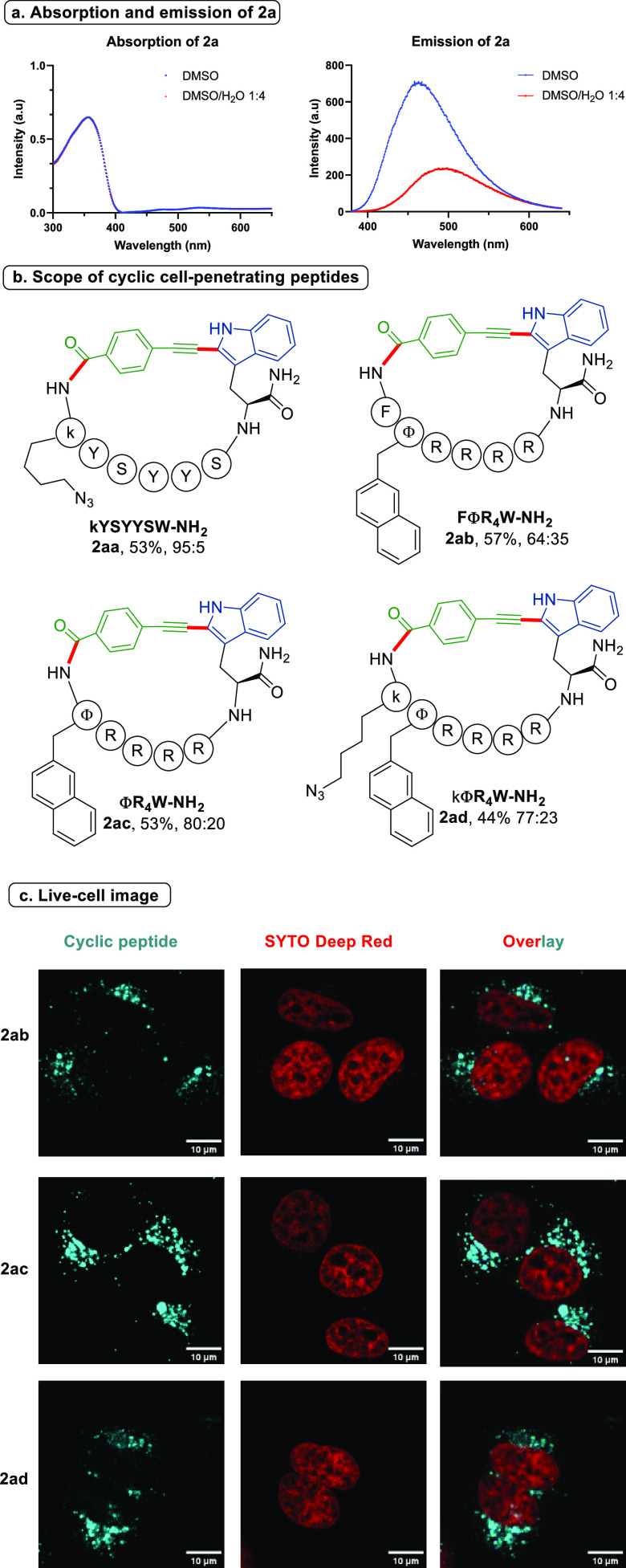
(a) Absorption and Emission of Cyclic Peptide **2a** in DMSO and DMSO/H_2_O (1:4) (20 μΜ); (b) Scope of Cell Penetrating Peptides; (c) Live-Cell Images of HeLa Cells after 3 h Incubation of 10 μΜ **2ab**, **2ac**, and **2ad** by Using a Confocal Spinning Disk Microscope Isolated yields are given; the ratio of C2 and C4 regioisomers **2**/**3** was determined by HPLC-UV. The nucleus was stained by SYTO Deep Red at 1 μM (scale bar: 10 μm).

In conclusion, we have developed the first gold(I)-catalyzed macrocyclization of peptide-EBXs via Trp C_2_ C–H activation. This intramolecular alkynylation reaction proceeded in a fast, mild, and efficient way at room temperature with unprotected linear peptide-EBXs. The unique aromatic linker formed during the reaction allowed us to realize live-cell visualization without further installation of other fluorophores. We envision that the optical properties of our fluorescent linkers can be improved by extending the aromatic system on the alkyne partner or by adding electron donor groups on Trp to form better donor–acceptor systems.^25^

## Data Availability

Raw NMR, MS, IR, fluorescence, and imaging data are available at zenodo.org: 10.5281/zenodo.10124981.

## Supporting Information Available

General procedures, HPLC methods, synthesis procedures, and characterization data for all compounds (PDF)

## ABBREVIATIONS

EBX: ethynylbenziodoxolone
PPIs: protein–protein interactions
