# Supplementary material for: Synthesis of Fluorescent Cyclic Peptides via Gold(I)-Catalyzed Macrocyclization
Source: J Am Chem Soc. 2023 Nov 30;145(49):26525–31. doi: 10.1021/jacs.3c09261 (PMC10722513; doi:10.1021/jacs.3c09261)

Supporting Information

Synthesis of Fluorescent Cyclic Peptides via Gold(I)-Catalyzed Macrocyclization

Xing-Yu Liu, Wei Cai, Nathan Ronceray, Aleksandra Radenovic, Beat Fierz, Jerome Waser*

Table of Contents

1. General procedures 2

2. HPLC-MS and preparative HPLC information 3

HPLC-MS analysis 3

Preparative HPLC 3

Solid-Phase Peptide Synthesis (SPPS): 3

MS/MS fragmentation: 4

3. Synthesis of bifunctional alkynylation reagents 5

4. Scope of peptide-EBXs 7

5. General procedure for synthesis of peptide-EBXs 8

6. Condition screening for the Au(I)-catalyzed peptide-EBXs cyclization 60

6.1 Calibration of the cyclization reaction 60

6.2 Reaction optimization: 60

6.3 Scope of cyclization 62

6.4 Attempt of cyclization on solid phase 94

6.5 ICP analysis of cyclic peptides 96

7. Studies of the structure of two regioisomers: 97

7.2 Cyclization under different reaction temperature: 106

8. Absorption and emission of cyclic peptides 107

9. Excited state lifetime of 2ab 108

10. Cell experiments 111

11. NMR spectra 113

1. General procedures

All reactions using anhydrous conditions were performed with oven-dried glassware, under an atmosphere of nitrogen, unless stated otherwise. Tetrahydrofuran, acetonitrile, diethyl ether and dichloromethane (DCM) were dried by passage over activated alumina, under nitrogen atmosphere, on an Innovative Technology Solvent Delivery System (water content < 10 ppm, Karl-Fischer titration). Dichloroethane and ethanol were purchased from Acros and trifluoroethanol was purchased from Fluorochem. DMSO was purchased from Sigma-Aldrich. All the Fmoc-protected amino acids (including the non-canonical ones) and Rink Amide MBHA resin were purchased from GL Biochem or Bachem. 1-[Bis(dimethylamino)methylene]-1H-1,2,3-triazolo[4,5-b]pyridinium 3-oxide hexafluorophosphate (HATU, Bachem) and N,N-diisopropylethylamine (DIPEA, Iris Biotech GmbH) were used as received. All the other reagents were purchased from ABCR, Acros, AlfaAesar, Apollo Scientific, Fluorochem, Fluka, Roth, Sigma-Aldrich and TCI and were used as such. For flash chromatography, distilled technical grade solvents were used. Chromatographic purification was performed as flash chromatography using Macherey-Nagel silica 40-63, 60 Å, using the solvents indicated as eluent with 0.1 – 0.5 bar pressure. TLC was performed on Merck silica gel 60 F254 TLC aluminum or glass plates and visualized with UV light or permanganate stain. Melting points were measured on a Büchi B-540 melting point apparatus using open glass capillaries. ^{1}H-NMR spectra were recorded on a Brucker DPX-400 400 MHz spectrometer in $CDCl_3$, DMSO-d_6, CD_3OD, or D_2O. All signals are reported in ppm with the internal $CHCl_3$ signal at 7.26 ppm, the internal DMSO signal at 2.50 ppm and CD_3OD as 3.35 ppm as standard. The data is being reported as: s = singlet, d = doublet, t = triplet, q = quadruplet, qi = quintet, m = multiplet or unresolved, br = broad signal, app = apparent, coupling constant(s) in Hz, integration, interpretation.^{13}C-NMR spectra were recorded with 1H-decoupling on a Brucker DPX-400 100 MHz spectrometer in $CDCl_3$, DMSO-d_6 or CD_3OD. All signals are reported in ppm with the internal $CHCl_3$ signal at 77.16 ppm or the internal DMSO signal at 39.52 ppm as standard. Spectra were fully assigned using COSY, HSQC, HMBC and ROESY. High-resolution mass spectrometric measurements were performed by the mass spectrometry service of ISIC at the EPFL on LTQ Orbitrap ELITE ETD (Thermo fisher), Xevo G2-S QTOF (Waters), or LTQ Orbitrap ELITE ETD (Thermo fisher). UV/Vis spectroscopy was performed on an Agilent Cary 60 UV-Vis and steady-state luminescence spectroscopy was recorded on a Varian Cary Eclipse spectrophotometer. ICP analysis was performed by the mass spectrometry service of ISIC at the EPFL using ICP-MS Nexlon 350 (Perkin Elmer).

2. HPLC-MS and preparative HPLC information

HPLC-MS analysis

HPLC-MS measurements were performed on an Agilent 1290 Infinity HPLC system with a G4226a 1290 Autosampler, a G4220A 1290 Bin Pump and a G4212A 1290 DAD detector, connected to a 6130 Quadrupole LC/MS, coupled with a Waters XBridge C18 column (250 x 4.6 mm, 5 µm). Water:acetonitrile 95:5 (solvent A) and water:acetonitrile 5:95 (solvent B), each containing 0.1% formic acid, were used as the mobile phase, at a flow rate of 0.6 $mL.min^{-1}$. The gradient was programmed as follows:

Method 1: 100% A to 100% B in 20 minutes then isocratic for 5 minutes.

The column temperature was set up to 25 °C. Low-resolution mass spectrometric measurements were acquired using the following parameters: positive electrospray ionization (ESI), temperature of drying gas = 350 °C, flow rate of drying gas = 12 L. min^{-1}, pressure of nebulizer gas = 60 psi, capillary voltage = 2500 V and fragmentor voltage = 70 V.

Preparative HPLC

Preparative RP-HPLC were performed on an Agilent 1260 HPLC system with a G2260A 1260 Prep ALS Autosampler, a G1361a 1260 Prep Pump, a G1365C 1260 MWD detector and a G1364B 1260 FC-PS collector, coupled with a Waters XBridge semi-preparative C18 column (19 x 150 mm, 5 µm). Water (solvent A) and water:acetonitrile 5:95 (solvent B), each containing 0.1% TFA, were used as the mobile phase at a flow rate of 20 $mL.min^{-1}$.

Method 2: 100% A to 100% B in 20 minutes then isocratic for 5 minutes.

Method 3: 100% A to 100% B in 25 minutes then isocratic for 5 minutes.

Solid-Phase Peptide Synthesis (SPPS):

Peptides were synthesized on an MultiPep RSi parallel peptide synthesizer (Intavis) using standard Fmoc SPPS-chemistry, 2-chlorotrityl chloride resin (1.38 mmol/g, 100-200 mesh) and Rink-amide resin (0.33 mmol/g). For 2-chlorotrityl chloride resin, the first amino acid was loaded on the resin by incubation of the Fmoc-protected monomer (3 equiv of the number of active sites on the resin), DIPEA (4 equiv) in dichloromethane for 2 h. Each coupling cycle was initiated by Fmoc deprotection achieved by shaking the resin with 800 µL of 20% v/v piperidine in dimethylformamide (DMF) at 400 rpm, over 5 minutes twice. Then the resin was washed with DMF (6000 µL x7). The coupling was carried out by shaking resin with a Fmoc-protected monomer (4.0 equiv.), HATU (4.0 equiv.), *N*-Methylmorpholine (6.0 equiv.), in DMF (1.3 mL), at 400 rpm, over 30 minutes twice. Capping using Cap Mixture (5% v/v Ac_2O and 6% v/v 2,6-lutidine in DMF) was carried out at the end of each cycle, followed by a DMF wash (6000 µL x7). The synthesis was finished by deprotection of Fmoc using 20% v/v piperidine in dimethylformamide at 400 rpm, over 5 minutes two times. The N-terminus was either left unprotected or was acylated. Acetylation of the N-terminal was achieved by incubating the resin with Cap Mixture three times. Next, washing steps were performed with dimethylformamide (5 x 3 mL). Finally, resin was dried with dichloromethane (5 x 3 mL).

Peptide cleavage and deprotection:

Peptides without protecting groups

Peptides were deprotected and cleaved from the resin by treatment with 2.5% v/v water and 2.5% v/v Triisopropyl silane in neat trifluoroacetic acid (2 mL) (Note: For polyArg sequence, reagent R (TFA:thioanisole:EDT:anisole 90:5:3:2) was used). The resulting mixture was shaken for 2 hours, at room temperature. The resin was removed by filtration and peptides were precipitated in cold diethyl ether (50 mL), followed by a 2 hours incubation at -20 °C. Peptides were pelleted by centrifugation at 4000 rpm, for 5 minutes. Finally, the mother liquors were carefully removed.

The precipitations were further dissolved in water and acetonitrile, shell freeze and lyophilize to yield the desired crude peptides. If necessary, preparative HPLC purification was carried out.

Peptide analysis:

MS/MS fragmentation:

The regioselectivity of the introduction of EBX onto peptides was confirmed using MS/MS analysis. The spectra were obtained by the mass spectrometry service of ISIC at the EPFL using Thermo Orbitrap Elite instrument. The desired ion was selected using mass filters and submitted to fragmentations. The obtained data was analyzed using fragment generation program on eln.epfl.ch.[1] For the calculations peak threshold for intensity was set to 0.5% and 0.03% for quantity, precision was set to 5 ppm and minimal similarity: 70%. The peaks were compared to theoretical peaks. The theoretical peak width was calculated from the mass of the ion by the formula provided in the script. The zone was set to -0.5 to 3.5 ppm. y and b fragments with and without linker were selected and reported. In the cases were fragmentation was low, c and z fragments and/or fragments arising from neutral losses were included.

[1] a) Desport, J.S., Frache, G., and Patiny, L. (2020), MSPolyCalc: A web-based App for polymer mass spectrometry data interpretation. The case study of a pharmaceutical excipient. Rapid Commun. Mass Spectrom. *34*, e8652.; b) Ortiz, D., Gasilova, N., Sepulveda, F., Patiny, L., Dyson, P.J., and Menin, L. (2020), Aom2S: A new web-based application for DNA/RNA tandem mass spectrometry data interpretation. Rapid Commun. Mass Spectrom. *34*, e8927.

3. Synthesis of bifunctional alkynylation reagents

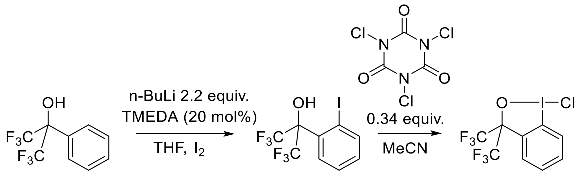

S1 S2 S3

Following a reported procedure,[2] TMEDA (0.900 mL, 6.00 mmol, 0.200 equiv) was added to a solution of n-BuLi (2.5 M in hexanes, 26.4 mL, 66.0 mmol, 2.20 equiv). After 15 min, the cloudy solution was cooled to 0 °C and **S1** (5.05 mL, 30.0 mmol, 1 equiv) in THF (6 mL) was added dropwise. The reaction was stirred 30 min at 0 °C and then at RT for 6 hours. I_2 (7.95 g, 31.5 mmol, 1.05 equiv) was then added portionwise at 0 °C and the mixture stirred at 0 °C for 30 min at RT. The reaction was quenched with saturated $NaSO_3$ (20 mL) and the layers were separated. The aqueous layer was then extracted twice with EtOAc (3 x 50 mL). The organic layers were combined, dried over Na_2SO_4, filtered and concentrated under reduced pressure to afford 12.4 g as a brown oil which was used without further purification. The crude oil was dissolved in MeCN (40 mL) in the dark under air. Trichloroisocyanuric acid (2.44 g, 10.5 mmol, 0.350 equiv.) was then added portionwise at RT. After 30 min, the resulting suspension was filtered to afford **S3** (6.12 g, 15.1 mmol, 50%) as a yellow solid. ^1H NMR (400 MHz, $CDCl_3$) δ 8.09 (d, J = 8.5 Hz, 1H), 7.88 – 7.81 (m, 1H), 7.75 – 7.70 (m, 2H). The ^1H NMR correspond to the reported values.

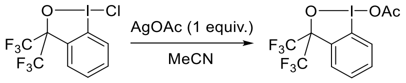

S3 S4

Following a reported procedure,[2] 1-Chloro-1,3,-dihydro-3,3-bis(trifluoromethyl)-1,2-benziodoxole **S3** (6.12 g, 15.2 mmol) and AgOAc (2.54 g, 15.2 mmol, 1.00 equiv.) were suspended in MeCN (60 mL, 0.25 M). After being stirred overnight in the dark, AgCl precipitated and was filtered off. The residue was washed with MeCN. The solvent was removed in vacuo to give **S4** (4.90 g, 11.5 mmol, 75%) as a white solid. ^1H NMR (400 MHz, $CDCl_3$) δ 7.96 (dd, J = 8.3, 1.1 Hz, 1H), 7.80 (ddd, J = 8.5, 7.1, 1.6 Hz, 1H), 7.75 (d, J = 7.9, 1H), 7.70 – 7.63 (m, 1H), 2.21 (s, 3H). The NMR values correspond to the reported ones.

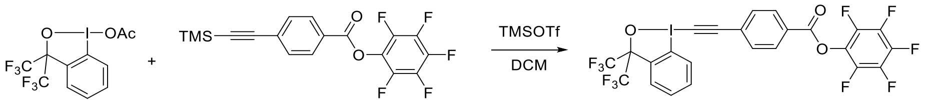

S4 S5 S6

To a solution of 3,3-bis(trifluoromethyl)-1λ3 -benzo[d][1,2]iodaoxol-1(3H)-yl acetate (**S4**) (2.14 g, 5.00 mmol, 1.00 equiv.) in dry DCM (25 ml) was added trimethylsilyl trifluoromethanesulfonate (0.998 mL, 5.50 mmol, 1.10 equiv.) dropwise at room temperature and the reaction mixture was stirred for 1 h. After this time, **S5** (2.11 g, 5.50 mmol, 1.10 equiv.) was added and the mixture was stirred for 4 h at room temperature. The reaction mixture was then quenched with saturated aqueous $NaHCO_3$ solution and extracted with dichloromethane (3 times). The combined organic extracts were dried over Na_2SO_4, filtered and concentrated under reduced pressure. The crude solid was washed by Et_2O to afford the desired product as

[2] Caramenti, P.; Nandi, R. K.; Waser, J., Metal-Free Oxidative Cross Coupling of Indoles with Electron-Rich (Hetero)arenes. *Chem.–Eur. J.* **2018,** *24* (40), 10049-10053.

colorless solid (2.70 g, 3.97 mmol, 79%). The synthesis of **S6** can be achieved using a recent one-pot approach from **S1**.[3]

^{1}H NMR (500 MHz, $CDCl_3$) δ 8.28 – 8.25 (m, 1H, ArH), 8.23 (d, *J* = 8.4 Hz, 2H, ArH(phenylacetylene)), 7.88 (d, *J* = 7.8 Hz, 1H, ArH), 7.76 – 7.73 (m, 2H, ArH), 7.71 (d, *J* = 8.4 Hz, 2H, ArH(phenylacetylene)).

^{13}C NMR (126 MHz, $CDCl_3$) δ 161.9, 141.5 (dm, *J* = 239.6 Hz, C_{Ar}-F), 138.1 (dm, *J* = 253.5 Hz, C_{Ar}-F), 138.1 (dm, *J* = 253.5 Hz, C_{Ar}-F), 133.3, 133.0, 131.6, 131.0, 130.2, 130.1, 128.5, 127.9, 127.9, 125.4 – 125.0 (m, C_{Ar}-O), 123.6 (q, *J* = 288.5Hz, CF_3), 111.4, 103.3, 82.38 – 81.35 (m), 60.1.

HRMS (nanochip-ESI/LTQ-Orbitrap) m/z: $[M + H]^+$ Calcd for $C_{24}H_9F_{11}IO_3^+$ 680.9415; Found 680.9393.

[3] Milzarek, T. M.; Ramirez, N. P.; Liu, X.-Y.; Waser, J., One-pot synthesis of functionalized bis(trifluoromethylated)benziodoxoles from iodine(i) precursors. *Chem. Commun.* **2023**. doi: 10.1039/D3CC04525K.

4. Scope of peptide-EBXs

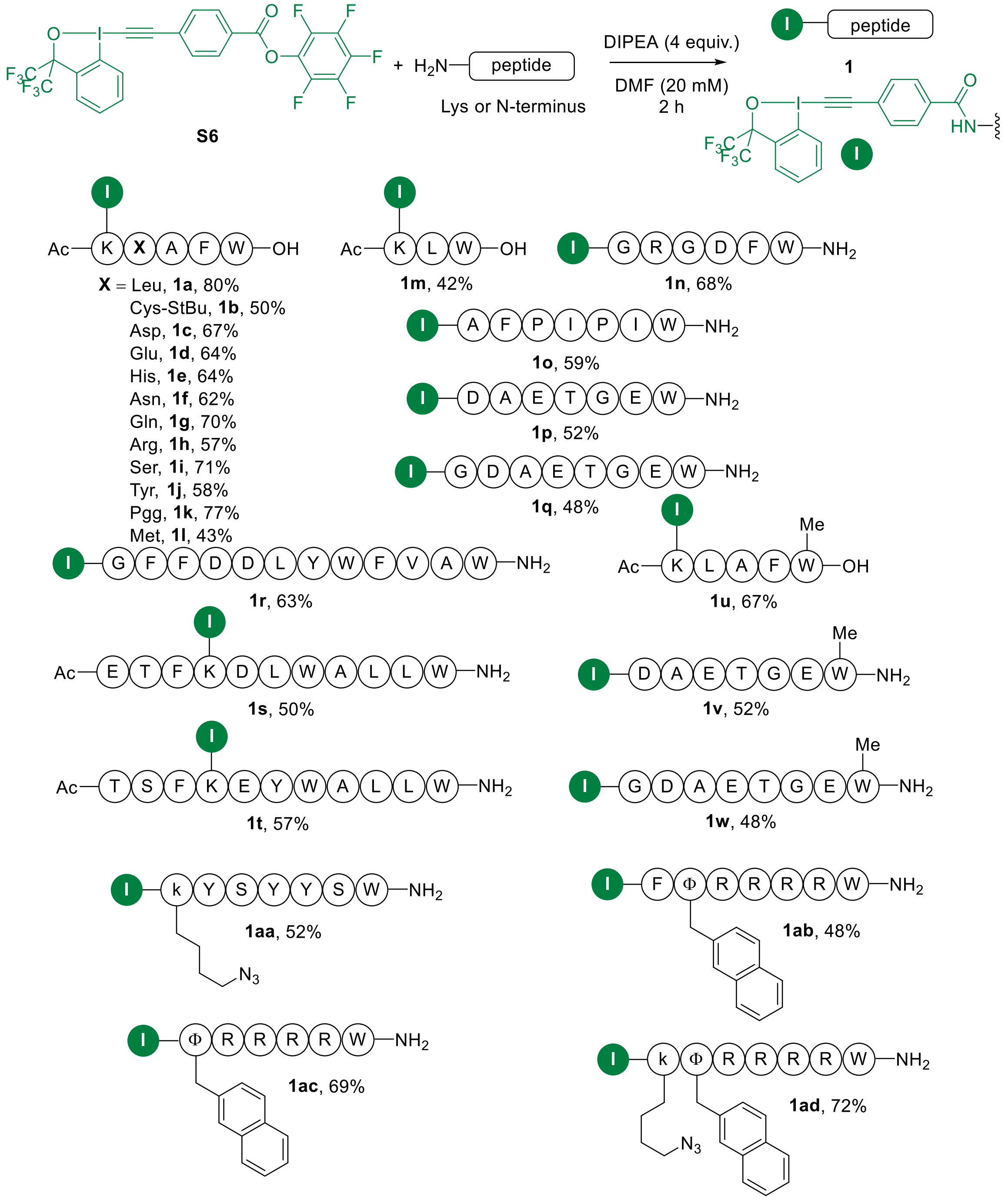

Isolated yields are given.

Figure S1: Scope of peptide-EBXs

5. General procedure for synthesis of peptide-EBXs

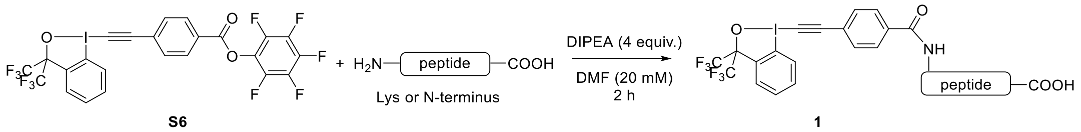

To a solution of peptide (0.03 mmol), bifunctional EBX reagent (0.033 mmol, 1.1 equiv.) in DMF (1.5 mL), DIPEA (20 µL, 0.12 mmol, 4 equiv.) was added into the solution (concentration: 20 mM) and the mixture was stirred for 2 hours without protection of atmosphere or light. For the isolation, the crude was subjected to Prep-HPLC without dilution, followed by lyophilization.

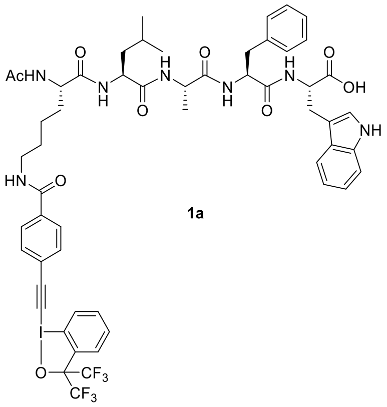

1a

Following the general procedure, the reaction was conducted in 0.041 mmol scale. The desired product **1a** (39.6 mg, 0.0330 mmol, 80% yield) was isolated by **Method 2**.

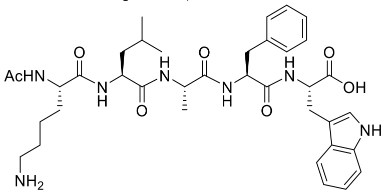

HPLC-UV chromatogram (210 nm) of **AcKLAFW-OH**:

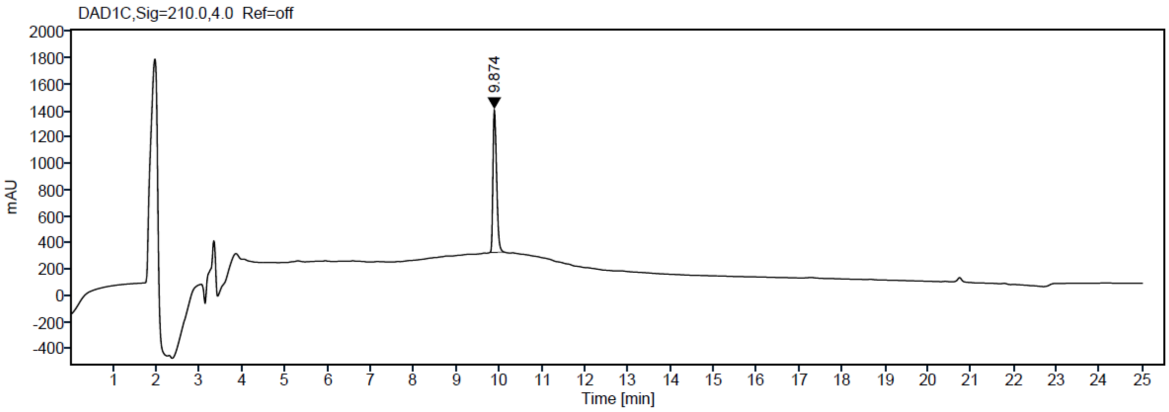

Retention time: 9.903 min **Area Percent:** 100%

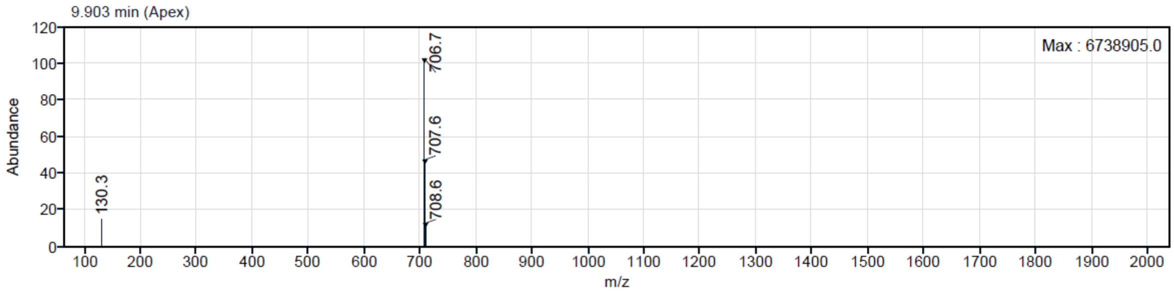

HPLC-UV chromatogram (210 nm) of **1a**:

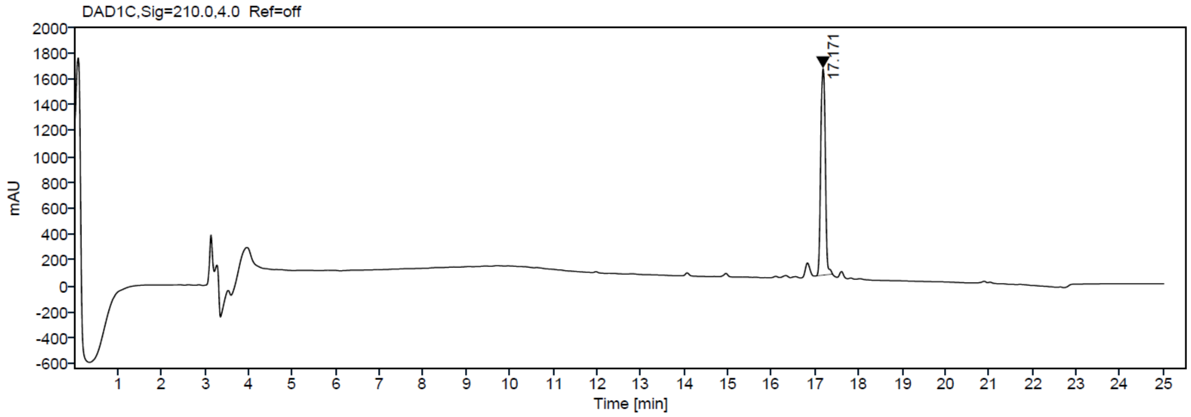

Retention time: 17.216 min **Area Percent:** 100%

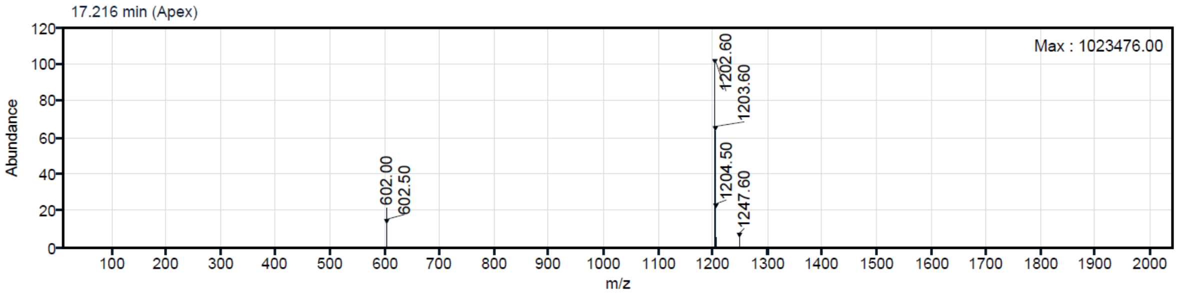

^{1}H NMR (500 MHz, MeOD) δ 8.41 – 8.35 (m, 1H, ArH), 7.89 – 7.83 (m, 3H, ArH(Trp+phenylacetylene)), 7.81 – 7.76 (m, 2H, ArH), 7.68 (d, *J* = 8.3 Hz, 2H, ArH(phenylacetylene)), 7.52 (d, *J* = 7.9 Hz, 1H, ArH(Trp)), 7.30 (d, *J* = 8.1 Hz, 1H, ArH(Trp)), 7.17 (m, 5H, ArH(Phe)), 7.08 (s, 1H, ArH(Trp C2)), 7.06 (t, *J* = 7.6 Hz, 1H, ArH(Trp)), 6.99 (t, *J* = 7.4 Hz, 1H, ArH(Trp)), 4.71 – 4.65 (m, 1H), 4.61 – 4.54 (m, 1H), 4.34 (dt, *J* = 10.5, 5.5 Hz, 1H), 4.26 (dd, *J* = 8.1, 5.9 Hz, 1H), 4.20 (q, *J* = 7.0 Hz, 1H), 3.45 (m, 1H), 3.39 (m, 2H), 3.22 – 3.15 (m, 1H), 3.10 (dd, *J* = 14.0, 5.4 Hz, 1H), 2.88 (dd, *J* = 14.0, 8.6 Hz, 1H), 1.98 (s, 3H), 1.88 – 1.77 (m, 1H), 1.75 – 1.58 (m, 4H), 1.59 – 1.53 (m, 2H), 1.47 (dd, *J* = 16.1, 8.3 Hz, 2H), 1.20 (d, *J* = 7.3 Hz, 3H, CH_3(Ala)), 0.90 (dd, *J* = 24.8, 6.5 Hz, 6H, CH_3(Leu)).
^{13}C NMR (126 MHz, MeOD) δ 174.8, 174.7, 174.5, 174.4, 173.6, 173.0, 169.0, 138.3, 138.0, 137.0, 134.6, 133.7, 132.5, 131.8, 130.9, 130.4, 129.4, 128.7, 127.7, 126.3, 125.6, 124.6, 122.4, 119.8, 119.3, 112.4, 112.3, 110.8, 104.9, 56.1, 55.8, 55.2, 54.7, 53.3, 50.5, 41.5, 40.8, 38.7, 32.5, 30.0, 28.5, 25.9, 24.3, 23.5, 22.4, 21.8, 18.0.
HRMS (nanochip-ESI/LTQ-Orbitrap) m/z: $[M + H]^+$ Calcd for $C_{54}H_{61}IN_7O_{10}^+$ 1094.3519; Found 1094.3447.

MS/MS fragmentation of **1a**:

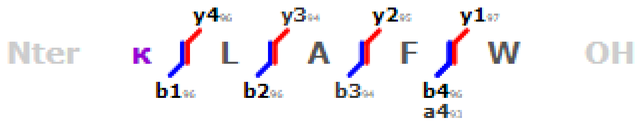

κ = Lys(C18H7F6IO2)
Nter = C2H3O

Sequence	Type	MF	MF Mass	m/z	Intensity	Similarity
KLAF	b4	C44H47F6IN5O7(+1)	998.2424	998.2419	101.33	97.69%
W	y1	C11H13N2O2(+1)	205.0977	205.0972	23.72	96.91%
LAFW	y4	C29H38N5O5(+1)	536.2873	536.2867	12.53	95.84%
K	b1	C26H22F6IN2O4(+1)	667.0528	667.0523	17.24	95.78%
KL	b2	C32H33F6IN3O5(+1)	780.1369	780.1364	11.39	95.55%
KLA	b3	C35H38F6IN4O6(+1)	851.174	851.1735	43	95.15%
KLAF	b4	C44H47F6IN5O7(+1)	998.2424	499.6246	25.58	95.10%
FW	y2	C20H22N3O3(+1)	352.1661	352.1656	36.65	95.03%
AFW	y3	C23H27N4O4(+1)	423.2032	423.2027	4.71	94.18%
KLAF	a4	C43H47F6IN5O6(+1)	970.2475	970.247	3.72	92.96%
KLA	b3	C35H38F6IN4O6(+1)	851.174	426.0904	2.05	92.10%

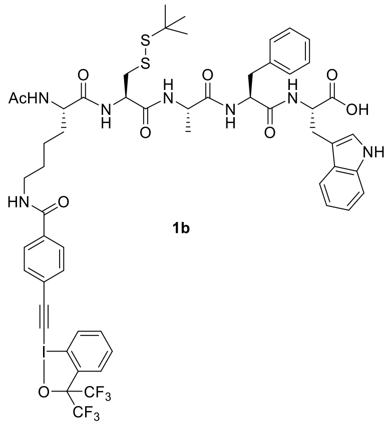

1b

Following the general procedure, the reaction was conducted on 0.02 mmol scale. The desired product **1b** (12 mg, 0.010 mmol, 50% yield) was isolated by **Method 2**.

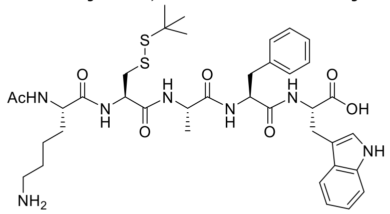

HPLC-UV chromatogram (210 nm) of **AcKC(S-tBu)AFW-OH**:

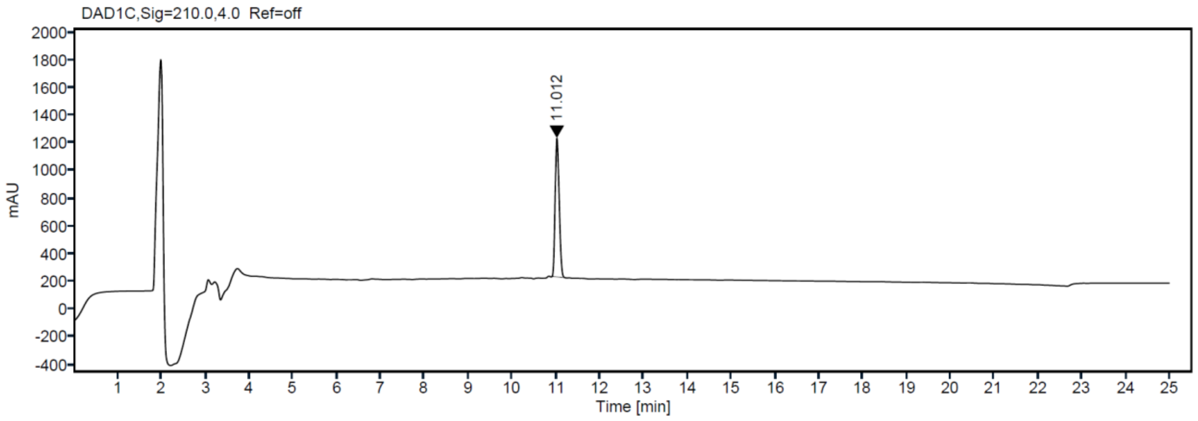

Retention time: 11.039 min **Area Percent:** 100%

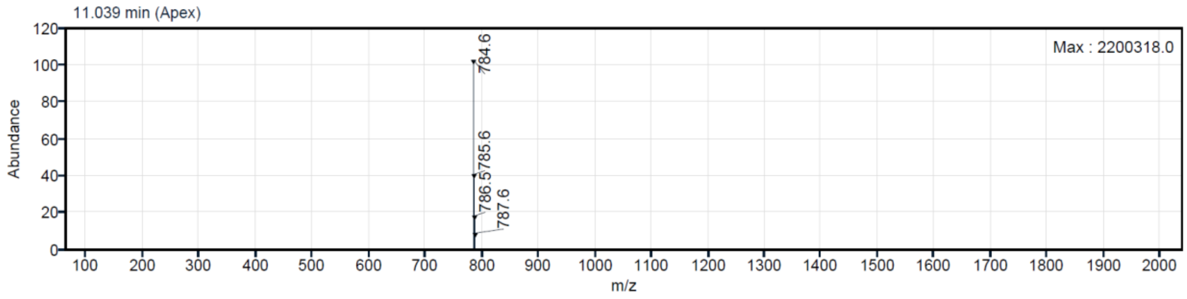

HPLC-UV chromatogram (210 nm) of **1b**:

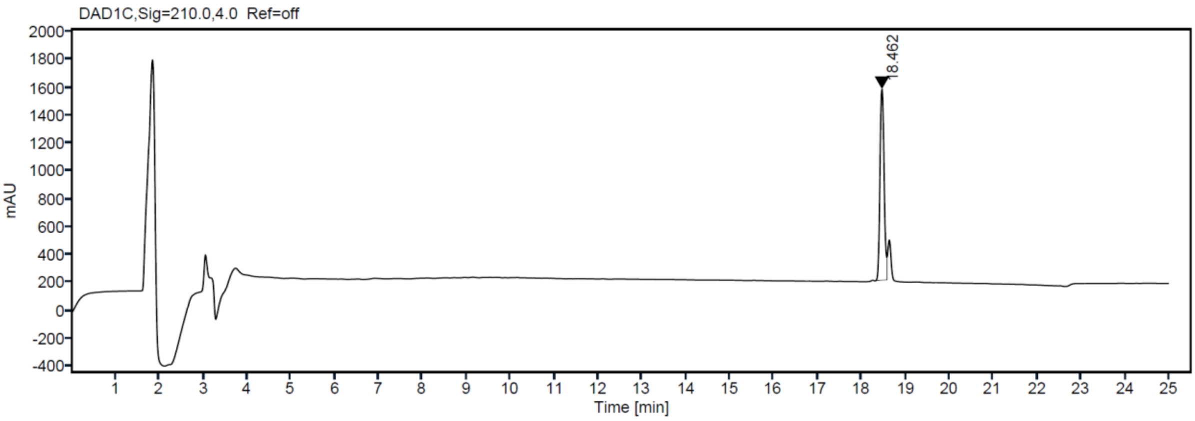

Retention time: 18.495 min **Area Percent:** 100%

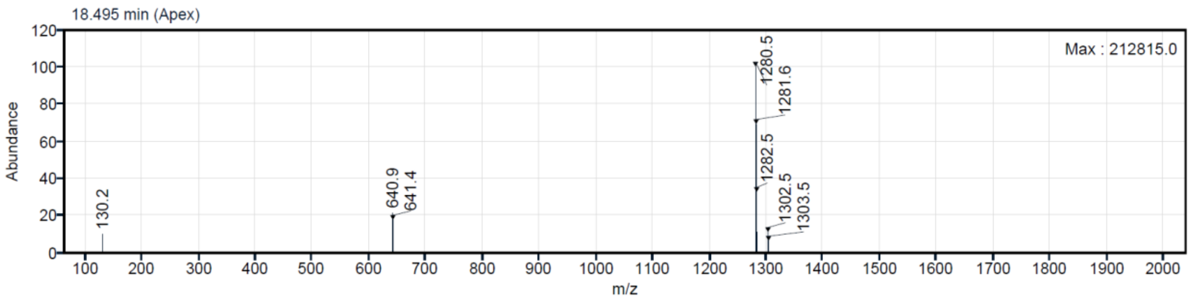

HRMS (nanochip-ESI/LTQ-Orbitrap) m/z: $[M + H_2]^{+2}$ Calcd for $C_{56}H_{62}F_6IN_7O_9S_2{}^{+2}$ 640.6494; Found 640.6476.

MS/MS fragmentation of **1b**:

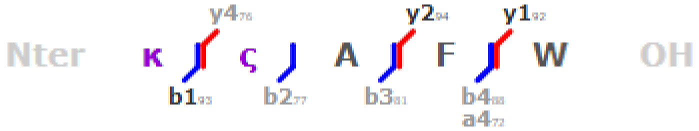

κ = Lys(C18H7F6IO2)
ç = Cys(SC4H8)
Nter = C2H3O

Sequence	Type	MF	MF Mass	m/z	Intensity	Similarity
FW	y2	C20H22N3O3(+1)	352.17	352.17	24.78	94.12%
K	b1	C26H22F6IN2O4(+1)	667.05	667.05	13.78	92.79%
W	y1	C11H13N2O2(+1)	205.1	205.1	14.82	91.87%
KCAF	b4	C45H49F6IN5O7S2(+1)	1076.2	1076.2	100.1	90.05%
KCA	b3	C36H40F6IN4O6S2(+1)	929.13	929.13	30.33	86.76%
KCAF	b4	C45H49F6IN5O7S2(+1)	1076.2	538.6	35.92	86.45%
KC	b2	C33H35F6IN3O5S2(+1)	858.1	858.1	3.62	77.11%
CAFW	y4	C30H40N5O5S2(+1)	614.25	614.25	8.19	75.98%
KCA	b3	C36H40F6IN4O6S2(+1)	929.13	465.07	4.72	75.84%
KCAF	a4	C44H49F6IN5O6S2(+1)	1048.2	1048.2	4.84	72.48%

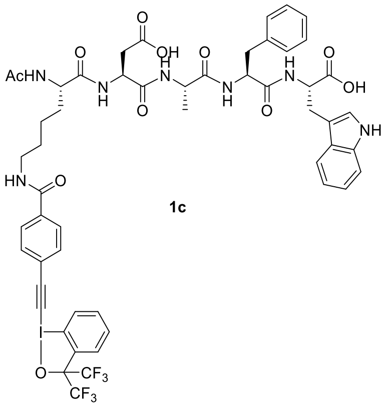

1c

Following the general procedure, the reaction was conducted in 0.03 mmol scale. The desired product **1c** (24.1 mg, 0.0201 mmol, 67% yield) was isolated by **Method 2**.

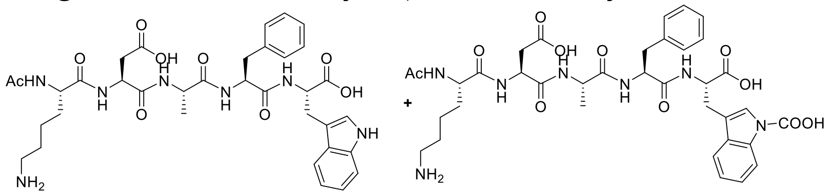

HPLC-UV chromatogram (210 nm) of **AcKDAFW-OH**:

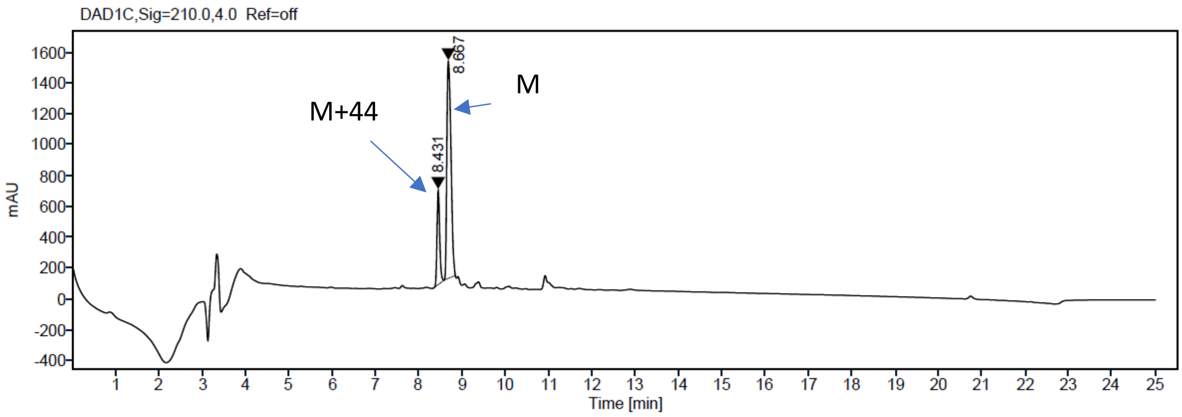

Retention time: 8.459 min **Area Percent:** 23%

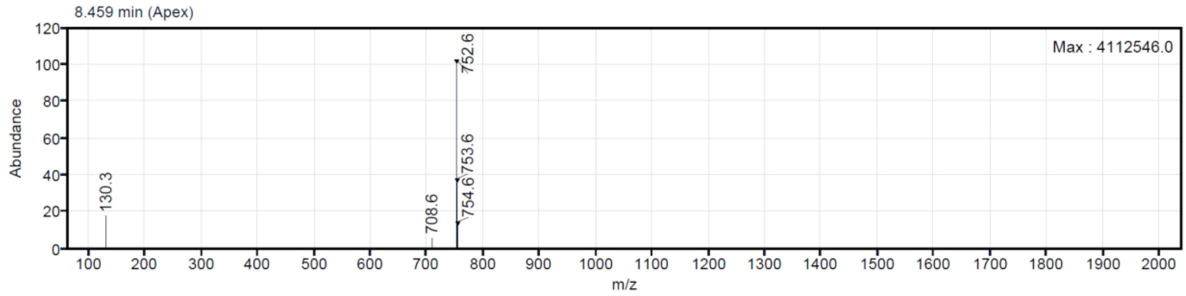

Retention time: 8.684 min **Area Percent:** 77%

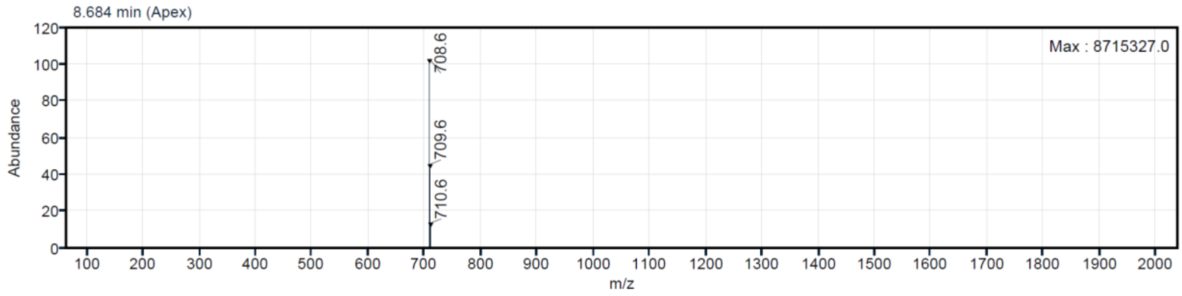

HPLC-UV chromatogram (210 nm) of **1c**:

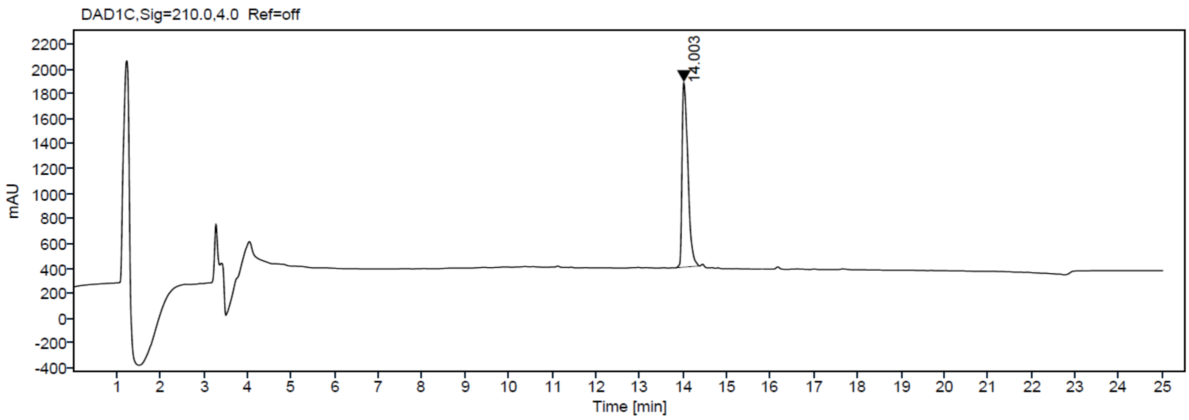

Retention time: 16.079 min **Area Percent:** 100%

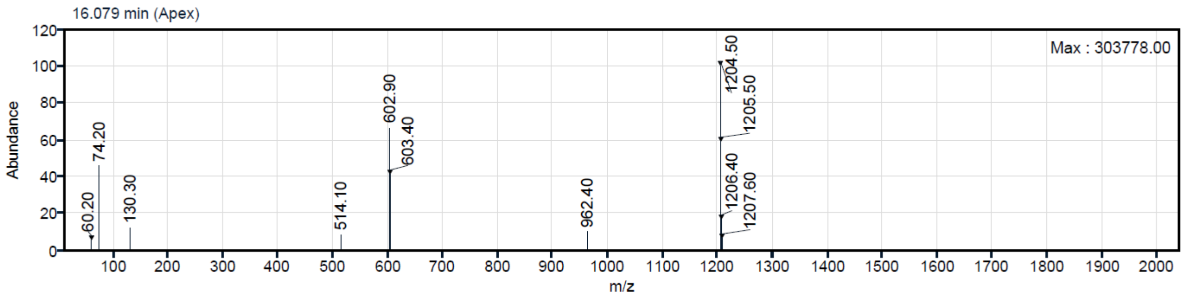

HRMS (nanochip-ESI/LTQ-Orbitrap) m/z: $[M + H]^+$ Calcd for $C_{53}H_{53}F_6IN_7O_{11}^+$ 1204.2746; Found 1204.2773.

MS/MS fragmentation of **1c**:

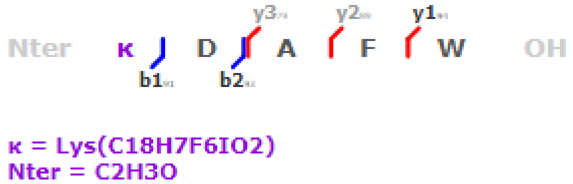

Sequence	Type	MF	MF Mass	m/z	Intensity	Similarity
W	y1	C11H13N2O2(+1)	205.0977	205.0972	20.56	94.14%
KD	b2	C30H27F6IN3O7(+1)	782.0798	782.0792	21.7	92.99%
K	b1	C26H22F6IN2O4(+1)	667.0528	667.0523	15.8	90.89%
FW	y2	C20H22N3O3(+1)	352.1661	352.1656	9.18	89.25%
AFW	y3	C23H27N4O4(+1)	423.2032	212.105	100.13	78.64%

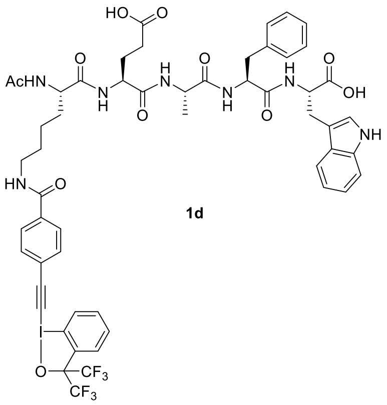

1d

Following the general procedure, the reaction was conducted in 0.03 mmol scale. The desired product **1d** (23.5 mg, 0.0193 mmol, 64% yield) was isolated by **Method 2**.

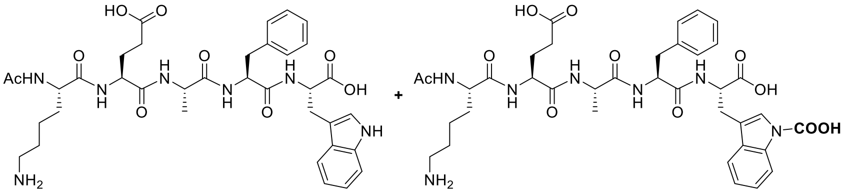

HPLC-UV chromatogram (210 nm) of **AcKEAFW-OH**:

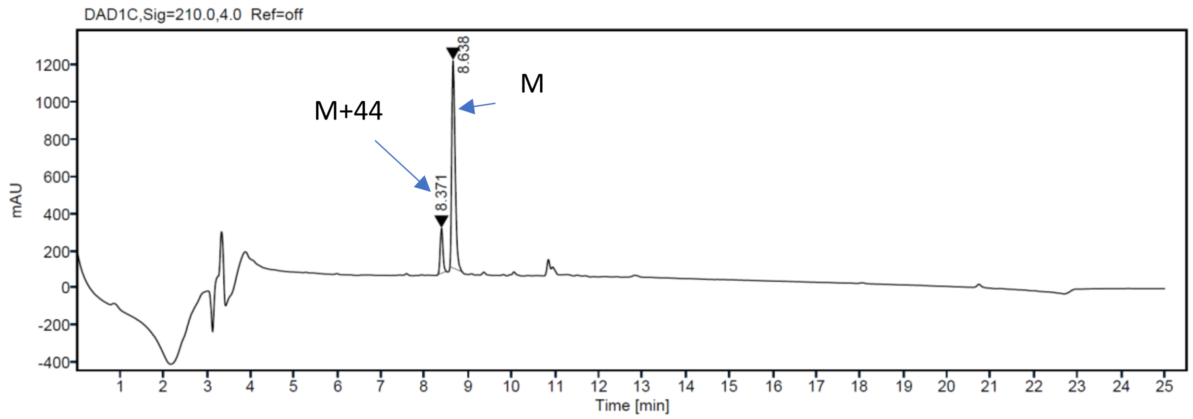

Retention time: 8.403 min **Area Percent:** 15%

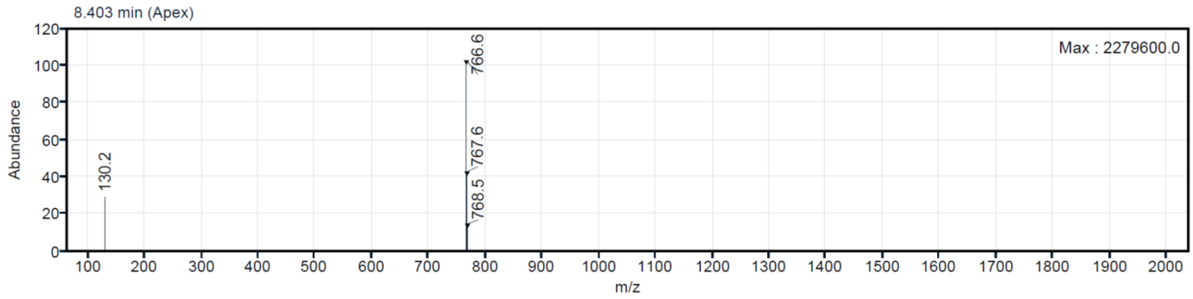

Retention time: 8.675 min **Area Percent:** 85%

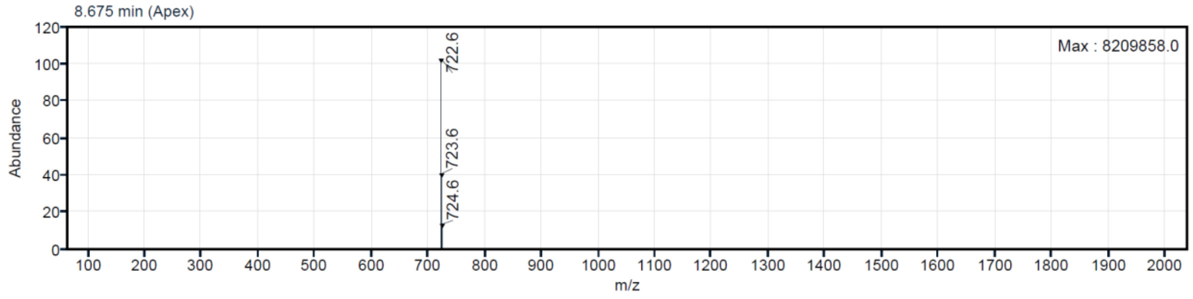

HPLC-UV chromatogram (210 nm) of **1d**:

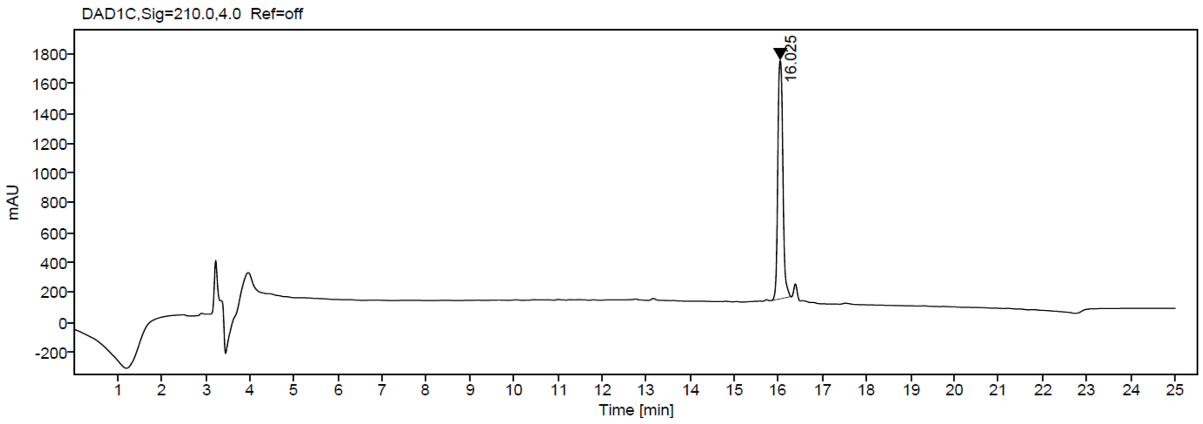

Retention time: 16.055 min **Area Percent:** 100%

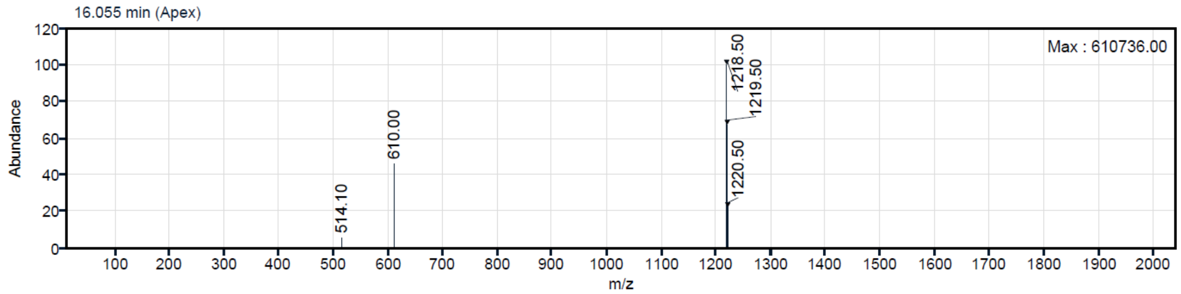

HRMS (nanochip-ESI/LTQ-Orbitrap) m/z: $[M + H]^+$ Calcd for $C_{54}H_{55}F_6IN_7O_{11}^+$ 1218.2903; Found 1218.2927.

MS/MS fragmentation of **1d**:

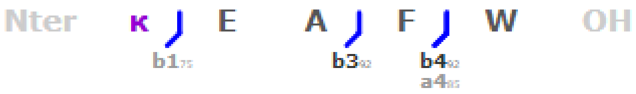

κ = Lys(C18H7F6IO2)
Nter = C2H3O

Sequence	Type	MF	MF Mass	m/z	Intensity	Similarity
KEA	b3	C34H34F6IN4O8(+1)	867.1326	867.132	21.96	92.00%
KEAF	b4	C43H43F6IN5O9(+1)	1014.201	1014.2	69.29	91.60%
KEAF	a4	C42H43F6IN5O8(+1)	986.2061	986.2055	7.49	85.36%
K	b1	C26H22F6IN2O4(+1)	667.0528	667.0523	9.83	74.51%

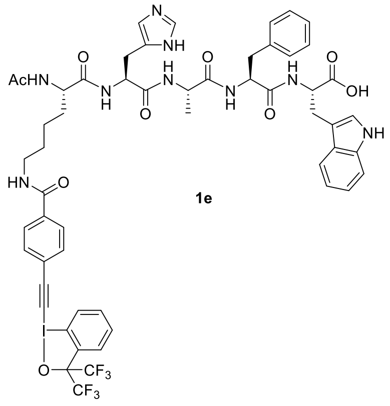

1e

Following the general procedure, the reaction was conducted in 0.03 mmol scale. The desired product **1e** (23.6 mg, 0.0193 mmol, 64% yield) was isolated by **Method 2**.

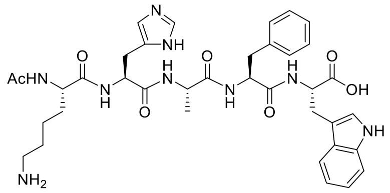

HPLC-UV chromatogram (210 nm) of **AcKHAFW-OH**:

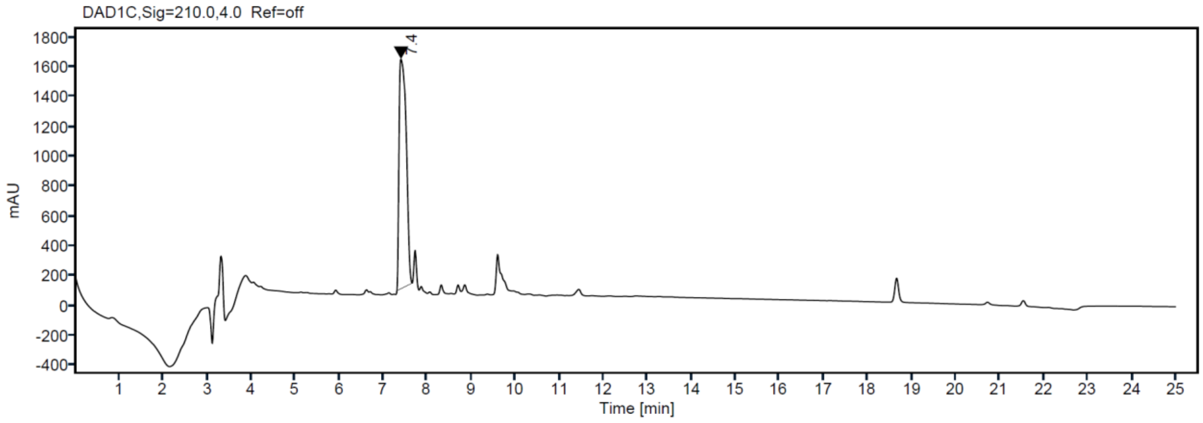

Retention time: 7.429 min **Area Percent:** 100%

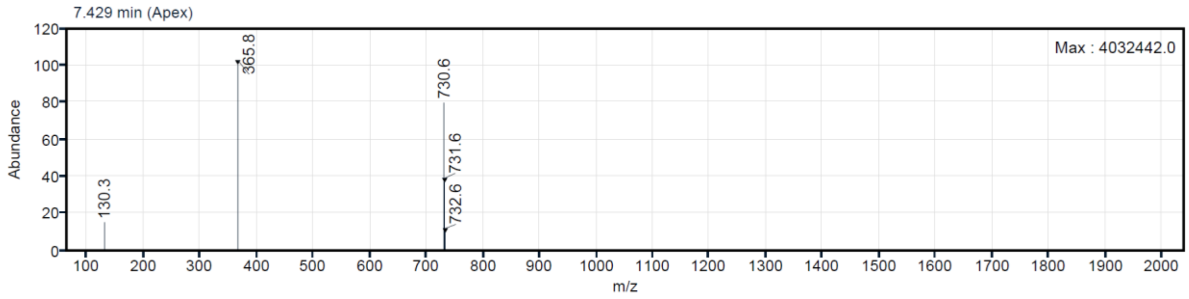

HPLC-UV chromatogram (210 nm) of **1e**:

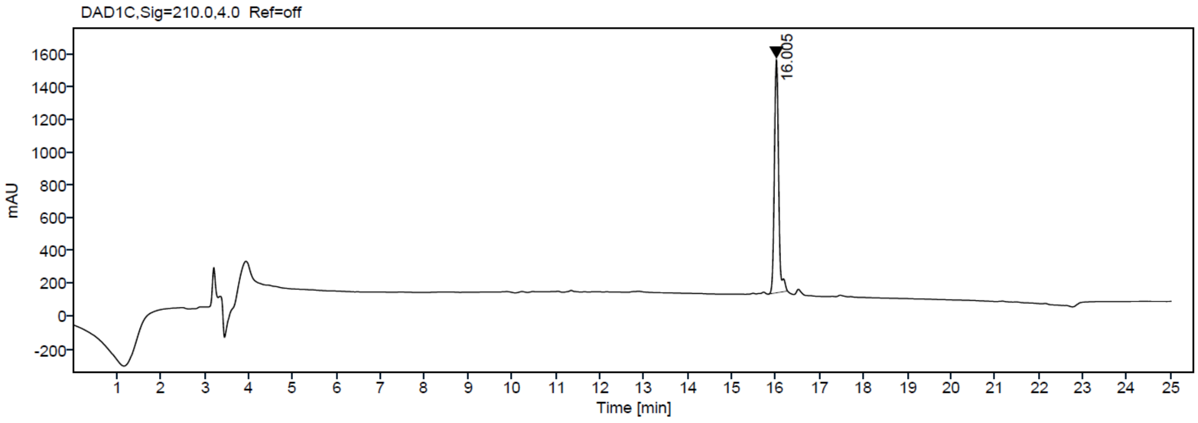

Retention time: 14.021 min **Area Percent:** 100%

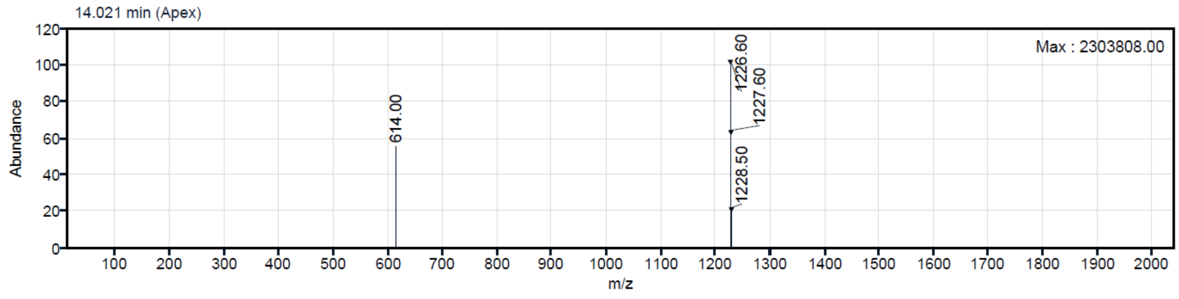

MS/MS fragmentation of **1e**:

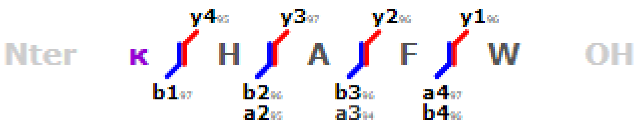

κ = Lys(C18H7F6IO2)
Nter = C2H3O

Sequence	Type	MF	MF Mass	m/z	Intensity	Similarity
KHAF	a4	C43H43F6IN7O6(+1)	994.2224	497.6146	6.34	98.34%
KH	b2	C32H29F6IN5O5(+1)	804.1118	402.5592	12.29	97.21%
KHAF	b4	C44H43F6IN7O7(+1)	1022.217	1022.217	26.6	97.16%
AFW	y3	C23H27N4O4(+1)	423.2032	423.2027	1.27	96.74%
K	b1	C26H22F6IN2O4(+1)	667.0528	667.0523	10.86	96.51%
FW	y2	C20H22N3O3(+1)	352.1661	352.1656	26.54	96.38%
KHAF	a4	C43H43F6IN7O6(+1)	994.2224	994.2218	2.37	96.32%
KHA	b3	C35H34F6IN6O6(+1)	875.1489	438.0778	36.76	96.04%
W	y1	C11H13N2O2(+1)	205.0977	205.0972	7.84	95.77%
KHA	b3	C35H34F6IN6O6(+1)	875.1489	875.1483	40.21	95.77%
KH	b2	C32H29F6IN5O5(+1)	804.1118	804.1112	15.53	95.39%
HAFW	y4	C29H34N7O5(+1)	560.2621	560.2616	9.49	95.25%
KH	a2	C31H29F6IN5O4(+1)	776.1168	776.1163	0.6	95.06%
KHAF	b4	C44H43F6IN7O7(+1)	1022.217	511.612	66.03	94.48%
KHA	a3	C34H34F6IN6O5(+1)	847.154	847.1534	2.13	94.17%
KHA	a3	C34H34F6IN6O5(+1)	847.154	424.0803	1.42	94.16%

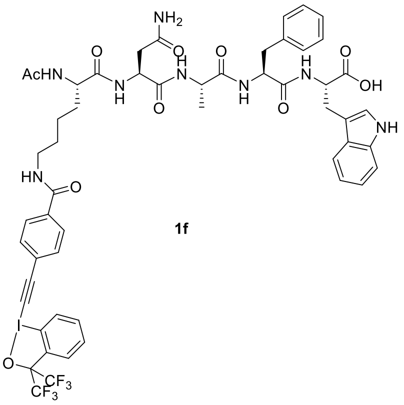

1f

Following the general procedure, the reaction was conducted in 0.03 mmol scale. The desired product **1f** (22.4 mg, 0.0192 mmol, 62% yield) was isolated by **Method 2**.

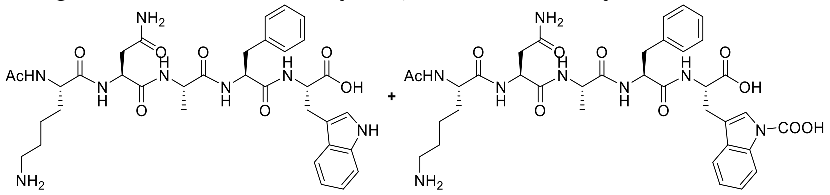

HPLC-UV chromatogram (210 nm) of **AcKNAFW-OH**:

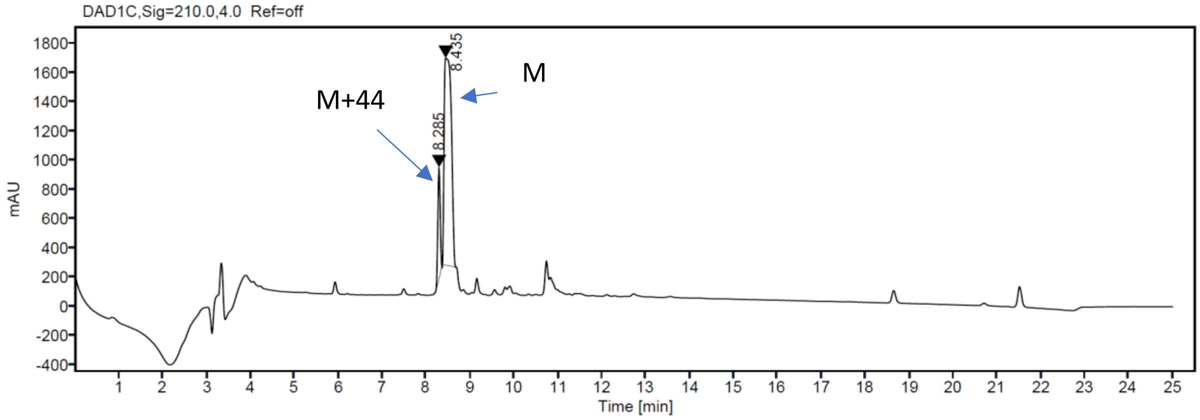

HPLC-UV chromatogram (210 nm) of **1f**:

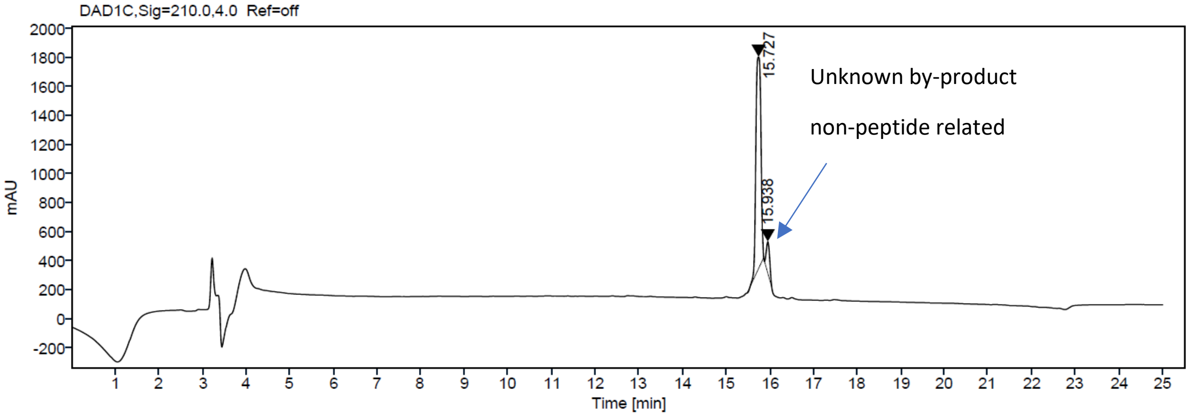

Retention time: 15.765 min **Area Percent:** 100%

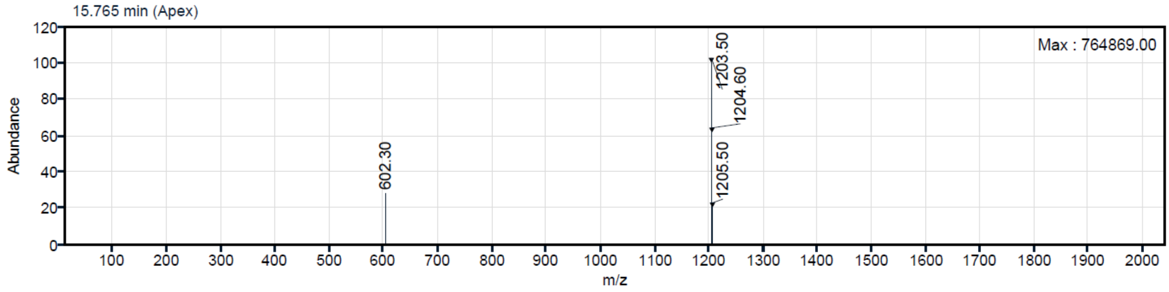

HRMS (nanochip-ESI/LTQ-Orbitrap) m/z: $[M + H]^+$ Calcd for $C_{53}H_{54}F_6IN_8O_{10}^+$ 1203.2906; Found 1203.2938.

MS/MS fragmentation of **1f**:

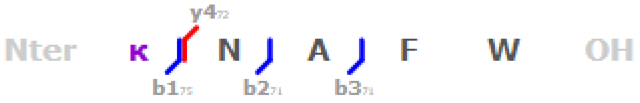

κ = Lys(C18H7F6IO2)
Nter = C2H3O

Sequence	Type	MF	MF Mass	m/z	Intensity	Similarity
K	b1	C26H22F6IN2O4(+1)	667.0528	667.0523	2.05	74.51%
NAFW	y4	C27H33N6O6(+1)	537.2462	537.2456	0.93	72.36%

KNA	b3	C33H33F6IN5O7(+1)	852.1329	852.1323	30.47	70.90%
KN	b2	C30H28F6IN4O6(+1)	781.0958	781.0952	2.81	70.70%

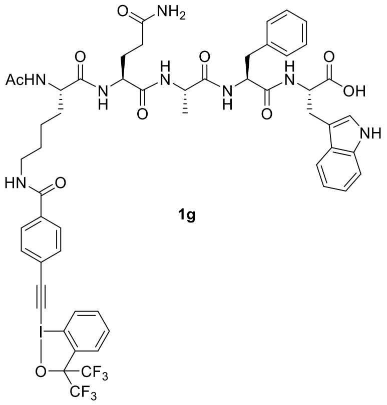

1g

Following the general procedure, the reaction was conducted in 0.03 mmol scale. The desired product **1g** (25.5 mg, 0.0210 mmol, 70% yield) was isolated by **Method 2**.

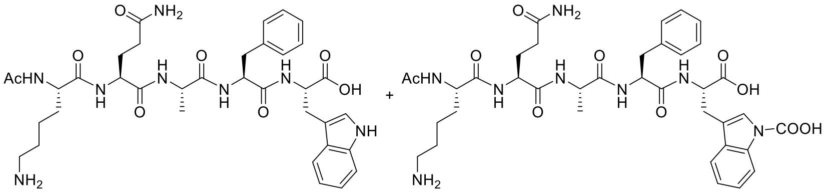

HPLC-UV chromatogram (210 nm) of **AcKQAFW-OH**:

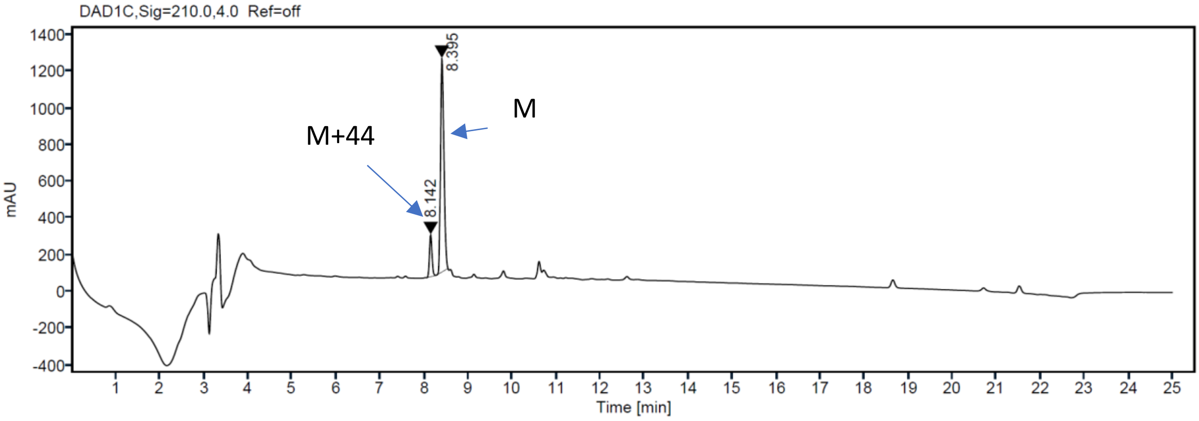

Retention time: 8.173 min **Area Percent:** 15%

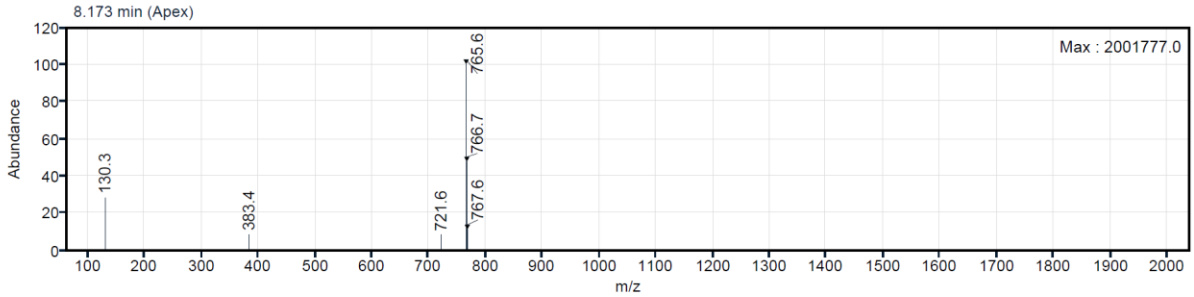

Retention time: 8.431 min **Area Percent:** 85%

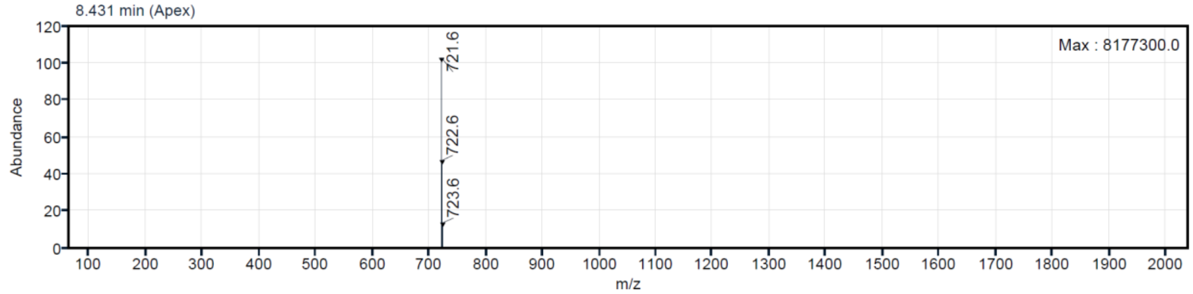

HPLC-UV chromatogram (210 nm) of **1g**:

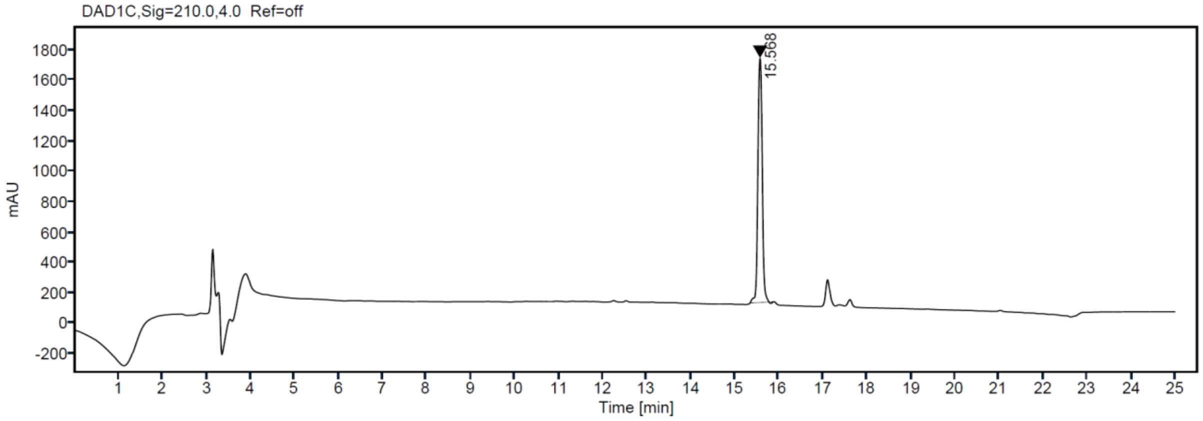

Retention time: 15.608 min **Area Percent:** 100%

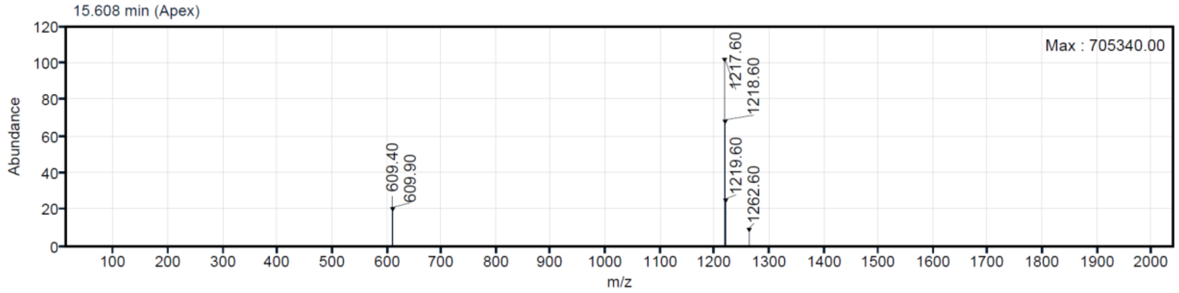

HRMS (Nanochip-based ESI/LTQ-Orbitrap) m/z: $[M + H]^+$ Calcd for $C_{54}H_{56}F_6IN_8O_{10}^+$ 1217.3063; Found 1217.3060.

MS/MS fragmentation of **1g**:

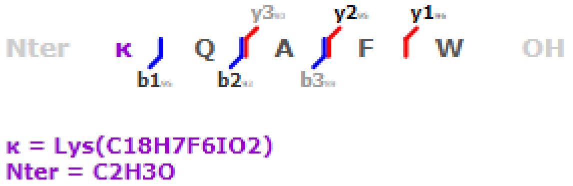

Sequence	Type	MF	MF Mass	m/z	Intensity	Similarity
W	y1	C11H13N2O2(+1)	205.0977	205.0972	24.12	95.78%
FW	y2	C20H22N3O3(+1)	352.1661	352.1656	17.38	95.21%
K	b1	C26H22F6IN2O4(+1)	667.0528	667.0523	65.75	94.66%
KQ	b2	C31H30F6IN4O6(+1)	795.1114	795.1109	13.63	93.46%
KQA	b3	C34H35F6IN5O7(+1)	866.1485	866.148	5.78	89.07%

AFW	y3	C23H27N4O4(+1)	423.2032	212.105	50.35	79.55%

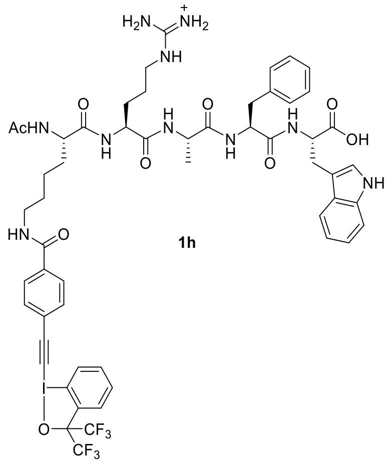

1h

Following the general procedure, the reaction was conducted in 0.03 mmol scale. The desired product **1h** (21.4 mg, 0.0172 mmol, 57% yield) was isolated by **Method 2**.

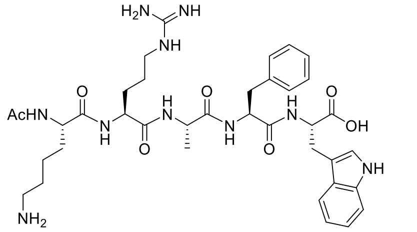

HPLC-UV chromatogram (210 nm) of **AcKRAFW-OH**:

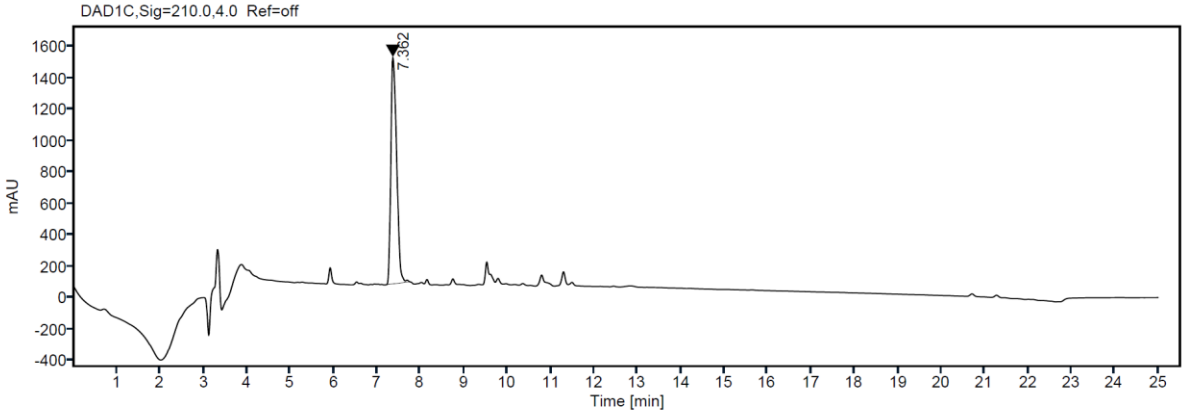

Retention time: 7.386 min **Area Percent:** 100%

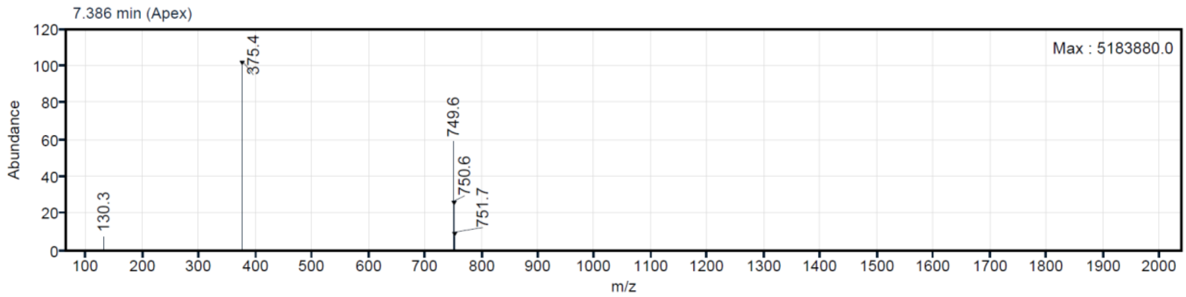

HPLC-UV chromatogram (210 nm) of **1h**:

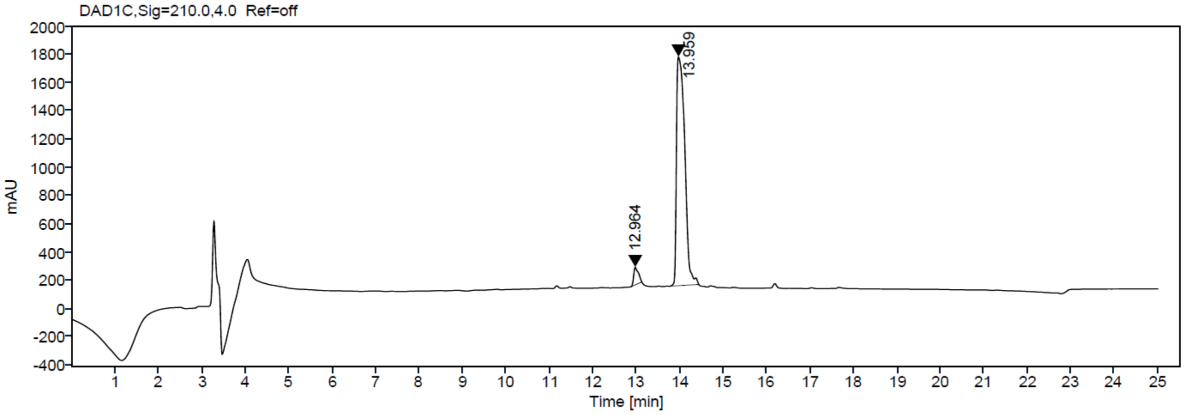

Retention time: 13.995 min **Area Percent:** 100%

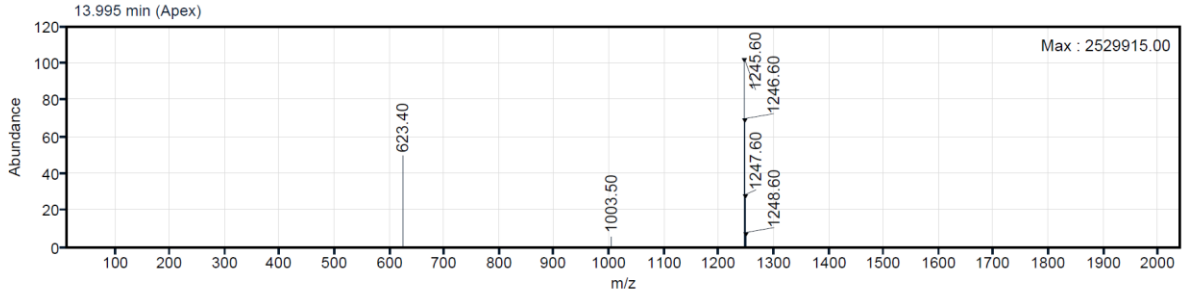

HRMS (nanochip-ESI/LTQ-Orbitrap) m/z: $[M + H_2]^{+2}$ Calcd for $C_{55}H_{61}F_6IN_{10}O_9^{+2}$ 623.1780; Found 623.1770.

MS/MS fragmentation of **1h**:

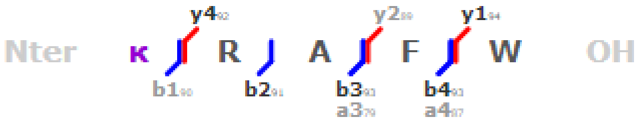

κ = Lys(C18H7F6IO2)
Nter = C2H3O

Sequence	Type	MF	MF Mass	m/z	Intensity	Similarity
KRAF	b4	C44H48F6IN8O7(+1)	1041.26	1041.259	6.74	94.76%
W	y1	C11H13N2O2(+1)	205.0977	205.0972	1.45	94.02%
KRA	b3	C35H39F6IN7O6(+1)	894.1911	894.1905	2.29	93.00%
RAFW	y4	C29H39N8O5(+1)	579.3043	579.3038	2.73	91.82%
KRAF	b4	C44H48F6IN8O7(+1)	1041.26	521.1331	2.41	91.48%
KR	b2	C32H34F6IN6O5(+1)	823.154	823.1534	2.5	90.89%
K	b1	C26H22F6IN2O4(+1)	667.0528	667.0523	0.87	89.79%
FW	y2	C20H22N3O3(+1)	352.1661	352.1656	1.49	89.09%
KRAF	a4	C43H48F6IN8O6(+1)	1013.265	1013.264	2.22	86.82%
KRA	a3	C34H39F6IN7O5(+1)	866.1962	866.1956	0.64	79.35%

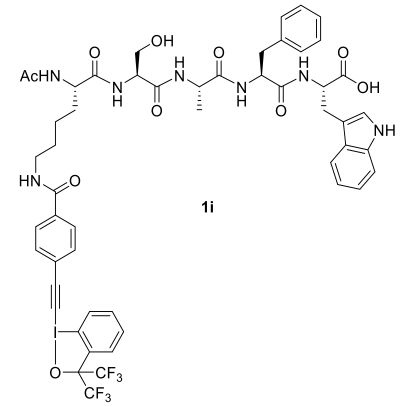

1i

Following the general procedure, the reaction was conducted in 0.03 mmol scale. The desired product **1i** (25.2 mg, 0.0214 mmol, 71% yield) was isolated by **Method 2**.

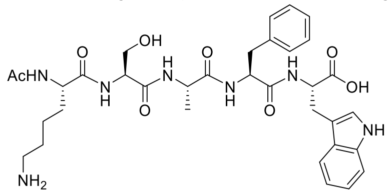

HPLC-UV chromatogram (210 nm) of **AcKSAFW-OH**:

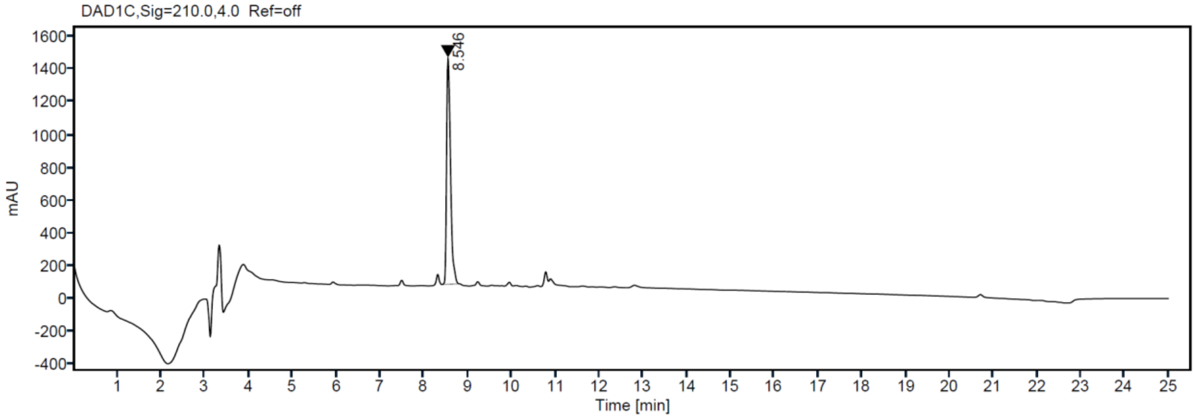

Retention time: 8.576 min **Area Percent:** 100%

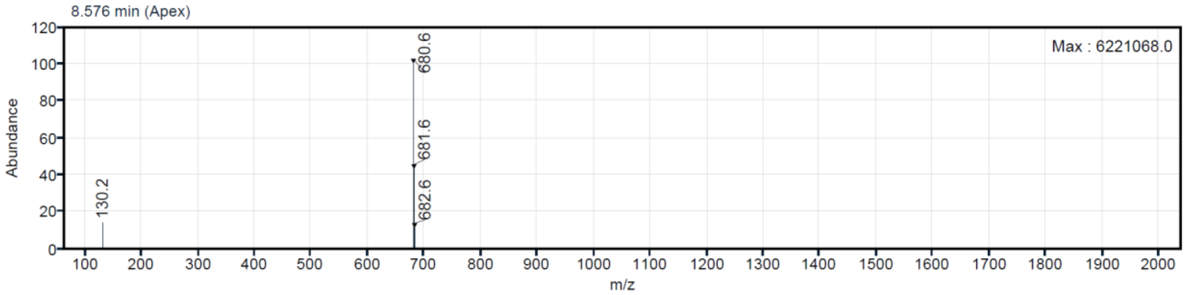

HPLC-UV chromatogram (210 nm) of **1i**:

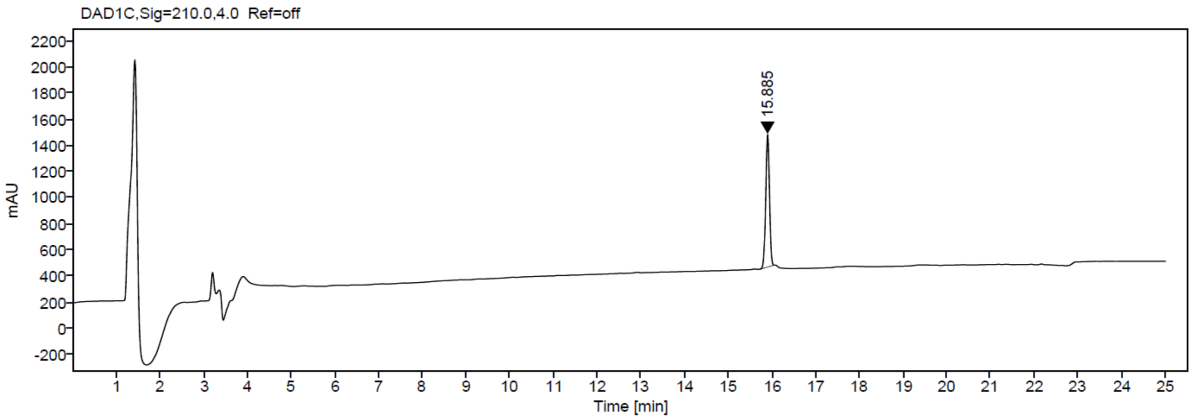

Retention time: 15.971 min **Area Percent:** 100%

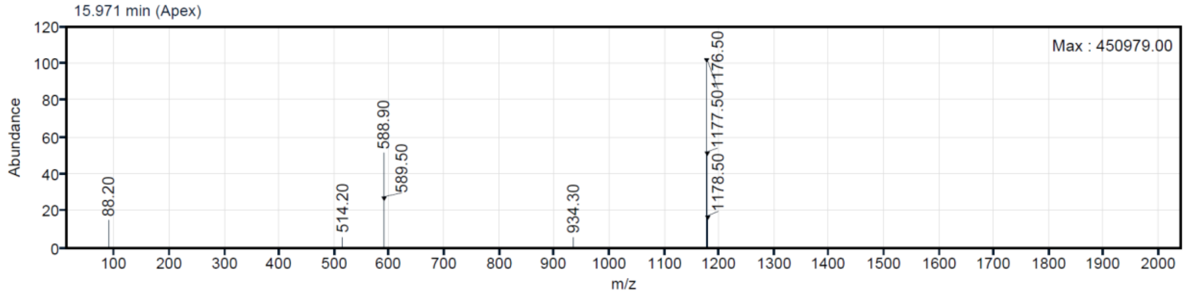

HRMS (nanochip-ESI/LTQ-Orbitrap) m/z: $[M + H]^+$ Calcd for $C_{52}H_{53}F_6IN_7O_{10}^+$ 1176.2797; Found 1176.2817.

MS/MS fragmentation of **1i**:

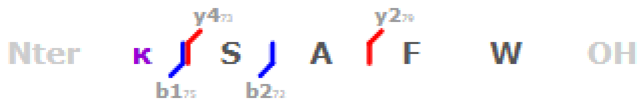

κ = Lys(C18H7F6IO2)
Nter = C2H3O

vv	Type	MF	MF Mass	m/z	Intensity	Similarity
FW	y2	C20H22N3O3(+1)	352.1661	352.1656	1.01	79.20%
K	b1	C26H22F6IN2O4(+1)	667.0528	667.0523	8.76	74.51%
SAFW	y4	C26H32N5O6(+1)	510.2353	510.2347	3.48	73.37%
KS	b2	C29H27F6IN3O6(+1)	754.0849	754.0843	1.56	71.68%

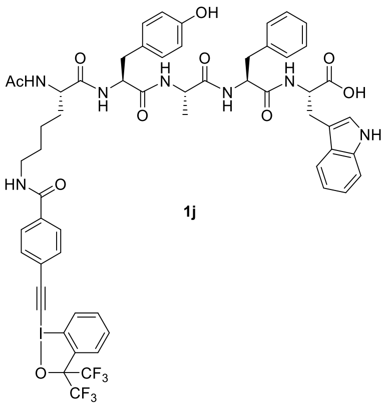

1j

Following the general procedure, the reaction was conducted in 0.03 mmol scale. The desired product **1j** (21.7 mg, 0.0173 mmol, 58% yield) was isolated by **Method 2**.

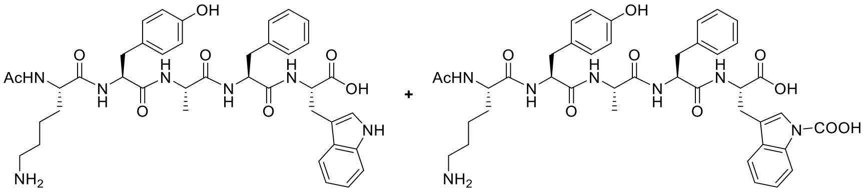

HPLC-UV chromatogram (210 nm) of **AcKYAFW-OH**:

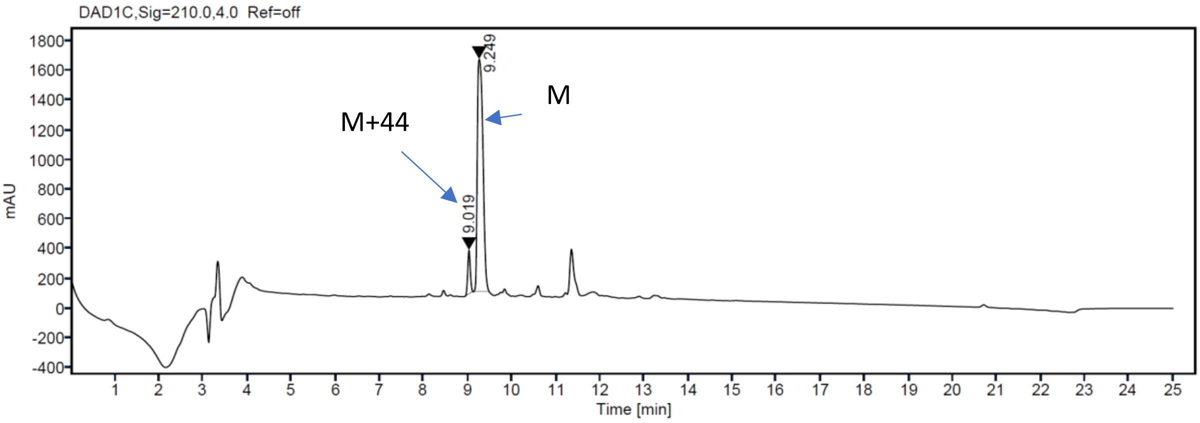

Retention time: 9.048 min **Area Percent:** 11%

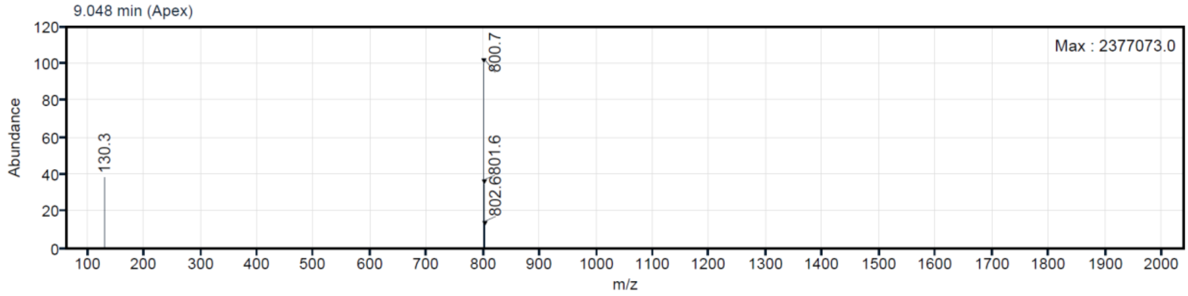

Retention time: 9.293 min **Area Percent:** 89%

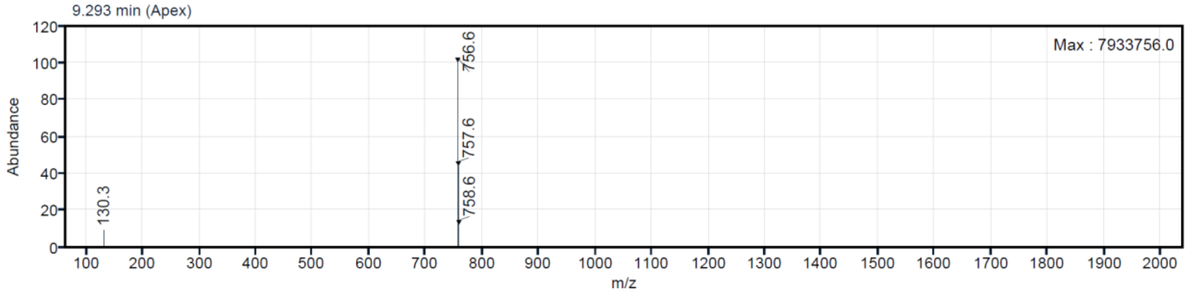

HPLC-UV chromatogram (210 nm) of **1j**:

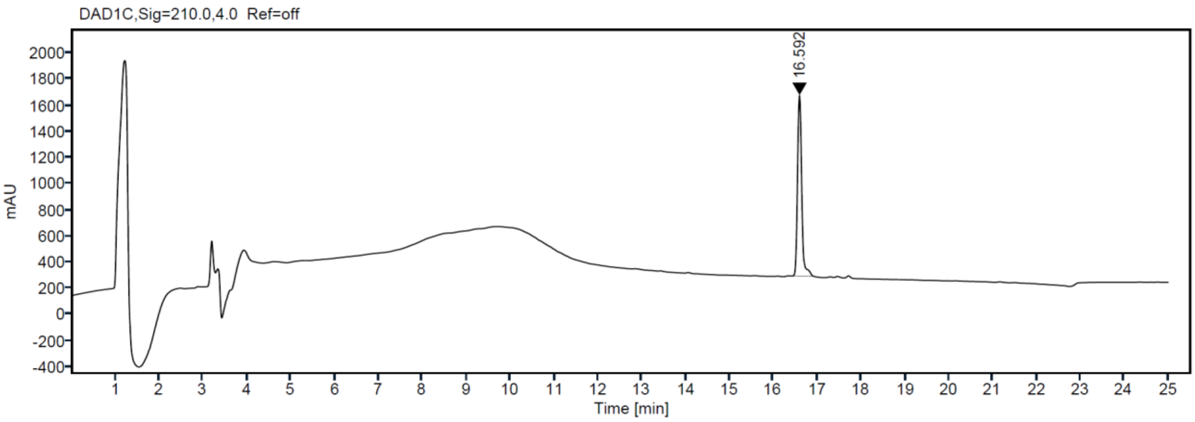

Retention time: 16.644 min **Area Percent:** 100%

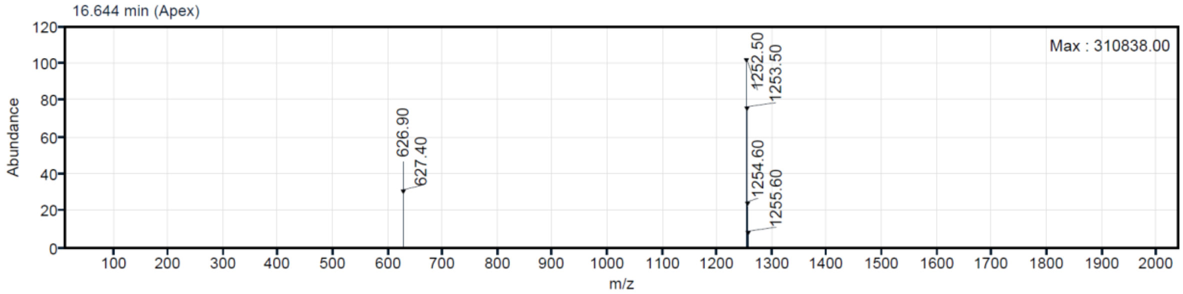

HRMS (nanochip-ESI/LTQ-Orbitrap) m/z: $[M + H]^+$ Calcd for $C_{58}H_{57}F_6IN_7O_{10}^+$ 1252.3110; Found 1252.3127.

MS/MS fragmentation of **1j**:

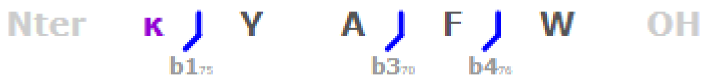

κ = Lys(C18H7IO2F6)
Nter = C2H3O

Sequence	Type	MF	MF Mass	m/z	Intensity	Similarity
KYAF	b4	C47H45F6IN5O8(+1)	1048.222	1048.221	48.57	75.80%
K	b1	C26H22F6IN2O4(+1)	667.0528	667.0523	4.56	74.51%
KYA	b3	C38H36F6IN4O7(+1)	901.1533	901.1527	27.72	70.17%

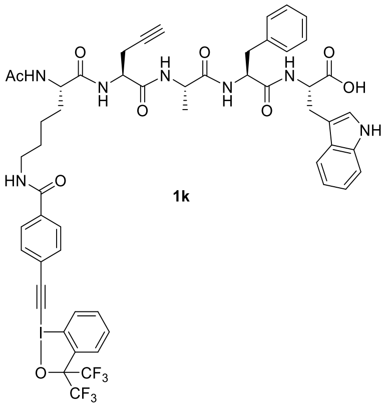

1k

Following the general procedure, the reaction was conducted in 0.03 mmol scale. The desired product **1k** (27.3 mg, 0.0231 mmol, 77% yield) was isolated by **Method 2**.

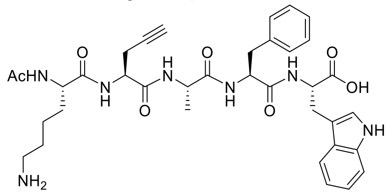

HPLC-UV chromatogram (210 nm) of **AcKPraAFW-OH**:

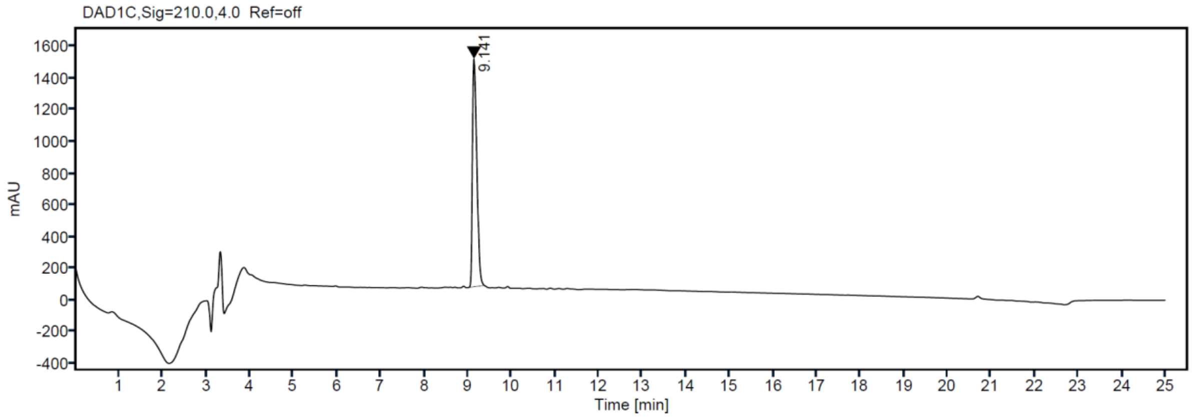

Retention time: 9.172 min **Area Percent:** 100%

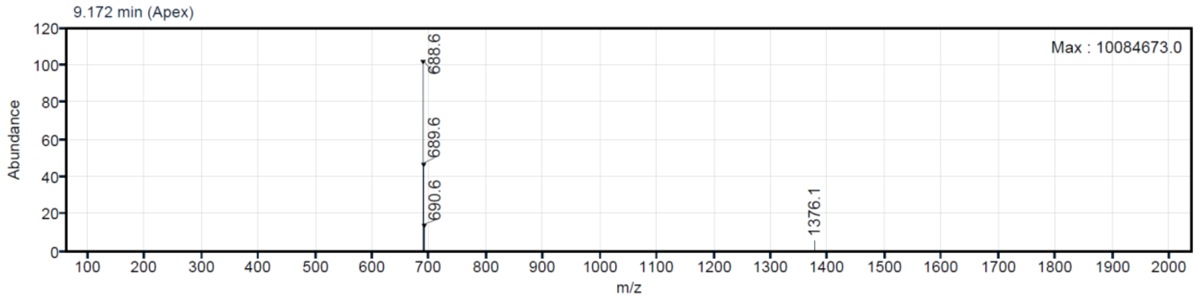

HPLC-UV chromatogram (210 nm) of **1k**:

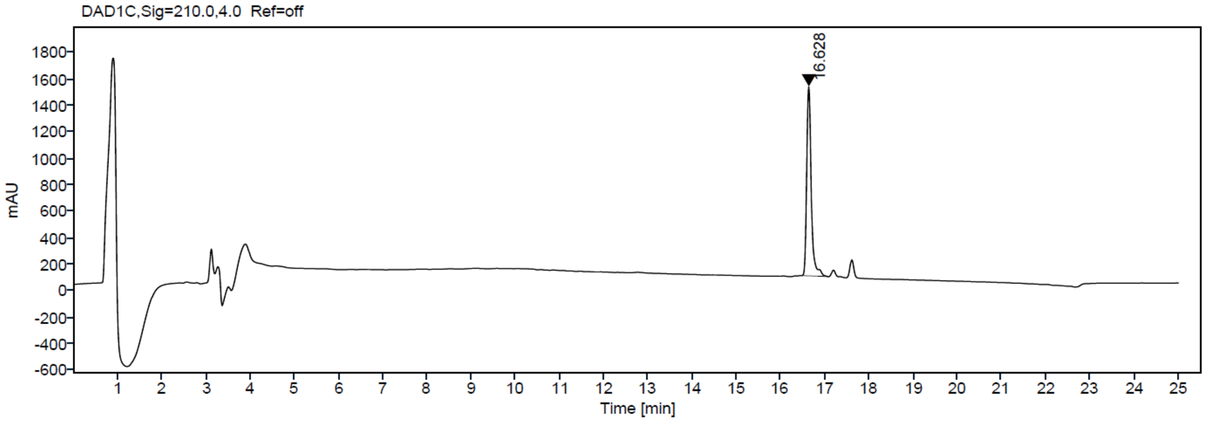

Retention time: 16.651 min **Area Percent:** 100%

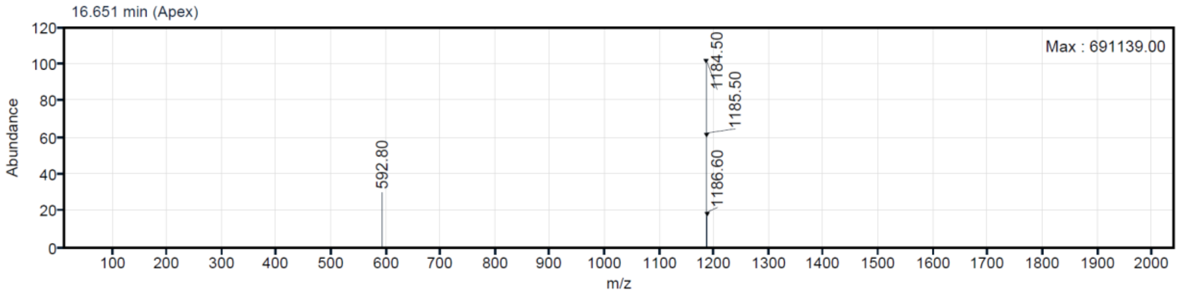

HRMS (nanochip-ESI/LTQ-Orbitrap) m/z: $[M + H]^+$ Calcd for $C_{54}H_{53}F_6IN_7O_9^+$ 1184.2848; Found 1184.2840.

MS/MS fragmentation of **1k**:

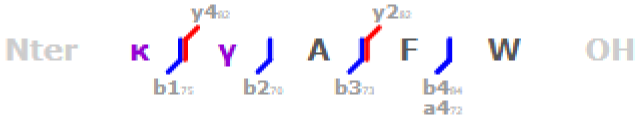

κ = Lys(C18H7F6IO2)
γ = Gly(C3H2)
Nter = C2H3O

Sequence	Type	MF	MF Mass	m/z	Intensity	Similarity
KGAF	b4	C43H41F6IN5O7(+1)	980.1955	980.1949	61.04	83.68%
GAFW	y4	C28H32N5O5(+1)	518.2403	518.2398	2.47	81.92%
FW	y2	C20H22N3O3(+1)	352.1661	352.1656	0.81	81.71%
K	b1	C26H22F6IN2O4(+1)	667.0528	667.0523	4.21	74.51%
KGA	b3	C34H32F6IN4O6(+1)	833.1271	833.1265	20.23	73.33%
KGAF	a4	C42H41F6IN5O6(+1)	952.2006	952.2	7.5	71.91%
KG	b2	C31H27F6IN3O5(+1)	762.09	762.0894	4.17	70.35%

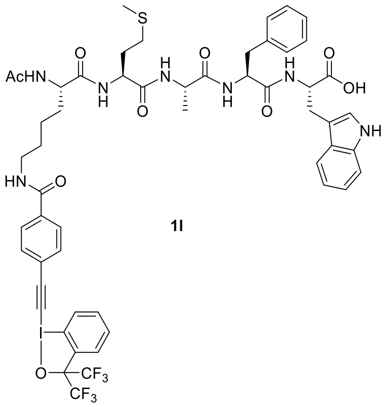

1l

Following the general procedure, the reaction was conducted in 0.036 mmol scale. The desired product **1l** (17.4 mg, 0.0143 mmol, 43% yield) was isolated by **Method 2**.

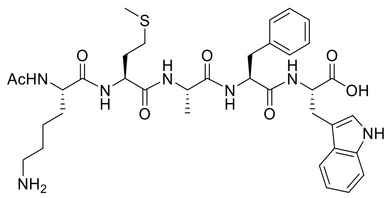

HPLC-UV chromatogram (210 nm) of **AcKMAFW-OH**:

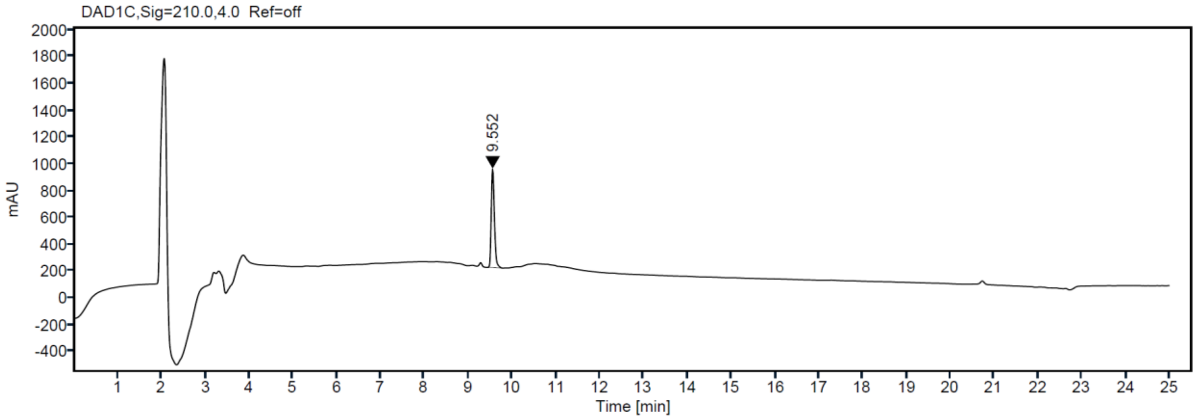

Retention time: 9.577 min **Area Percent:** 100%

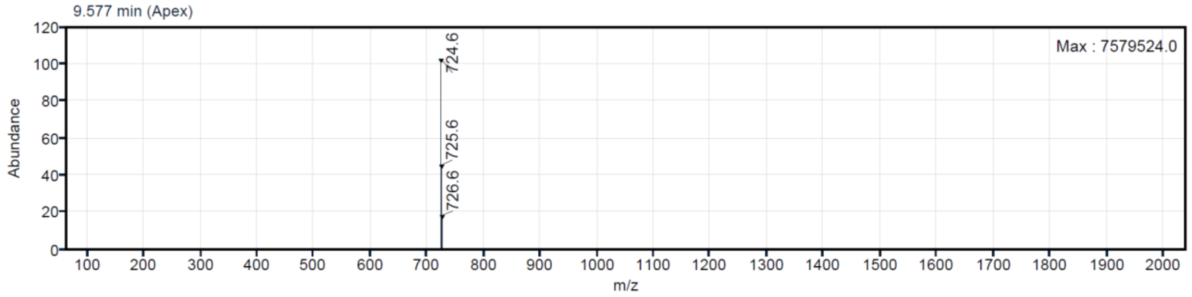

HPLC-UV chromatogram (210 nm) of **1l**:

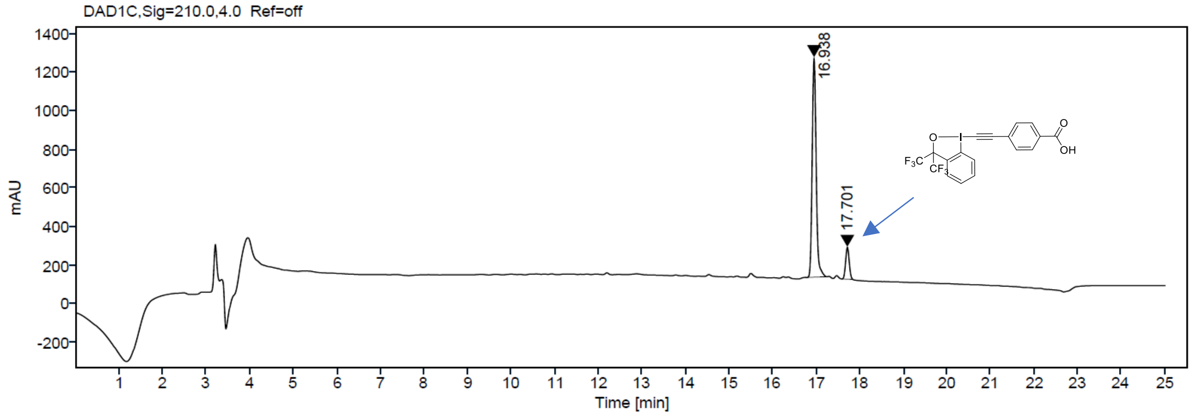

HRMS (nanochip-ESI/LTQ-Orbitrap) m/z: $[M + H]^+$ Calcd for $C_{54}H_{57}F_6IN_7O_9S^+$ 1220.2882; Found 1220.2892.

MS/MS fragmentation of **1l**:

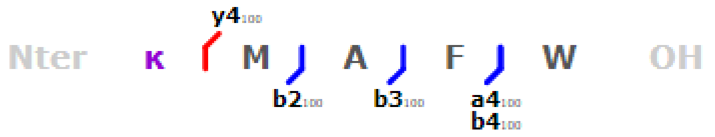

κ = Lys(C18H7IO2F6)
Nter = C2H3O

Sequence	Type	MF	MF Mass	m/z	Intensity	Similarity
MAFW	y4	C28H36N5O5S(+1)	554.2437	554.2432	2.32	100.00%
KMAF	a4	C42H45F6IN5O6S(+1)	988.2039	988.2034	0.8	100.00%
KM	b2	C31H31F6IN3O5S(+1)	798.0933	798.0928	0.58	100.00%
KMA	b3	C34H36F6IN4O6S(+1)	869.1304	869.1299	8.98	100.00%
KMAF	b4	C43H45F6IN5O7S(+1)	1016.199	1016.198	34.3	99.96%
KMAFW		C54H56F6IN7O9S	1219.281	1220.288	97.83	97.27%

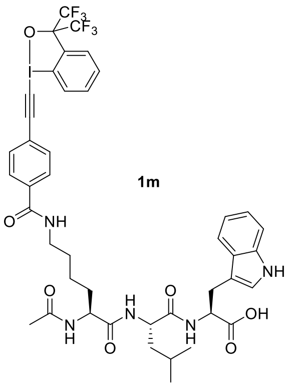

1m

Following the general procedure, the reaction was conducted in 0.034 mmol scale. The desired product **1m** (12.4 mg, 0.0142 mmol, 42% yield) was isolated by **Method 2**.

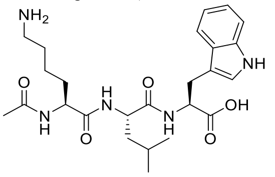

HPLC-UV chromatogram (210 nm) of **AcKLW-OH**:

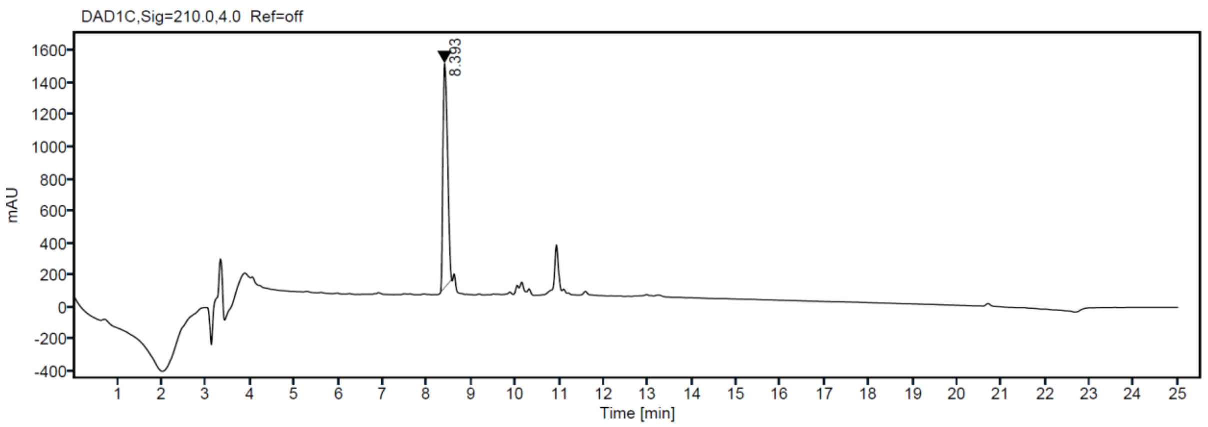

Retention time: 8.427 min **Area Percent:** 100%

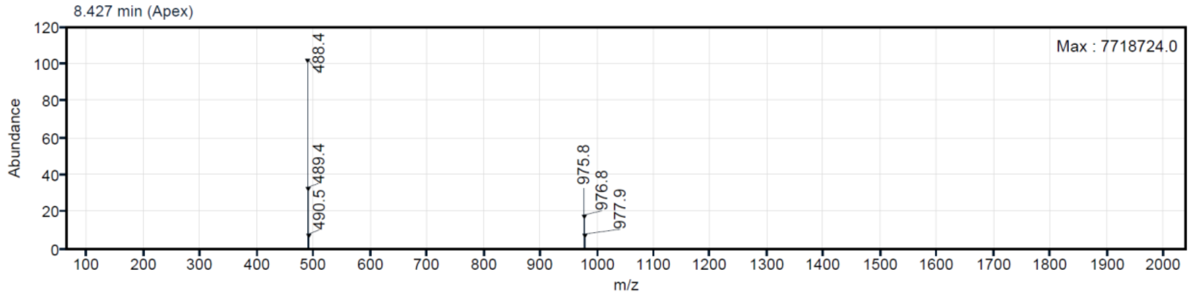

HPLC-UV chromatogram (210 nm) of **1m**:

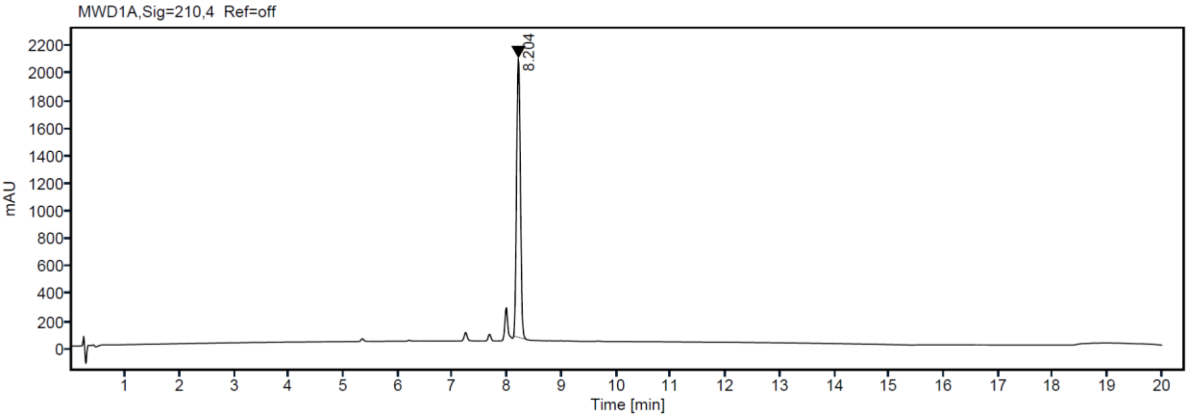

Retention time: 8.295 min **Area Percent:** 100%

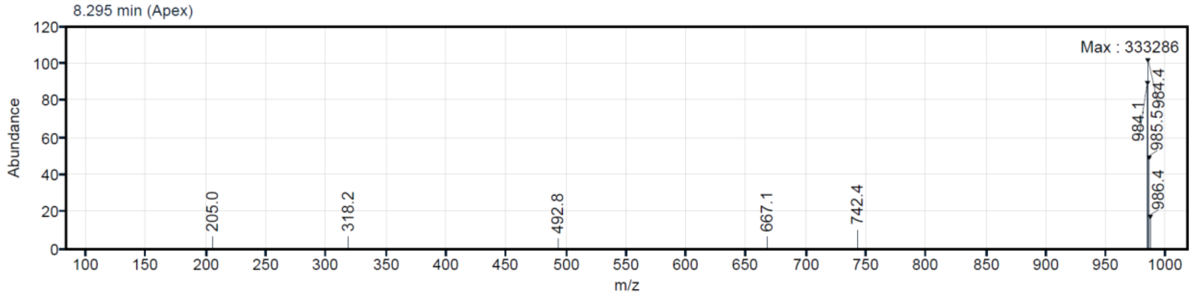

HRMS (ESI/QTOF) m/z: $[M + H]^+$ Calcd for $C_{43}H_{45}F_6IN_5O_7^+$ 984.2262; Found 984.2253.
MS/MS fragmentation of **1m**:

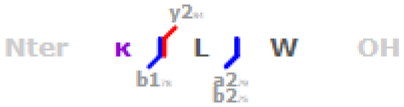

κ = Lys(C18H7F6IO2)
Nter = C2H3O

Sequence	Type	MF	MF Mass	m/z	Intensity	Similarity
LW	y2	C17H24N3O3(+1)	318.1818	318.1812	25.59	84.29%
KL	a2	C31H33F6IN3O4(+1)	752.142	752.1414	0.57	79.48%
K	b1	C26H22F6IN2O4(+1)	667.0528	667.0523	88.51	77.97%
KL	b2	C32H33F6IN3O5(+1)	780.1369	780.1364	102.93	75.13%

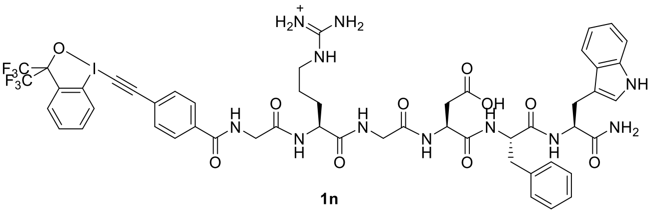

1n

Following the general procedure, the reaction was conducted in 0.029 mmol scale. The desired product **1n** (24.5mg, 0.0200 mmol, 68% yield) was isolated by **Method 2**.

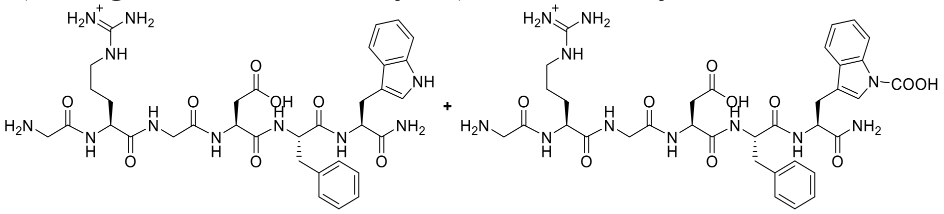

HPLC-UV chromatogram (210 nm) of **GRGDFW-NH₂**:

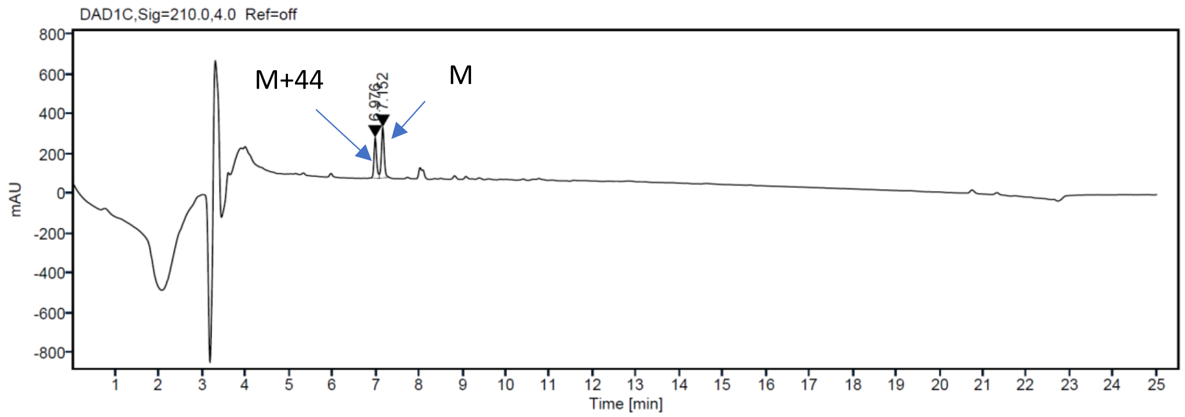

Retention time: 6.996 min **Area Percent:** 54%

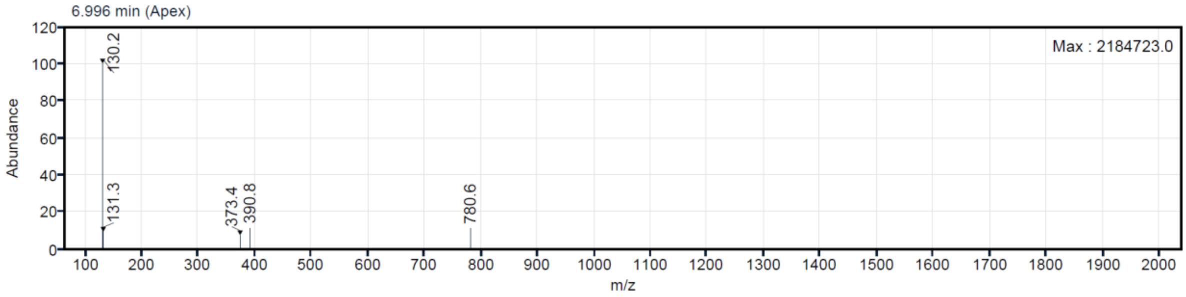

Retention time: 7.188 min **Area Percent:** 46%

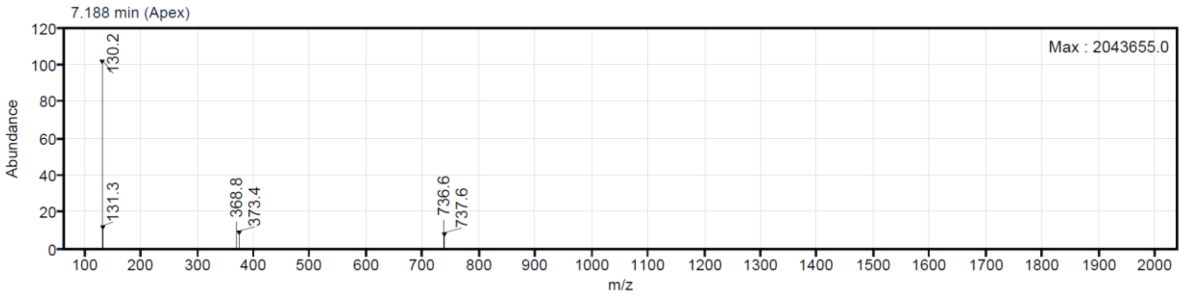

HPLC-UV chromatogram (210 nm) of **1n**:

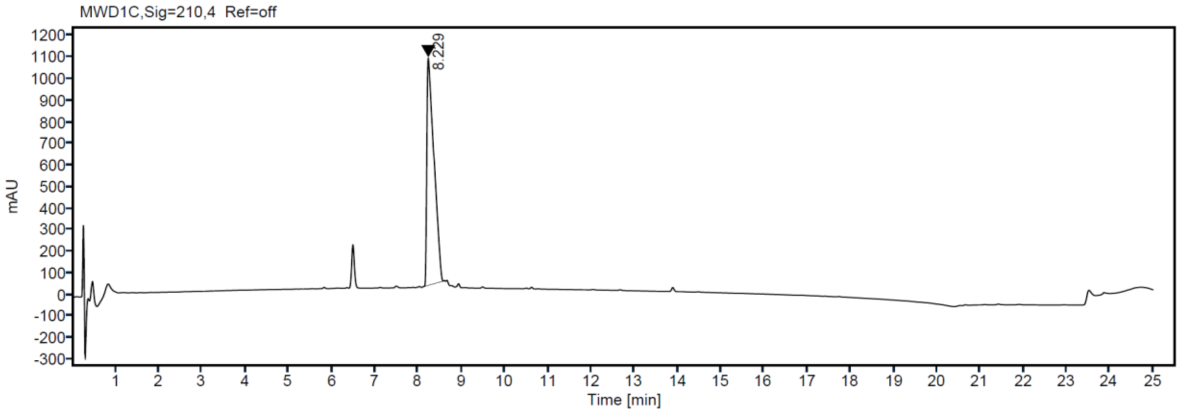

Retention time: 8.38 min **Area Percent:** 100%

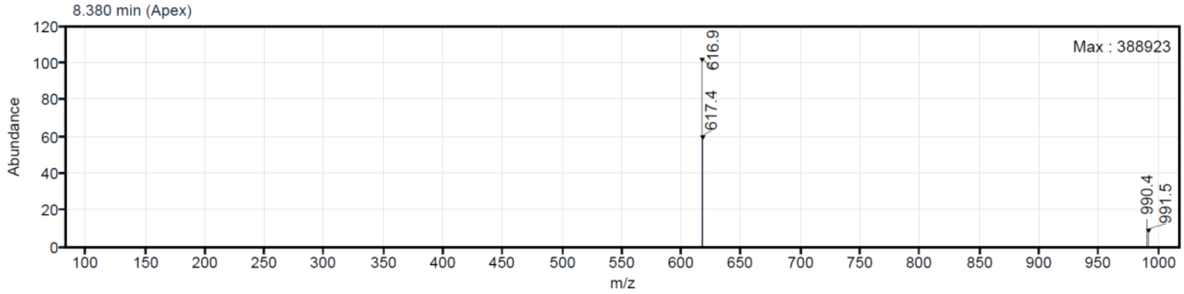

HRMS (nanochip-ESI/LTQ-Orbitrap) m/z: $[M + H_2]^{+2}$ Calcd for $C_{52}H_{54}F_6IN_{11}O_{10}^{+2}$ 616.6497; Found 616.6502.

MS/MS fragmentation of **1n**:

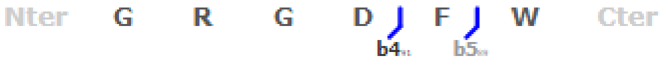

Nter = C18H8IO2F6
Cter = NH2

Sequence	Type	MF	MF Mass	m/z	Intensity	Similarity
GRGD	b4	C32H31F6IN7O8(+1)	882.1183	441.5625	0.75	91.92%
GRGD	b4	C32H31F6IN7O8(+1)	882.1183	882.1178	5.43	90.02%
GRGDF	b5	C41H40F6IN8O9(+1)	1029.1867	1029.186	3.77	89.34%

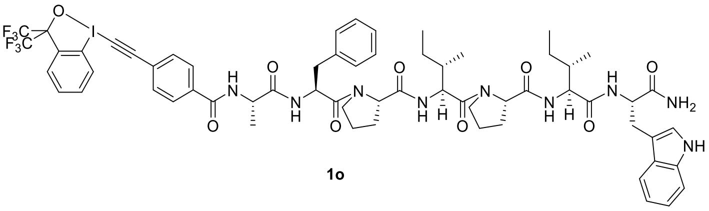

1o

Following the general procedure, the reaction was conducted in 0.020 mmol scale. The desired product **1o** (15.7 mg, 0.0173 mmol, 59% yield) was isolated by **Method 2**.

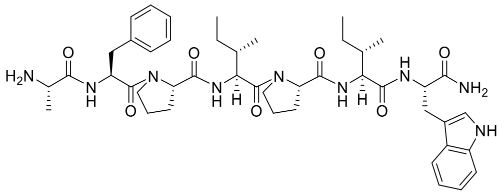

HPLC-UV chromatogram (210 nm) of **AFPIPIW-NH$_2$**:

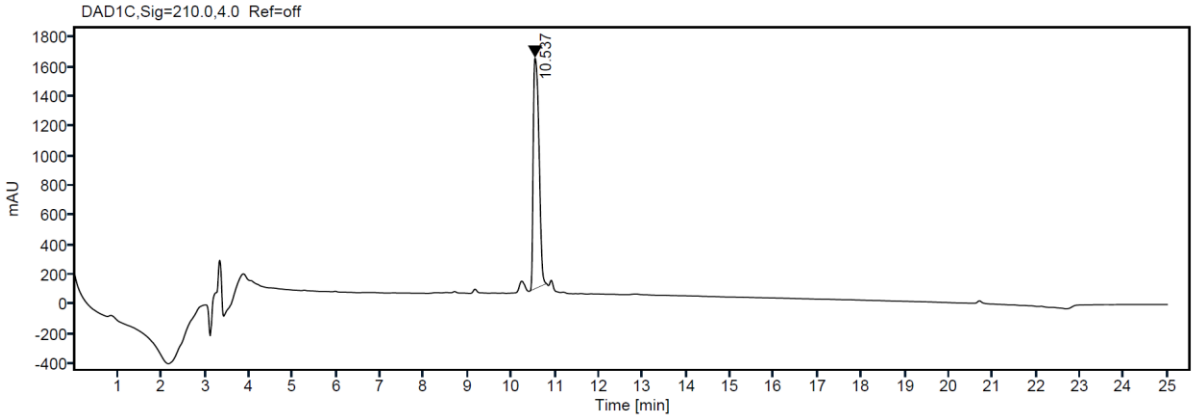

Retention time: 10.559 min **Area Percent:** 100%

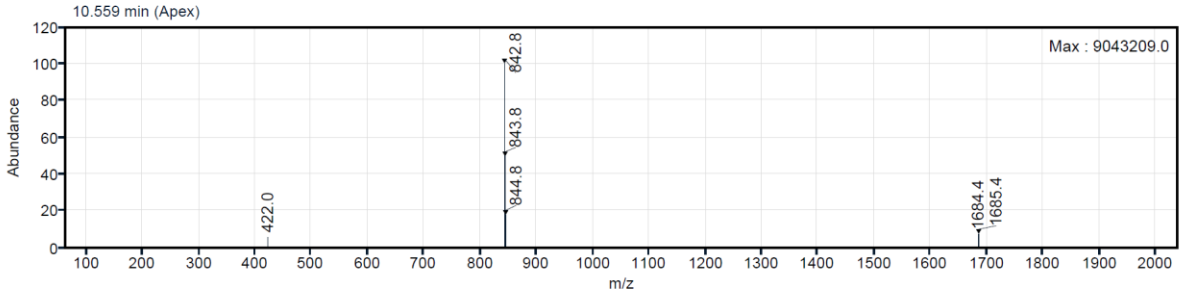

HPLC-UV chromatogram (210 nm) of **1o**:

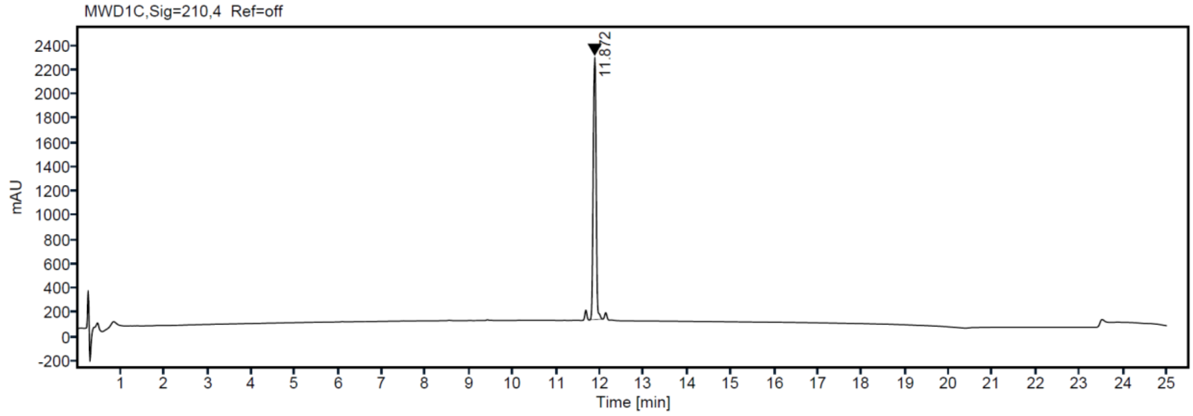

Retention time: 11.971 min **Area Percent:** 100%

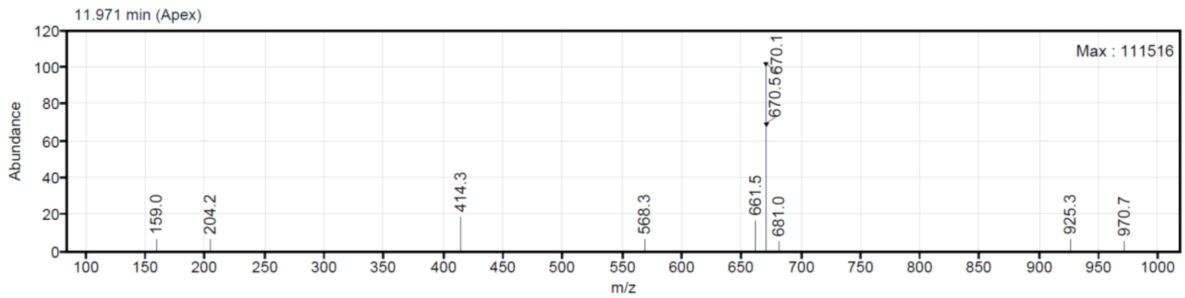

HRMS (ESI/QTOF) m/z: [M + H$_2$]$^{+2}$ Calcd for $C_{63}H_{72}F_6IN_9O_9^{+2}$ 669.7195; Found 669.7195.
MS/MS fragmentation of **1o**:

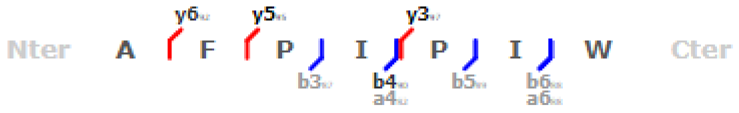

Nter = C18H8F6IO2
Cter = NH2

Sequence	Type	MF	MF Mass	m/z	Intensity	Similarity
PIW	y3	C22H32N5O3(+1)	414.2505	414.25	15.49	97.13%
PIPIW	y5	C33H50N7O5(+1)	624.3873	624.3868	28.73	95.06%
FPIPIW	y6	C42H59N8O6(+1)	771.4558	771.4552	23.85	92.02%
AFPI	b4	C41H40F6IN4O6(+1)	925.1897	925.1891	11.05	90.37%
AFPIP	b5	C46H47F6IN5O7(+1)	1022.2424	1022.242	1.7	89.49%
AFPIPI	b6	C52H58F6IN6O8(+1)	1135.3265	1135.326	100.07	88.34%
AFPIPI	a6	C51H58F6IN6O7(+1)	1107.3316	1107.331	3.67	87.76%
AFP	b3	C35H29F6IN3O5(+1)	812.1056	812.1051	1.46	86.68%
AFPI	a4	C40H40F6IN4O5(+1)	897.1948	897.1942	0.81	82.34%

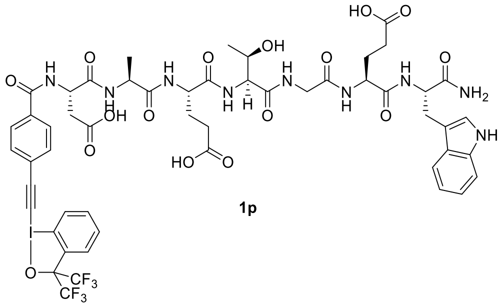

1p

Following the general procedure, the reaction was conducted in 0.024 mmol scale. The desired product **1p** (17.0 mg, 0.0125 mmol, 52% yield) was isolated by **Method 3**.

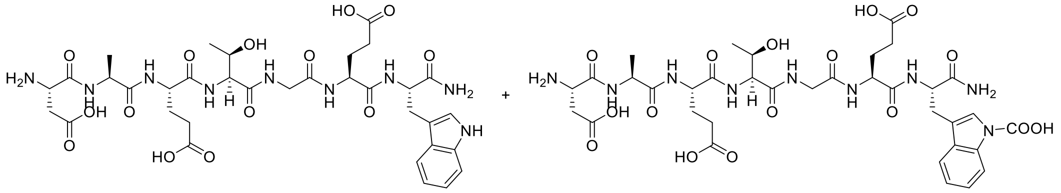

HPLC-UV chromatogram (210 nm) of **DAETGEW-NH$_2$**:

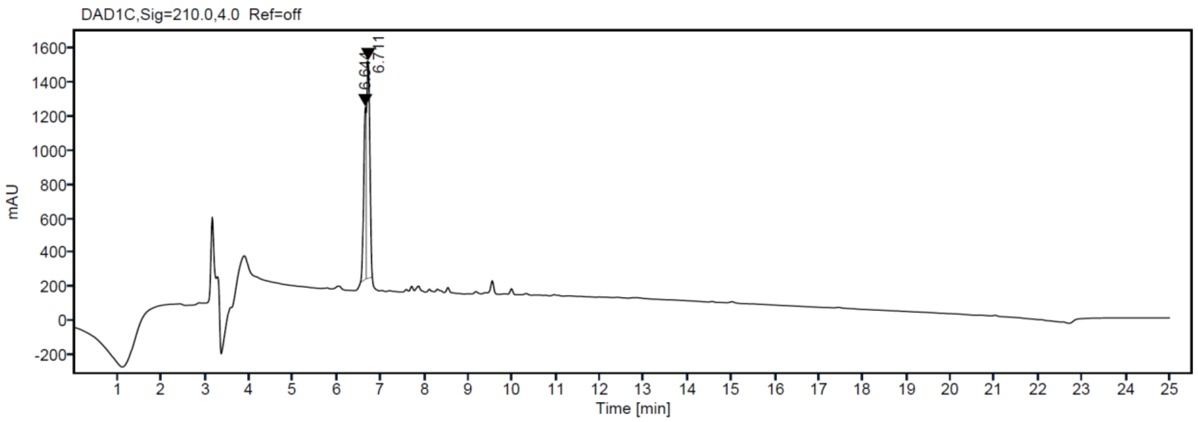

Retention time: 6.668 min **Area Percent:** 38%

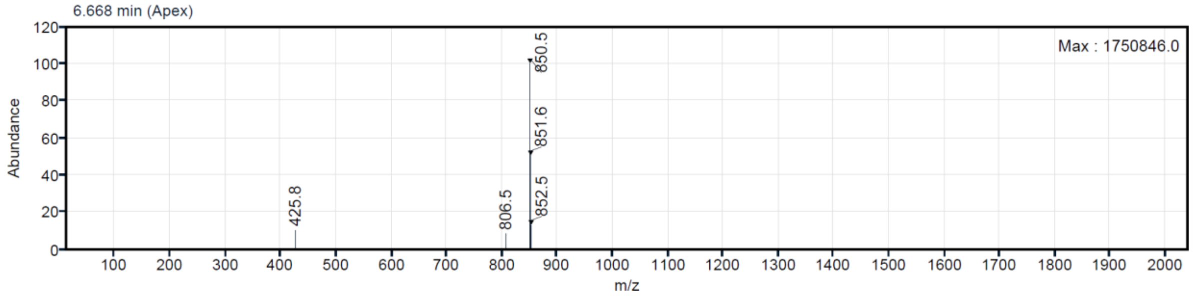

Retention time: 6.734 min **Area Percent:** 62%

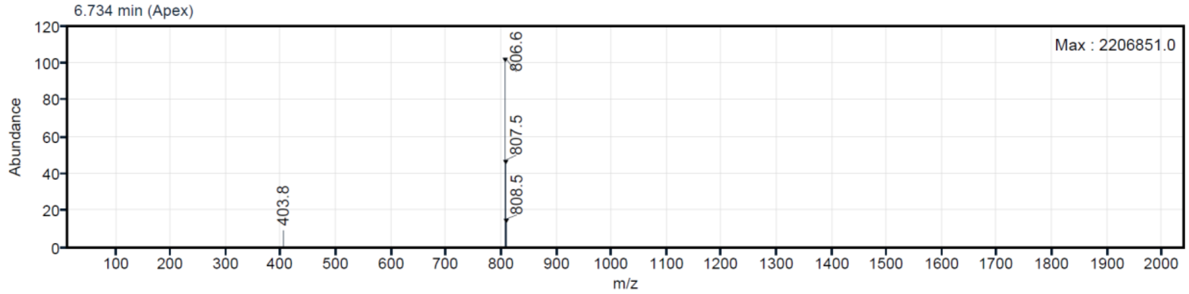

HPLC-UV chromatogram (210 nm) of **1p**:

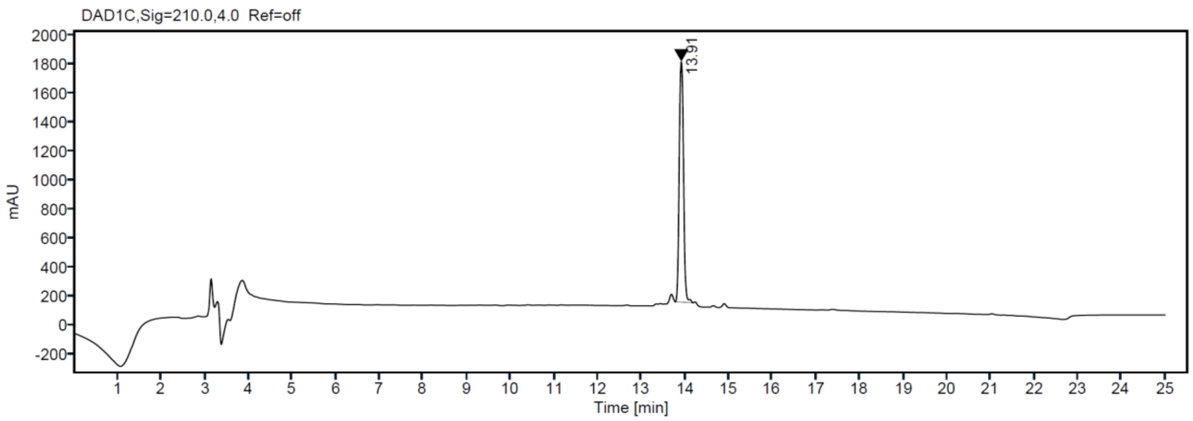

Retention time: 13.997 min **Area Percent:** 100%

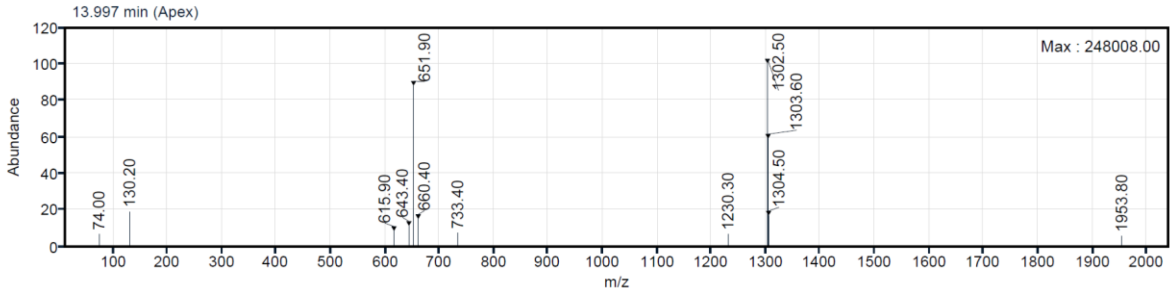

HRMS (Nanochip-based ESI/LTQ-Orbitrap) m/z: [M + H]$^+$ Calcd for $C_{52}H_{55}F_6IN_9O_{16}^+$ 1302.2710; Found 1302.2722.
MS/MS fragmentation of **1p**:

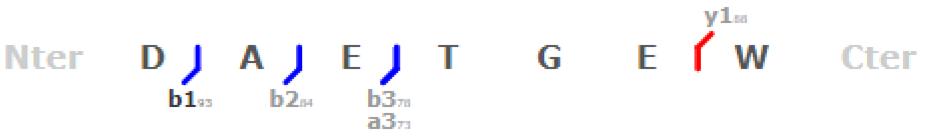

Nter = C18H8IO2F6
Cter = NH2

Sequence	Type	MF	MF Mass	m/z	Intensity	Similarity
DA	b2	C25H18F6IN2O6(+1)	683.0114	683.0108	29.76	93.11%
D	b1	C22H13F6INO5(+1)	611.9743	611.9737	25.18	93.08%
W	y1	C11H14N3O(+1)	204.1137	204.1131	8.53	88.32%
DAE	b3	C30H25F6IN3O9(+1)	812.054	812.0534	7.52	78.19%
DA	b2	C25H18F6IN2O6(+1)	683.0114	228.3418	2.49	75.02%
DAE	a3	C29H25F6IN3O8(+1)	784.0591	784.0585	1	72.83%

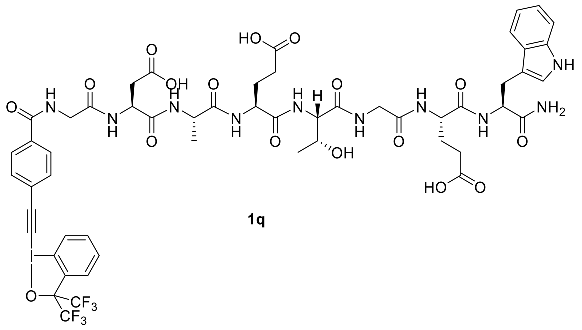

1q

Following the general procedure, the reaction was conducted in 0.021 mmol scale. The desired product **1q** (13.4 mg, 0.010 mmol, 48% yield) was isolated by **Method 3**.

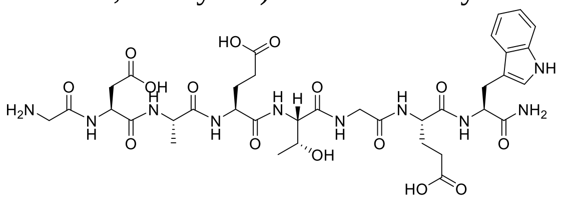

HPLC-UV chromatogram (210 nm) of **GDAETGEW-NH**$_2$:

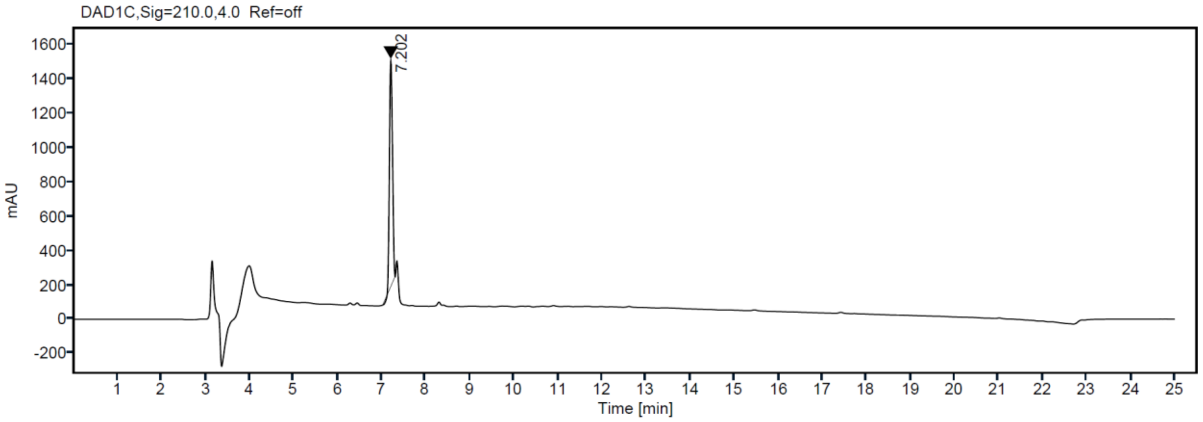

Retention time: 7.228 min **Area Percent:** 100%

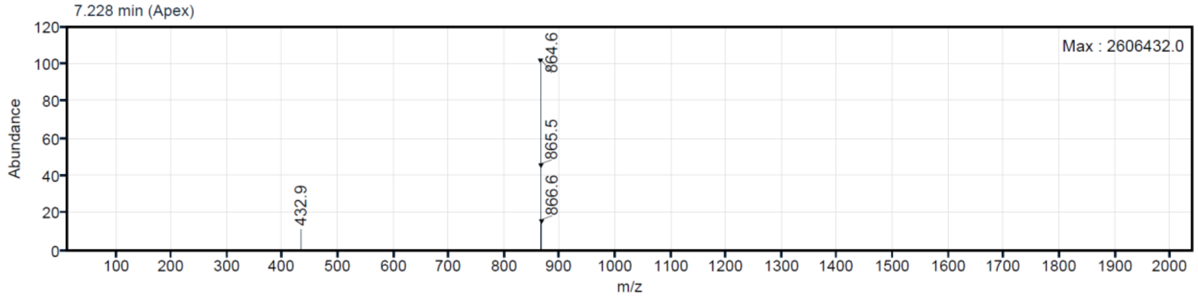

HPLC-UV chromatogram (210 nm) of **1q**:

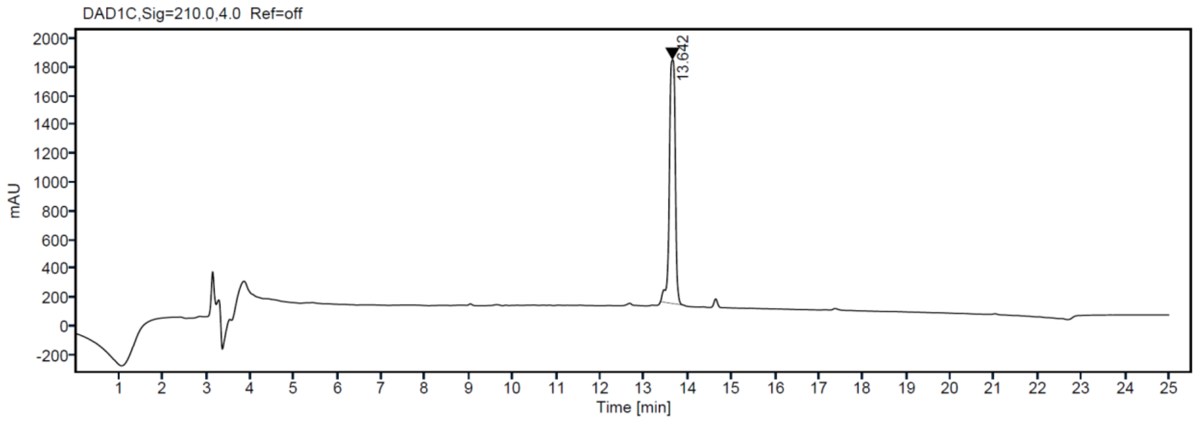

Retention time: 13.758 min **Area Percent:** 100%

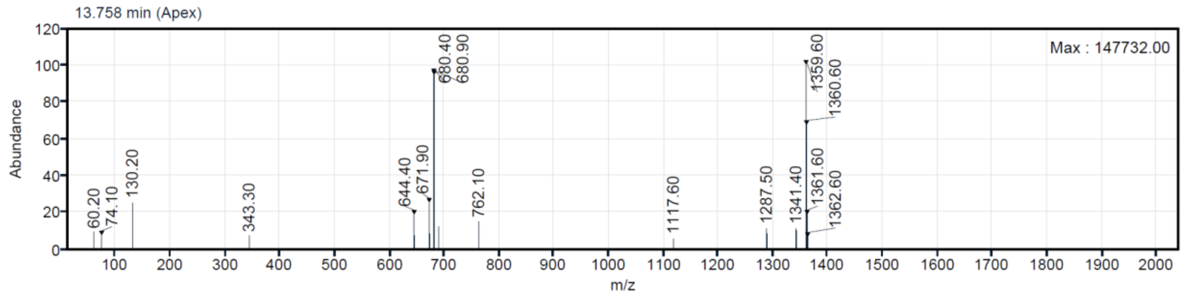

HRMS (Nanochip-based ESI/LTQ-Orbitrap) m/z: $[M + H]^+$ Calcd for $C_{54}H_{58}F_6IN_{10}O_{17}^+$ 1359.2925; Found 1359.2933.

MS/MS fragmentation of **1q**:

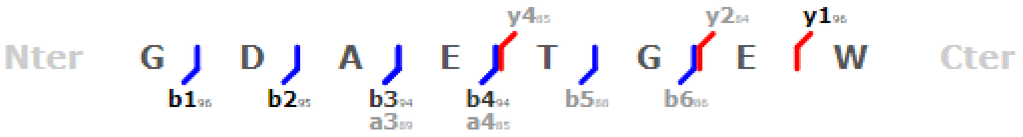

Nter = C18H8IO2F6
Cter = NH2

Sequence	Type	MF	MF Mass	m/z	Intensity	Similarity
G	b1	C20H11F6INO3(+1)	553.9688	553.9682	10.02	95.83%
W	y1	C11H14N3O(+1)	204.1137	204.1131	13.94	95.60%
GD	b2	C24H16F6IN2O6(+1)	668.9957	668.9952	100.03	95.02%
GDA	b3	C27H21F6IN3O7(+1)	740.0328	740.0323	52.02	94.34%
GDAE	b4	C32H28F6IN4O10(+1)	869.0754	869.0749	10.93	93.93%
GDA	a3	C26H21F6IN3O6(+1)	712.0379	712.0374	2.43	89.40%
GDAET	b5	C36H35F6IN5O12(+1)	970.1231	970.1226	3.29	88.17%
GDAETG	b6	C38H38F6IN6O13(+1)	1027.145	1027.144	0.67	86.28%
GDAE	a4	C31H28F6IN4O9(+1)	841.0805	841.08	1.06	85.35%
TGEW	y4	C22H31N6O7(+1)	491.2254	491.2249	0.88	85.09%
EW	y2	C16H21N4O4(+1)	333.1563	167.0815	2.15	84.05%

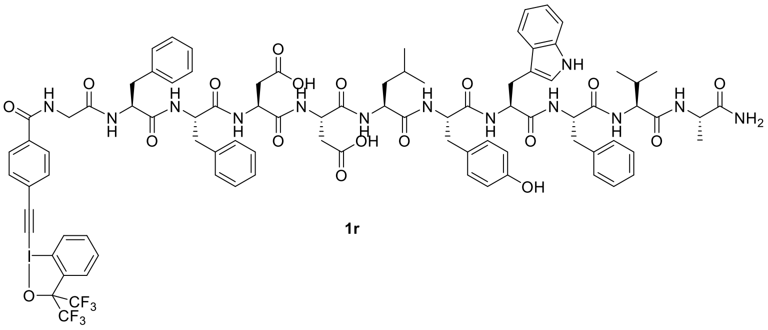

1r

Following the general procedure, the reaction was conducted in 0.024 mmol scale. The desired product **1r** (23.4 mg, 0.0150 mmol, 63% yield) was isolated by **Method 2**.

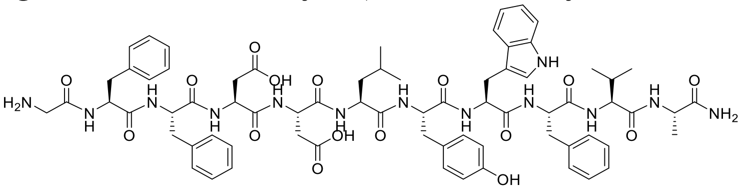

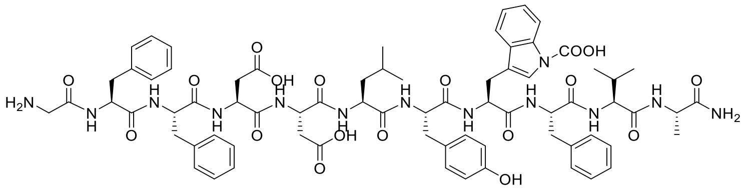

HPLC-UV chromatogram (210 nm) of **GFFDDLYWFVA-NH$_2$:**

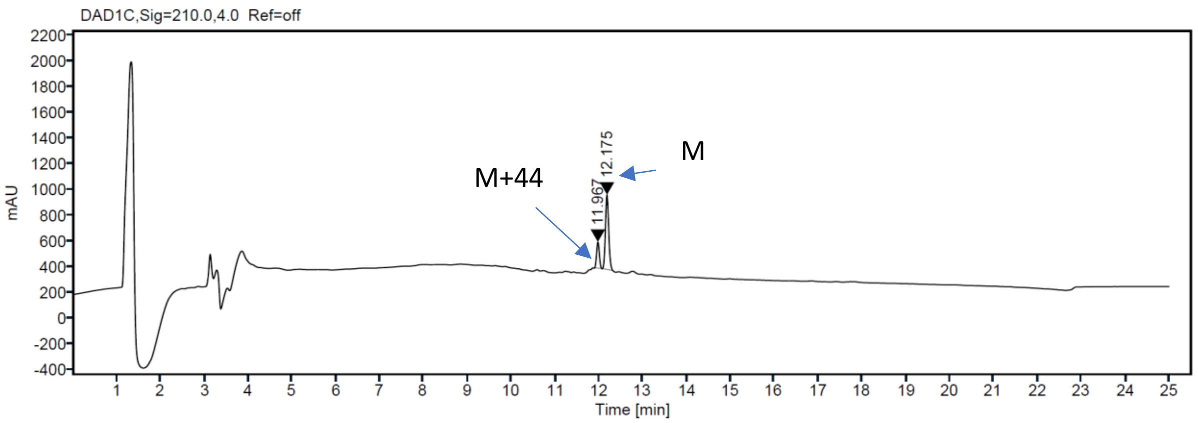

Retention time: 11.977 min **Area Percent:** 53%

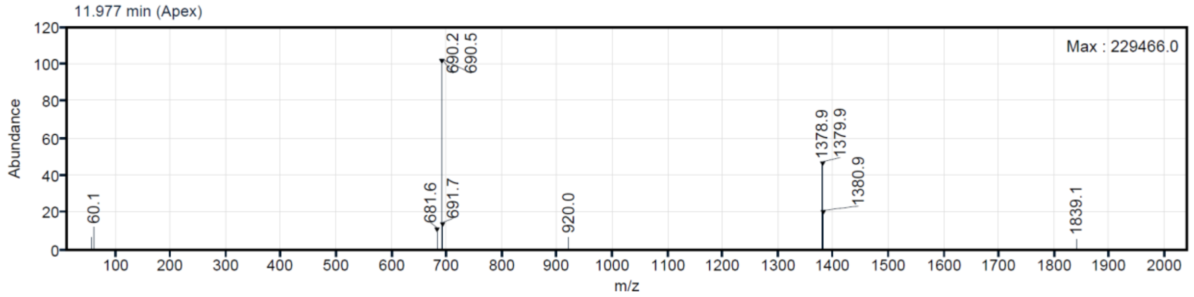

Retention time: 12.223 min **Area Percent:** 47%

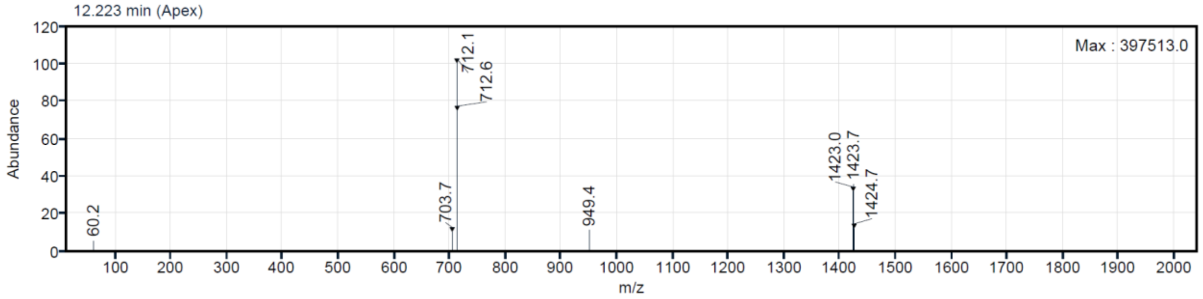

HPLC-UV chromatogram (210 nm) of **1r**:

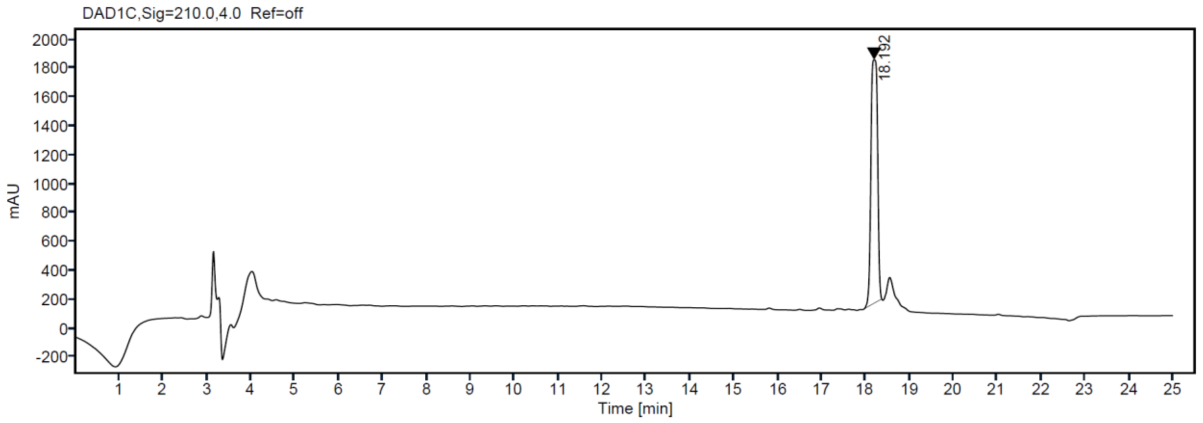

Retention time: 18.235 min **Area Percent:** 100%

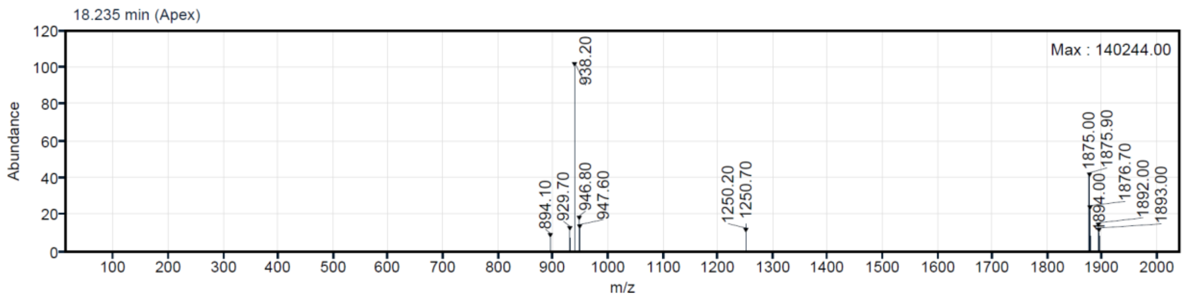

HRMS (Nanochip-based ESI/LTQ-Orbitrap) m/z: $[M + H_2]^{+2}$ Calcd for $C_{89}H_{96}F_6IN_{13}O_{18}{}^{+2}$ 937.7967; Found 937.8001.

MS/MS fragmentation of **1r**:

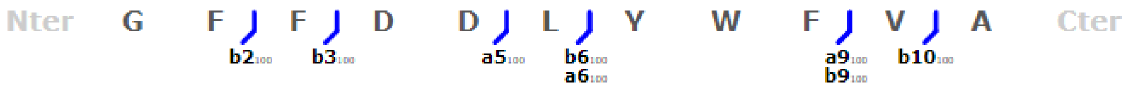

Nter = C18H8F6IO2
Cter = NH2

Sequence	Type	MF	MF Mass	m/z	Similarity
GFF	b3	C38H29F6IN3O5(+1)	848.1056	848.1051	100.00%
GFFDD	a5	C45H39F6IN5O10(+1)	1050.165	1050.164	100.00%
GFFDDLYWF	a9	C80H78F6IN10O15(+1)	1659.46	553.8246	100.00%
GF	b2	C29H20F6IN2O4(+1)	701.0372	701.0366	99.99%
GFFDDLYWF	b9	C81H78F6IN10O16(+1)	1687.455	563.1562	99.95%
GFFDDL	b6	C52H50F6IN6O12(+1)	1191.244	596.1251	99.88%
GFFDDL	a6	C51H50F6IN6O11(+1)	1163.249	582.1277	99.87%
GFFDDLYWFV	b10	C86H87F6IN11O17(+1)	1786.523	596.179	99.76%

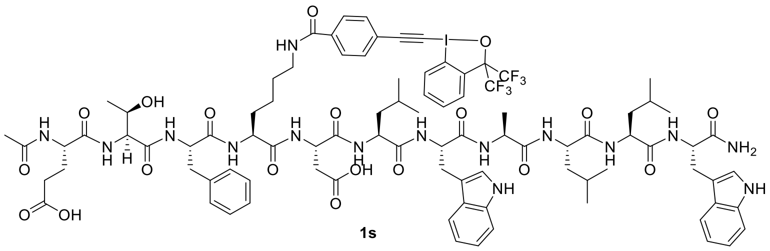

1s

Following the general procedure, the reaction was conducted in 0.014 mmol scale. The desired product **1s** (14.1 mg, 0.00700 mmol, 50% yield) was isolated by **Method 2**.

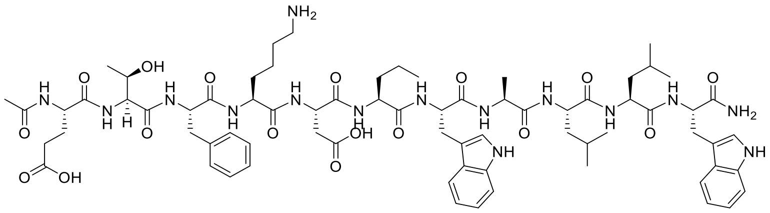

HPLC-UV chromatogram (210 nm) of **AcETFKDLWALLW-NH2**:

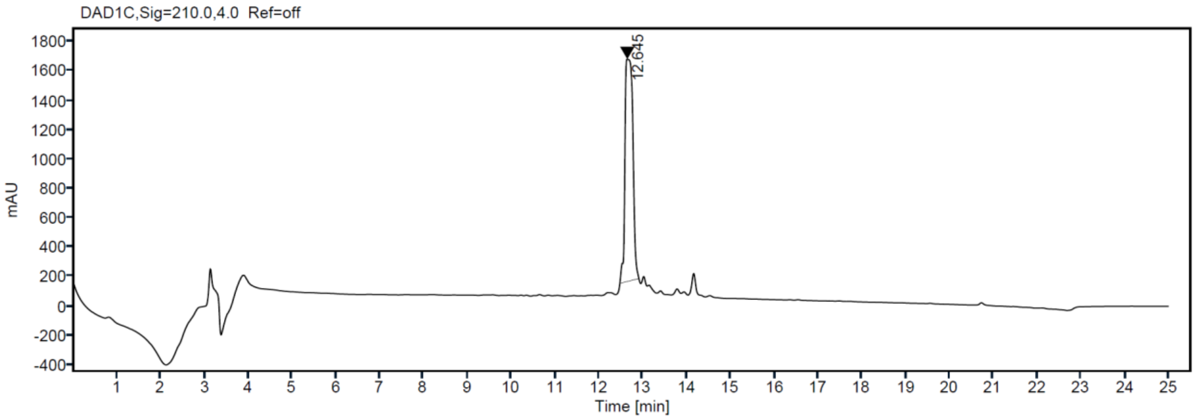

Retention time: 12.633 min **Area Percent:** 100%

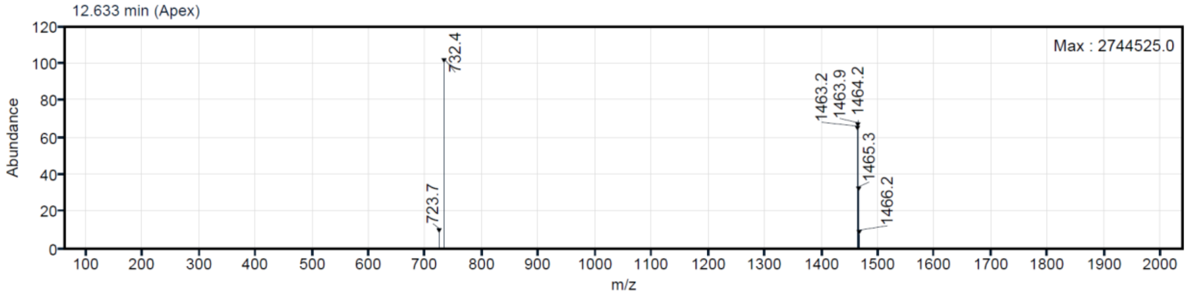

HPLC-UV chromatogram (210 nm) of **1s**:

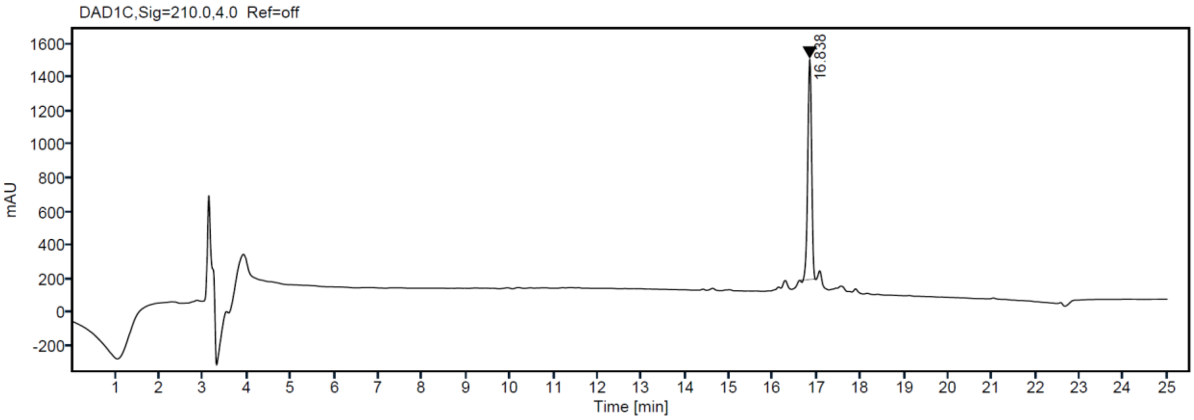

Retention time: 16.914 min **Area Percent:** 100%

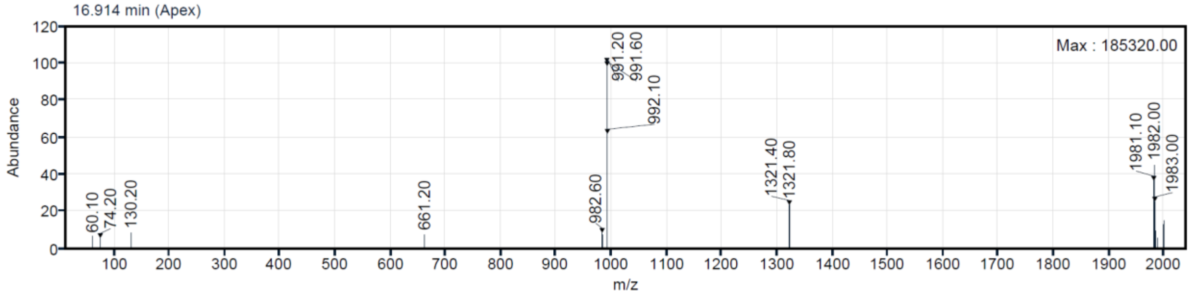

HRMS (Nanochip-based ESI/LTQ-Orbitrap) m/z: $[M + H_2]^{+2}$ Calcd for $C_{93}H_{110}F_6IN_{15}O_{19}^{+2}$ 990.8520; Found 990.8533.

MS/MS fragmentation of **1s**:

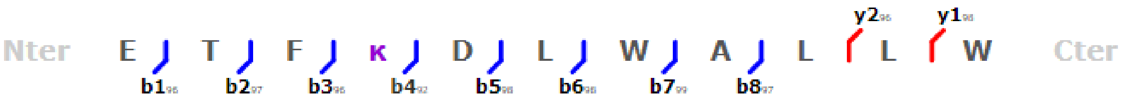

κ = Lys(C18H7F6IO2)
Nter = C2H3O
Cter = NH2

Sequence	Type	MF	MF Mass	m/z	Intensity	Similarity
ETFKDLW	b7	C65H71F6IN9O15(+1)	1458.402	729.7043	5.56	99.80%
ETFKD	b5	C48H50F6IN6O13(+1)	1159.239	1159.238	9.25	98.88%
ETFKDL	b6	C54H61F6IN7O14(+1)	1272.323	1272.322	3.18	98.69%
W	y1	C11H14N3O(+1)	204.1137	204.1131	35.87	98.49%
ETFK	b4	C44H45F6IN5O10(+1)	1044.212	1044.211	2.02	98.36%
ETFKDLW	b7	C65H71F6IN9O15(+1)	1458.402	1458.401	1.71	97.93%
ETFKDLWA	b8	C68H76F6IN10O16(+1)	1529.439	765.2228	1.62	97.55%
ETFKDL	b6	C54H61F6IN7O14(+1)	1272.323	636.6646	2.25	97.24%
ET	b2	C11H17N2O6(+1)	273.1087	273.1081	3.3	97.15%
ETFKDLWA	b8	C68H76F6IN10O16(+1)	1529.439	1529.438	1.62	96.59%
ETFKD	b5	C48H50F6IN6O13(+1)	1159.239	580.1226	1.12	96.39%
LW	y2	C17H25N4O2(+1)	317.1978	317.1972	2.52	95.79%
E	b1	C7H10NO4(+1)	172.061	172.0604	2.01	95.59%
ETF	b3	C20H26N3O7(+1)	420.1771	420.1765	2.86	95.51%
ETFK	b4	C44H45F6IN5O10(+1)	1044.212	522.6091	0.67	86.01%

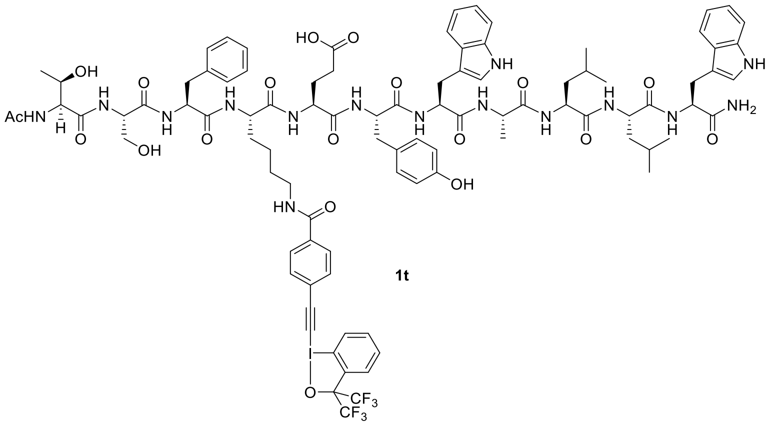

1t

Following the general procedure, the reaction was conducted in 0.014 mmol scale. The desired product **1t** (16 mg, 0.0079 mmol, 57% yield) was isolated by **Method 2**.

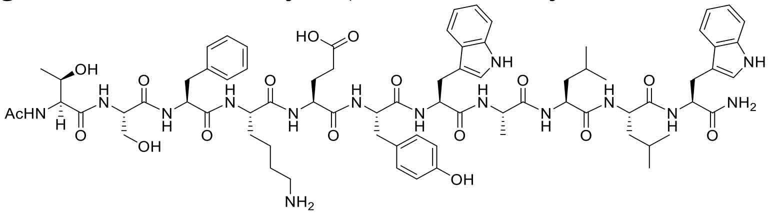

HPLC-UV chromatogram (210 nm) of **AcTSFKEYWALLW-NH2**:

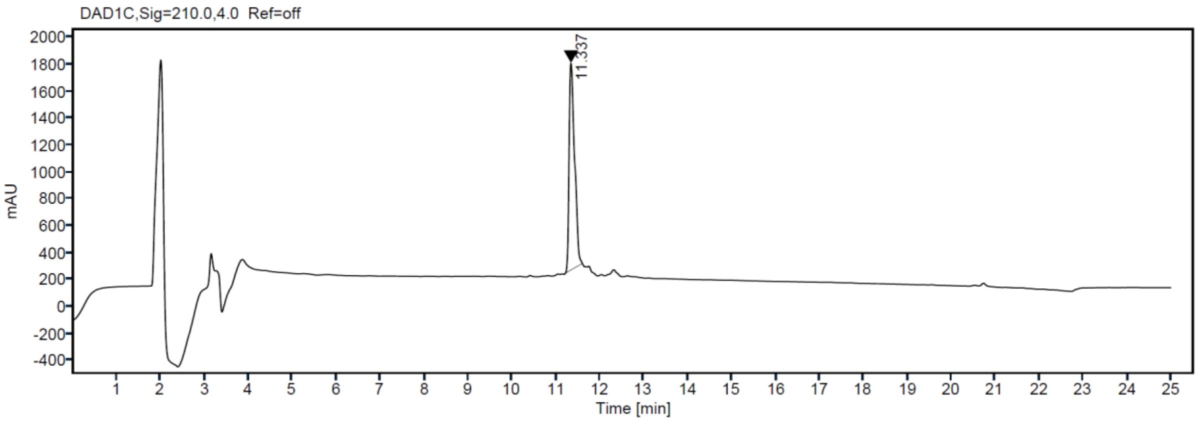

Retention time: 11.365 min **Area Percent:** 100%

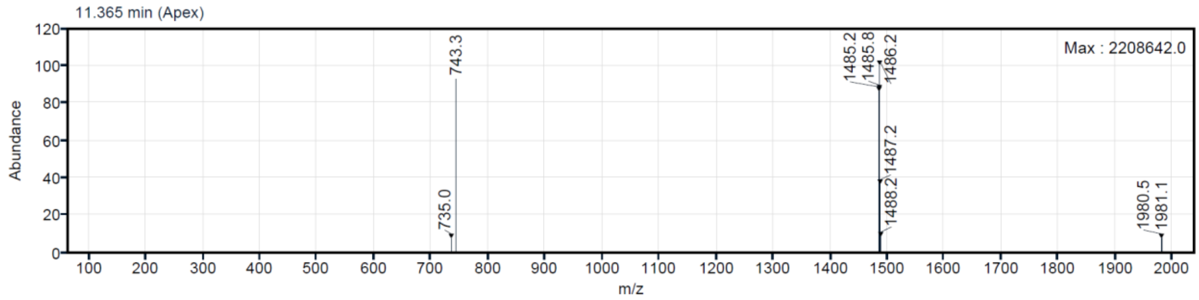

HPLC-UV chromatogram (210 nm) of **1t**:

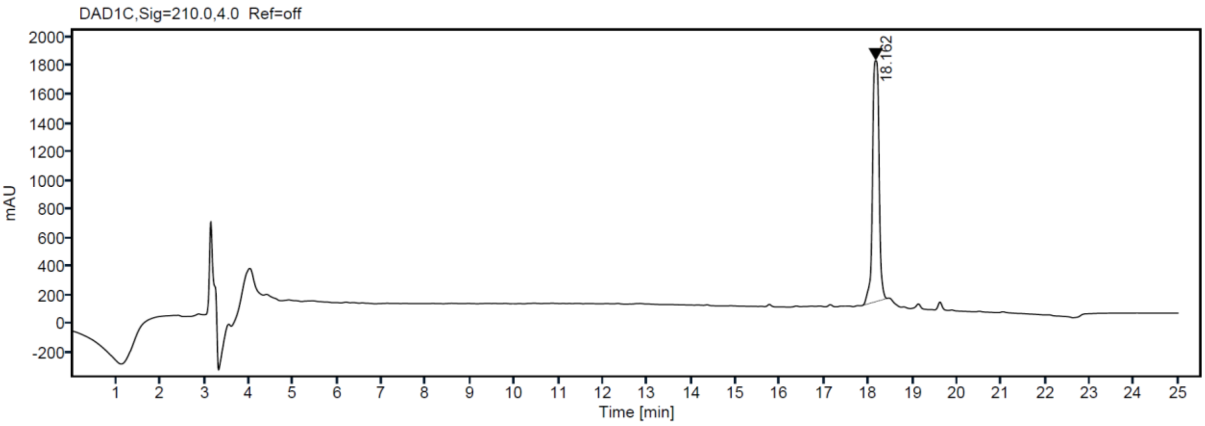

Retention time: 18.155 min **Area Percent:** 100%

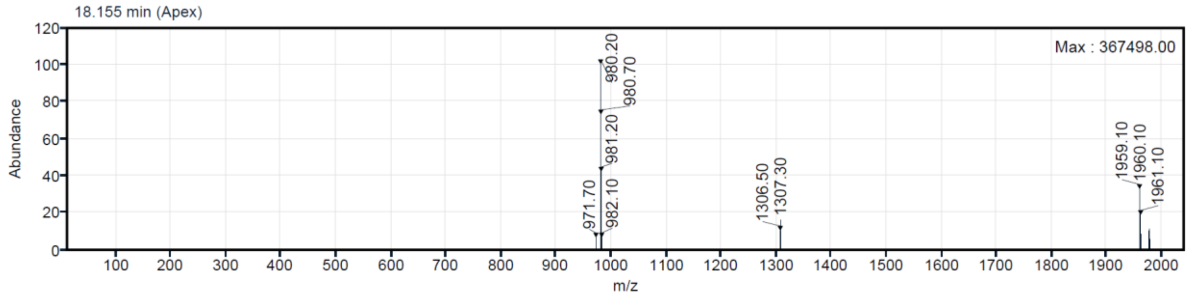

HRMS (Nanochip-based ESI/LTQ-Orbitrap) m/z: $[M + H_2]^{+2}$ Calcd for $C_{91}H_{112}F_6IN_{15}O_{19}^{+2}$ 979.8598; Found 979.8616.

MS/MS fragmentation of **1t**:

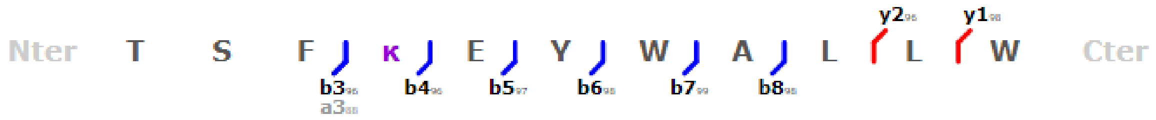

κ = Lys(C18H7IO2F6)
Nter = C2H3O
Cter = NH2

Sequence	Type	MF	MF Mass	m/z	Intensity	Similarity
TSFKEYW	b7	C67H69F6IN9O15(+1)	1480.386	1480.386	0.92	99.02%
TSFKEYWA	b8	C70H74F6IN10O16(+1)	1551.423	1551.423	1.01	98.83%
TSFKEYW	b7	C67H69F6IN9O15(+1)	1480.386	740.6965	2.87	98.37%
TSFKEY	b6	C56H59F6IN7O14(+1)	1294.307	1294.306	1.61	98.12%
TSFKE	b5	C47H50F6IN6O12(+1)	1131.244	1131.243	4.97	97.98%
TSFKEYWA	b8	C70H74F6IN10O16(+1)	1551.423	776.215	0.82	97.81%
W	y1	C11H14N3O(+1)	204.1137	204.1131	34.95	97.80%
TSFKEY	b6	C56H59F6IN7O14(+1)	1294.307	647.6568	1.43	97.45%
TSFK	b4	C42H43F6IN5O9(+1)	1002.201	1002.2	2.72	96.76%
TSFKE	b5	C47H50F6IN6O12(+1)	1131.244	566.1251	1.17	96.36%
TSF	b3	C18H24N3O6(+1)	378.1665	378.166	2.02	96.24%
LW	y2	C17H25N4O2(+1)	317.1978	317.1972	2.3	96.11%
TSFK	b4	C42H43F6IN5O9(+1)	1002.201	501.6038	0.91	95.65%
TSF	a3	C17H24N3O5(+1)	350.1716	350.171	0.59	87.69%

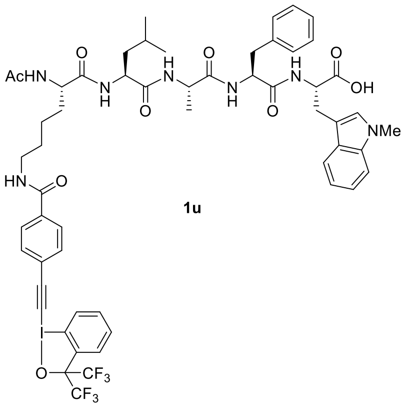

1u

Following the general procedure, the reaction was conducted in 0.03 mmol scale. The desired product **1u** (24 mg, 0.020 mmol, 67% yield) was isolated by **Method 2**.

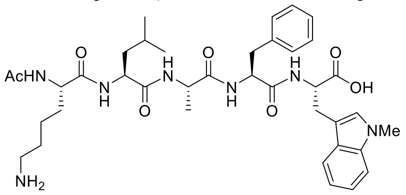

HPLC-UV chromatogram (210 nm) of **Ac-KLAFW(N-Me)**:

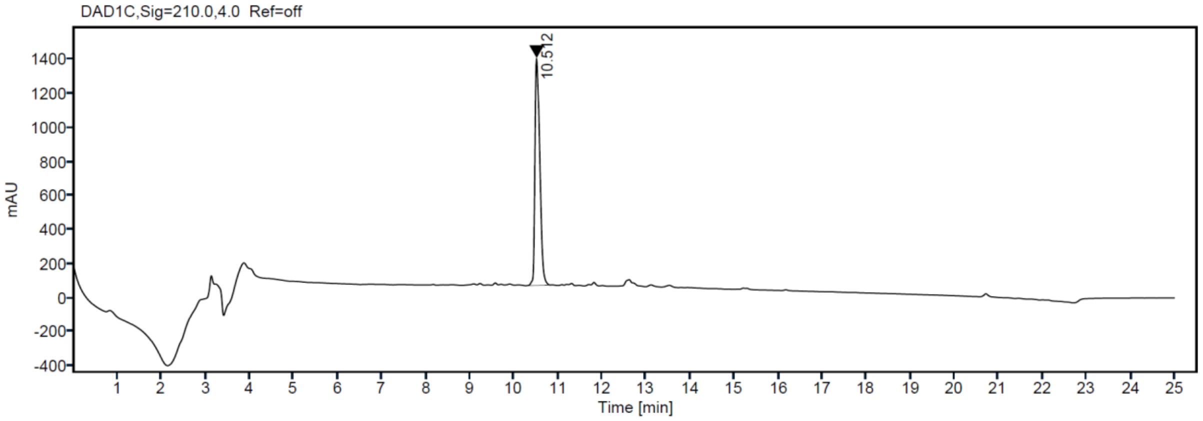

Retention time: 10.552 min **Area Percent:** 100%

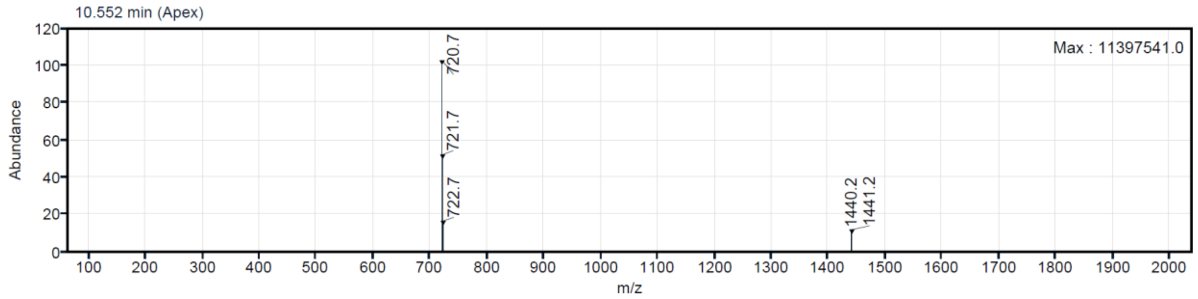

HPLC-UV chromatogram (210 nm) of **1u**:

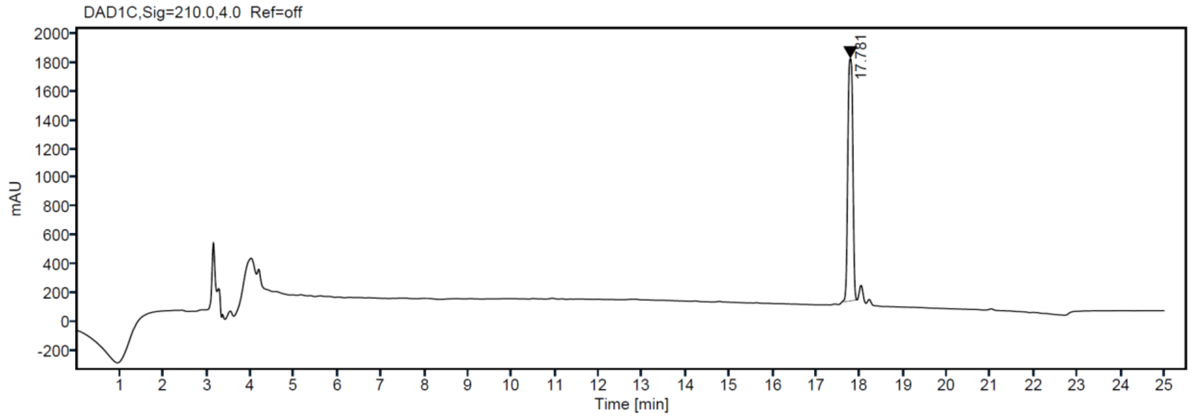

Retention time: 17.881 min **Area Percent:** 100%

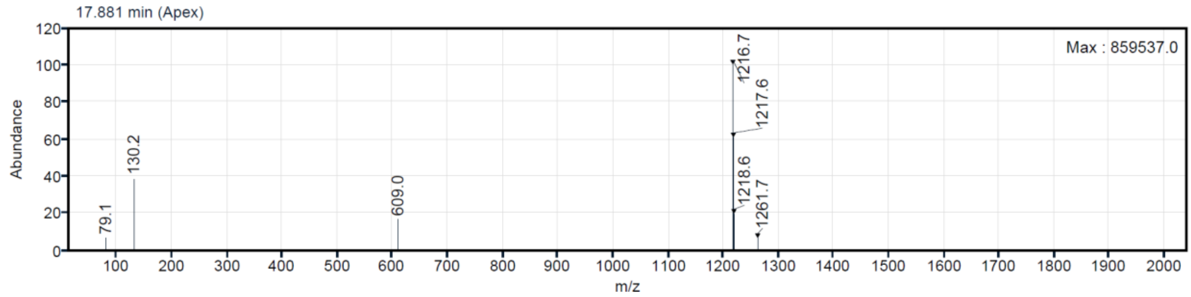

HRMS (Nanochip-based ESI/LTQ-Orbitrap) m/z: $[M + H]^+$ Calcd for $C_{56}H_{61}F_6IN_7O_9^+$ 1216.3474; Found 1216.3475.

MS/MS fragmentation of **1u**:

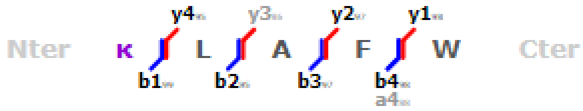

κ = Lys(C18H7F6IO2)
Nter = C2H3O
Cter = CH3O

Sequence	Type	MF	MF Mass	m/z	Intensity	Similarity
KLA	b3	C35H38F6IN4O6(+1)	851.174	851.1735	83.3	98.65%
KLAF	b4	C44H47F6IN5O7(+1)	998.2424	998.2419	18.45	98.59%
K	b1	C26H22F6IN2O4(+1)	667.0528	667.0523	39.09	98.58%
W	y1	C12H15N2O2(+1)	219.1134	219.1128	100.2	98.21%
KLAF	b4	C44H47F6IN5O7(+1)	998.2424	499.6246	18.93	98.10%
KL	b2	C32H33F6IN3O5(+1)	780.1369	780.1364	49.54	98.06%
FW	y2	C21H24N3O3(+1)	366.1818	366.1812	60.57	97.24%
KLA	b3	C35H38F6IN4O6(+1)	851.174	426.0904	20.66	96.12%
LAFW	y4	C30H40N5O5(+1)	550.3029	550.3024	6.31	95.12%
KLAF	a4	C43H47F6IN5O6(+1)	970.2475	970.247	2.51	93.67%
AFW	y3	C24H29N4O4(+1)	437.2189	437.2183	4.47	93.51%
KL	b2	C32H33F6IN3O5(+1)	780.1369	390.5718	1.27	92.85%
KLAF	a4	C43H47F6IN5O6(+1)	970.2475	485.6271	0.7	81.60%
AFW	y3	C24H29N4O4(+1)	437.2189	219.1128	100.2	78.60%

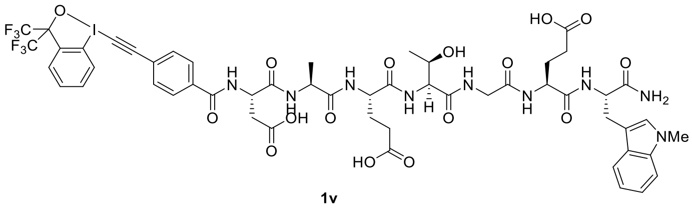

1v

Following the general procedure, the reaction was conducted in 0.014 mmol scale. The desired product **1v** (10 mg, 0.0079 mmol, 63% yield) was isolated by **Method 3**.

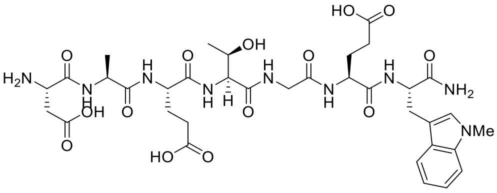

HPLC-UV chromatogram (210 nm) of **DAETGEW(N-Me)-NH$_2$**:

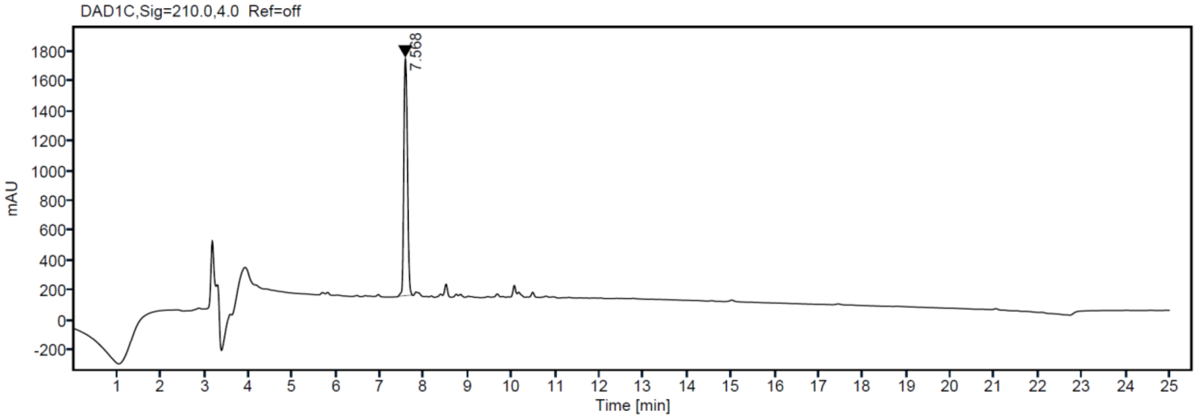

Retention time: 7.597 min **Area Percent:** 100%

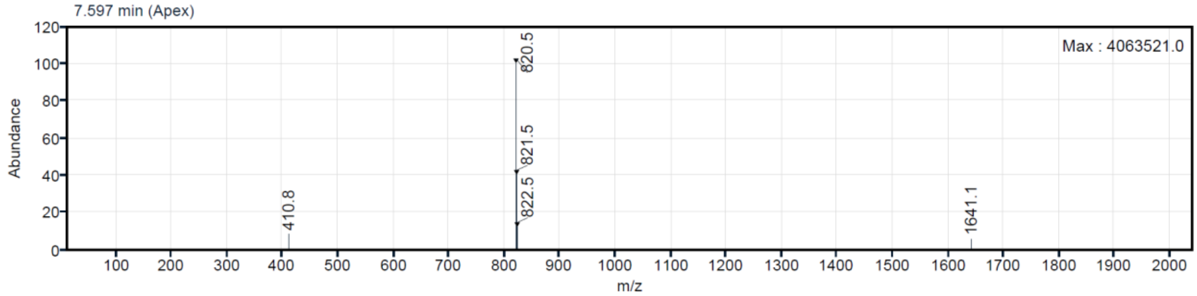

HPLC-UV chromatogram (210 nm) of **1v**:

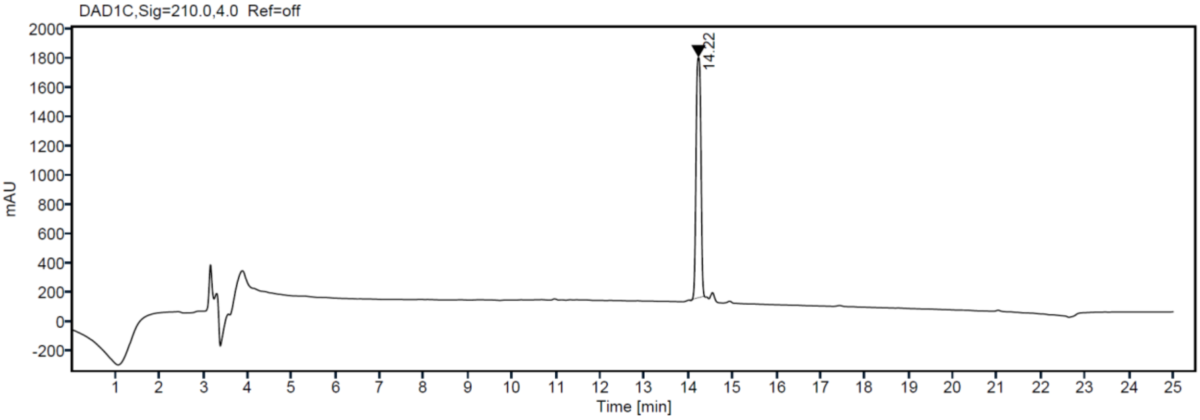

Retention time: 14.232 min **Area Percent:** 100%

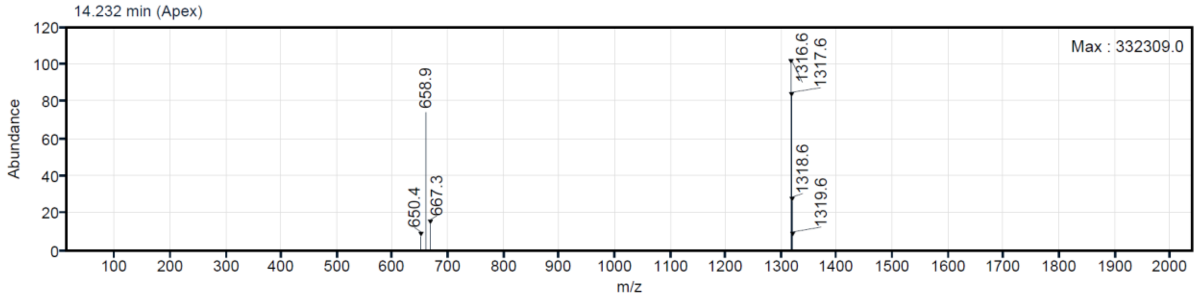

HRMS (Nanochip-based ESI/LTQ-Orbitrap) m/z: $[M + H]^+$ Calcd for $C_{53}H_{57}F_6IN_9O_{16}^+$ 1316.2867; Found 1316.2878.

MS/MS fragmentation of **1v**:

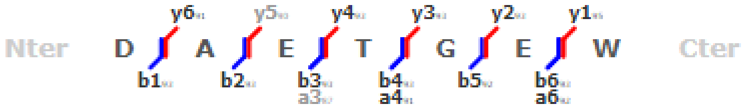

Nter = C18H8IO2F6
Cter = CH2NH2

Sequence	Type	MF	MF Mass	m/z	Intensity	Similarity
W	y1	C12H16N3O(+1)	218.1293	218.1288	8.99	94.87%
DA	b2	C25H18F6IN2O6(+1)	683.0114	683.0108	6.14	93.18%
DAETGE	b6	C41H42F6IN6O15(+1)	1099.1657	1099.165	48.84	93.13%
D	b1	C22H13F6INO5(+1)	611.9743	611.9737	3.84	93.03%
DAET	b4	C34H32F6IN4O11(+1)	913.1016	913.1011	35.71	92.82%
DAE	b3	C30H25F6IN3O9(+1)	812.054	812.0534	28.06	92.79%
GEW	y3	C19H26N5O5(+1)	404.1934	404.1928	2.46	92.69%
TGEW	y4	C23H33N6O7(+1)	505.2411	505.2405	7.38	92.63%
EW	y2	C17H23N4O4(+1)	347.1719	347.1714	2.2	92.63%
DAETGE	a6	C40H42F6IN6O14(+1)	1071.1708	1071.17	7.12	92.44%
DAETG	b5	C36H35F6IN5O12(+1)	970.1231	970.1226	12.76	92.01%
AETGEW	y6	C31H45N8O11(+1)	705.3208	705.3202	1.89	90.74%
DAET	a4	C33H32F6IN4O10(+1)	885.1067	885.1062	2.6	90.62%
ETGEW	y5	C28H40N7O10(+1)	634.2837	634.2831	1.2	89.52%
DAE	a3	C29H25F6IN3O8(+1)	784.0591	784.0585	1.97	87.32%

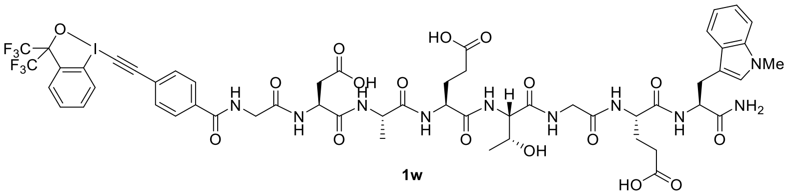

1w

Following the general procedure, the reaction was conducted in 0.014 mmol scale. The desired product **1w** (10 mg, 0.0079 mmol, 63% yield) was isolated by **Method 3**.

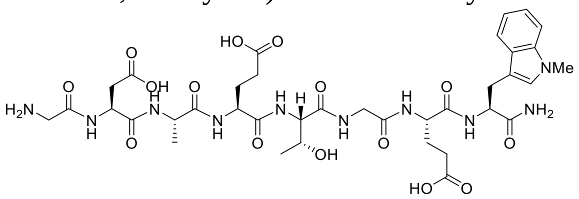

HPLC-UV chromatogram (210 nm) of **GDAETGEW(N-Me)-NH**$_2$:

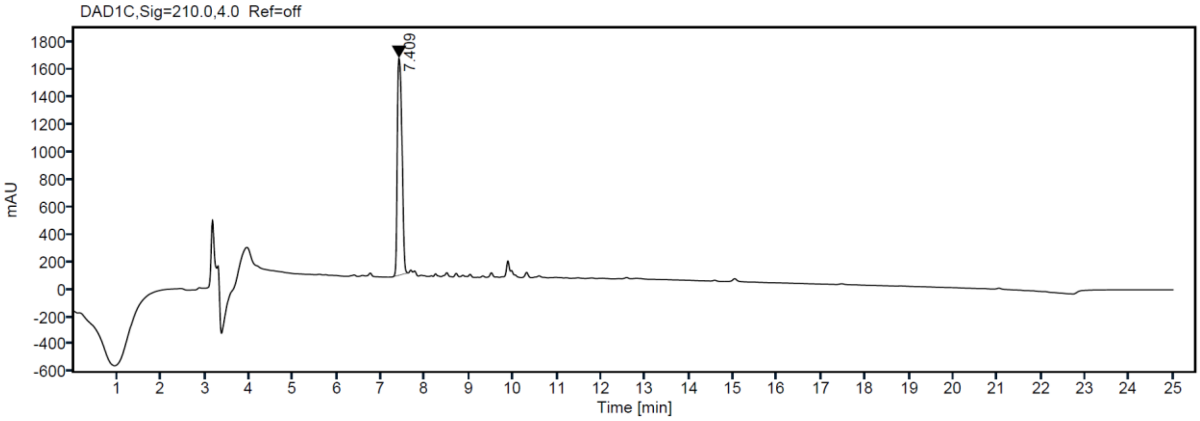

Retention time: 7.438 min **Area Percent:** 100%

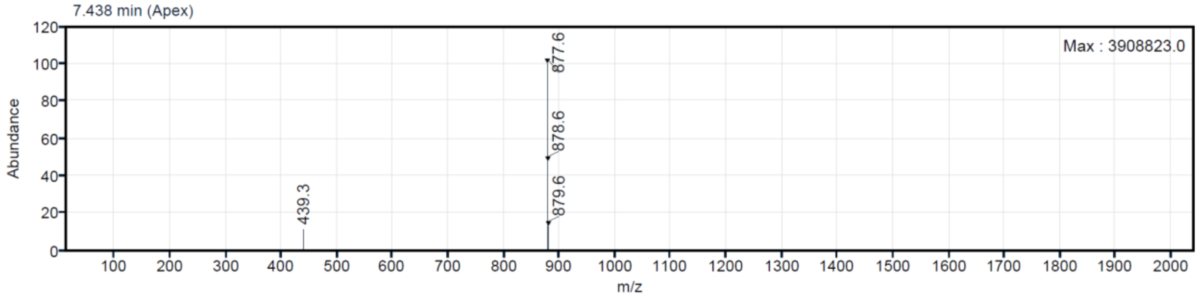

HPLC-UV chromatogram (210 nm) of **1w.** The M-18 peak correspond to a side product formed by cyclization of Asp on the close amide:

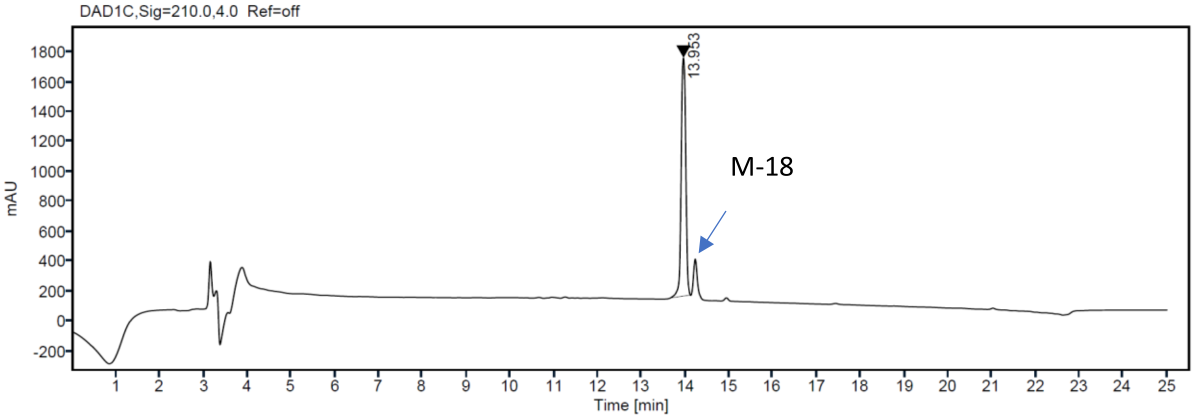

Retention time: 14.02 min **Area Percent:** 100%

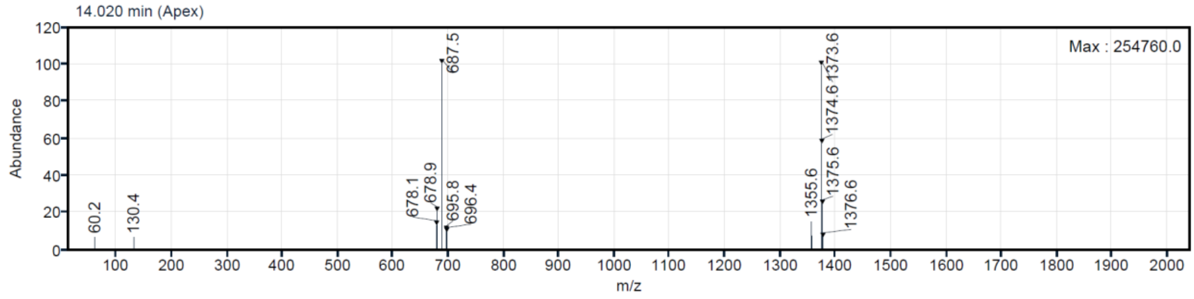

HRMS (Nanochip-based ESI/LTQ-Orbitrap) m/z: $[M + H]^+$ Calcd for $C_{55}H_{60}F_6IN_{10}O_{17}^+$ 1373.3081; Found 1373.3082.

MS/MS fragmentation of **1w**:

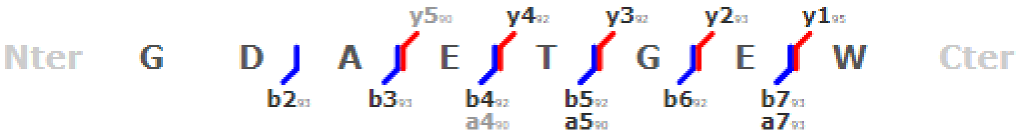

Nter = C18H8IO2F6
Cter = CH4N

Sequence	Type	MF	MF Mass	m/z	Intensity	Similarity
W	y1	C12H16N3O(+1)	218.1293	218.1288	3.11	94.68%
GDAETGEW		C55H59F6IN10O17	1372.301	1373.308	10.86	94.63%
GDAETGE	b7	C43H45F6IN7O16(+1)	1156.187	1156.187	54.51	93.39%
GD	b2	C24H16F6IN2O6(+1)	668.9957	668.9952	2.21	93.21%
GDAETGE	a7	C42H45F6IN7O15(+1)	1128.192	1128.192	5.44	92.88%
GDA	b3	C27H21F6IN3O7(+1)	740.0328	740.0323	7.73	92.71%
EW	y2	C17H23N4O4(+1)	347.1719	347.1714	1.32	92.68%
GDAETG	b6	C38H38F6IN6O13(+1)	1027.145	1027.144	6.92	92.37%
GDAE	b4	C32H28F6IN4O10(+1)	869.0754	869.0749	24.44	92.35%
GDAET	b5	C36H35F6IN5O12(+1)	970.1231	970.1226	21.85	92.14%
TGEW	y4	C23H33N6O7(+1)	505.2411	505.2405	8.01	92.00%
GEW	y3	C19H26N5O5(+1)	404.1934	404.1928	1.45	92.00%
GDAET	a5	C35H35F6IN5O11(+1)	942.1282	942.1276	0.93	90.13%
ETGEW	y5	C28H40N7O10(+1)	634.2837	634.2831	1.71	89.89%
GDAE	a4	C31H28F6IN4O9(+1)	841.0805	841.08	1.15	89.57%

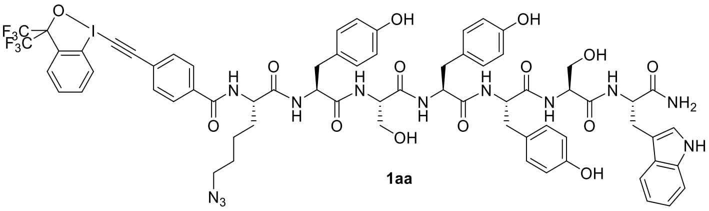

1aa

Following the general procedure, the reaction was conducted in 0.024 mmol scale. The desired product **1aa** (19 mg, 0.012 mmol, 52% yield) was isolated by **Method 2**.

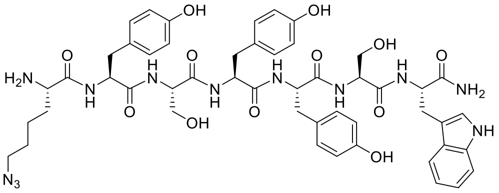

HPLC-UV chromatogram (210 nm) of **K(N$_3$)YSYYSW-NH$_2$**:

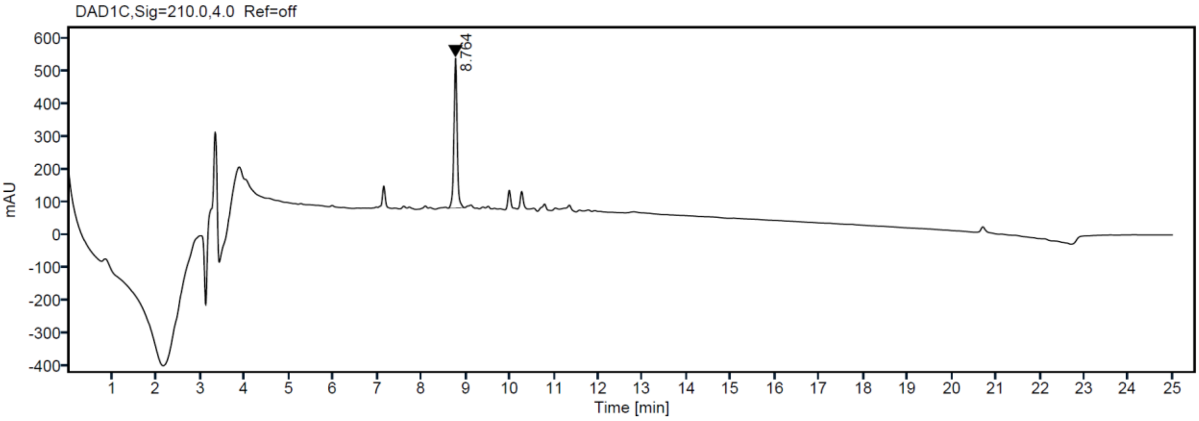

Retention time: 8.796 min **Area Percent:** 100%

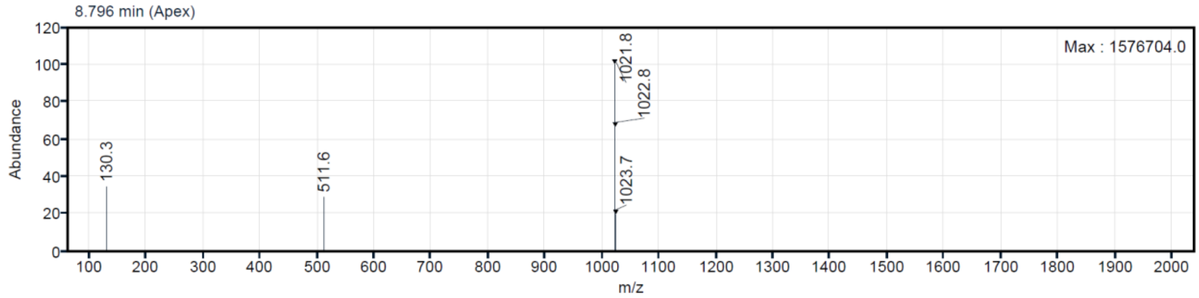

HPLC-UV chromatogram (210 nm) of **1aa**:

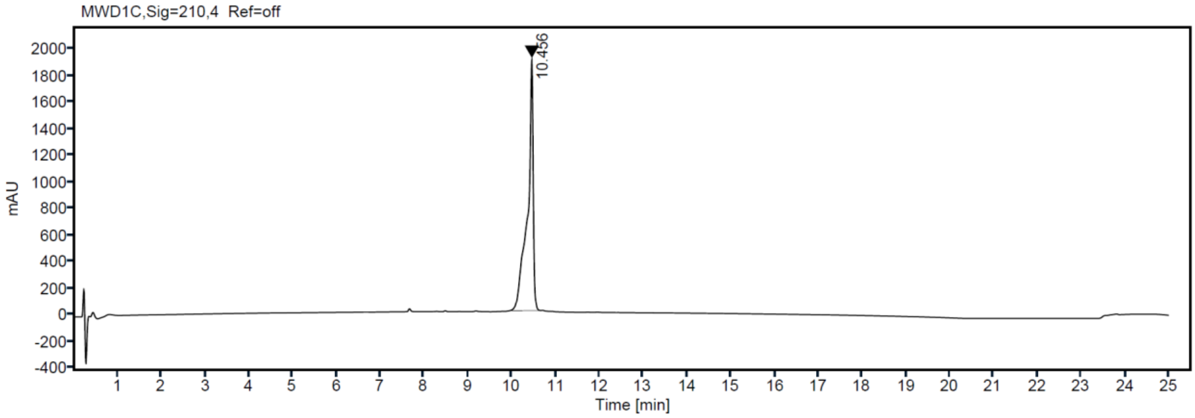

Retention time: 10.481 min **Area Percent:** 100%

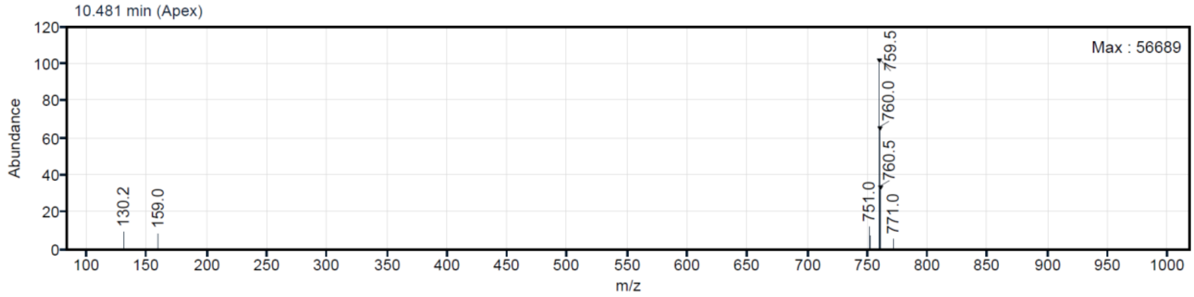

HRMS (nanochip-ESI/LTQ-Orbitrap) m/z: $[M + H_2]^{+2}$ Calcd for $C_{68}H_{69}F_6IN_{12}O_{14}^{+2}$ 759.1997; Found 759.2002.

MS/MS fragmentation of **1aa**:

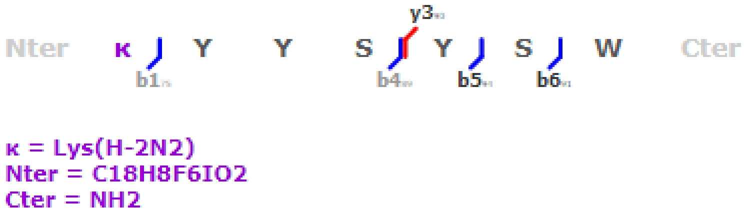

Sequence	Type	MF	MF Mass	m/z	Intensity	Similarity
KYYSYS	b6	C57H55F6IN9O13(+1)	1314.287	1314.286	6.07	97.07%
KYYSY	b5	C54H50F6IN8O11(+1)	1227.255	1227.254	3.15	94.12%
YSW	y3	C23H28N5O5(+1)	454.209	454.2085	2.05	90.14%
KYYS	b4	C45H41F6IN7O9(+1)	1064.192	1064.191	1.63	89.26%
KYYSYS	b6	C57H55F6IN9O13(+1)	1314.287	657.6468	0.53	84.48%
K	b1	C24H18F6IN4O3(+1)	651.0328	651.0322	0.52	75.44%

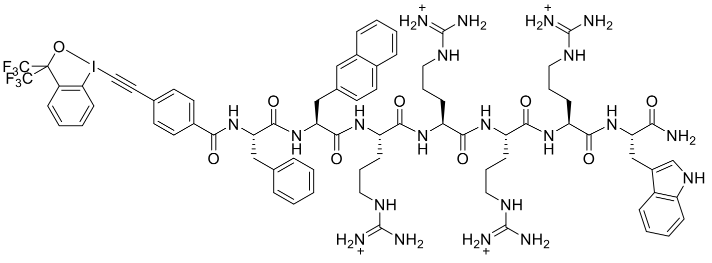

1ab

Following the general procedure, the reaction was conducted in 0.011 mmol scale. The desired product **1ab** (11.8 mg, 0.00775 mmol, 69% yield) was isolated by **Method 2**.

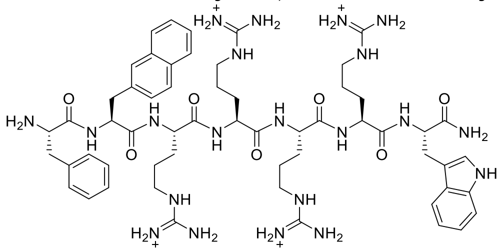

HPLC-UV chromatogram (210 nm) of **FΦRRRRW-NH**$_2$:

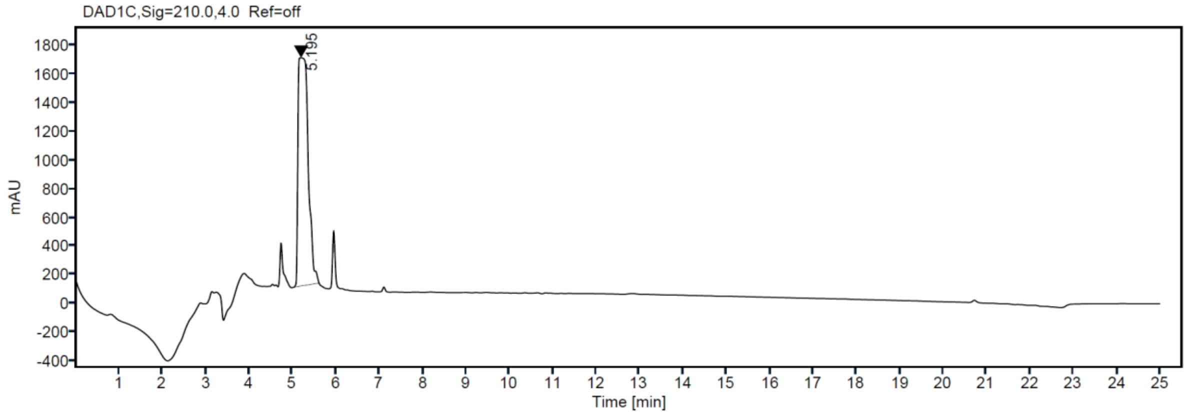

Retention time: 5.202 min **Area Percent:** 100%

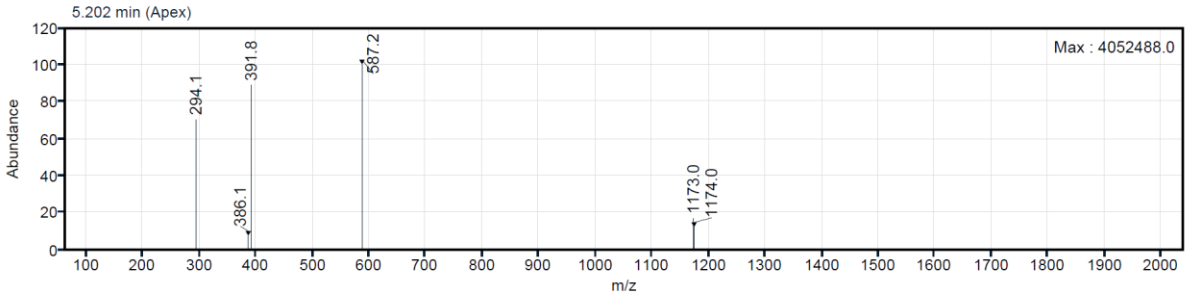

HPLC-UV chromatogram (210 nm) of **1ab**:

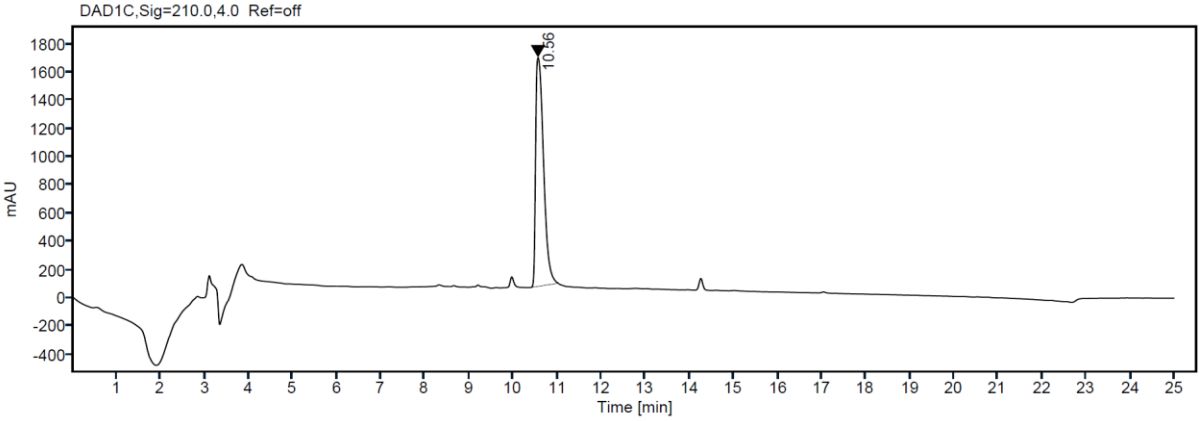

Retention time: 10.603 min **Area Percent:** 100%

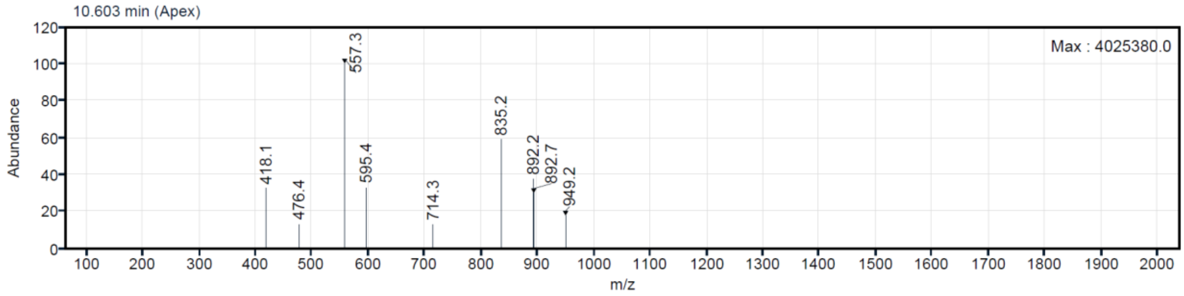

HRMS (nanochip-ESI/LTQ-Orbitrap) m/z: $[M + H_2]^{+2}$ Calcd for $C_{75}H_{90}F_6IN_{21}O_9^{+2}$ 834.8084; Found 834.8082.

MS/MS fragmentation of **1ab**:

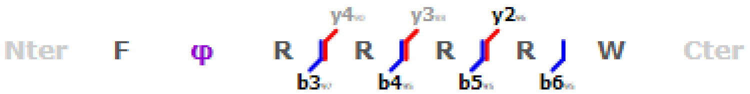

φ = Phe(C4H2)
Nter = C18H8F6IO2
Cter = NH2

Sequence	Type	MF	MF Mass	m/z	Intensity	Similarity
FFR	b3	C46H40F6IN6O5(+1)	997.2009	997.2004	5.53	96.55%
RW	y2	C17H26N7O2(+1)	360.2148	360.2142	7.28	95.51%
FFRRR	b5	C58H64F6IN14O7(+1)	1309.4031	1309.403	3.74	95.23%
FFRRRR	b6	C64H76F6IN18O8(+1)	1465.5042	733.2555	7.61	95.05%

FFRR	b4	C52H52F6IN10O6(+1)	1153.302	1153.302	3.95	95.01%
RRRW	y4	C29H50N15O4(+1)	672.417	672.4165	5.11	89.61%
RRW	y3	C23H38N11O3(+1)	516.3159	516.3154	4.93	88.40%

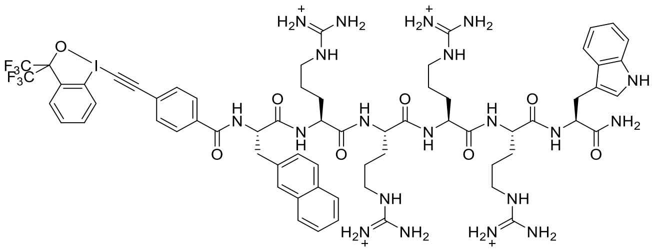

1ac

Following the general procedure, the reaction was conducted in 0.011 mmol scale. The desired product **1ac** (11.8 mg, 0.00775 mmol, 69% yield) was isolated by **Method 2**.

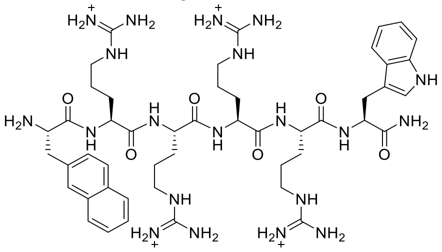

HPLC-UV chromatogram (210 nm) of **ΦRRRRW-NH$_2$**:

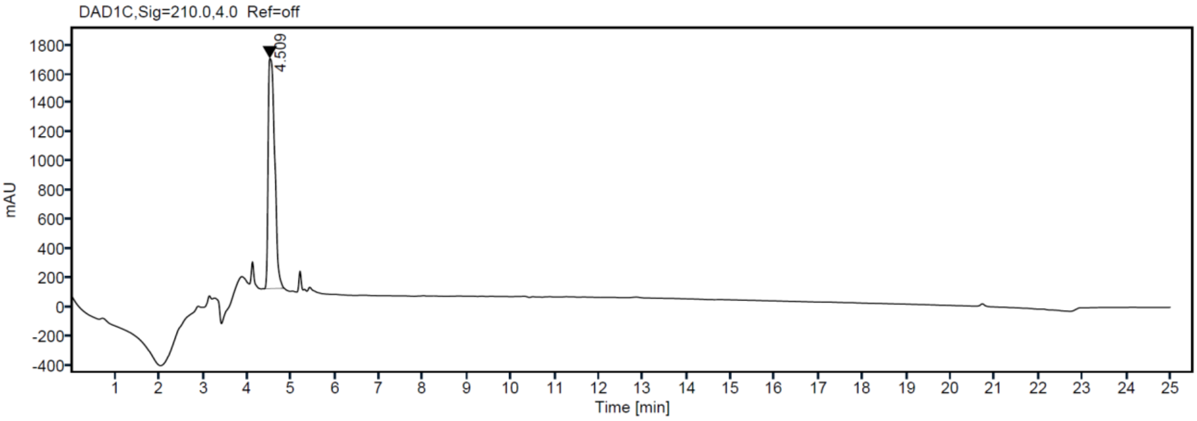

Retention time: 4.547 min **Area Percent:** 100%

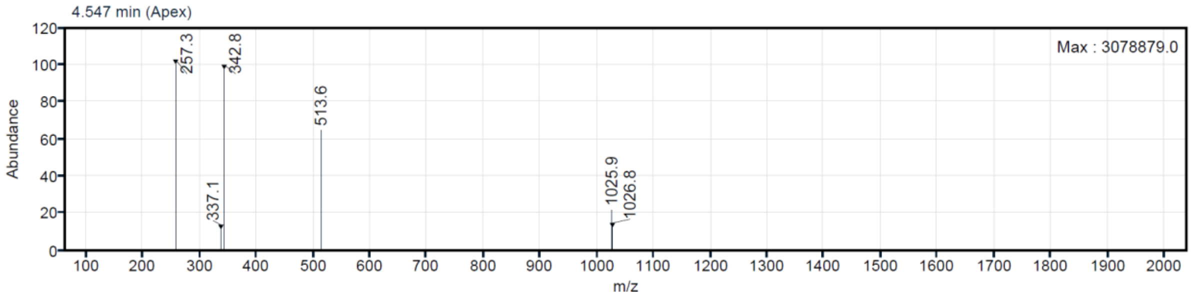

HPLC-UV chromatogram (210 nm) of **1af**:

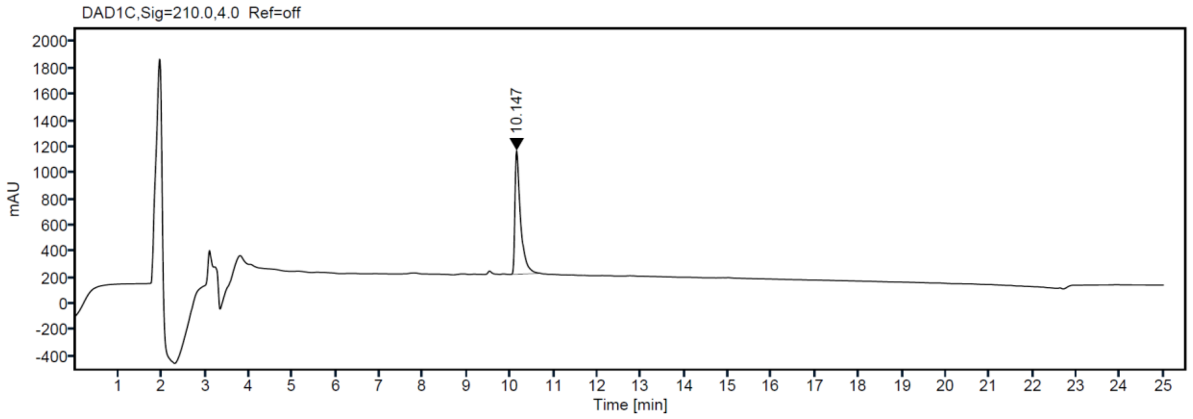

Retention time: 10.161 min **Area Percent:** 100%

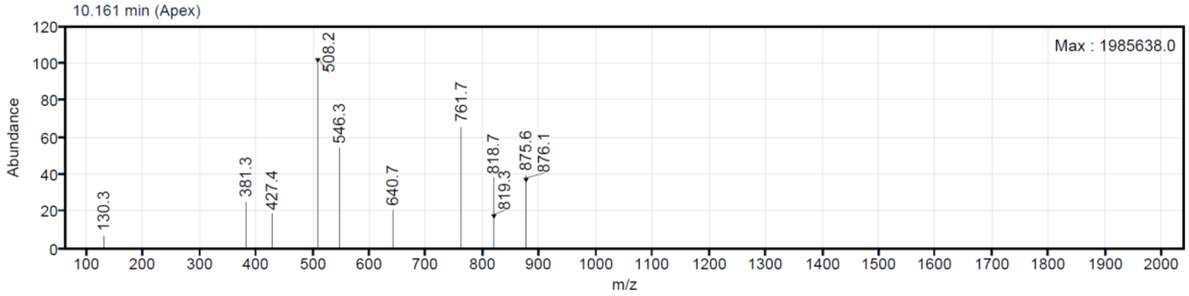

HRMS (nanochip-ESI/LTQ-Orbitrap) m/z: $[M + H_2]^{+2}$ Calcd for $C_{66}H_{81}F_6IN_{20}O_8^{+2}$ 761.2742; Found 761.2750.

MS/MS fragmentation of **1ac**:

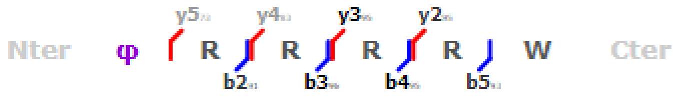

φ = Phe(C4H2)
Nter = C18H8F6IO2
Cter = NH2

Sequence	Type	MF	MF Mass	m/z	Intensity	Similarity
FRR	b3	C43H43F6IN9O5(+1)	1006.2336	1006.233	4.03	95.82%
RRW	y3	C23H38N11O3(+1)	516.3159	516.3154	5.32	95.23%
FRRR	b4	C49H55F6IN13O6(+1)	1162.3347	1162.334	3.15	95.03%
RW	y2	C17H26N7O2(+1)	360.2148	360.2142	7.33	94.60%
FRRRR	b5	C55H67F6IN17O7(+1)	1318.4358	659.7213	6.57	92.96%
FR	b2	C37H31F6IN5O4(+1)	850.1325	850.1319	8.14	91.09%
RRRW	y4	C29H50N15O4(+1)	672.417	672.4165	7.26	90.89%
FRRRRW		C66H79F6IN20O8	1520.5339	507.8519	0.81	88.93%
RRRW	y4	C29H50N15O4(+1)	672.417	336.7119	0.83	74.61%
RRRRW	y5	C35H62N19O5(+1)	828.5181	414.7624	1.73	73.19%

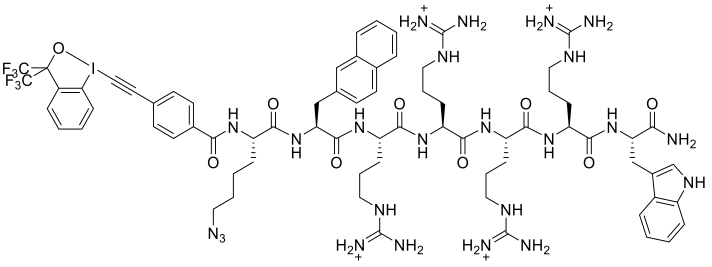

1ad

Following the general procedure, the reaction was conducted in 0.012 mmol scale. The desired product **1ad** (12 mg, 0.0076 mmol, 72% yield) was isolated by **Method 2**.

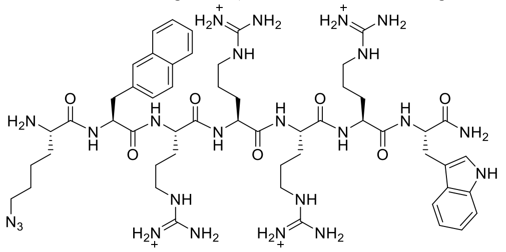

HPLC-UV chromatogram (210 nm) of **K(N$_3$)FRRRRW-NH$_2$**:

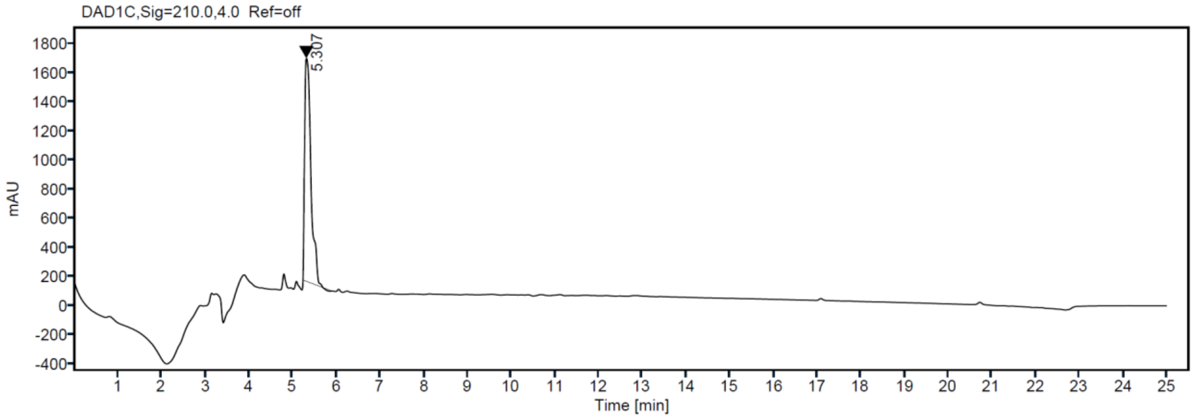

Retention time: 5.316 min **Area Percent:** 100%

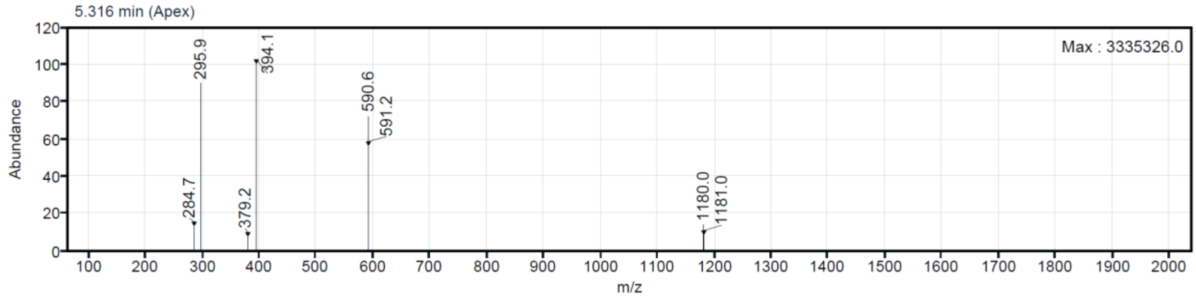

HPLC-UV chromatogram (210 nm) of **1ad**:

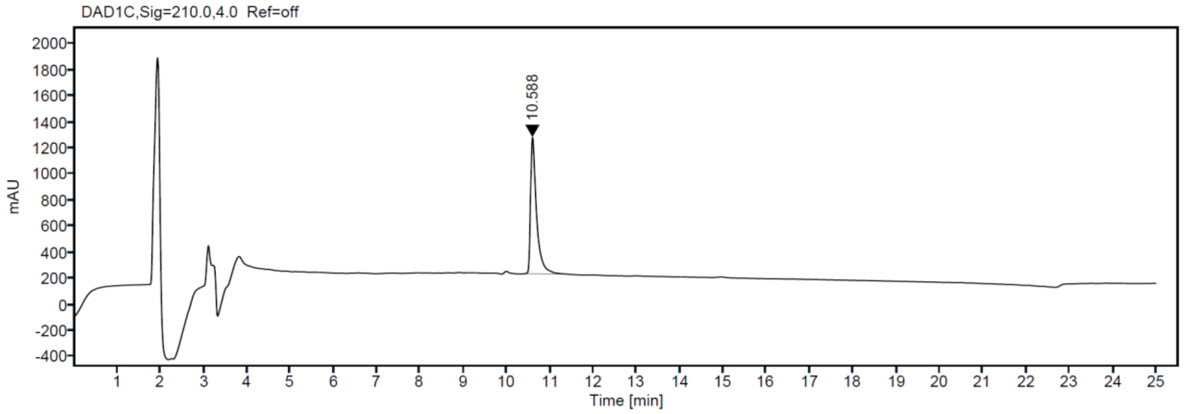

Retention time: 10.635 min **Area Percent:** 100%

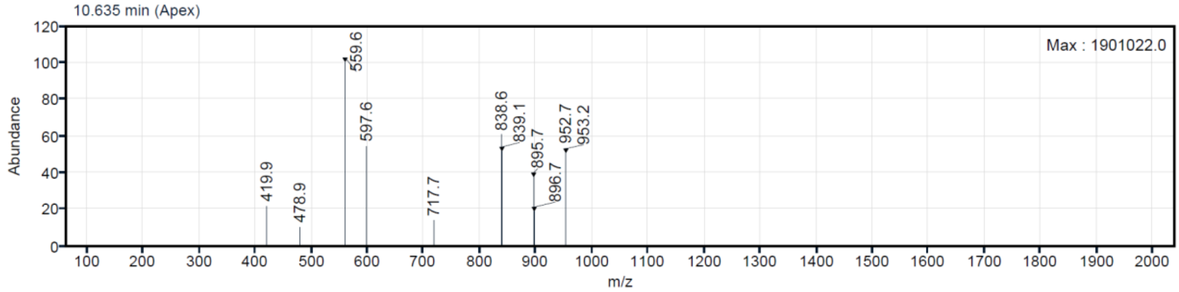

HRMS (nanochip-ESI/LTQ-Orbitrap) m/z: $[M + H_3]^{+3}$ Calcd for $C_{72}H_{92}F_6IN_{24}O_9{}^{+3}$ 559.2137; Found 559.2152.

MS/MS fragmentation of **1ad**:

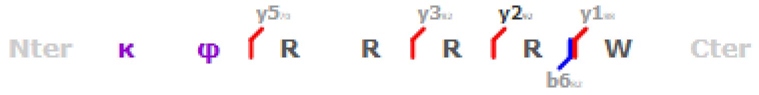

κ = Lys(N2H-2)
φ = Phe(C4H2)
Nter = C18H8F6IO2
Cter = NH2

Sequence	Type	MF	MF Mass	m/z	Intensity	Similarity
RW	y2	C17H26N7O2(+1)	360.2148	360.2142		91.98%
W	y1	C11H14N3O(+1)	204.1137	204.1131		87.52%
RRW	y3	C23H38N11O3(+1)	516.3159	516.3154		83.07%
KFRRRR	b6	C61H77F6IN21O8(+1)	1472.5213	736.764		82.45%
RRW	y3	C23H38N11O3(+1)	516.3159	258.6613		80.58%
KFRRRRW		C72H89F6IN24O9	1674.6193	838.3169		78.08%
RRRRW	y5	C35H62N19O5(+1)	828.5181	414.7624		70.00%

6. Condition screening for the Au(I)-catalyzed peptide-EBXs cyclization

General Procedure:

To a solution of peptide-EBXs **1** (0.01 mmol) in 1.87 mL HFIP in an open flask vial, 125 µL of freshly prepared AuCl·Me_2S (20 mM) solution in HFIP was added into the vial without light or atmosphere protection (Concentration: X mM). The reaction finished in 10 min with the formation of a yellow reaction mixture. The cyclic peptides were isolated by Prep-RP-HPLC, followed by lyophilization.

Common by-product:

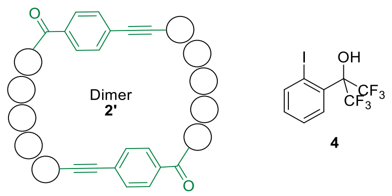

6.1 Calibration of the cyclization reaction

Conc. (mM)	Absorbance (mAU)
0.1	415.93
0.2	646.43
0.5	1706.82
1	2721.75

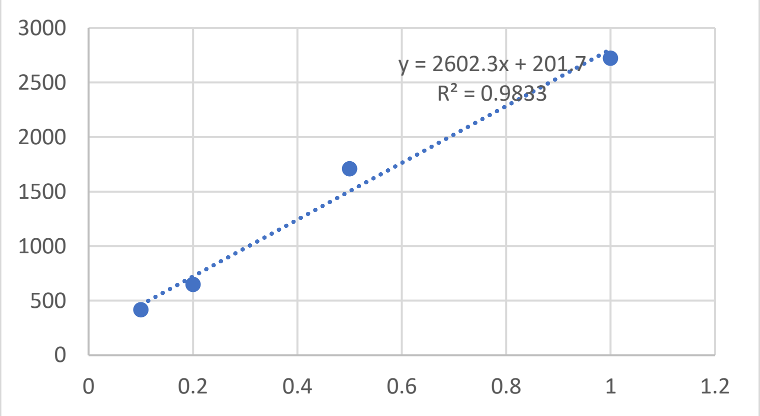

Figure S2: Linear equation of the absorbance (mAU) versus concentration (mM) of the cyclic peptide (2a)

6.2 Reaction optimization:

Table S1: Reaction optimization

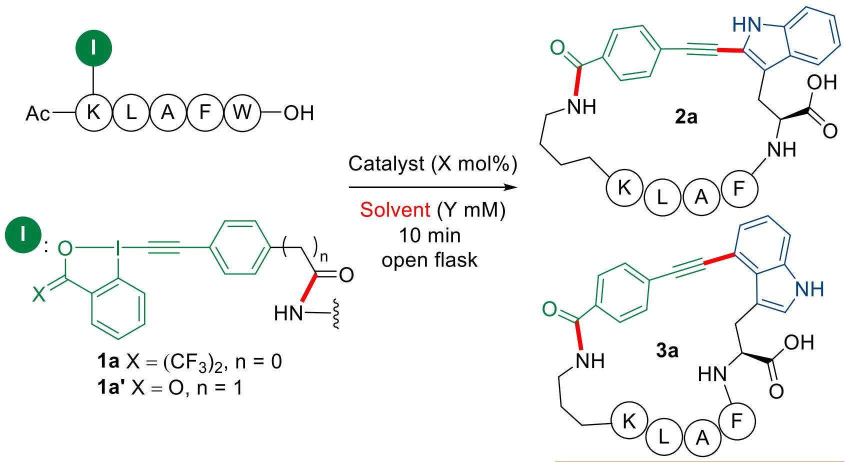

Entry	Catalyst	C(mM)	Solvent	P%
1	AuCl·Me_2S (100 mol%)	5	DMF	0
2	AuCl·Me_2S (100 mol%)	5	DMSO	0
3	AuCl·Me_2S (100 mol%)	5	MeCN (2% TFA)	64(99:1)
4	AuCl·Me_2S (100 mol%)	5	MeOH	69(95:5)
5	AuCl·Me_2S (100 mol%)	5	Dioxane	42(98:2)
6	AuCl·Me_2S (100 mol%)	5	TFE	84(99:1)
7	AuCl·Me_2S (100 mol%)	5	HFIP	92(97:3)
8	AuCl·Me_2S (50 mol%)	5	HFIP	91(97:3)
9	AuCl·Me_2S (25 mol%)	5	HFIP	93(97:3)
10	AuCl·Me_2S (10 mol%)	5	HFIP	90 (81%)[a] (56%)[b](97:3)
11	AuCl·Me_2S (10 mol%)	10	HFIP	66(97:3)
12	AuCl·Me_2S (10 mol%)	2.5	HFIP	64(97:3)
13	$AgBF_4$ (100 mol%)	5	HFIP	0
14	$Pd(MeCN)_4(BF_4)_2$ (100 mol%)	5	HFIP	0
15	AuCl (100 mol%)	5	HFIP	88(97:3)
16[c]	AuCl·Me_2S (100 mol%)	5	HFIP	20(94:6)
17	-	5	HFIP	0

Condition screening was conducted on 1 μmol scale of **1a**. The yield was determined based on the HPLC-UV ratio of desired product peak area/total peptide related peaks. The ratio of C2/C4 regioisomers (**2a**:**3a**) is provided in parenthesis. [a]The yield was calculated based on the calibration curve (y = 2602.3x + 201.7). [b]Isolated yield. [c]Benzoiodoxolone reagent **1a'** was used instead of bis-CF_3 reagent **1a**.

6.3 Scope of cyclization

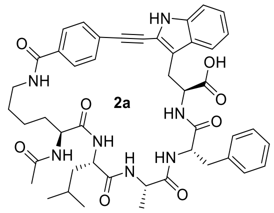

Following the general procedure, the reaction was conducted in 0.01 mmol scale. The desired product **2a** (4.7 mg, 5.6 µmol, 56% yield) was isolated by **Method 2**.

HPLC-UV chromatogram (210 nm) of the crude reaction mixture

HPLC-UV ratio of **2a**:**3a**: 97:3

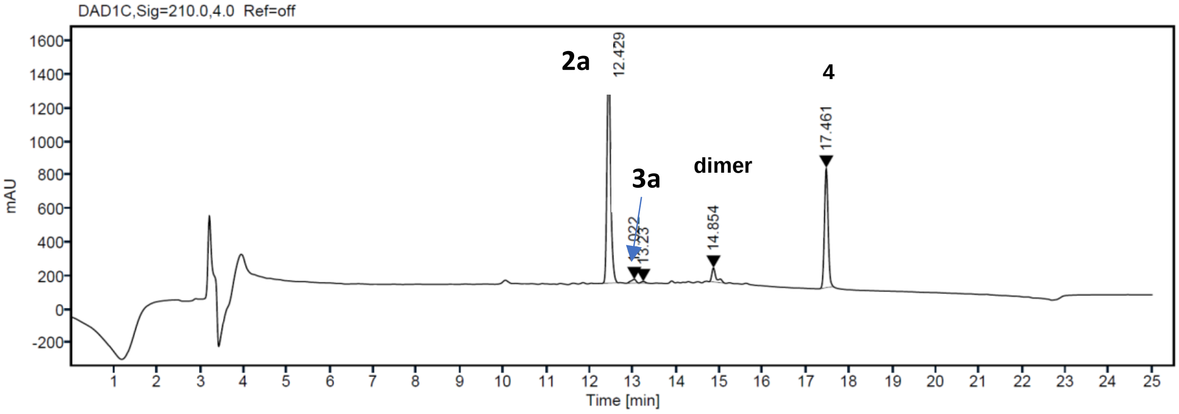

HPLC-UV chromatogram (210 nm) of **2a**

HPLC-UV ratio of P:P': Not determined due to the overlap.

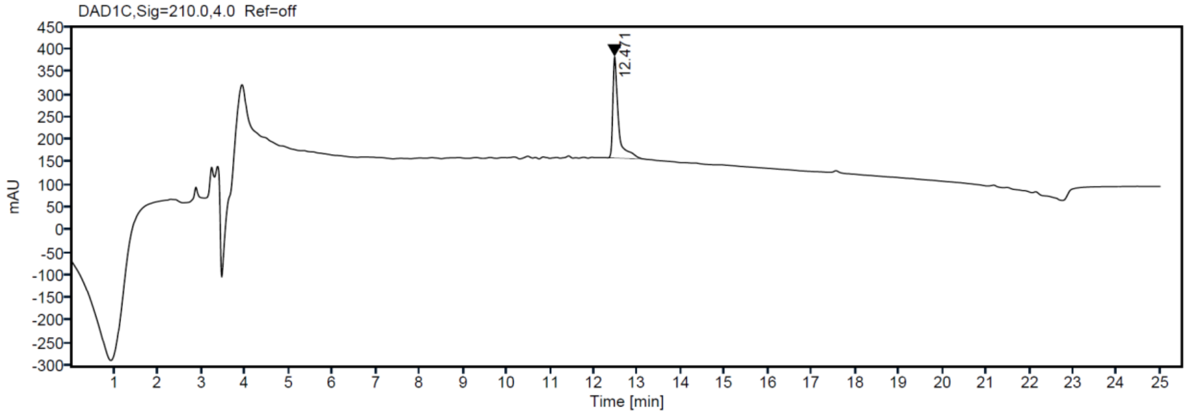

Retention time: 12.498 min **Area Percent:** 100%

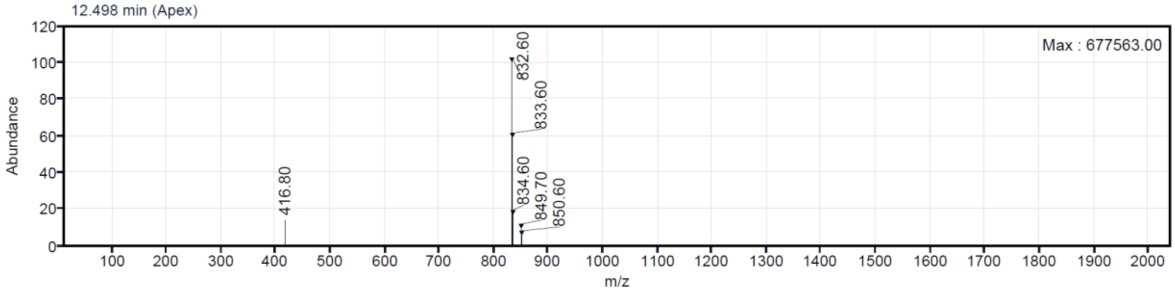

^{1}H NMR (500 MHz, DMSO) δ 11.59 (s, 1H, NH(Trp)), 8.46 (d, J = 8.1 Hz, 1H, NH), 8.26 (d, J = 8.2 Hz, 1H, NH), 8.17 (t, J = 5.4 Hz, 1H, NH), 8.11 (d, J = 7.1 Hz, 1H, NH), 7.92 (d, J =

7.7 Hz, 1H, NH), 7.85 (d, J = 8.2 Hz, 2H, ArH(phenylacetylene)), 7.53 (d, J = 8.0 Hz, 1H, NH), 7.49 (d, J = 8.1 Hz, 2H, ArH(phenylacetylene)), 7.30 (d, J = 8.2 Hz, 1H, ArH(Trp)), 7.20 – 7.11 (m, 5H, ArH(Phe)), 7.08 (m, 2H, ArH(Trp) NH overlapping), 7.01 (dd, J = 13.9, 6.4 Hz, 1H, ArH(Trp)), 6.93 (d, J = 7.6 Hz, 1H, NH), 4.81 – 4.72 (m, 1H), 4.64 – 4.57 (m, 1H), 4.35 – 4.28 (m, 1H), 4.24 – 4.16 (m, 1H), 3.79 (p, J = 7.4 Hz, 1H), 2.93 (dd, J = 13.8, 4.6 Hz, 1H), 2.83 (dd, J = 13.9, 6.7 Hz, 1H), 1.80 (s, 3H), 1.64 – 1.53 (m, 4H), 1.50 – 1.42 (m, 4H), 1.38 – 1.28 (m, 5H), 0.88 (d, J = 7.0 Hz, 3H, CH_3(Ala)), 0.84 (d, J = 6.6 Hz, 3H, CH_3(Leu)), 0.79 (d, J = 6.5 Hz, 3H, CH_3(Leu)).
^{13}C NMR (126 MHz, DMSO) δ 173.1, 172.5, 171.5, 170.6, 169.7, 168.6, 165.9, 136.9, 136.2, 134.2, 130.5, 129.5, 127.9, 127.8, 126.8, 126.2, 124.6, 123.1, 119.4, 119.2, 118.0, 116.6, 111.3, 94.1, 83.8, 52.7, 52.0, 51.6, 51.2, 47.8, 32.7, 29.0, 28.4, 27.5, 24.1, 23.0, 22.5, 21.7, 21.4, 17.4.
HRMS (nanochip-ESI/LTQ-Orbitrap) m/z: $[M + H]^+$ Calcd for $C_{46}H_{54}N_7O_8^+$ 832.4028; Found 832.4024.

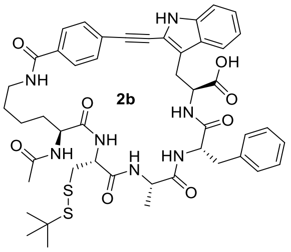

Following the general procedure, the reaction was conducted in 0.008 mmol scale. The desired product **2b** (1.8 mg, 2.0 µmol, 25% yield) was isolated by **Method 2**.
HPLC-UV chromatogram (210 nm) of the crude reaction mixture
HPLC-UV ratio of **2b**:**3b**: 97:3

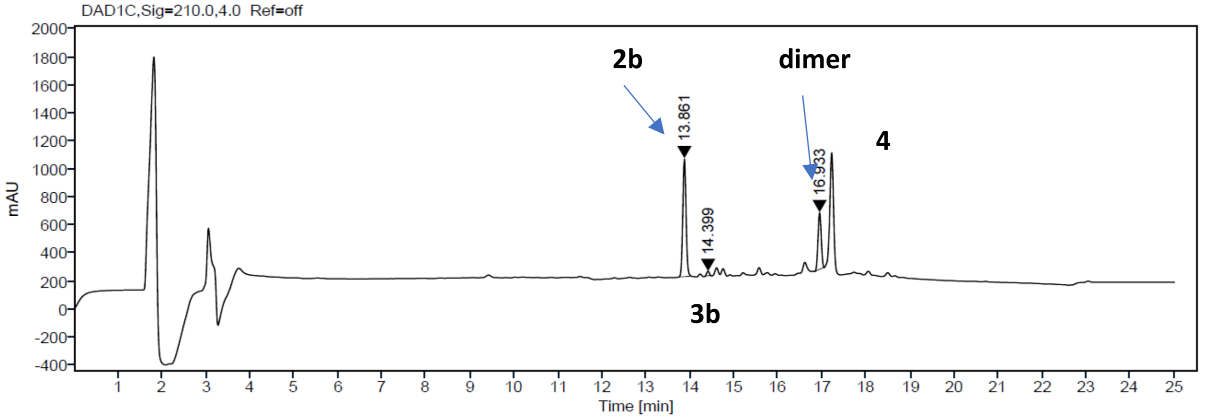

HPLC-UV chromatogram (210 nm) of **2b:** Only single isomer was isolated

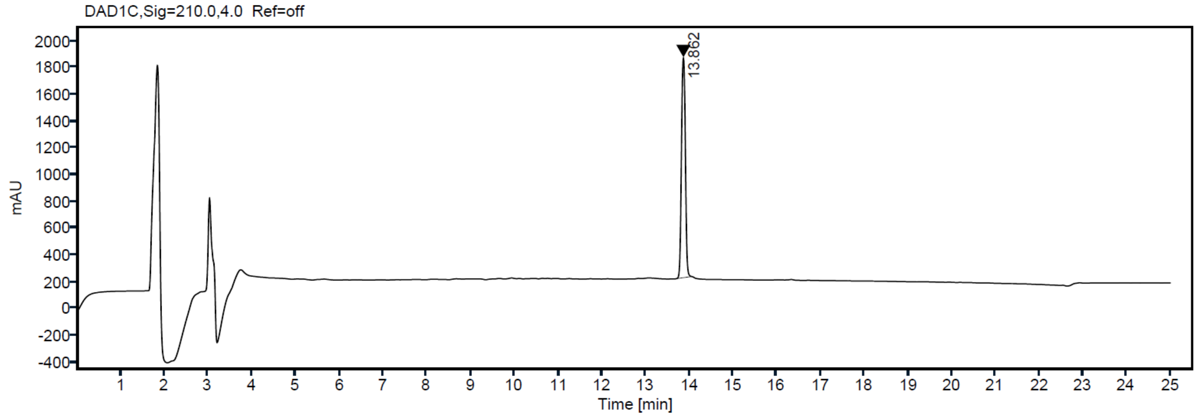

Retention time: 13.89 min Area Percent: 100%

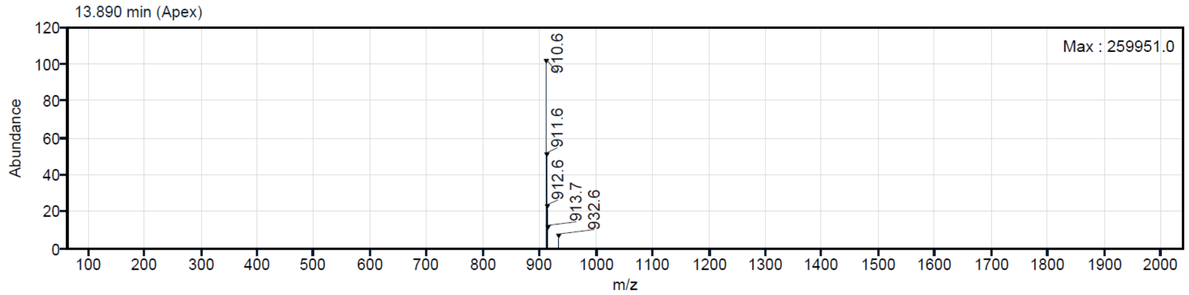

HRMS (ESI/QTOF) m/z: [M + Na]$^+$ Calcd for $C_{47}H_{55}N_7NaO_8S_2^+$ 932.3446; Found 932.3428.

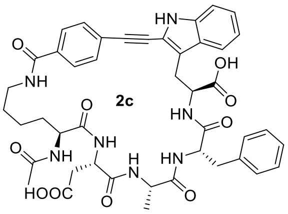

Following the general procedure, the reaction was conducted in 0.01 mmol scale. The desired product **2c** (4.3 mg, 5.1 µmol, 51% yield) was isolated by **Method 2**.

HPLC-UV chromatogram (210 nm) of the crude reaction mixture

HPLC-UV ratio of **2c**:**3c**: 96:4

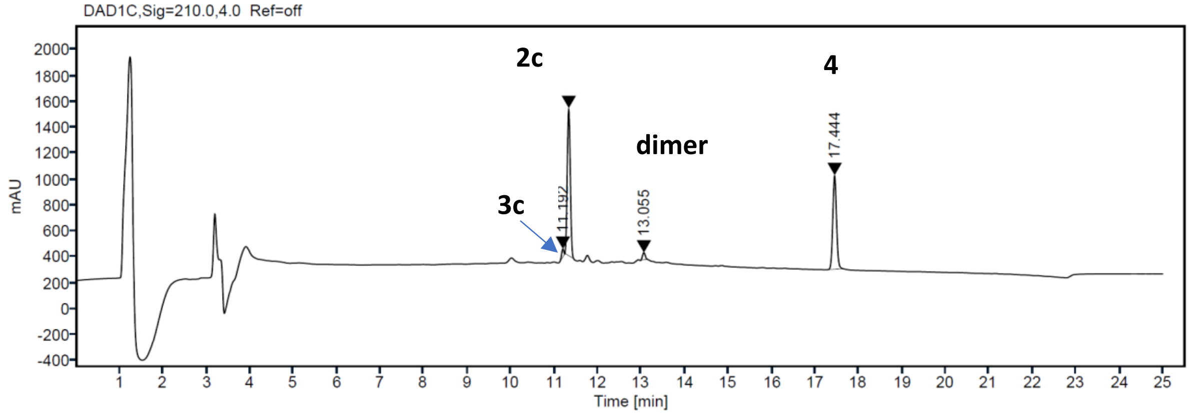

HPLC-UV chromatogram (210 nm) of **2c**

HPLC-UV ratio of **2c**:**3c** after isolation: 90:10 (The ratio is not precise due to the overlap)

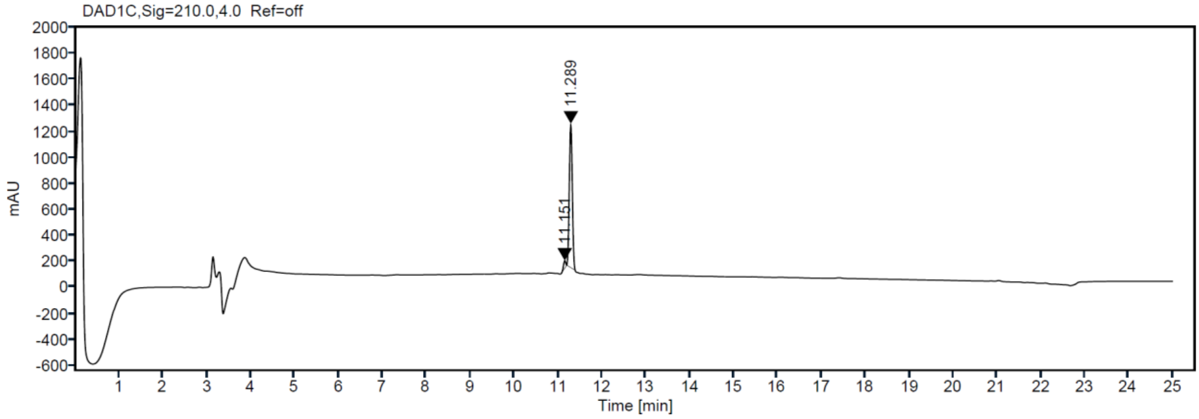

Retention time: 11.181 min **Area Percent:** 13%

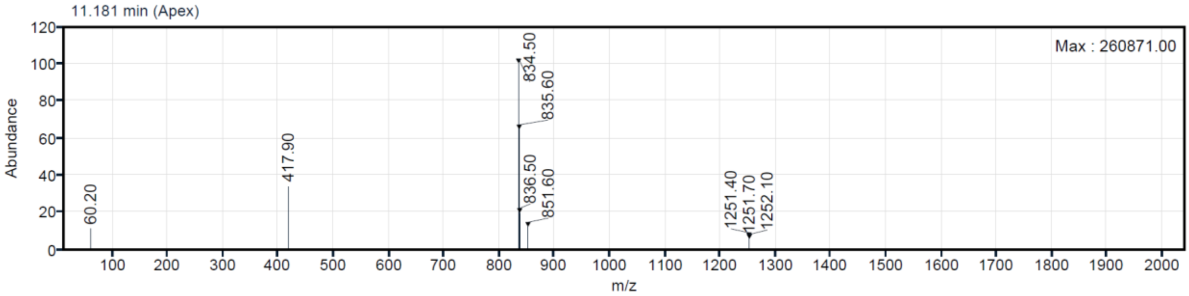

Retention time: 11.318 min **Area Percent:** 87%

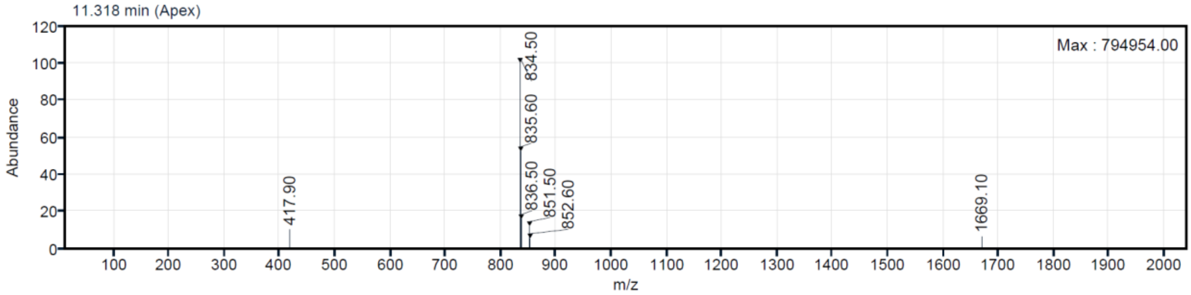

HRMS (Nanochip-based ESI/LTQ-Orbitrap) m/z: $[M + H]^+$ Calcd for $C_{44}H_{48}N_7O_{10}^+$ 834.3457; Found 834.3459.

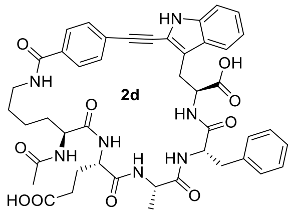

Following the general procedure, the reaction was conducted in 0.01 mmol scale. The desired product **2d** (3.4 mg, 3.7 µmol, 37% yield) was isolated by **Method 2**.
HPLC-UV chromatogram (210 nm) of the crude reaction mixture
HPLC-UV ratio of **2d:3d**: Not determined due to the overlap.

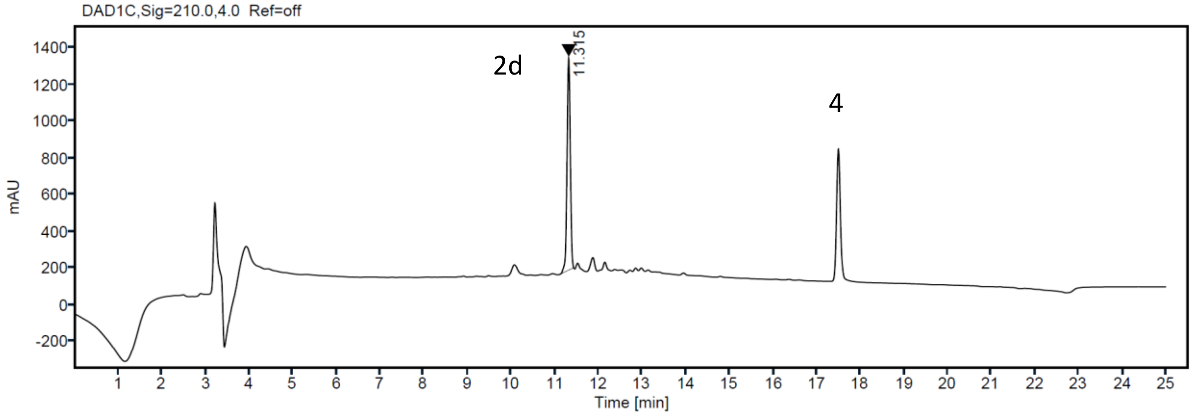

HPLC-UV chromatogram (210 nm) of **2d**

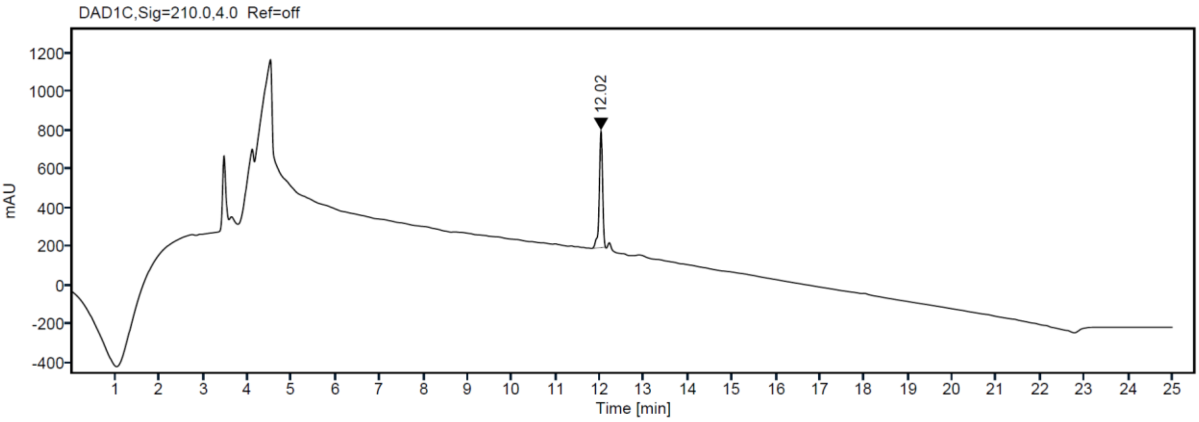

Retention time: 12.037 min **Area Percent:** 100%

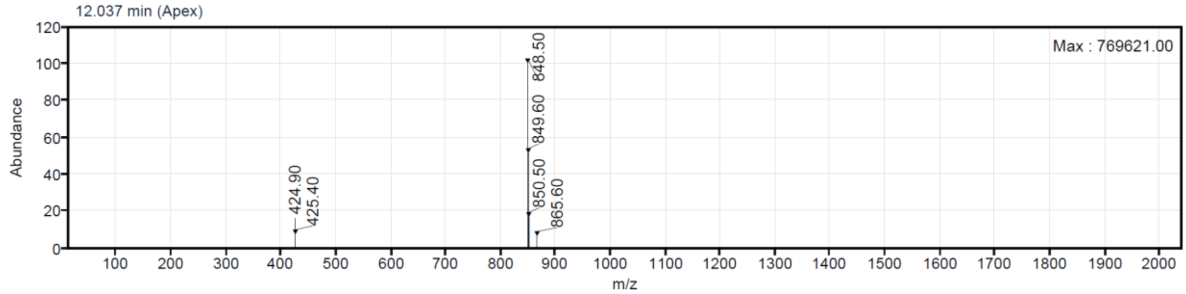

HRMS (Nanochip-based ESI/LTQ-Orbitrap) m/z: $[M + H]^+$ Calcd for $C_{45}H_{50}N_7O_{10}^+$ 848.3614; Found 848.3627.

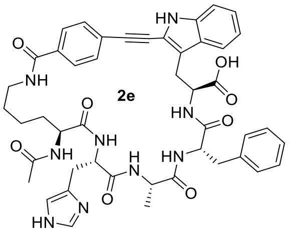

Following the general procedure, the reaction was conducted in 0.01 mmol scale. The desired product **2e** (2.6 mg, 3.0 µmol, 30% yield) was isolated by **Method 2**.
HPLC-UV chromatogram (210 nm) of the crude reaction mixture
HPLC-UV ratio of **2e**:**3e**: 87:12.

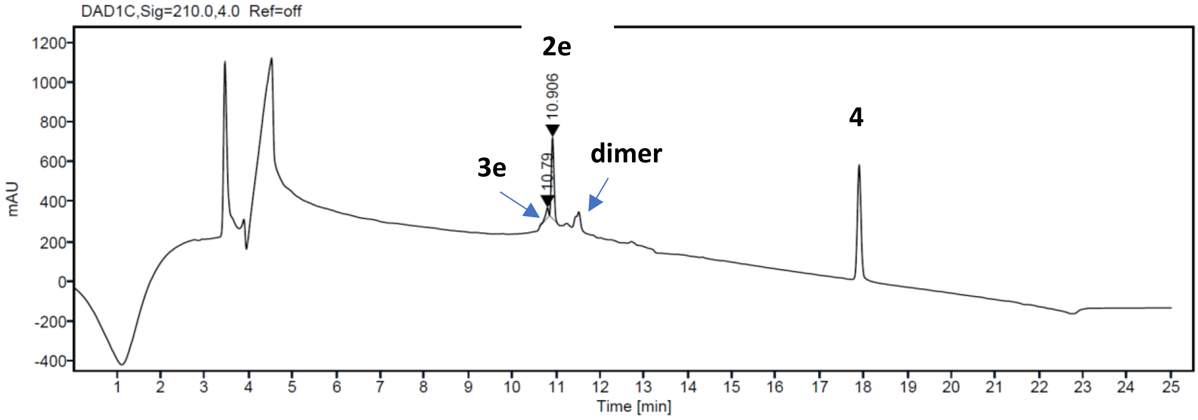

HPLC-UV chromatogram (210 nm) of **2e**:
HPLC-UV ratio of **2e**:**3e** after isolation: 90:10 (The ratio is not precise due to the overlap)

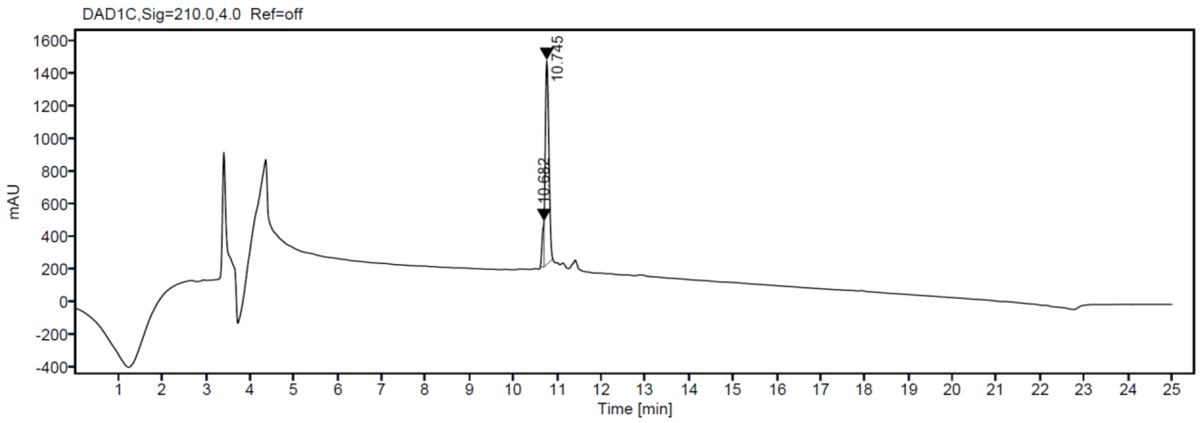

Retention time: 10.68 min **Area Percent:** 9%

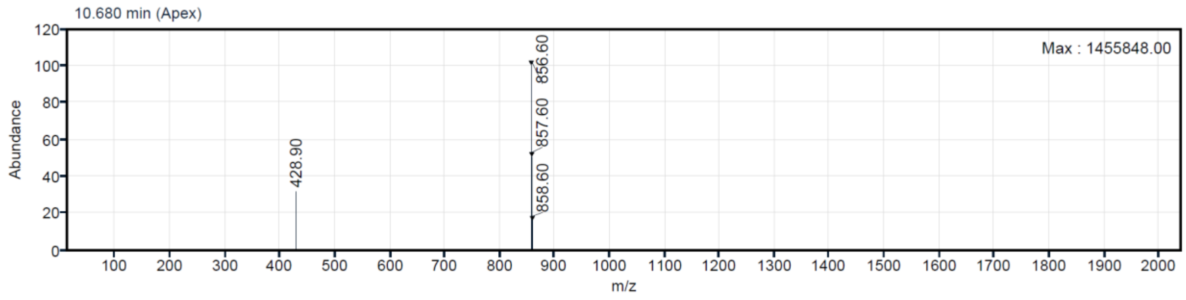

Retention time: 10.77 min **Area Percent:** 91%

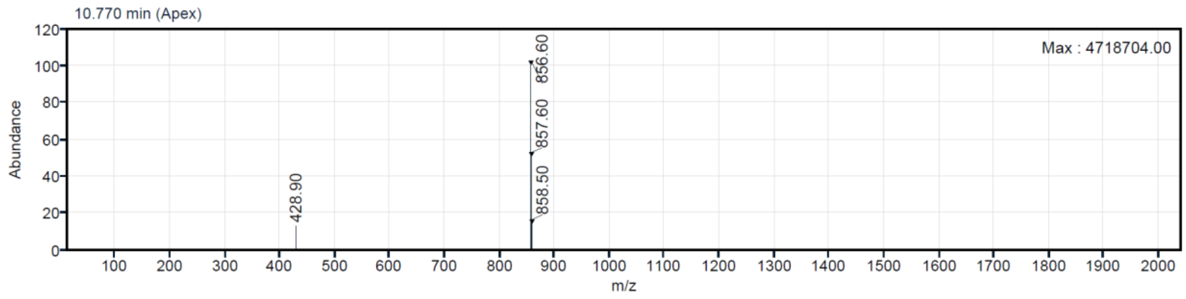

HRMS (nanochip-ESI/LTQ-Orbitrap) m/z: $[M + H]^+$ Calcd for $C_{46}H_{50}N_9O_8^+$ 856.3777; Found 856.3763.

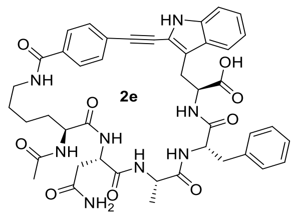

Following the general procedure, the reaction was conducted in 0.01 mmol scale. The desired product **2f** (4.3 mg, 5.2 µmol, 52% yield) was isolated by **Method 2**.
HPLC-UV chromatogram (210 nm) of the crude reaction mixture
HPLC-UV ratio of **2f**:**3f**: 92:7 (The ratio is not precise due to the overlap)

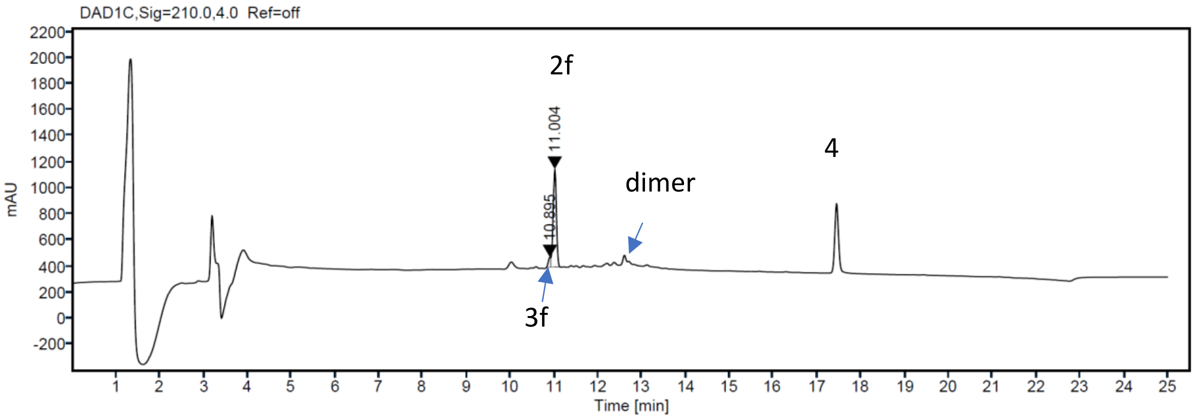

HPLC-UV chromatogram (210 nm) of **2f**:

HPLC-UV ratio of **2f**:**3f** after isolation: 90:10 (The ratio is not precise due to the overlap)

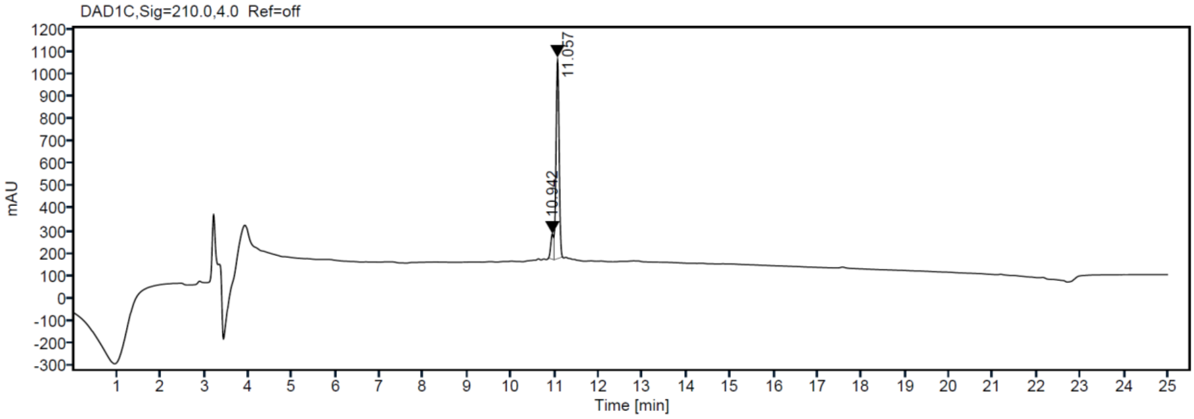

Retention time: 10.974 min **Area Percent:** 23%

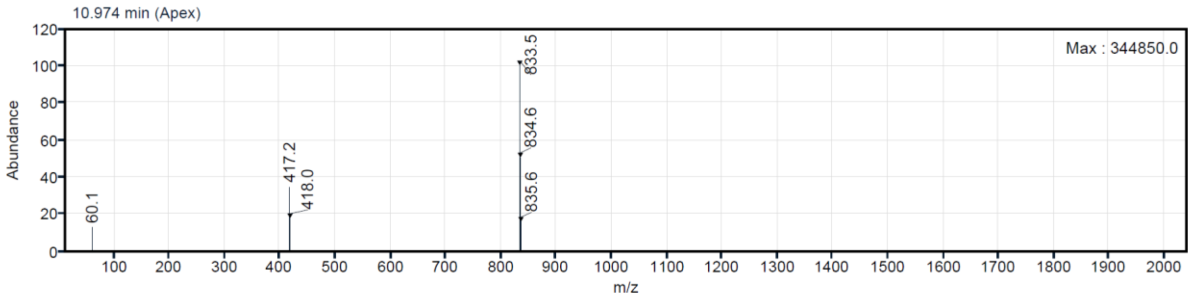

Retention time: 11.091 min **Area Percent:** 77%

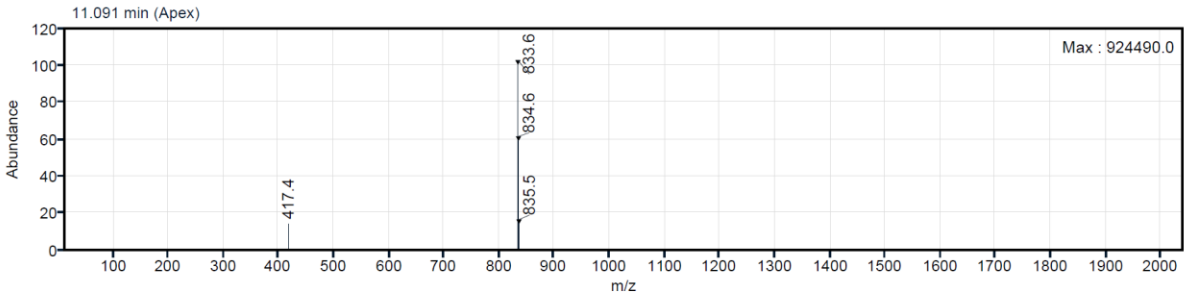

HRMS (nanochip-ESI/LTQ-Orbitrap) m/z: $[M + H]^+$ Calcd for $C_{44}H_{49}N_8O_9^+$ 833.3617; Found 833.3620.

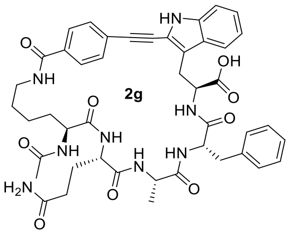

Following the general procedure, the reaction was conducted in 0.01 mmol scale. The desired product **2g** (4.7 mg, 5.6 µmol, 56% yield) was isolated by **Method 2**.

HPLC-UV chromatogram (210 nm) of the crude reaction mixture

HPLC-UV ratio of **2g**:**3g**: 97:3. (The ratio is not precise due to the overlap)

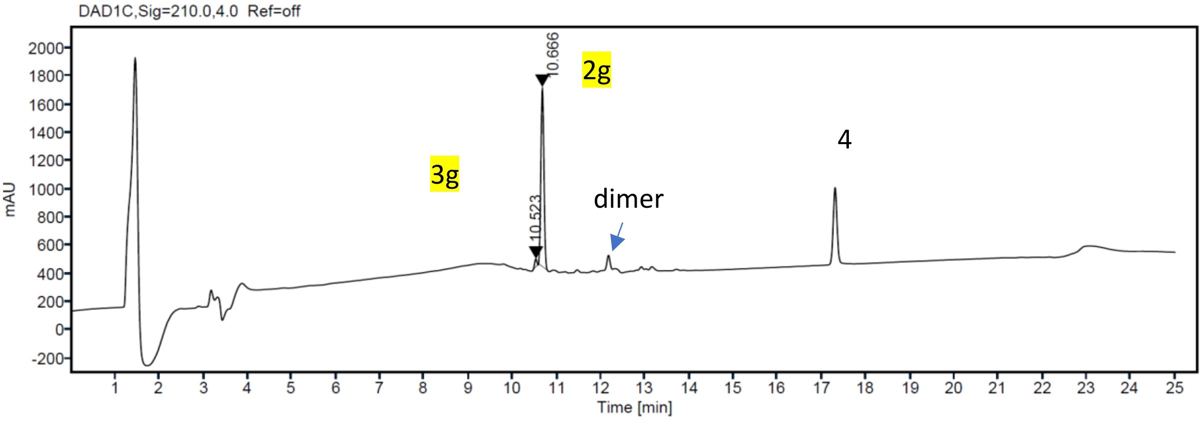

HPLC-UV chromatogram (210 nm) of **2g**:

HPLC-UV ratio of **2g**:**3g** after isolation: 96:4

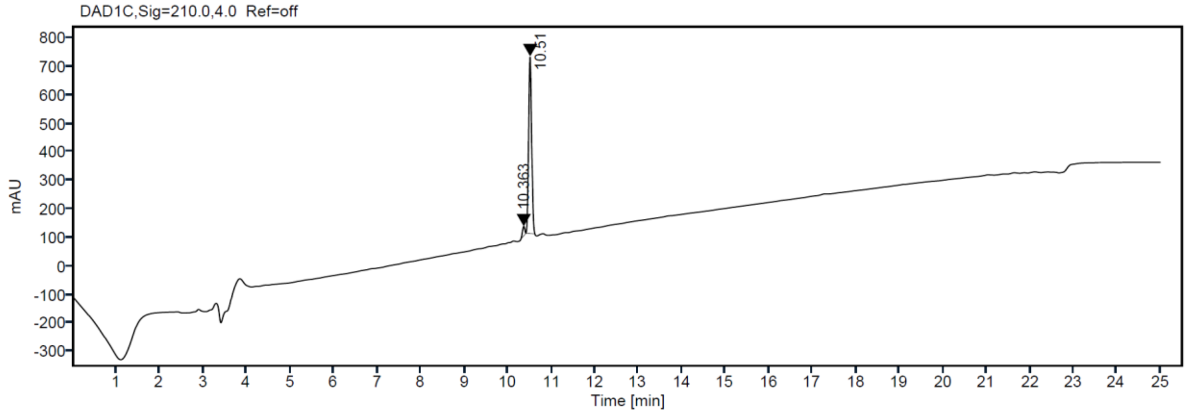

Retention time: 10.392 min **Area Percent:** 7%

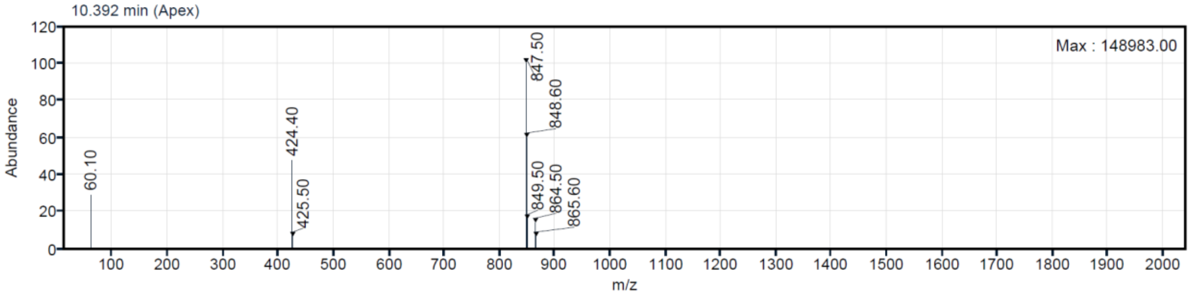

Retention time: 10.547 min **Area Percent:** 93%

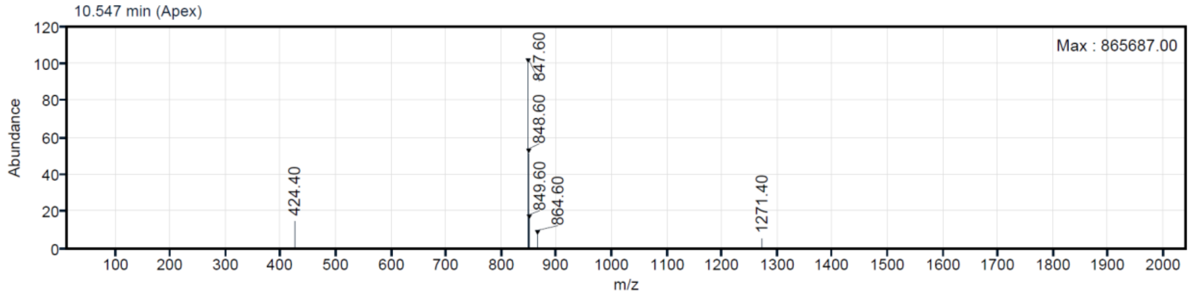

HRMS (nanochip-ESI/LTQ-Orbitrap) m/z: $[M + H]^+$ Calcd for $C_{45}H_{51}N_8O_9^+$ 847.3774; Found 847.3775.

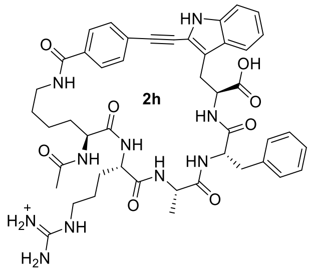

Following the general procedure, the reaction was conducted in 0.01 mmol scale. The desired product **2h** (3.6 mg, 4.2 µmol, 42% yield) was isolated by **Method 2**.
HPLC-UV chromatogram (210 nm) of the crude reaction mixture
HPLC-UV ratio of **2h**:**3h**: 97:3.

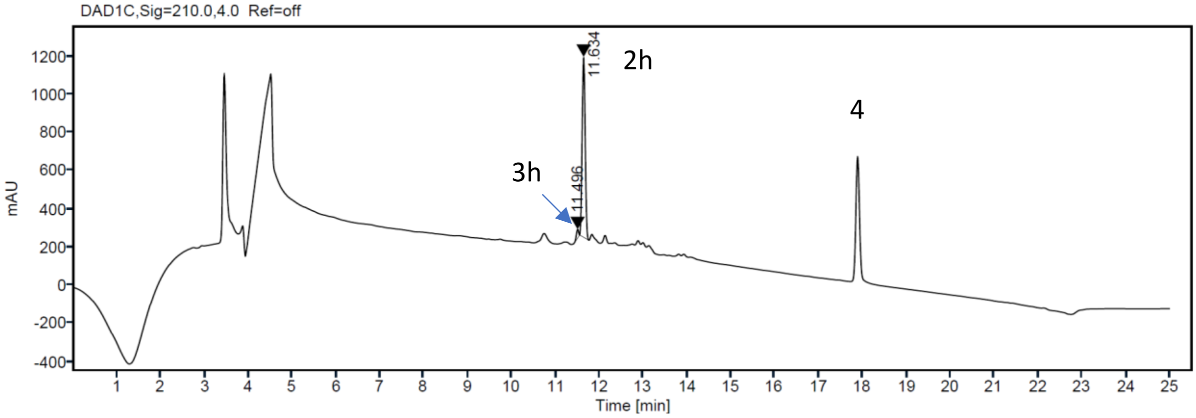

HPLC-UV chromatogram (210 nm) of **2h**:
HPLC-UV ratio of **2h**:**3h** after isolation: 90:10

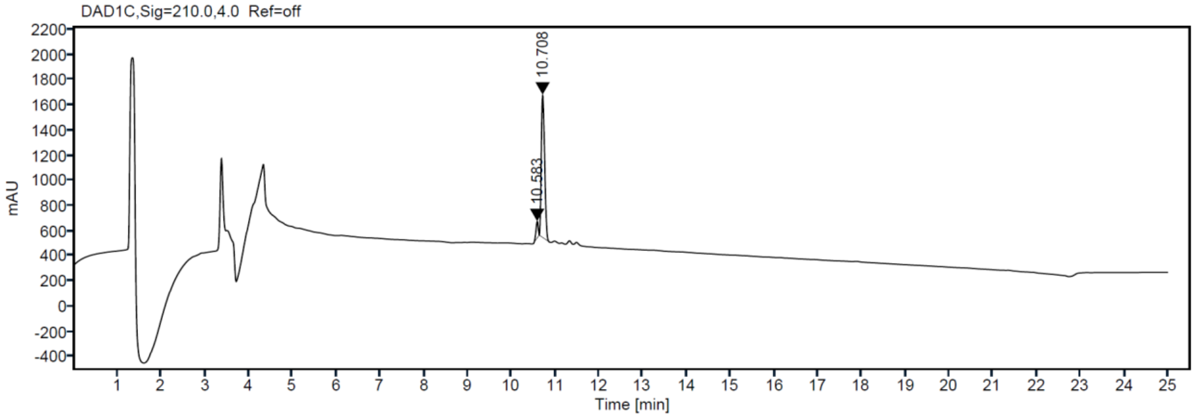

Retention time: 10.612 min **Area Percent:** 11%

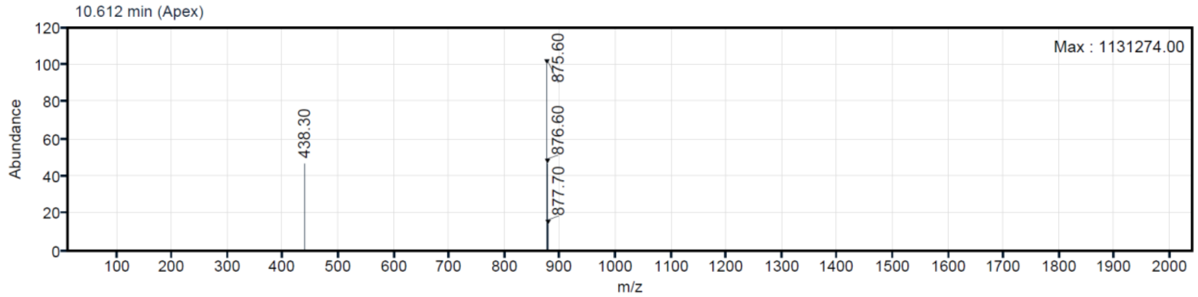

Retention time: 10.742 min **Area Percent:** 89%

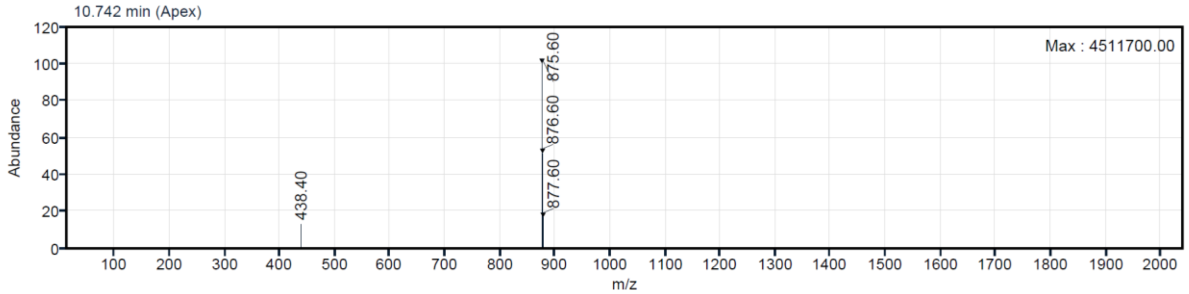

HRMS (nanochip-ESI/LTQ-Orbitrap) m/z: $[M + H]^+$ Calcd for $C_{46}H_{55}N_{10}O_8^+$ 875.4199; Found 875.4191.

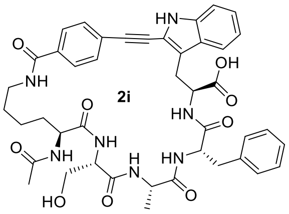

Following the general procedure, the reaction was conducted in 0.01 mmol scale. The desired product **2i** (5.4 mg, 6.7 µmol, 67% yield) was isolated by **Method 2**.

HPLC-UV ratio of **2i**:**3i**: Not determined due to the overlap

HPLC-UV chromatogram (210 nm) of the crude reaction mixture

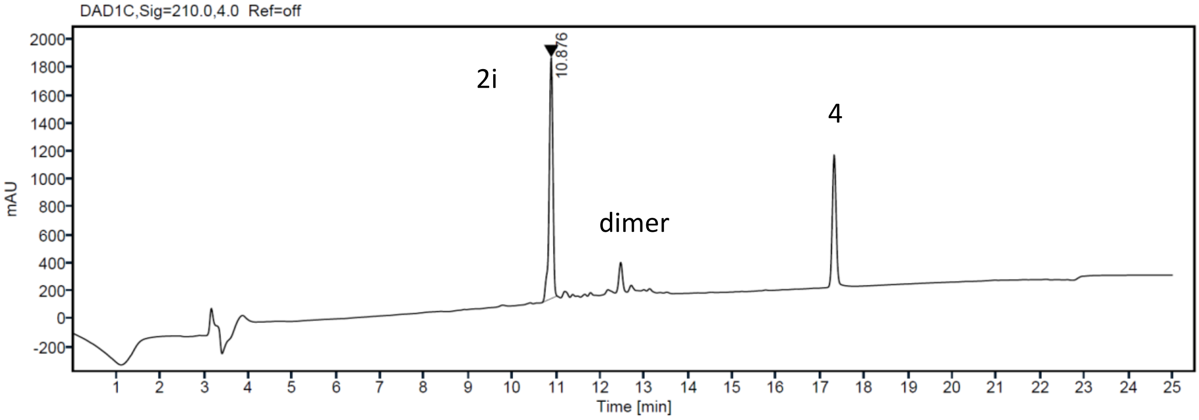

HPLC-UV chromatogram (210 nm) of **2i**:

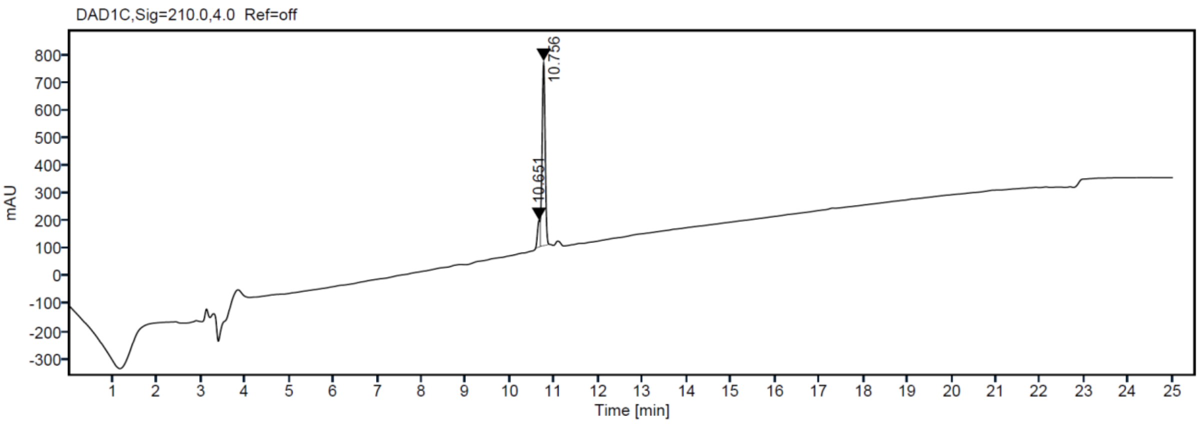

Retention time: 10.786 min **Area Percent:** 100%

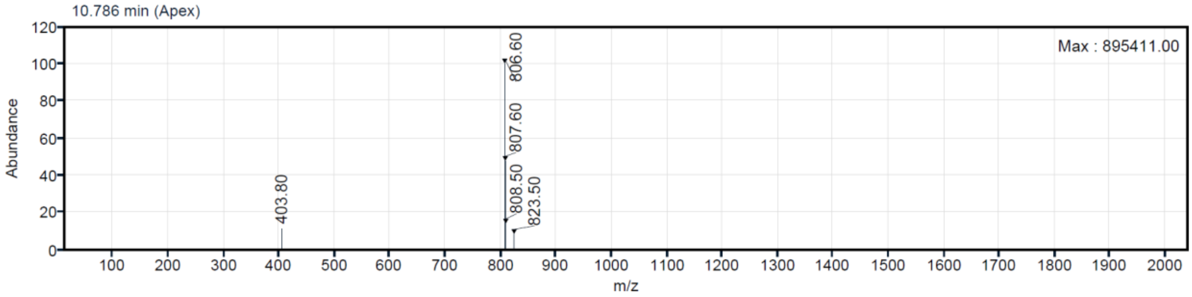

HRMS (nanochip-ESI/LTQ-Orbitrap) m/z: $[M + H]^+$ Calcd for $C_{43}H_{48}N_7O_9^+$ 806.3508; Found 806.3505.

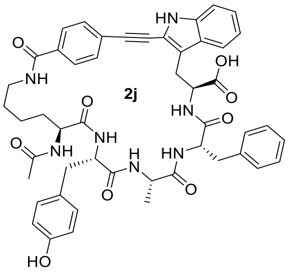

Following the general procedure, the reaction was conducted in 0.01 mmol scale. The desired product **2j** (4.4 mg, 5.3 µmol, 53% yield) was isolated by **Method 2**.
HPLC-UV chromatogram (210 nm) of the crude reaction mixture
HPLC-UV ratio of **2j**:**3j**: 98:2.

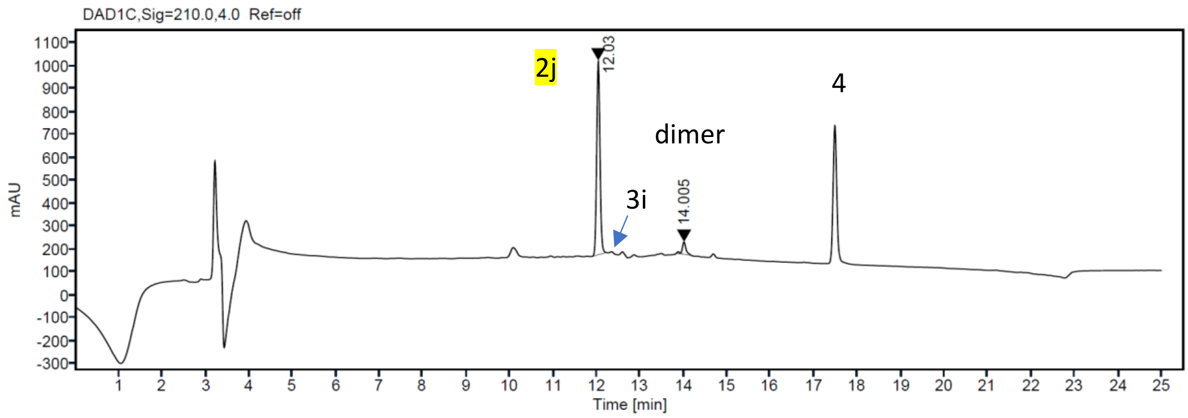

HPLC-UV chromatogram (210 nm) of **2j**:

HPLC-UV ratio of **2j**:**3j** after isolation: 98:2

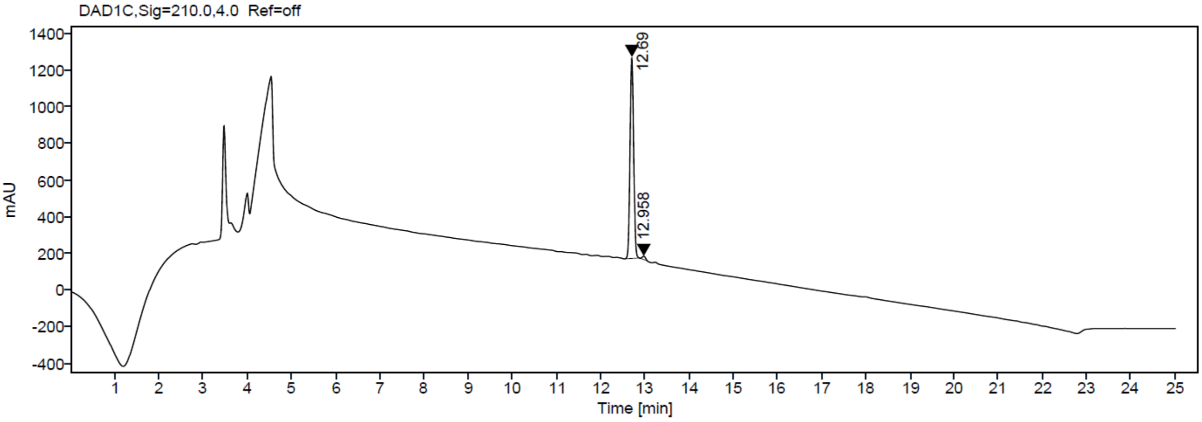

Retention time: 12.721 min **Area Percent:** 94%

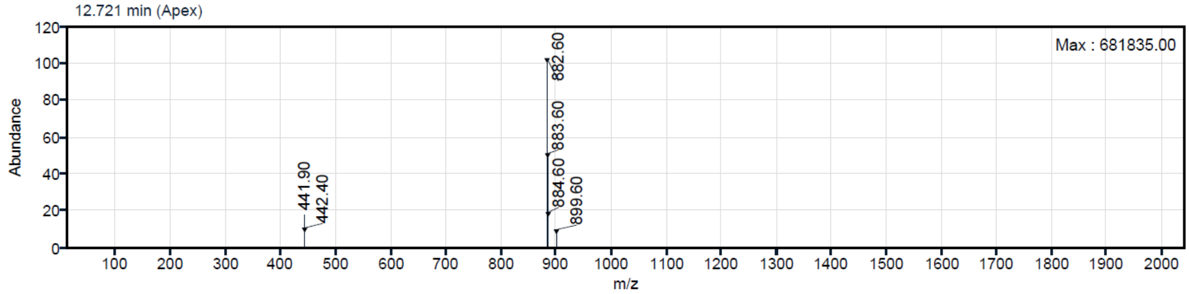

Retention time: 12.99 min **Area Percent:** 6%

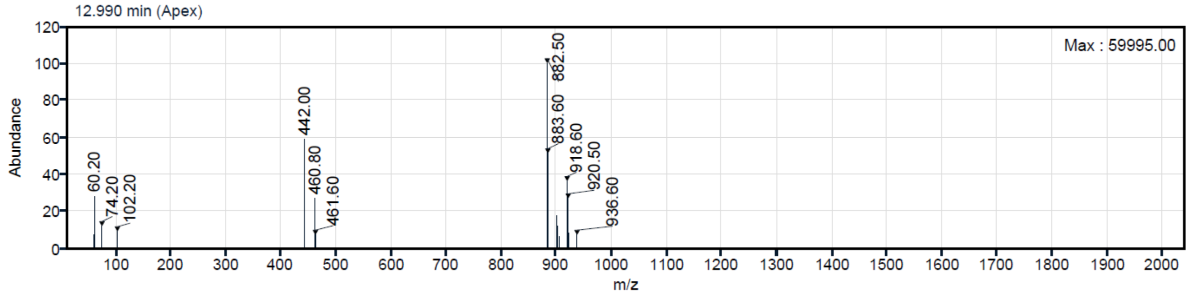

HRMS (Nanochip-based ESI/LTQ-Orbitrap) m/z: $[M + H]^+$ Calcd for $C_{49}H_{52}N_7O_9^+$ 882.3821; Found 882.3823.

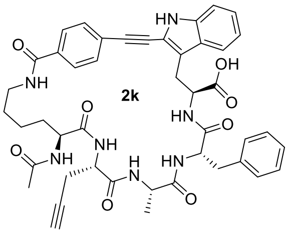

Following the general procedure, the reaction was conducted in 0.01 mmol scale. The desired product **2k** (4.8 mg, 5.7 µmol, 57% yield) was isolated by **Method 2**.
HPLC-UV ratio of **2k**:**3k** Not determined due to the overlap, Ratio of **2k**:**3k** was determined by the ^{1}H NMR of isolated product: 93:7 using the integration of Trp NH.
HPLC-UV chromatogram (210 nm) of the crude reaction mixture

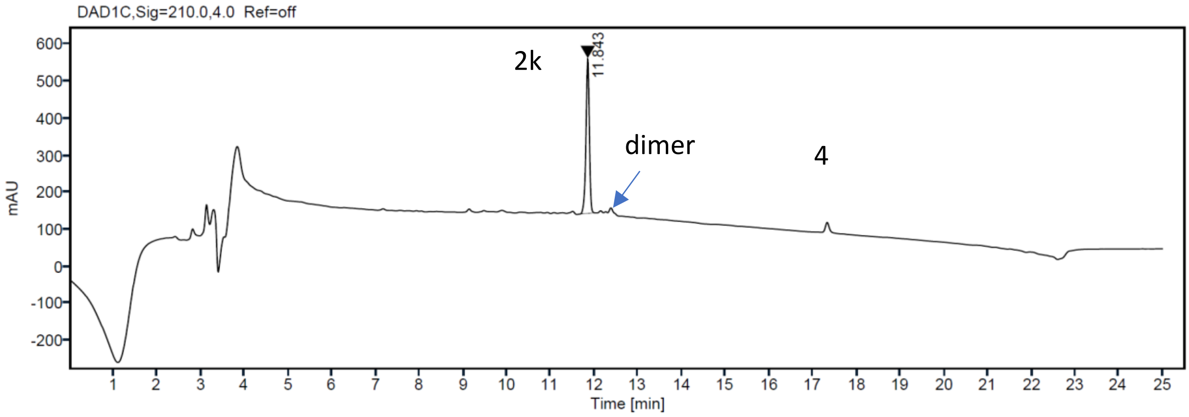

HPLC-UV chromatogram (210 nm) of **2k**:

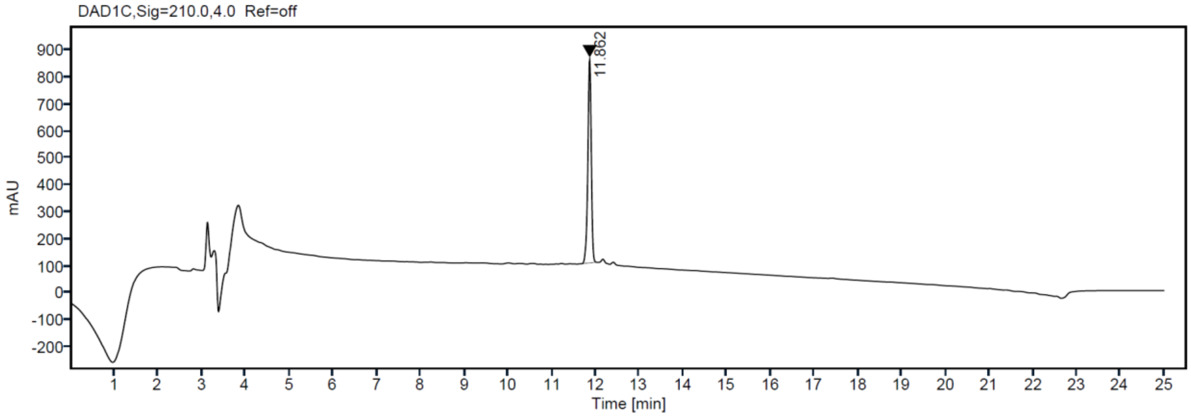

Retention time: 11.904 min **Area Percent:** 100%

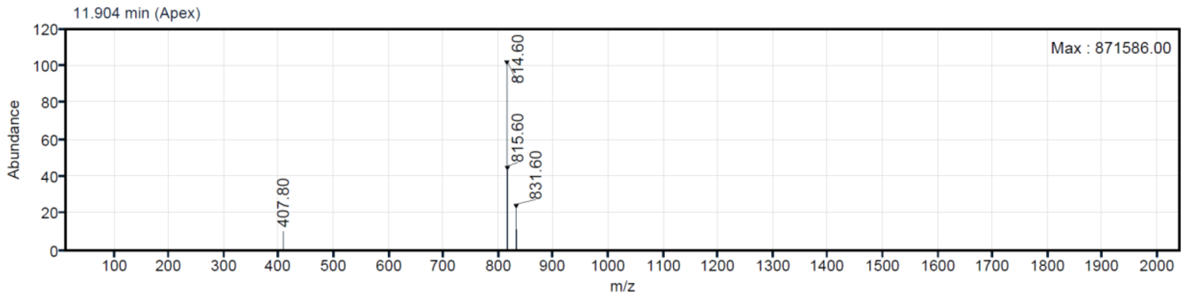

^{1}H NMR (600 MHz, DMSO) δ 11.62 (s, 1H, NH(Trp)), 8.53 (d, *J* = 8.1 Hz, 1H, NH), 8.45 (d, *J* = 7.1 Hz, 1H, NH), 8.30 (d, *J* = 8.2 Hz, 1H, NH), 8.16 (t, *J* = 5.3 Hz, 1H, NH), 8.01 (d, *J* = 8.0 Hz, 1H, NH), 7.86 (d, *J* = 8.0 Hz, 2H, ArH(Phenylacetylene)), 7.55 (d, *J* = 8.0 Hz, 1H, ArH(Trp)), 7.51 (d, *J* = 8.1 Hz, 2H, ArH(Phenylacetylene)), 7.32 (d, *J* = 8.2 Hz, 1H, ArH(Trp)), 7.22 – 7.15 (m, 5H, ArH(Phe)), 7.12 (d, *J* = 7.3 Hz, 2H, ArH(Trp), NH overlapping), 7.10 (d, *J* = 7.9 Hz, 1H, NH), 7.06 – 7.00 (m, 1H, ArH(Trp)), 4.83 – 4.77 (m, 1H), 4.63 (td, *J* = 7.5, 4.8 Hz, 1H), 4.38 (q, *J* = 7.1 Hz, 1H), 4.32 (dt, *J* = 8.8, 6.1 Hz, 1H), 3.84 (p, *J* = 7.2 Hz, 1H), 2.97 (dd, *J* = 13.9, 4.6 Hz, 1H), 2.87 – 2.83 (m, 1H), 2.83 – 2.81 (m, 1H), 2.46 – 2.38 (m, 2H), 1.83 (s, 3H), 1.63 – 1.54 (m, 3H), 1.50 – 1.43 (m, 1H), 1.35 (p, *J* = 7.8 Hz, 2H), 0.90 (d, *J* = 7.0 Hz, 3H, CH_3(Ala)).

^{13}C NMR (151 MHz, DMSO) δ 173.1 171.7, 170.5, 170.2, 170.0, 168.7, 165.9, 137.0, 136.2, 134.3, 130.6, 129.6, 127.9, 127.8, 126.9, 126.3, 124.6, 123.2, 119.3, 119.1, 116.6, 111.3, 94.3, 83.8, 80.8, 73.2, 52.8, 52.2, 52.1, 51.6, 47.9, 38.0, 32.7, 28.3, 27.5, 22.5, 21.7, 21.1, 17.7.

HRMS (nanochip-ESI/LTQ-Orbitrap) m/z: $[M + H]^+$ Calcd for $C_{45}H_{48}N_7O_8^+$ 814.3559; Found 814.3564.

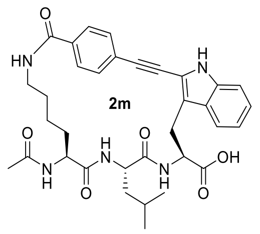

Following the general procedure, the reaction was conducted in 0.01 mmol scale. The desired product **2m** (1.8 mg, 2.9 µmol, 40% yield) was isolated by **Method 2**.

HPLC-UV chromatogram (210 nm) of the crude reaction mixture

HPLC-UV ratio of **2m**:**3m**: Not determined due to the overlap

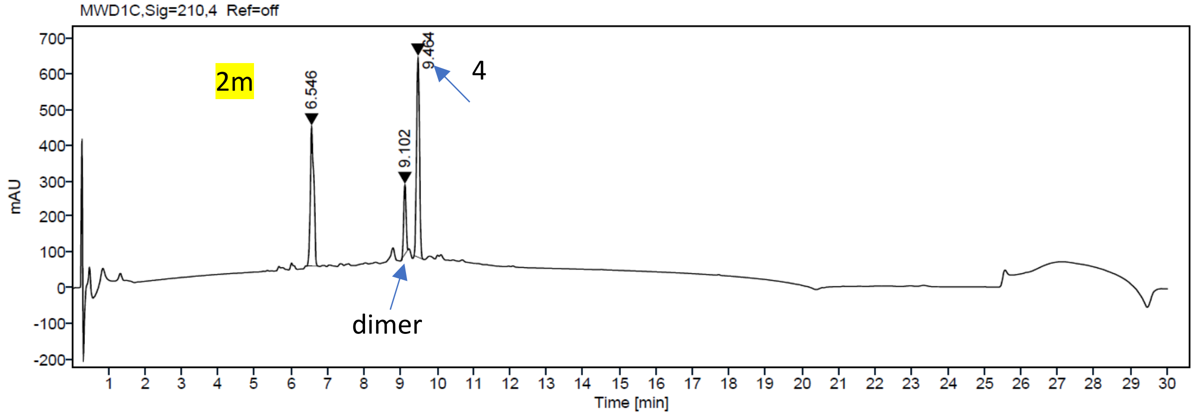

HPLC-UV chromatogram (210 nm) of **2m**:

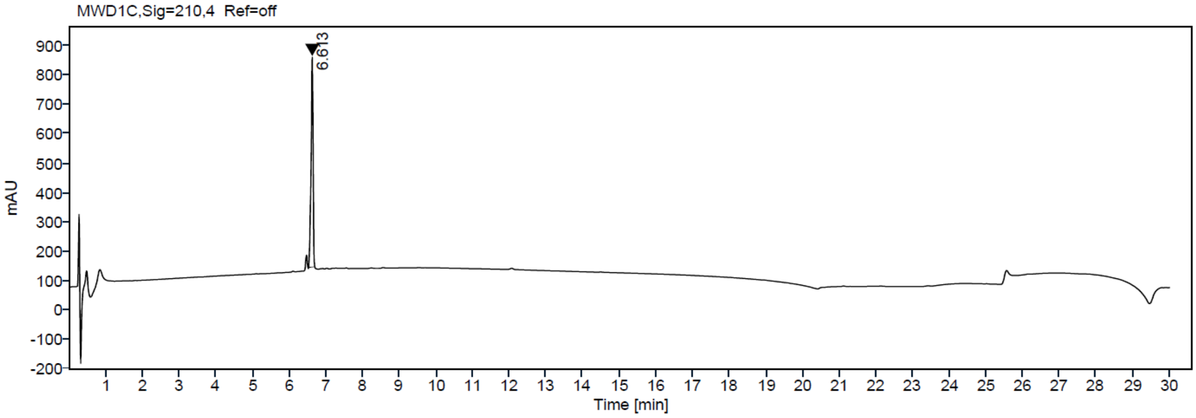

Retention time: 6.667 min **Area Percent:** 100%

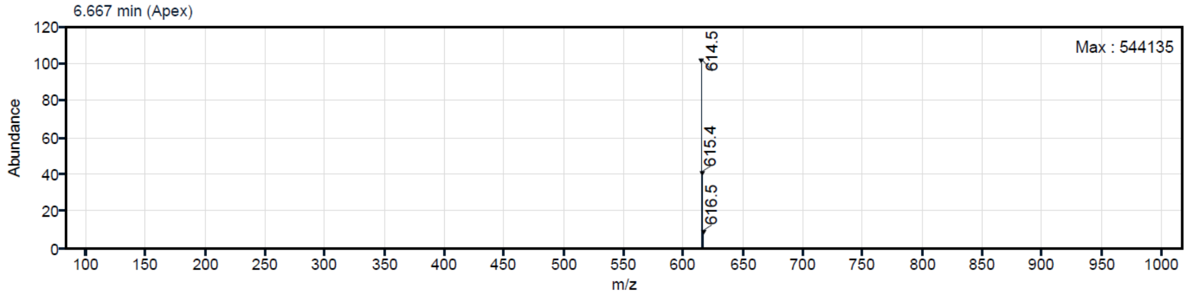

HRMS (ESI/QTOF) m/z: $[M + H]^+$ Calcd for $C_{34}H_{40}N_5O_6^+$ 614.2973; Found 614.2985.

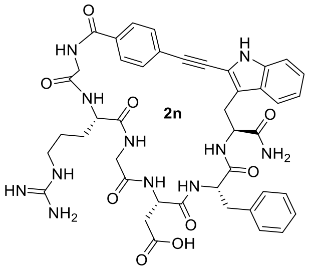

Following the general procedure, the reaction was conducted in 0.0083 mmol scale. The desired product **2n** (4.5 mg, 5.2 µmol, 63% yield) was isolated by **Method 2**.

HPLC-UV chromatogram (210 nm) of the crude reaction mixture

HPLC-UV ratio of **2n**:**3n**: 93:7. A single diastereomer was isolated.

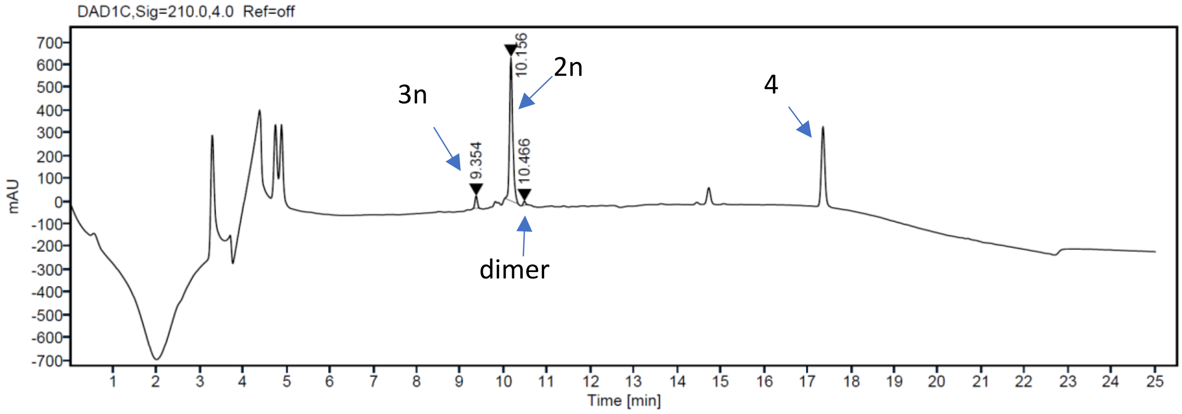

HPLC-UV chromatogram (210 nm) of **2n**:

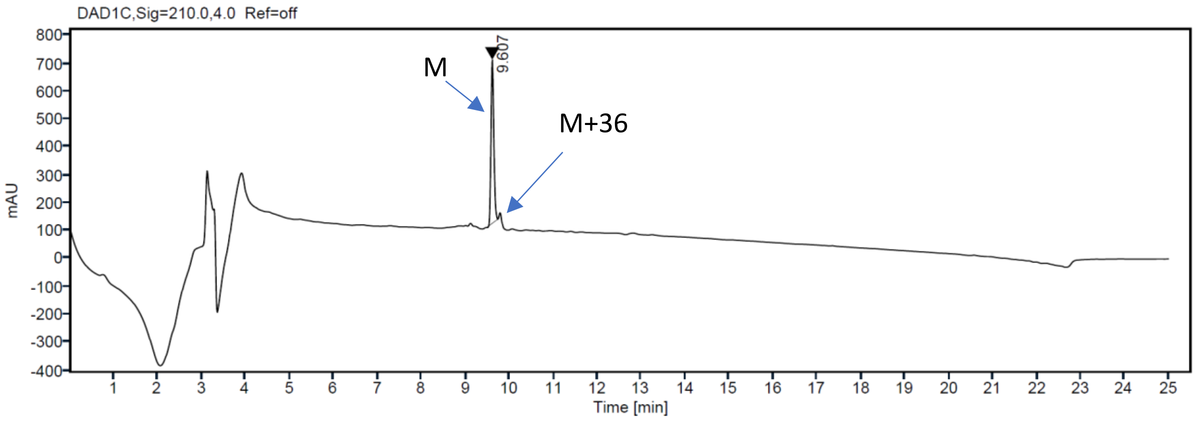

Retention time: 9.646 min **Area Percent:** 100%

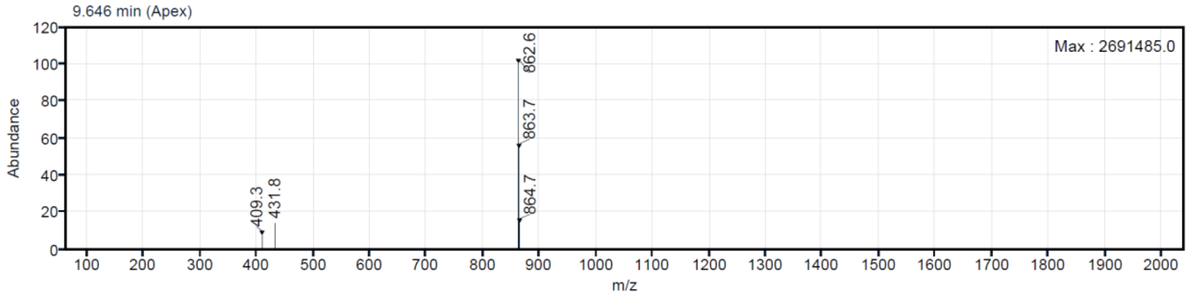

HRMS (nanochip-ESI/LTQ-Orbitrap) m/z: $[M + H]^+$ Calcd for $C_{43}H_{48}N_{11}O_9^+$ 862.3631; Found 862.3647.

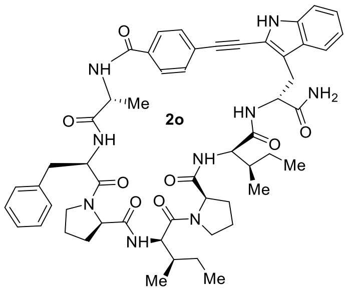

Following the general procedure, the reaction was conducted in 0.01 mmol scale. The desired product **2o** (4.4 mg, 5.3 µmol, 53% yield) was isolated by **Method 2**.

HPLC-UV chromatogram (210 nm) of the crude reaction mixture

HPLC-UV ratio of **2o**:**3o**: 93:7.

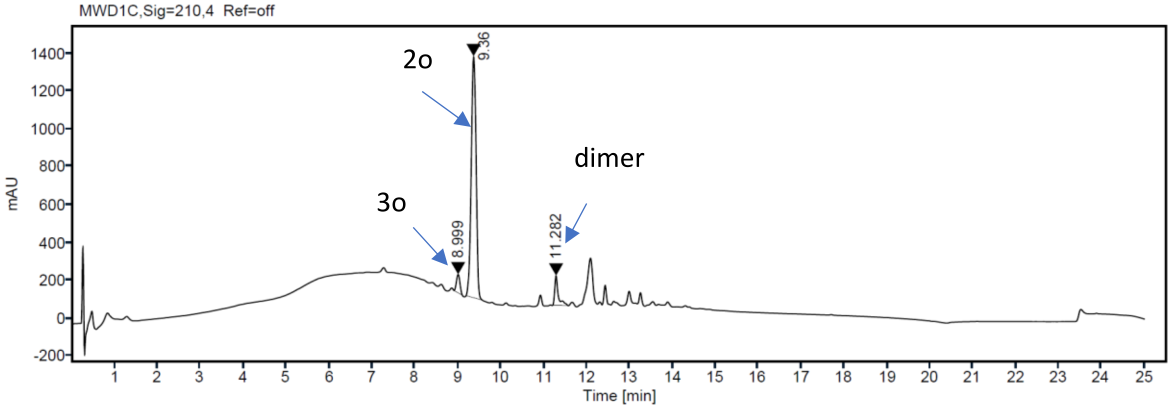

HPLC-UV chromatogram (210 nm) of **2o**:

HPLC-UV ratio of **2o**:**3o** after isolation: 89:11.

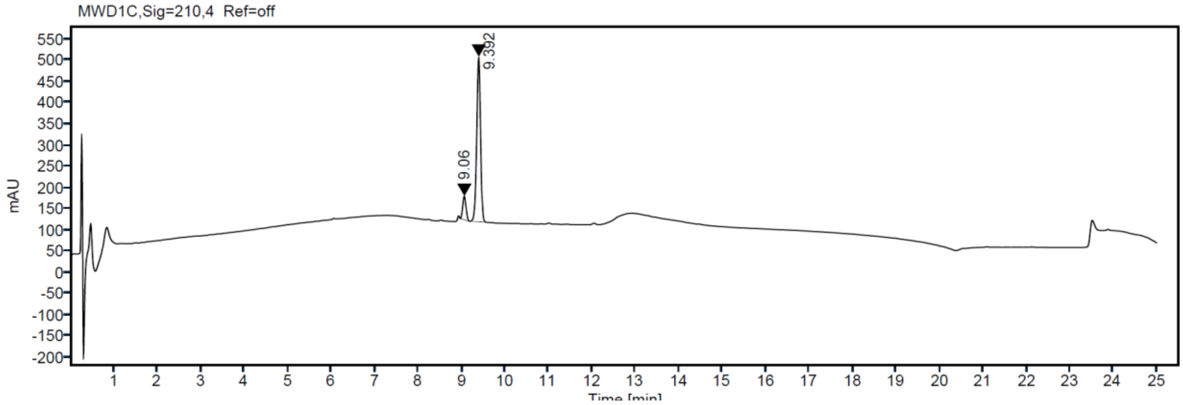

Retention time: 9.119 min **Area Percent:** 18%

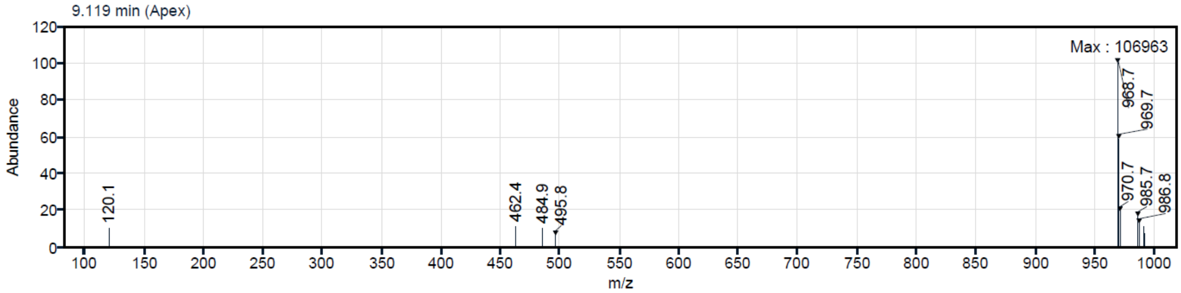

Retention time: 9.453 min **Area Percent:** 82%

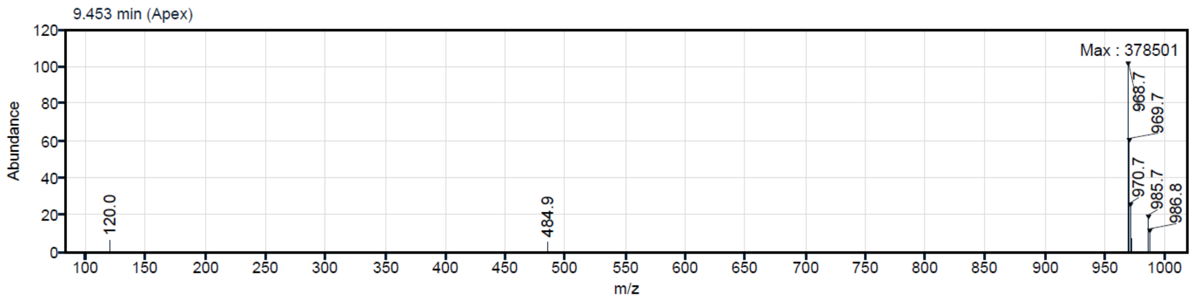

HRMS (ESI/QTOF) m/z: $[M + H]^+$ Calcd for $C_{54}H_{66}N_9O_8^+$ 968.5029; Found 968.5019.

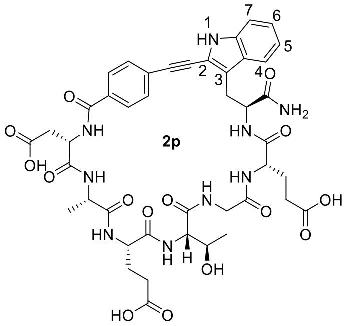

Following the general procedure, the reaction was conducted in 7.4 µmol scale. The desired product **2p** was isolated as two separable regiostereomers (**2p** 2.3 mg 2.5 µmol, 34% yield, **3p** 1.1 mg, 1.2 µmol, 16%, in total 49% yield) by **Method 3**.

HPLC-UV chromatogram (210 nm) of the crude reaction mixture

HPLC-UV ratio of P:P': 56:44.

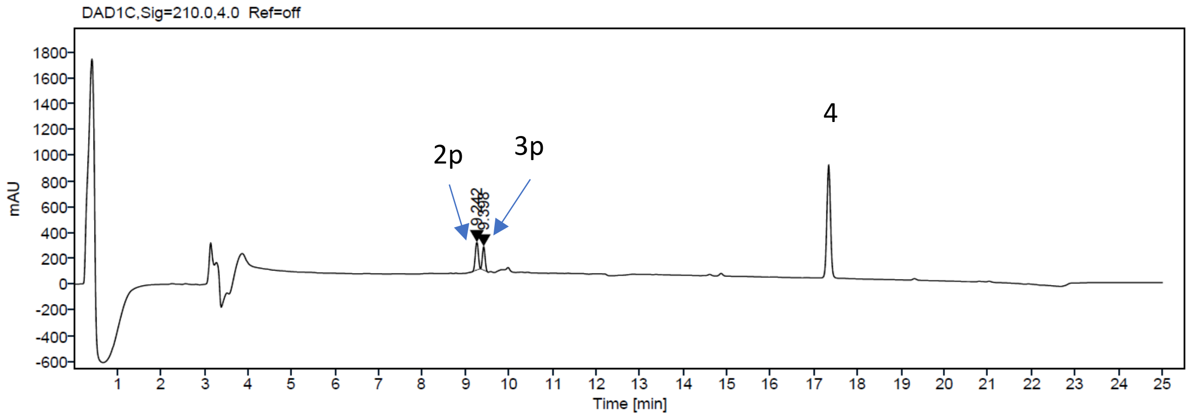

HPLC-UV chromatogram (210 nm) of **2p**:

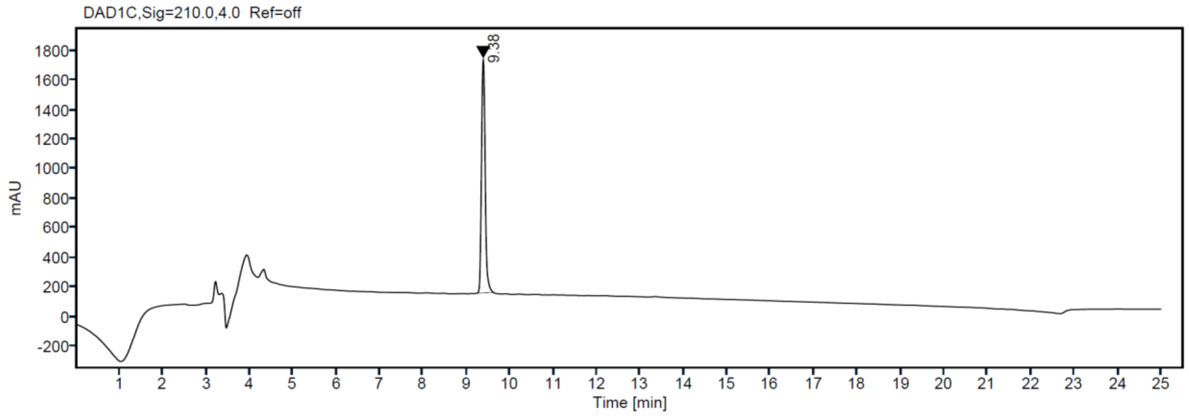

Retention time: 9.443 min **Area Percent:** 100%

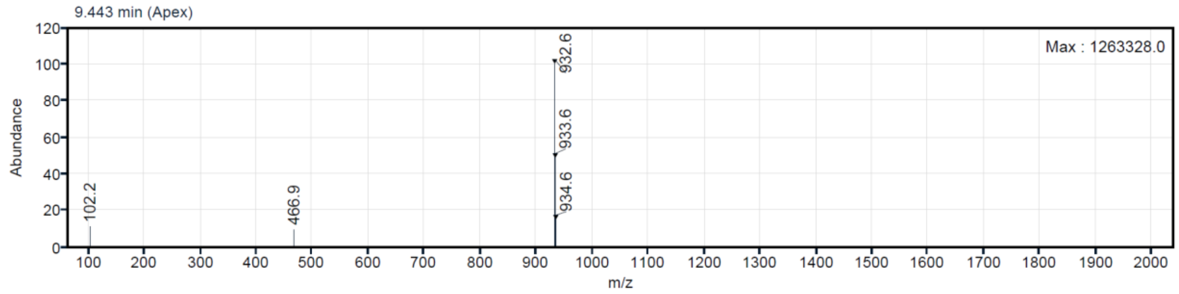

^{1}H NMR for **2p** (600 MHz, DMSO) δ 11.50 (s, 1H, NH(Trp)), 8.86 (d, *J* = 7.6 Hz, 1H, NH), 8.26 (d, *J* = 7.1 Hz, 1H, NH), 8.14 – 8.06 (m, 2H, 2 overlapping NH), 7.89 (d, *J* = 8.2 Hz, 2H, ArH(Phenylacetylene)), 7.82 (d, *J* = 6.4 Hz, 1H, NH), 7.73 (d, *J* = 7.8 Hz, 1H, NH), 7.66 (d, *J* = 8.1 Hz, 2H, ArH(Phenylacetylene)), 7.52 (d, *J* = 8.0 Hz, 1H, ArH(Trp C4)), 7.24 (d, *J* = 8.1 Hz, 1H, ArH(Trp C7)), 7.16 (d, *J* = 8.3 Hz, 1H, NH), 7.14 – 7.08 (m, 2H, NH and ArH(Trp C6)), 6.97 (t, *J* = 7.5 Hz, 1H, ArH(Trp C5)), 6.90 (s, 1H, NH), 4.69 (td, *J* = 7.8, 5.2 Hz, 1H), 4.56 (td, *J* = 8.3, 5.6 Hz, 1H), 4.32 (td, *J* = 8.1, 5.1 Hz, 1H), 4.21 – 4.10 (m, 3H), 4.04 – 3.97 (m, 1H), 3.70 (dd, *J* = 16.8, 5.8 Hz, 1H), 3.59 (dd, *J* = 16.8, 5.8 Hz, 1H), 3.23 – 3.19 (m, 1H), 3.08 (dd, *J* = 13.9, 5.4 Hz, 1H), 2.92 (dd, *J* = 16.6, 5.1 Hz, 1H), 2.75 (dd, *J* = 16.6, 8.2 Hz, 1H), 2.28 – 2.19 (m, 2H), 2.19 – 2.10 (m, 2H), 1.99 – 1.91 (m, 1H), 1.87 – 1.73 (m, 2H), 1.71 – 1.63 (m, 1H), 1.25 (d, *J* = 6.9 Hz, 3H, CH3(Thr)), 0.97 (d, *J* = 6.3 Hz, 3H, CH3(Ala)).
^{13}C NMR (126 MHz, DMSO) δ 174.0, 173.9, 172.8, 172.4, 172.3, 170.8, 170.5, 169.8, 169.7, 168.4, 166.1, 136.1, 133.4, 131.1, 127.8, 127.1, 125.4, 123.1, 119.4, 119.3, 117.5, 117.0, 111.2, 94.2, 84.2, 66.4, 57.5, 53.3, 52.9, 51.8, 50.5, 48.7, 42.2, 35.6, 30.2, 30.0, 28.0, 25.9, 19.2, 18.4.
HRMS (Nanochip-based ESI/LTQ-Orbitrap) m/z: $[M + H]^+$ Calcd for $C_{43}H_{50}N_9O_{15}^+$ 932.3421; Found 932.3426.

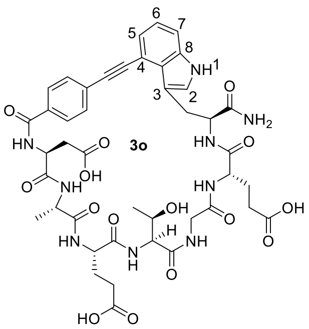

HPLC-UV chromatogram (210 nm) of **3p**:

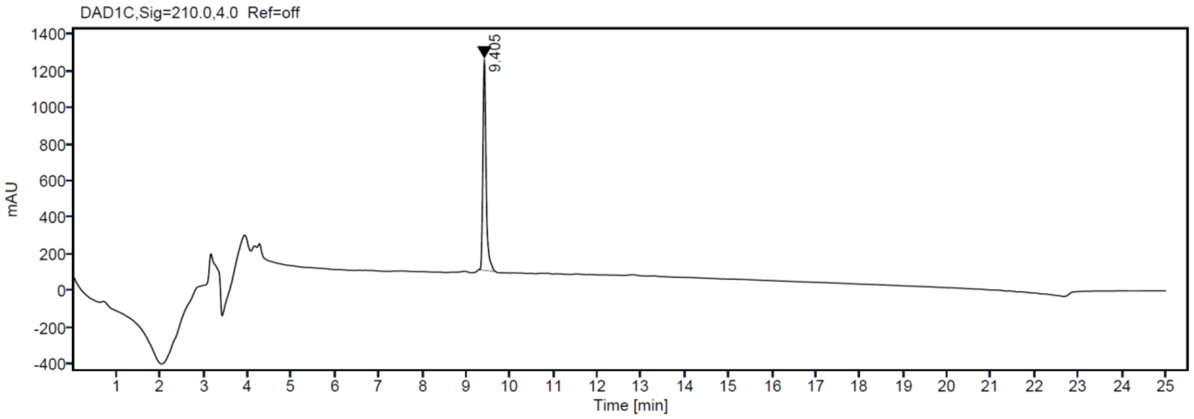

Retention time: 9.436 min **Area Percent:** 100%

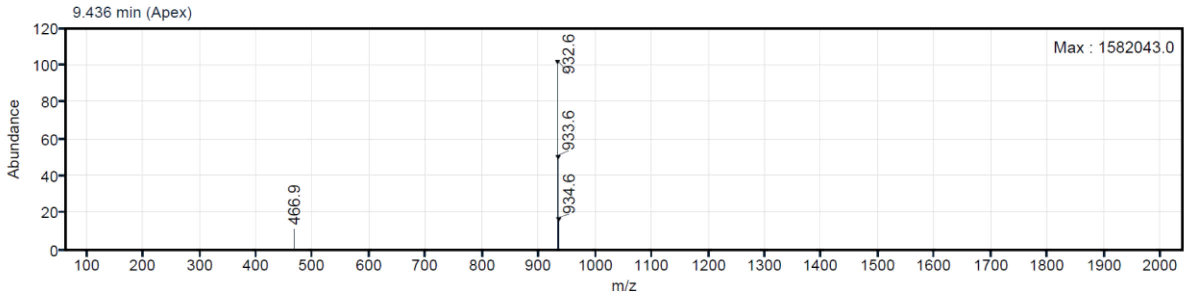

^{1}H NMR for **3p** (600 MHz, DMSO) δ 11.17 (s, 1H, NH(Trp)), 8.87 (d, *J* = 7.4 Hz, 1H, NH), 8.37 (d, *J* = 7.1 Hz, 1H, NH), 8.29 (d, *J* = 8.1 Hz, 1H, NH), 7.95 (d, *J* = 7.3 Hz, 1H, NH), 7.91 (d, *J* = 8.2 Hz, 2H, ArH(Phenylacetylene)), 7.90 – 7.86 (m, 2H, 2 overlapping NH), 7.67 (d, *J* = 8.2 Hz, 2H, ArH(Phenylacetylene)), 7.43 (dd, *J* = 8.1, 1.0 Hz, 1H, ArH (Trp C7)), 7.31 – 7.24 (m, 1H, ArH (Trp C5)), 7.22 (dd, *J* = 7.2, 1.0 Hz, 1H, NH), 7.17 (s, 1H, ArH (Trp C2)), 7.15 (s, 1H, NH), 7.14 – 7.08 (m, 2H, NH and ArH (Trp C6)), 4.78 (q, *J* = 7.2 Hz, 1H), 4.69 (q, *J* = 7.5 Hz, 1H), 4.34 – 4.24 (m, 2H), 4.22 – 4.17 (m, 1H), 4.15 – 4.09 (m, 1H), 4.05 – 3.99 (m, 1H), 3.95 (dd, *J* = 16.9, 6.6 Hz, 1H, CH2(Trp)), 3.64 (dd, *J* = 16.9, 4.4 Hz, 1H, CH2(Trp)), 3.53 (d, *J* = 7.2 Hz, 2H), 2.94 (dd, *J* = 16.5, 5.7 Hz, 1H), 2.74 (dd, *J* = 16.5, 7.9 Hz, 1H), 2.33 – 2.25 (m, 2H), 2.26 – 2.18 (m, 2H), 2.00 (dd, *J* = 14.0, 6.9 Hz, 1H), 1.90 – 1.79 (m, 2H), 1.71 (td, *J* = 14.2, 12.2, 7.4 Hz, 1H), 1.26 (d, *J* = 6.9 Hz, 3H), 0.95 (d, *J* = 6.3 Hz, 3H, CH_3(Ala)).
^{13}C NMR (126 MHz, DMSO) δ 174.0 173.8, 173.1, 172.3, 172.2, 171.0, 170.9, 170.0, 169.0, 166.0, 136.3, 132.8, 130.9, 127.9, 126.7, 126.2, 124.2, 123.8, 120.9, 113.1, 112.4, 111.1, 92.4, 90.7, 66.6, 57.7, 53.3, 52.7, 52.4, 50.3, 48.5, 42.2, 35.2, 30.2, 30.0, 29.0, 28.4, 27.7, 26.0, 19.5, 18.6.
HRMS (ESI/QTOF) m/z: $[M + Na]^+$ Calcd for $C_{43}H_{49}N_9NaO_{15}^+$ 954.3240; Found 954.3232.

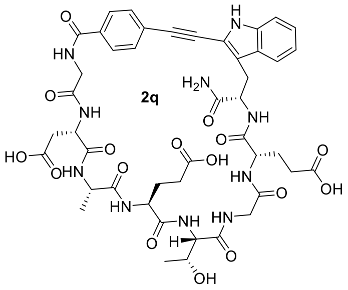

Following the general procedure, the reaction was conducted in 6.9 µmol scale. The desired product **2q** (3.2 mg, 3.2 µmol, 47% yield) was isolated by **Method 3**.
HPLC-UV chromatogram (210 nm) of the crude reaction mixture

HPLC-UV ratio of **2q**:**3q**: Not determined due to the overlap

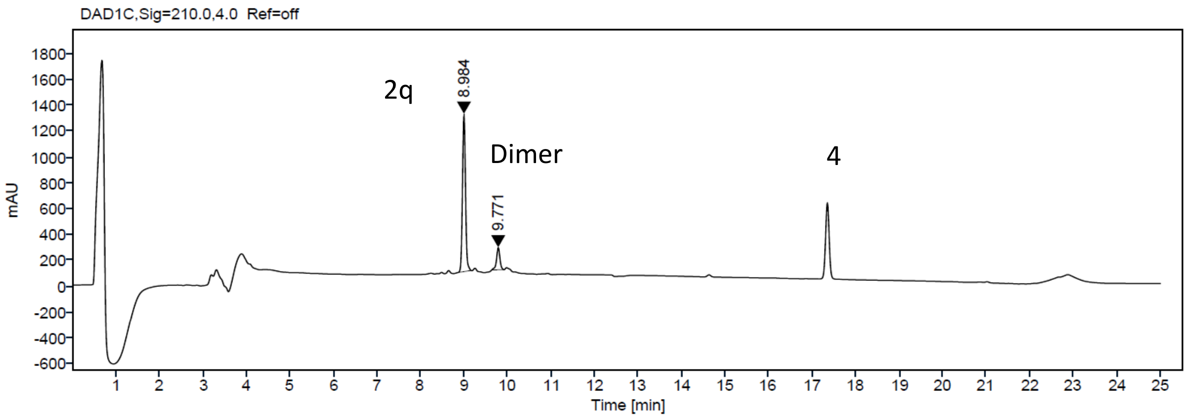

HPLC-UV chromatogram (210 nm) of **2q**:

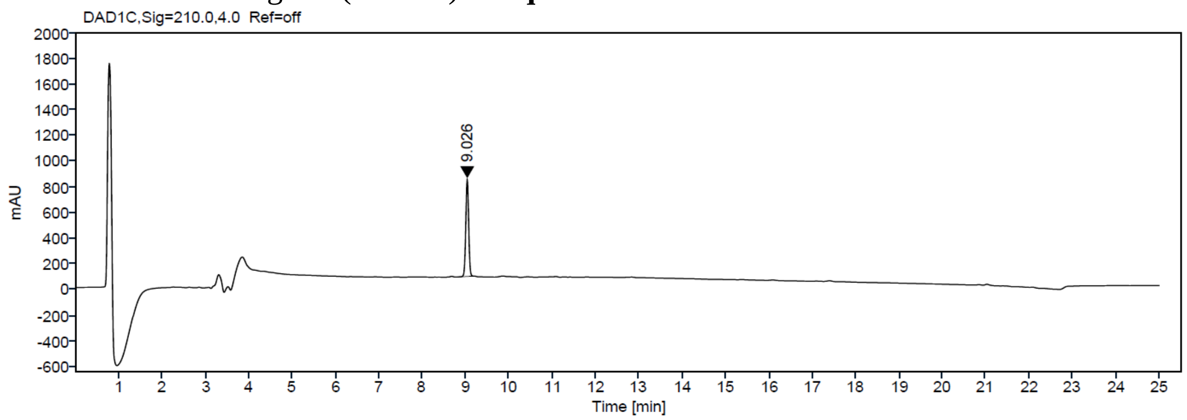

Retention time: 9.063 min **Area Percent:** 100%

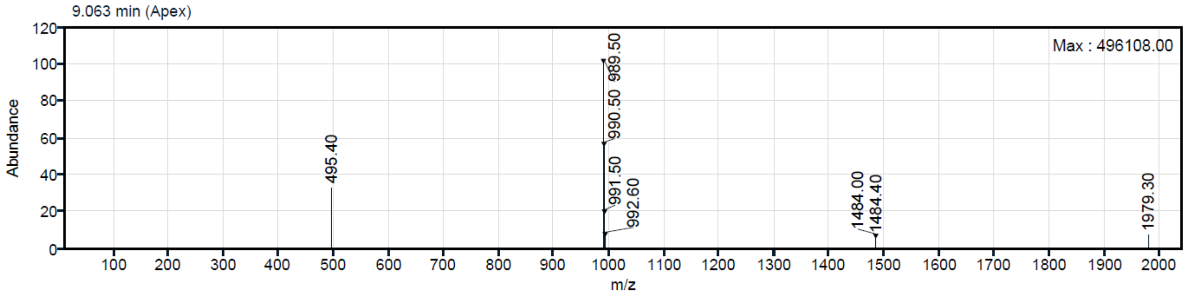

HRMS (Nanochip-based ESI/LTQ-Orbitrap) m/z: $[M + H]^+$ Calcd for $C_{45}H_{53}N_{10}O_{16}^+$ 989.3636; Found 989.3642.

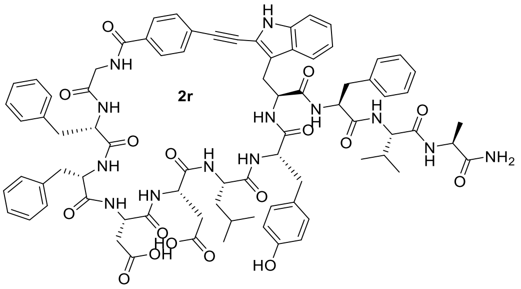

Following the general procedure, the reaction was conducted in 5.0 µmol scale. The desired product **2r** (4.2 mg, 2.8 µmol, 56% yield) was isolated by **Method 3**.

HPLC-UV chromatogram (210 nm) of the crude reaction mixture

HPLC-UV ratio of **2r**:**3r**: 99:1.

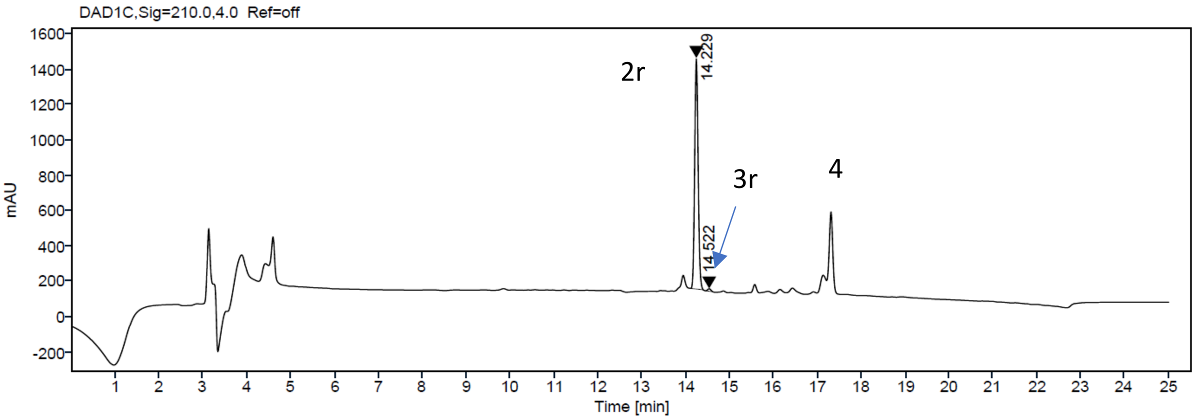

HPLC-UV chromatogram (210 nm) of **2r**:

HPLC-UV ratio of **2r**:**3r** after isolation: single diastereomer is isolated.

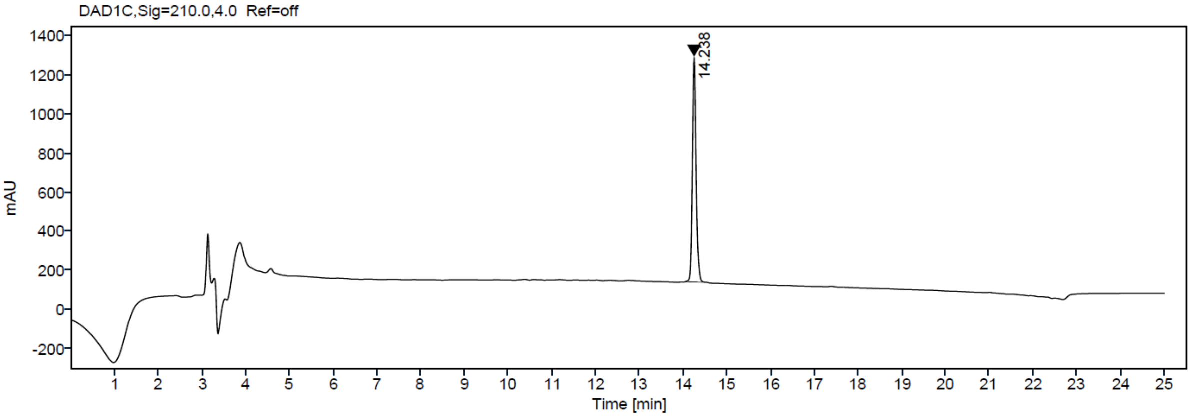

Retention time: 14.227 min **Area Percent:** 100%

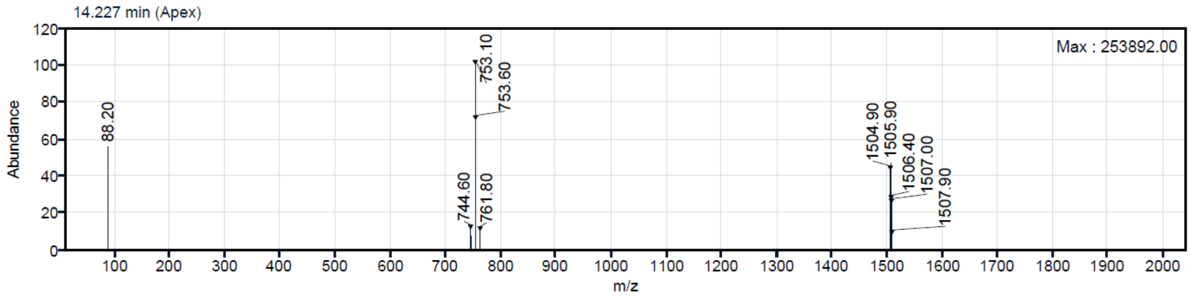

HRMS (Nanochip-based ESI/LTQ-Orbitrap) m/z: $[M + H]^+$ Calcd for $C_{80}H_{90}N_{13}O_{17}^+$ 1504.6572; Found 1504.6559.

MS/MS fragmentation of **2r**:

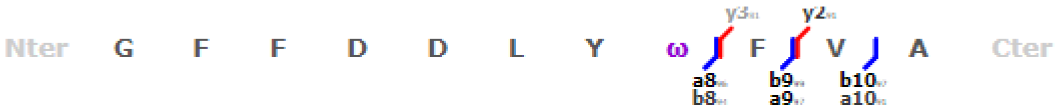

ω = Trp(H-1)
Nter = C9H4O
Cter = NH2

Sequence	Type	MF	MF Mass	m/z	Intensity	Similarity
GFFDDLYW	a8	C62H64N9O13(+1)	1142.462	1142.462	68.94	99.00%
GFFDDLYWF	b9	C72H73N10O15(+1)	1317.526	1317.525	41.45	98.98%
GFFDDLYWFVA		C80H89N13O17	1503.65	752.8322	100.4	98.76%
GFFDDLYW	b8	C63H64N9O14(+1)	1170.457	1170.457	50.35	98.37%
GFFDDLYWF	a9	C71H73N10O14(+1)	1289.531	645.2687	8.72	98.36%
GFFDDLYWF	b9	C72H73N10O15(+1)	1317.526	659.2662	11.28	98.20%
GFFDDLYWFV	b10	C77H82N11O16(+1)	1416.594	1416.594	6.07	97.25%
GFFDDLYWF	a9	C71H73N10O14(+1)	1289.531	1289.53	11.75	96.48%
GFFDDLYWFV	b10	C77H82N11O16(+1)	1416.594	708.8004	7.78	96.15%
GFFDDLYWFV	a10	C76H82N11O15(+1)	1388.599	694.803	5.08	95.45%
GFFDDLYW	a8	C62H64N9O13(+1)	1142.462	571.7345	7.29	93.91%
VA	y2	C8H18N3O2(+1)	188.1399	188.1394	10.78	90.87%
GFFDDLYW	b8	C63H64N9O14(+1)	1170.457	585.732	3.57	89.86%
GFFDDLYWFV	a10	C76H82N11O15(+1)	1388.599	1388.599	0.54	87.40%
FVA	y3	C17H27N4O3(+1)	335.2083	335.2078	2.33	81.41%

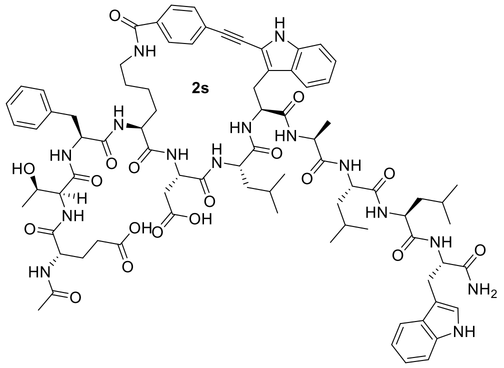

Following the general procedure, the reaction was conducted in 4.7 µmol scale. The desired product **2s** (2.8 mg, 1.8 µmol, 37% yield) was isolated by **Method 3**.

HPLC-UV chromatogram (210 nm) of the crude reaction mixture

HPLC-UV ratio of 4 isomers (i/i+3 and i/i+7): 7(**2s'** i/i+7) (14.498 min):2 (**3s'** i/i+7) (14.661 min):6 (**3s** i/i+3)(15.431 min):85 (**2s** i/i+3) (16.013 min)

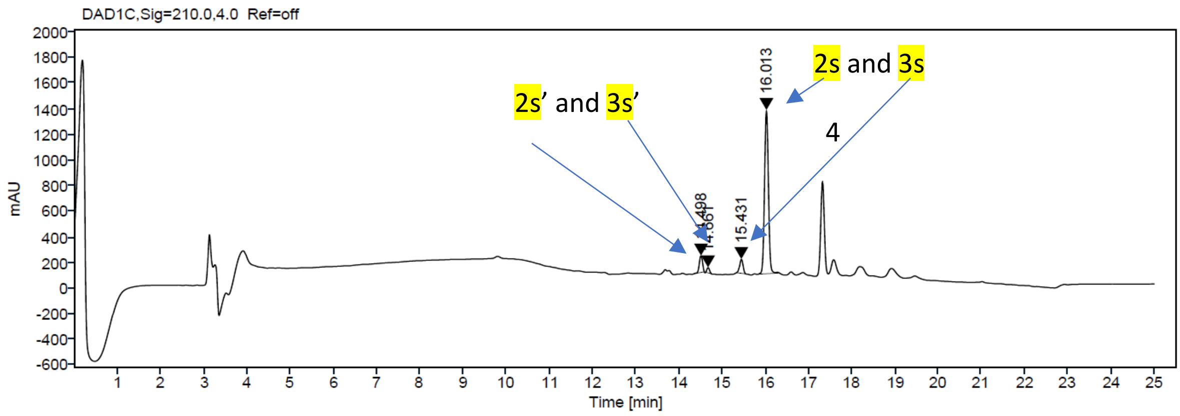

HPLC-UV chromatogram (210 nm) of **2s** (only major isomer was isolated):

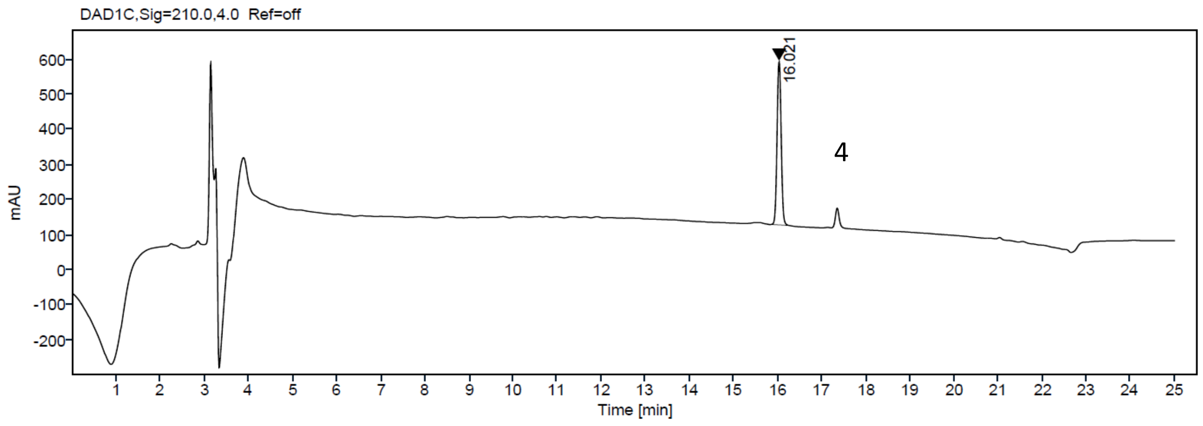

Retention time: 16.052 min **Area Percent:** 100%

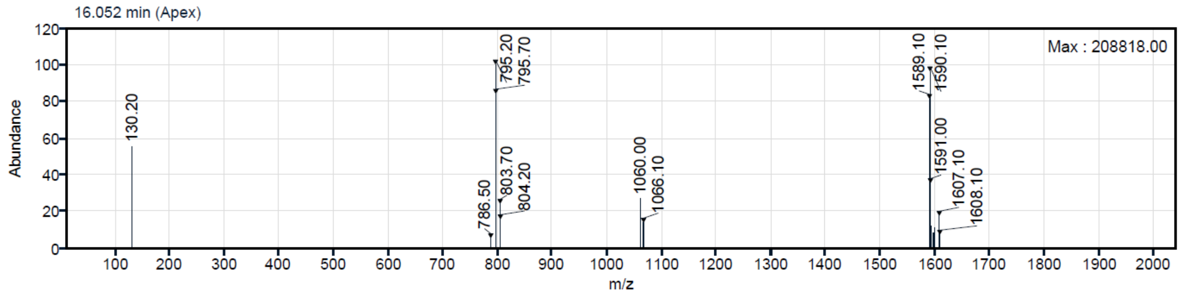

HRMS (Nanochip-based ESI/LTQ-Orbitrap) m/z: $[M + H_2]^{+2}$ Calcd for $C_{82}H_{107}N_{15}O_{18}{}^{+2}$ 794.8954; Found 794.8960.

MS/MS fragmentation of **2s**:

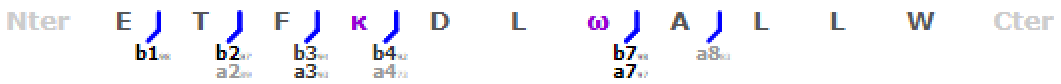

κ = Lys(C9H3O)
ω = Trp(H-1)
Nter = C2H3O
Cter = NH2

Sequence	Type	MF	MF Mass	m/z	Intensity	Similarity
ETFKDLW	b7	C56H66N9O14(+1)	1088.473	1088.472	3.75	98.40%
ETFKDLW	a7	C55H66N9O13(+1)	1060.478	1060.478	20.53	97.98%
E	b1	C7H10NO4(+1)	172.061	172.0604	18.46	97.91%
ET	b2	C11H17N2O6(+1)	273.1087	273.1081	32.91	97.32%
ETFKDLW	a7	C55H66N9O13(+1)	1060.478	530.7424	3.47	95.50%
ETF	b3	C20H26N3O7(+1)	420.1771	420.1765	4.06	94.09%
ETF	a3	C19H26N3O6(+1)	392.1822	392.1816	1.94	92.85%
ETFK	b4	C35H41N5O9(+1)	675.2904	675.2899	1.2	91.71%
ET	a2	C10H17N2O5(+1)	245.1137	245.1132	0.88	89.03%
ETFKDLWA	a8	C58H71N10O14(+1)	1131.515	1131.515	1.51	82.58%
ETFK	a4	C34H41N5O8(+1)	647.2955	647.295	3.13	72.97%

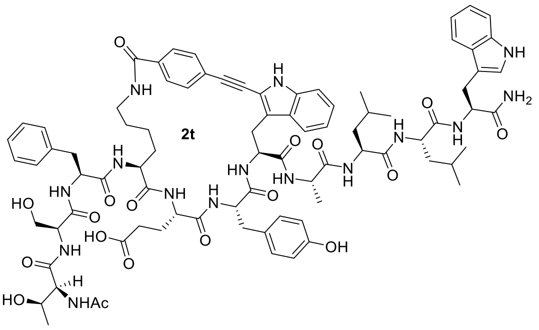

Following the general procedure, the reaction was conducted in 4.7 µmol scale. The desired product **2t** (3.4 mg, 2.1 µmol, 45% yield) was isolated by **Method 3**.

HPLC-UV chromatogram (210 nm) of the crude reaction mixture

HPLC-UV ratio of 4 isomers: 2(**2t’** i/i+7) (12.888 min):2 (**3t’** i/i+7) (13.01 min):3 (**3t** i/i+3) (13.182 min):92 (**2t** i/i+3) (14.036 min)

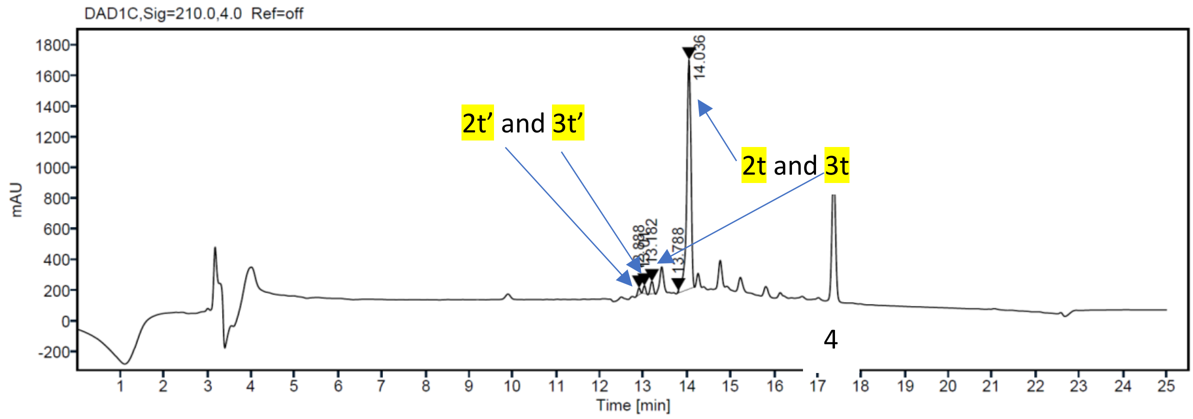

HPLC-UV chromatogram (210 nm) of **2t** (only major isomer was isolated):

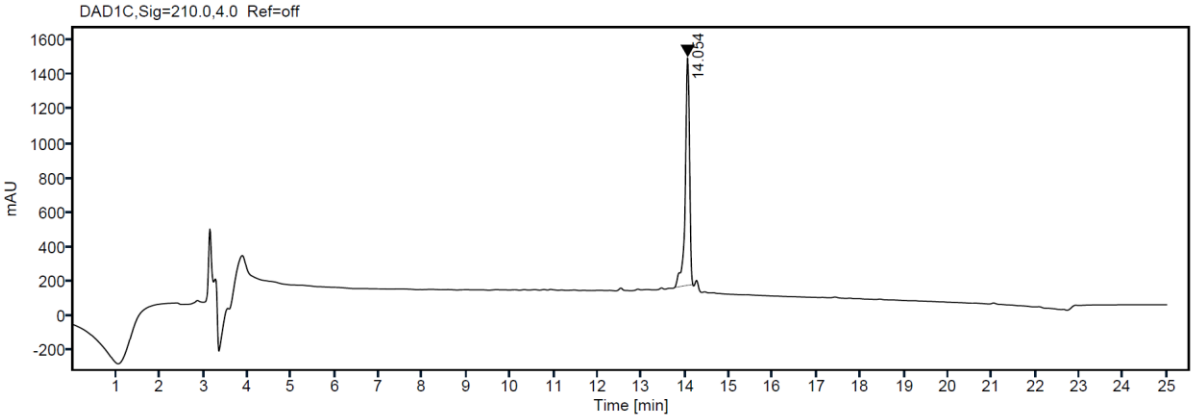

Retention time: 14.03 min **Area Percent:** 100%

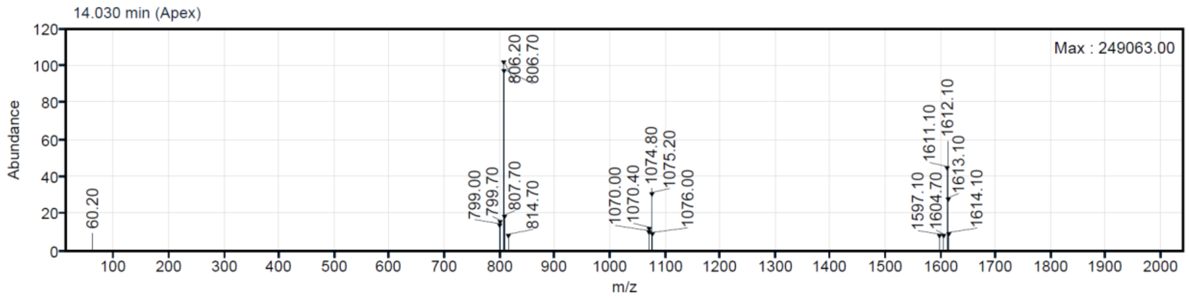

HRMS (Nanochip-based ESI/LTQ-Orbitrap) m/z: $[M + H_2]^{+2}$ Calcd for $C_{84}H_{105}N_{15}O_{18}{}^{+2}$ 805.8876; Found 805.8890.

MS/MS fragmentation of **2t**:

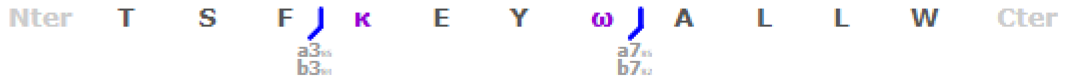

κ = Lys(C9H3O)
ω = Trp(H-1)
Nter = C2H3O
Cter = NH2

Sequence	Type	MF	MF Mass	m/z	Intensity	Similarity
TSFKEYW	a7	C57H64N9O13(+1)	1082.462	1082.462	17.73	95.20%
TSF	a3	C17H24N3O5(+1)	350.1716	350.171	6.04	84.86%
TSF	b3	C18H24N3O6(+1)	378.1665	378.166	6.87	83.86%
TSFKEYW	b7	C58H64N9O14(+1)	1110.457	1110.457	1.98	82.08%
TSFKEYW	a7	C57H64N9O13(+1)	1082.462	541.7345	2.29	74.83%

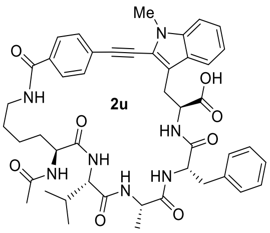

Following the general procedure, the reaction was conducted in 10 μmol scale. The desired product **2u** (5.3 mg, 6.3 μmol, 63% yield) was isolated by **Method 2**.
HPLC-UV chromatogram (210 nm) of the crude reaction mixture
HPLC-UV ratio of **2u**:**3u**: 98:2.

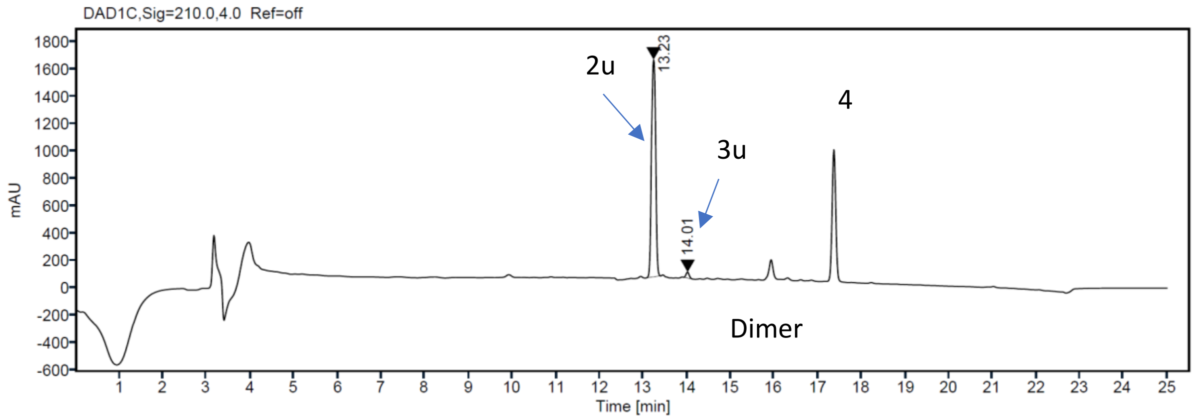

HPLC-UV chromatogram (210 nm) of **2u**
HPLC-UV ratio of **2u**:**3u**: only single diastereomer was isolated.

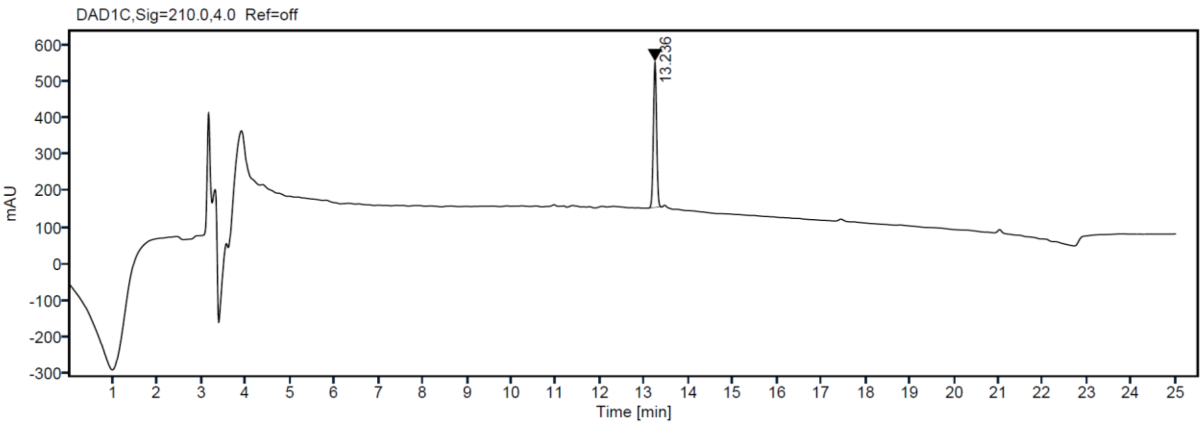

Retention time: 13.265 min **Area Percent:** 100%

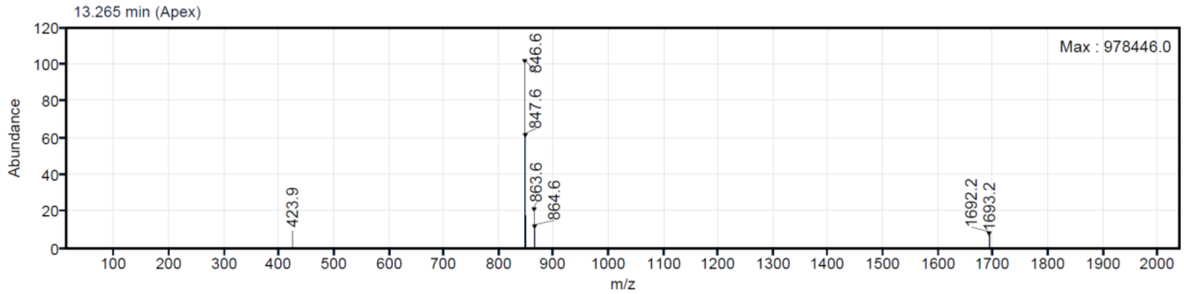

^{1}H NMR (500 MHz, DMSO) δ 8.46 (d, *J* = 8.2 Hz, 1H, NH), 8.29 (d, *J* = 8.2 Hz, 1H, NH), 8.19 (t, *J* = 5.4 Hz, 1H, NH), 8.15 (d, *J* = 7.0 Hz, 1H, NH), 7.94 (d, *J* = 7.7 Hz, 1H, NH), 7.87 (d, *J* = 8.0 Hz, 2H, ArH(Phenylacetylene)), 7.60 – 7.53 (m, 3H, ArH(phenylacetylene+Trp)), 7.47 (d, *J* = 8.3 Hz, 1H, ArH(Trp)), 7.25 (t, *J* = 7.7 Hz, 1H, ArH(Trp)), 7.20 – 7.14 (m, 3H, ArH(Phe)), 7.11 – 7.05 (m, 3H, ArH(Phe+Trp)), 6.93 (d, *J* = 7.5 Hz, 1H, NH), 4.80 (td, *J* = 7.8, 3.9 Hz, 1H), 4.64 – 4.57 (m, 1H), 4.34 (td, *J* = 7.9, 4.5 Hz, 1H), 4.26 – 4.18 (m, 1H), 3.87 (s, 3H), 3.84 – 3.77 (m, 1H), 2.95 (dd, *J* = 13.8, 4.6 Hz, 1H), 2.85 (dd, *J* = 13.8, 6.4 Hz, 1H), 1.82 (s, 3H), 1.68 – 1.54 (m, 4H), 1.52 – 1.44 (m, 2H), 1.38 – 1.28 (m, 3H), 0.90 (d, *J* = 7.0 Hz, 3H, CH_3(Ala)), 0.86 (d, *J* = 6.6 Hz, 3H, CH_3(Leu)), 0.82 (d, *J* = 6.5 Hz, 3H, CH_3(Leu)).
^{13}C NMR (126 MHz, DMSO) δ 173.0, 172.6, 171.5, 170.6, 169.6, 168.6, 165.7, 137.0, 136.8, 134.3, 130.6, 129.6, 127.9, 127.8, 126,5, 126.2, 124.4, 123.3, 121.5, 119.6, 119.4, 115.9, 110.0, 97.2, 82.1, 52.7, 51.9, 51.6, 51.2, 47.8, 37.9, 32.7, 30.9, 28.4, 27.7, 24.2, 23.0, 22.5, 21.7, 21.4, 17.4.
HRMS (ESI/QTOF) m/z: $[M + H]^+$ Calcd for $C_{47}H_{56}N_7O_8^+$ 846.4185; Found 846.4186.

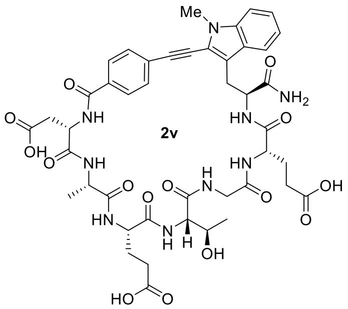

Following the general procedure, the reaction was conducted in 4.6 µmol scale. The desired product **2v** (3.7 mg, 3.9 µmol, 86% yield) was isolated by **Method 2**.
HPLC-UV chromatogram (210 nm) of the crude reaction mixture
HPLC-UV ratio of **2v**:**3v**: Not determined due to the overlap.

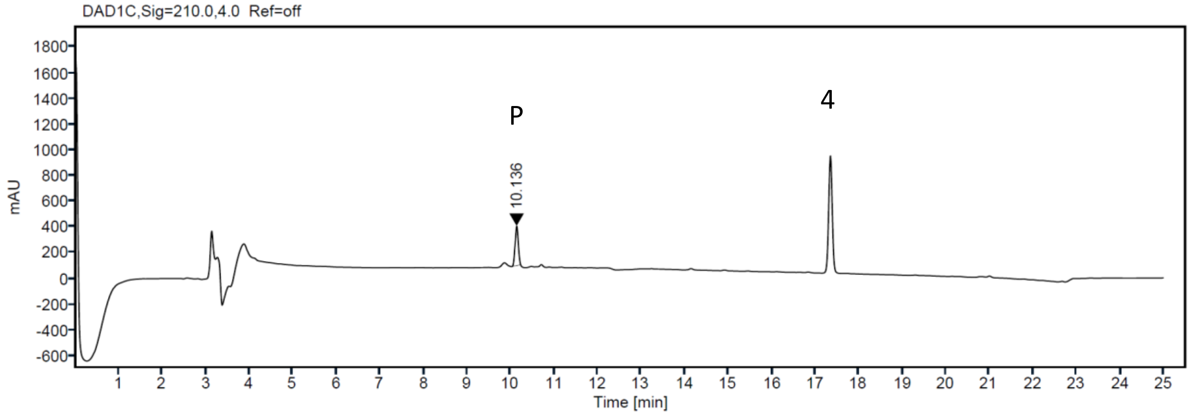

HPLC-UV chromatogram (210 nm) of **2v**:

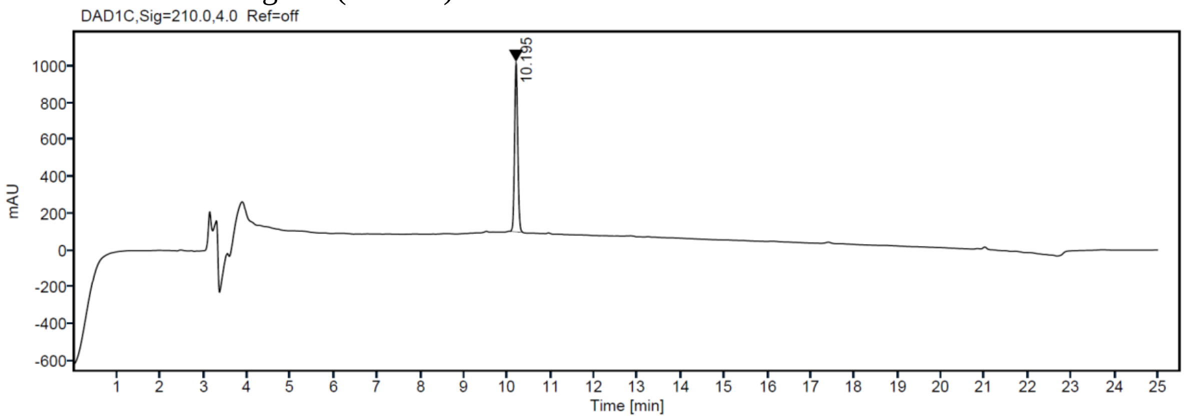

Retention time: 10.223 min **Area Percent:** 100%

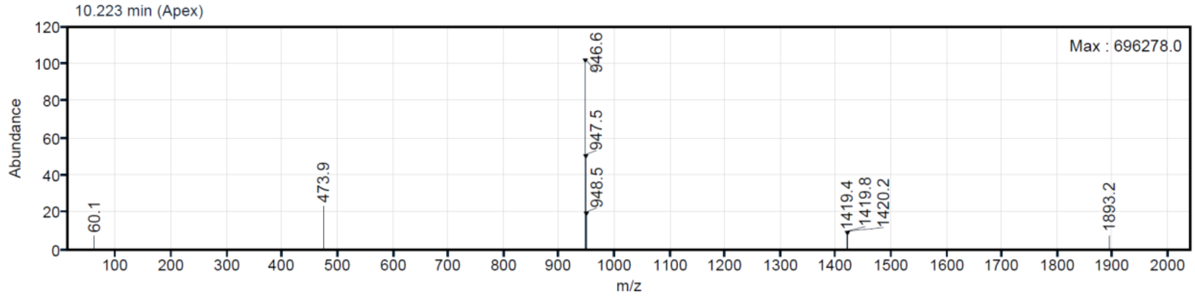

HRMS (ESI/QTOF) m/z: $[M + H]^+$ Calcd for $C_{44}H_{52}N_9O_{15}^+$ 946.3577; Found 946.3597.

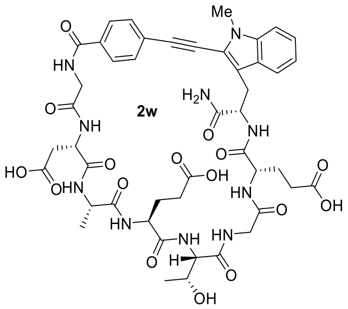

Following the general procedure, the reaction was conducted in 4.9 µmol scale. The desired product **2w** (3.0 mg, 3.0 µmol, 61% yield) was isolated by **Method 2**.
HPLC-UV chromatogram (210 nm) of the crude reaction mixture
HPLC-UV ratio of **2w**:**3w**: Not determined due to the overlap.

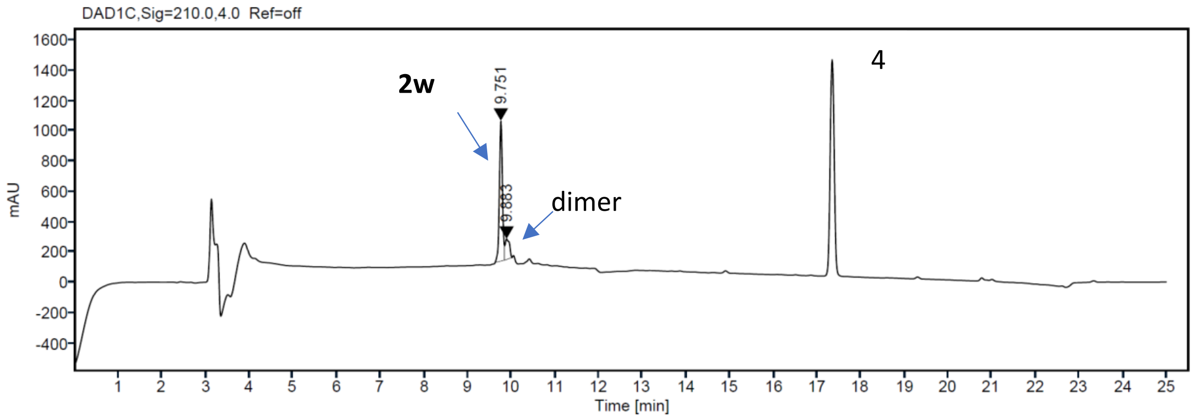

HPLC-UV chromatogram (210 nm) of **2w**:

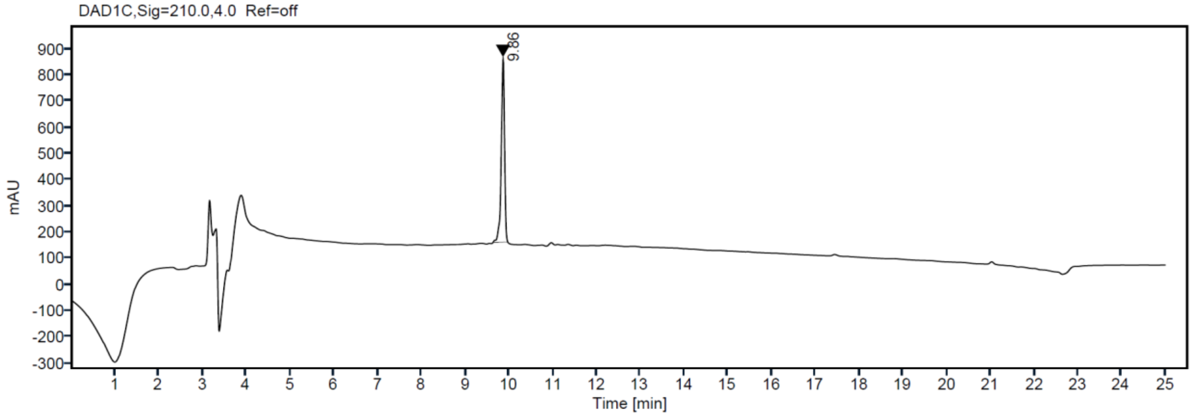

Retention time: 9.873 min **Area Percent:** 100%

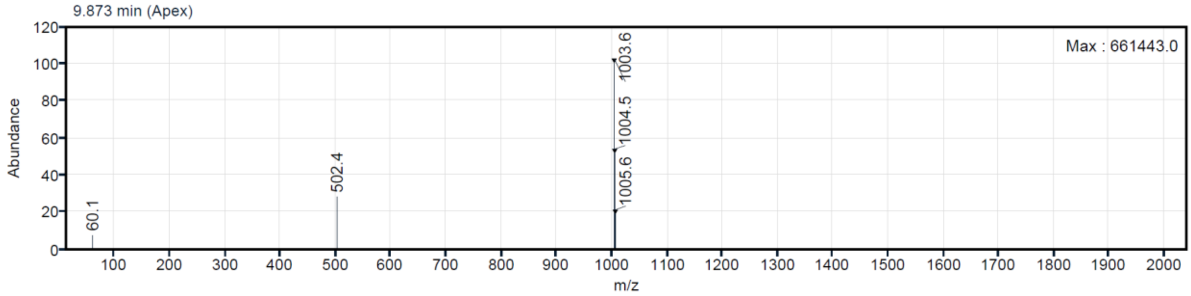

HRMS (ESI/QTOF) m/z: $[M + H]^+$ Calcd for $C_{46}H_{55}N_{10}O_{16}^+$ 1003.3792; Found 1003.3787.

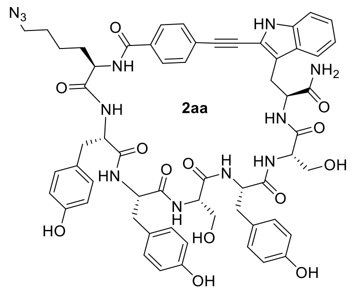

Following the general procedure, the reaction was conducted in 4.7 µmol scale. The desired product **2aa** (3.4 mg, 2.9 µmol, 53% yield) was isolated by **Method 2**.

HPLC-UV chromatogram (210 nm) of the crude reaction mixture

HPLC-UV ratio of **2aa**:**3aa**: 95:5

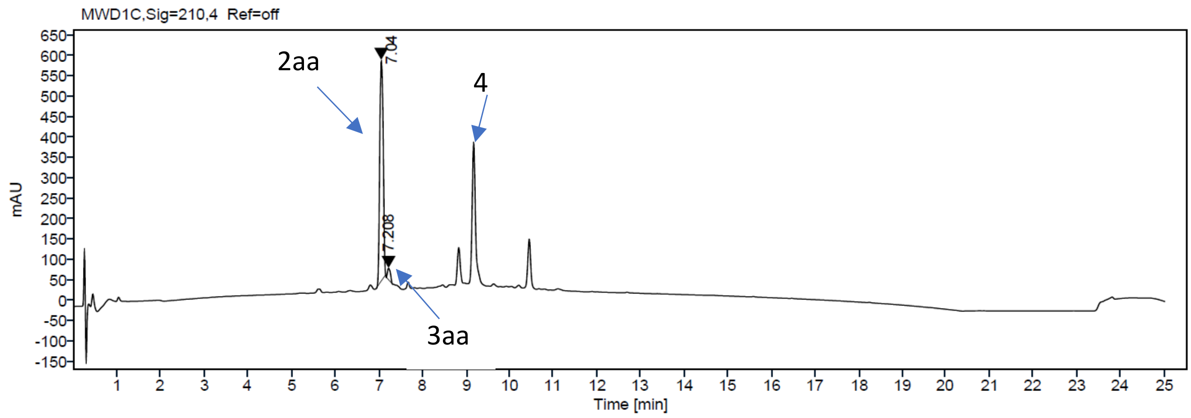

HPLC-UV chromatogram (210 nm) of **2aa**:

HPLC-UV ratio of **2aa**:**3aa** after isolation: 87:13

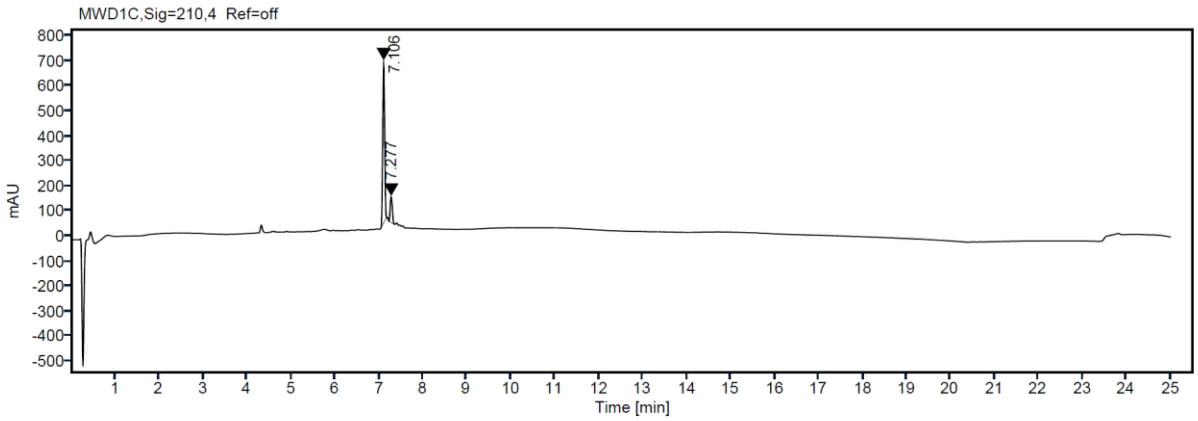

Retention time: 7.146 min **Area Percent:** 100%

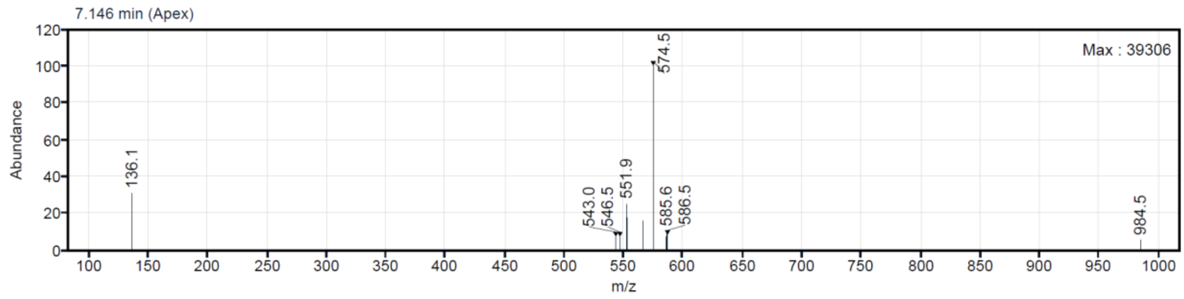

HRMS (nanochip-ESI/LTQ-Orbitrap) m/z: $[M + H]^+$ Calcd for $C_{59}H_{63}N_{12}O_{13}^+$ 1147.4632; Found 1147.4642.

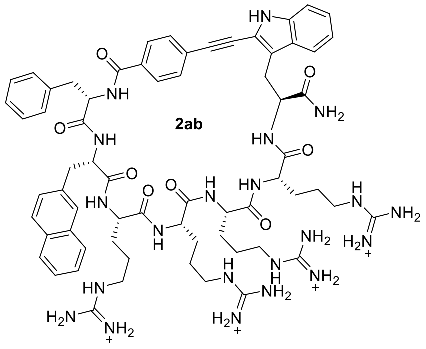

Following the general procedure, the reaction was conducted in 2.1 µmol scale. The desired product **2ab** (1.6 mg, 1.2 µmol, 57% yield) was isolated by **Method 3**.

HPLC-UV chromatogram (210 nm) of the crude reaction mixture

HPLC-UV ratio of **2ab**:**3ab**: 64:35.

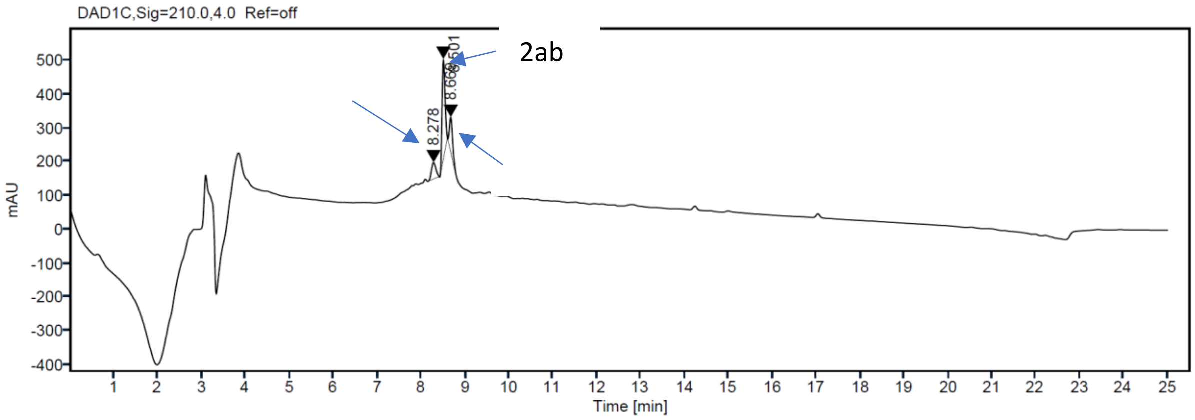

HPLC-UV chromatogram (210 nm) of **2ab**:

HPLC-UV ratio of **2ab**:**3ab** after isolation: 58:42.

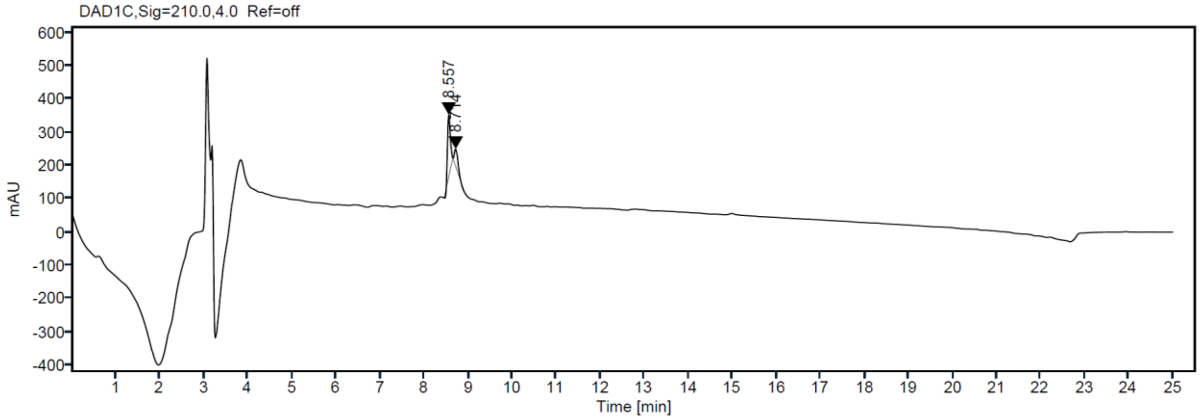

Retention time: 8.577 min **Area Percent:** 65%

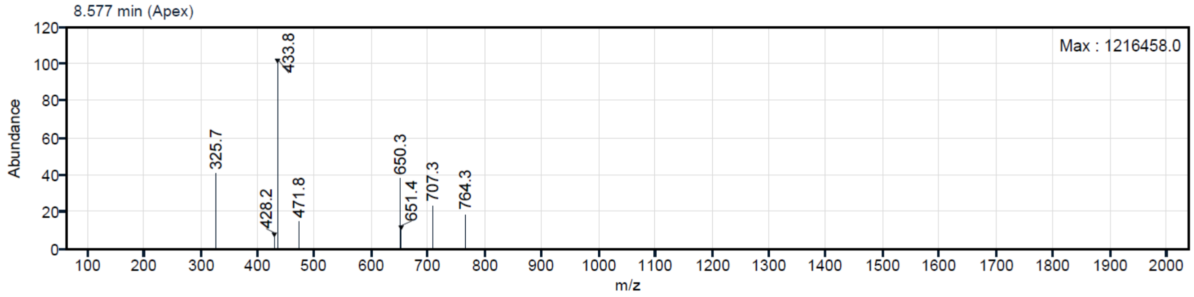

Retention time: 8.741 min **Area Percent:** 35%

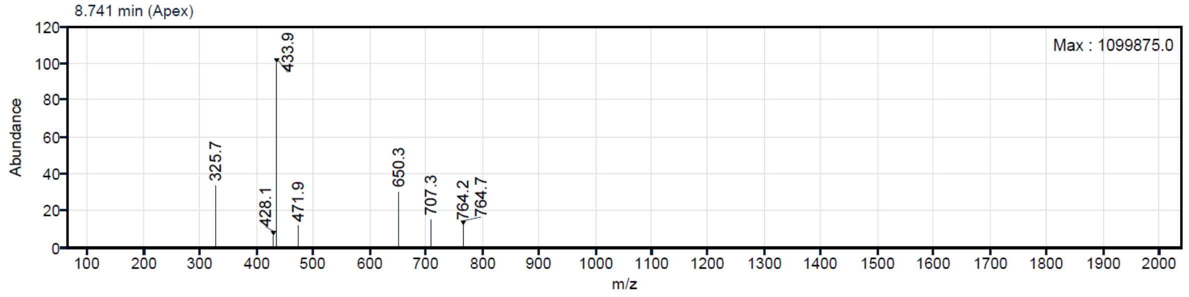

HRMS (nanochip-ESI/LTQ-Orbitrap) m/z: $[M + H_3]^{+3}$ Calcd for $C_{66}H_{86}N_{21}O_8^{+3}$ 433.5651; Found 433.5658.

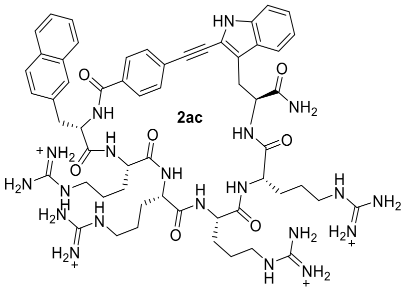

Following the general procedure, the reaction was conducted in 3.4 μmol scale. The desired product **2ac** (2.1 mg, 1.8 μmol, 53% yield) was isolated by **Method 2**.

HPLC-UV chromatogram (210 nm) of the crude reaction mixture

HPLC-UV ratio of **2ac**:**3ac**: 80:20. (The ratio is not precise due to the overlap)

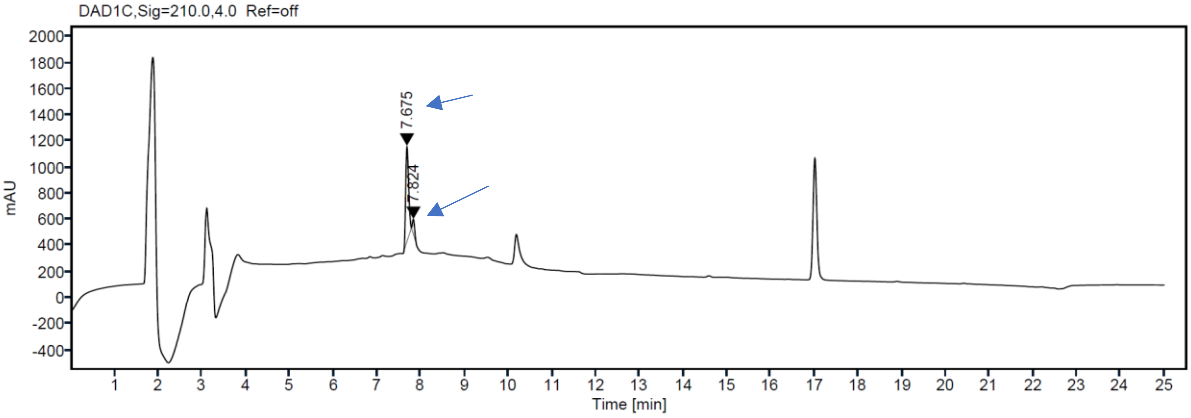

HPLC-UV chromatogram (210 nm) of **2ac:**

HPLC-UV ratio of P:P' after isolation: 80:20. (The ratio is not precise due to the overlap)

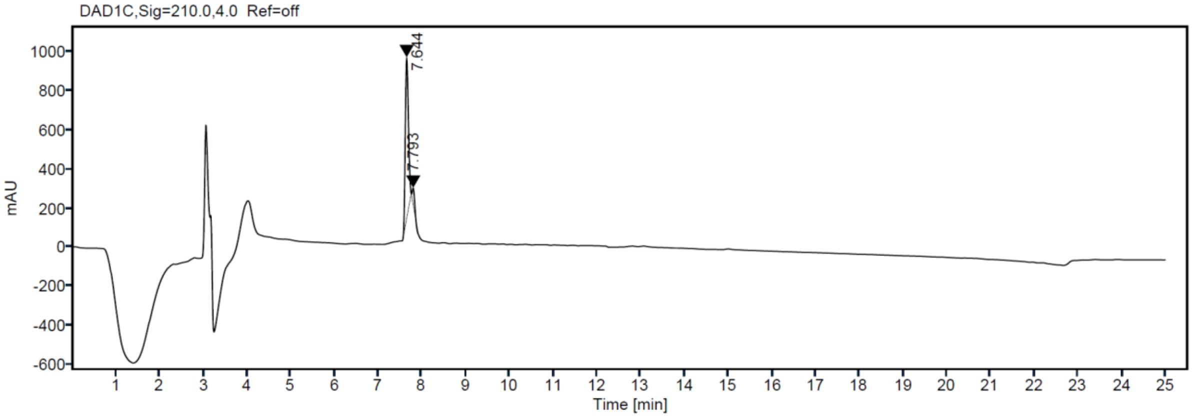

Retention time: 7.676 min **Area Percent:** 90%

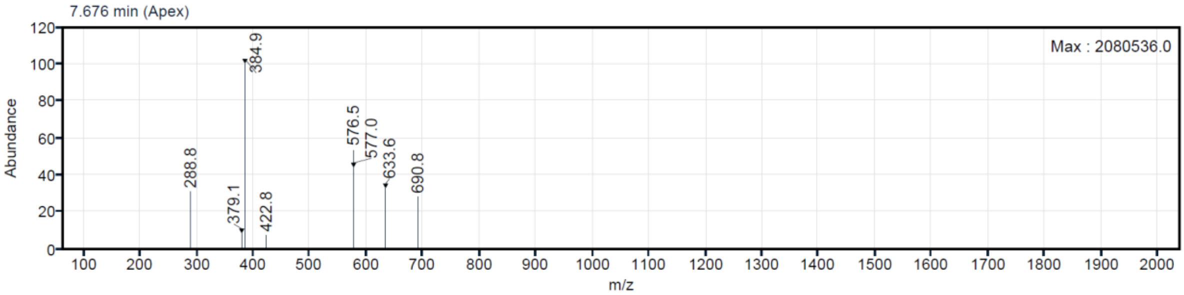

Retention time: 7.82 min **Area Percent:** 10%

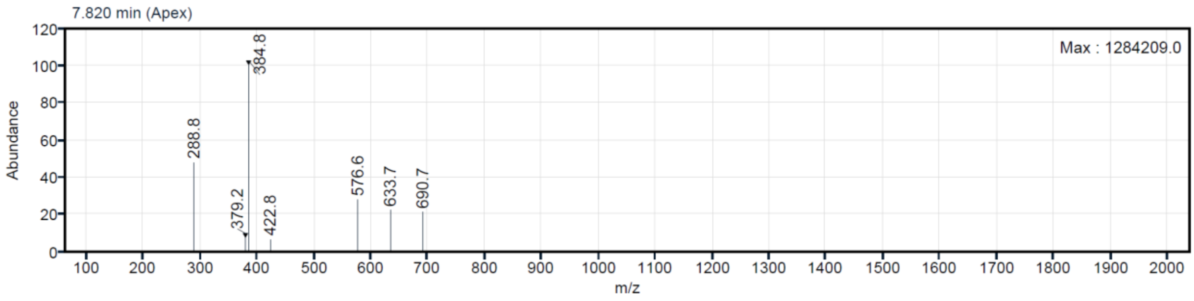

HRMS (nanochip-ESI/LTQ-Orbitrap) m/z: $[M + H_2]^{+2}$ Calcd for $C_{57}H_{76}N_{20}O_7^{+2}$ 576.3097; Found 576.3103.

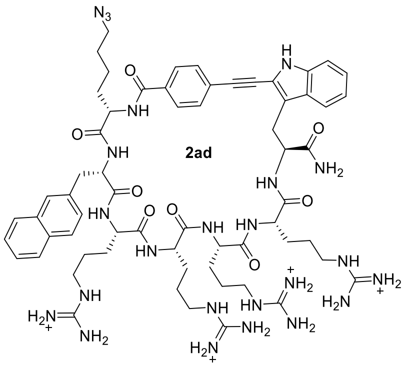

Following the general procedure, the reaction was conducted in 3.7 μmol scale. The desired product **2ad** (1.9 mg, 1.6 μmol, 44% yield) was isolated by **Method 2**.

HPLC-UV chromatogram (210 nm) of the crude reaction mixture

HPLC-UV ratio of **2ad**:**3ad**: 77:23. (The ratio is not precise due to the overlap)

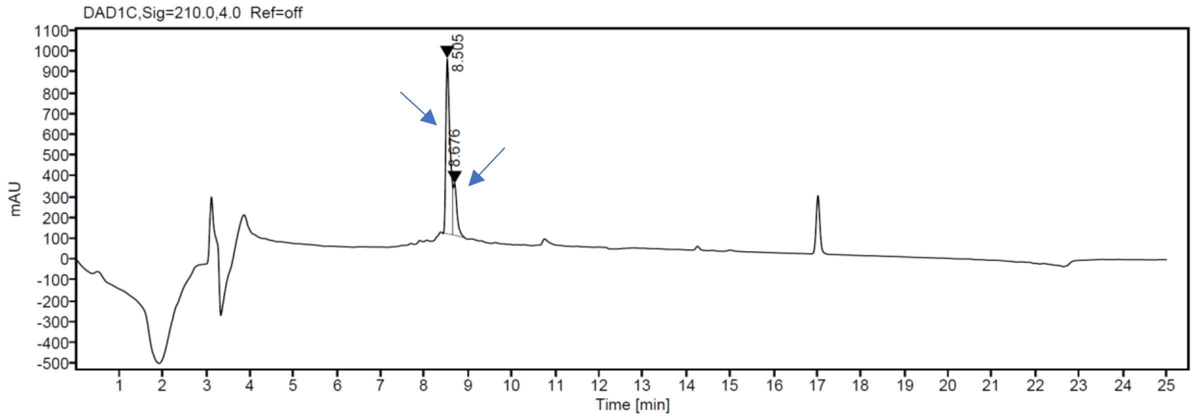

HPLC-UV chromatogram (210 nm) of the **2ad:**

HPLC-UV ratio of **2ad**:**3ad** after isolation: 77:23. (The ratio is not precise due to the overlap)

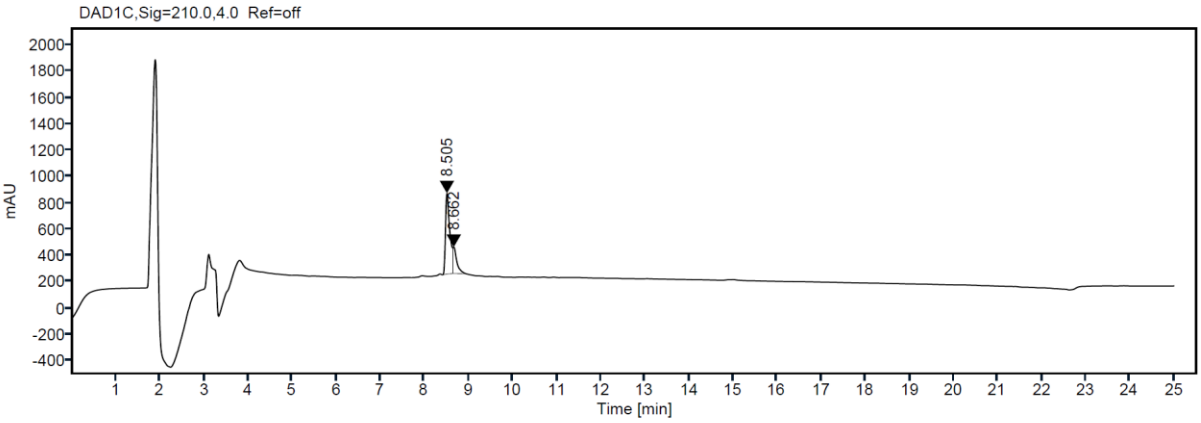

Retention time: 8.527 min **Area Percent:** 64%

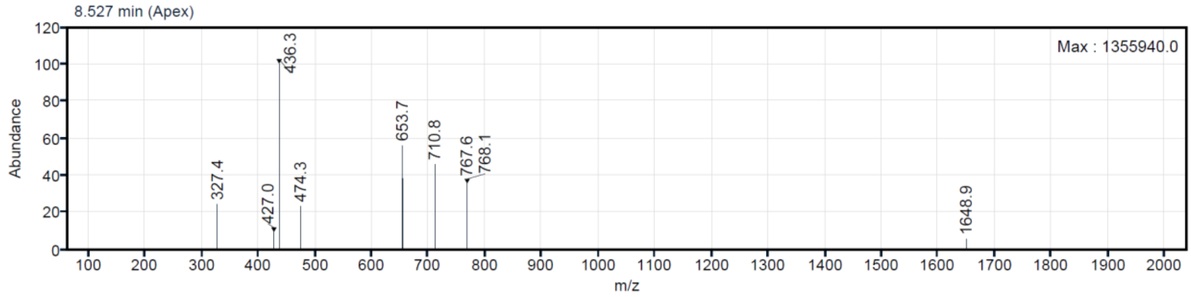

Retention time: 8.701 min **Area Percent:** 36%

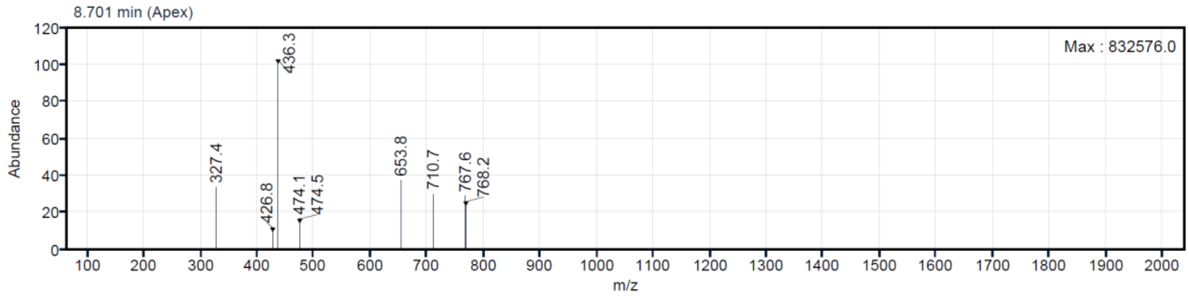

HRMS (nanochip-ESI/LTQ-Orbitrap) m/z: $[M + H_3]^{+3}$ Calcd for $C_{63}H_{87}N_{24}O_8{}^{+3}$ 435.9041; Found 435.9049.

6.4 Attempt of cyclization on solid phase

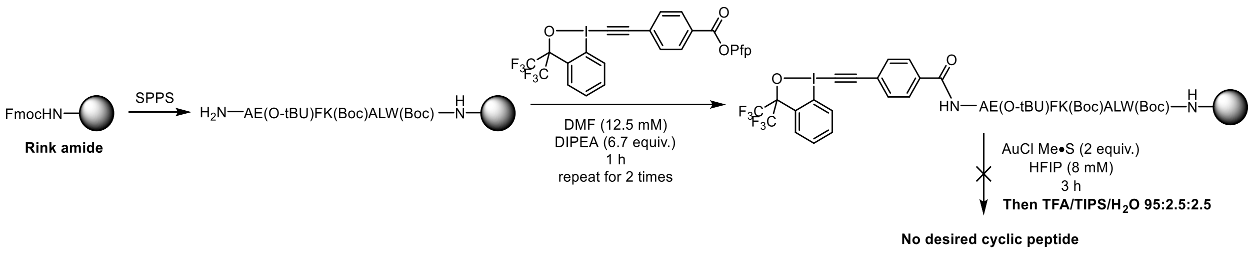

Figure S2: Attempt of cyclization on solid phase

Procedure: The synthesis was conducted on a 25 μmol scale from resin-bound substrate. Bifunctional EBX (34 mg, 50 μmol, 2.0 equiv.) was weighed into the syringe reactor. Then DIPEA (30.0 μL, 168 μmol, 6.7 equiv.) was added into the reactor and the mixture was agitated in DMF (12.5 mM) at room temperature for 1 hour. The resin was then filtered and washed with DCM (3 × 3 mL). The procedure was repeated one more time to ensure the *N*-terminus was fully reacted. Then the resin was agitated in HFIP (8 mM) with AuCl Me_2S (1.5 mg, 2.0

equiv.) for 3 hours. Following TFA/TIPS/H_2O (95:2.5:2.5) cleavage from resin and removal of volatiles, the crude peptide was analyzed by reverse-phase HPLC with **Method 2**. No desired cyclic peptide was observed.

Confirmation of solid phase synthesis of peptide-EBXs:

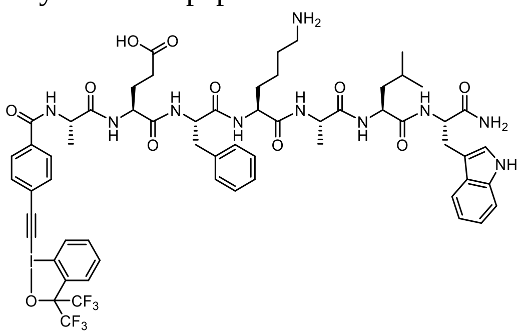

HPLC-UV chromatogram (210 nm) of peptide-EBXs crude after resin cleavage**:**

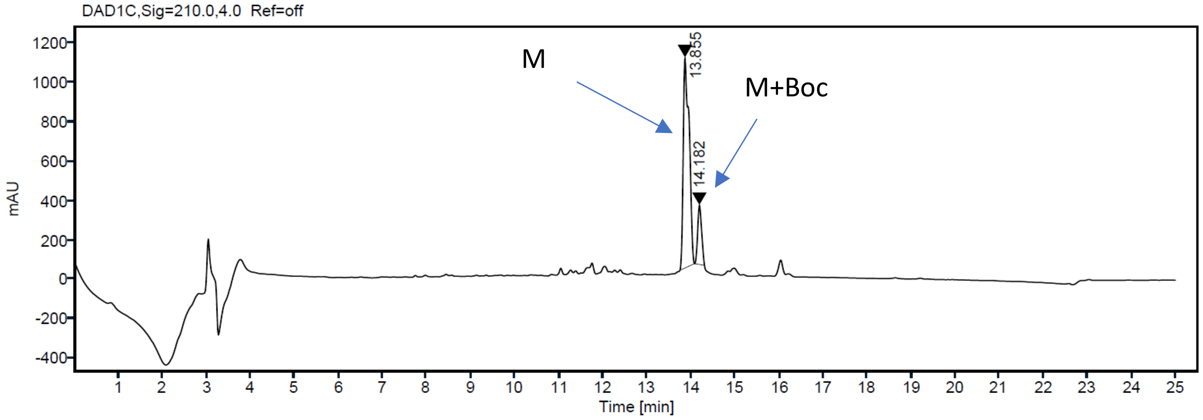

Retention time: 13.979 min **Area Percent:** 70%

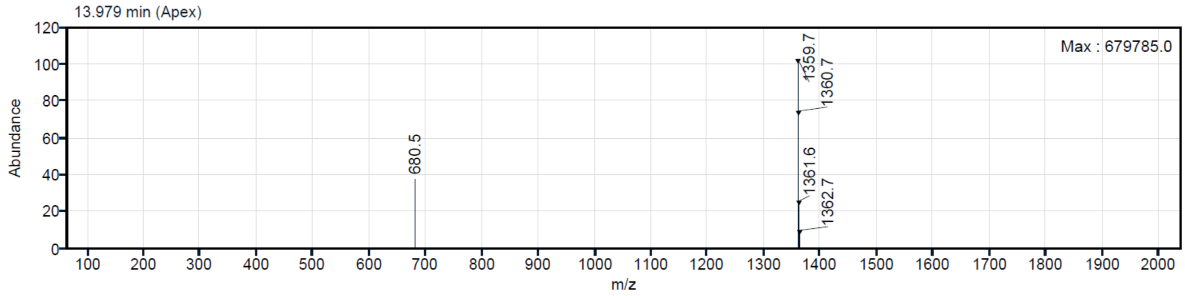

HPLC-UV chromatogram (210 nm) of the crude after on-resin cyclization:

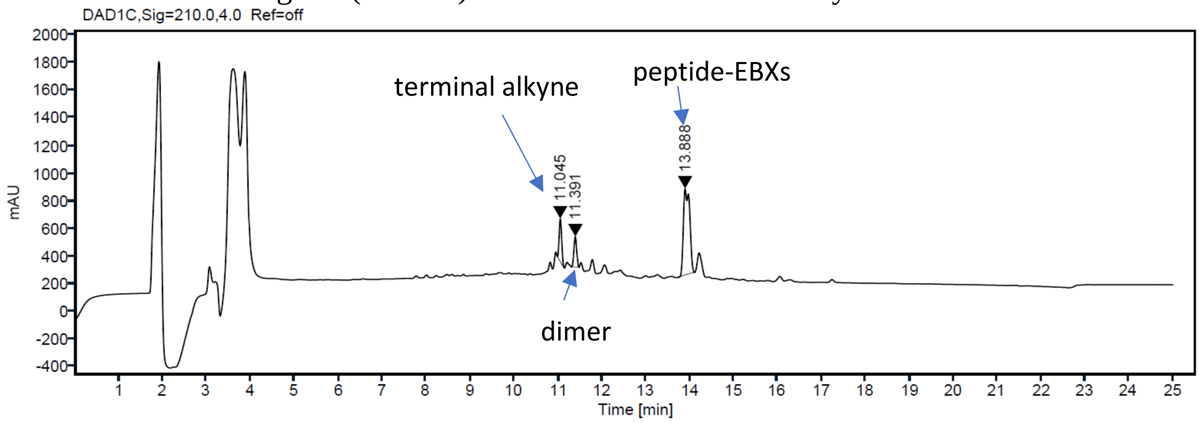

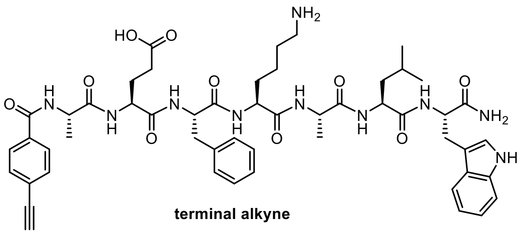

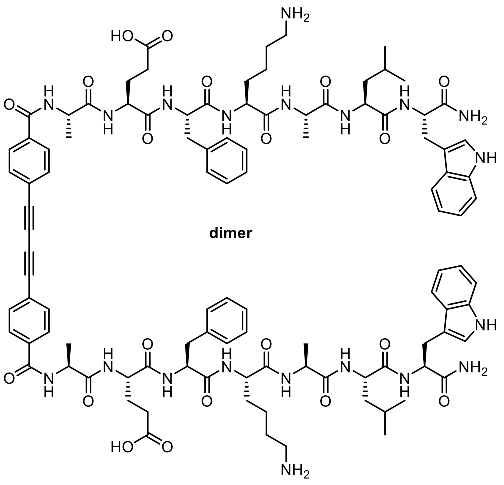

6.5 ICP analysis of cyclic peptides

Table S2: Determination of gold content by ICP-MS analysis

Sample name	Au, ng/mg[a]	Au%[b]
AcKLAFW (**2a**)	99.73	2.4%
GFFDDLYWFVA-NH_2 (**2r**)	57.22	2.4%
AcKSAFW (**2i**)	276.94	7.6%

[a]RSD: 2.50%. [b] % of gold incorporated in peptides

7. Studies of the structure of two regioisomers:

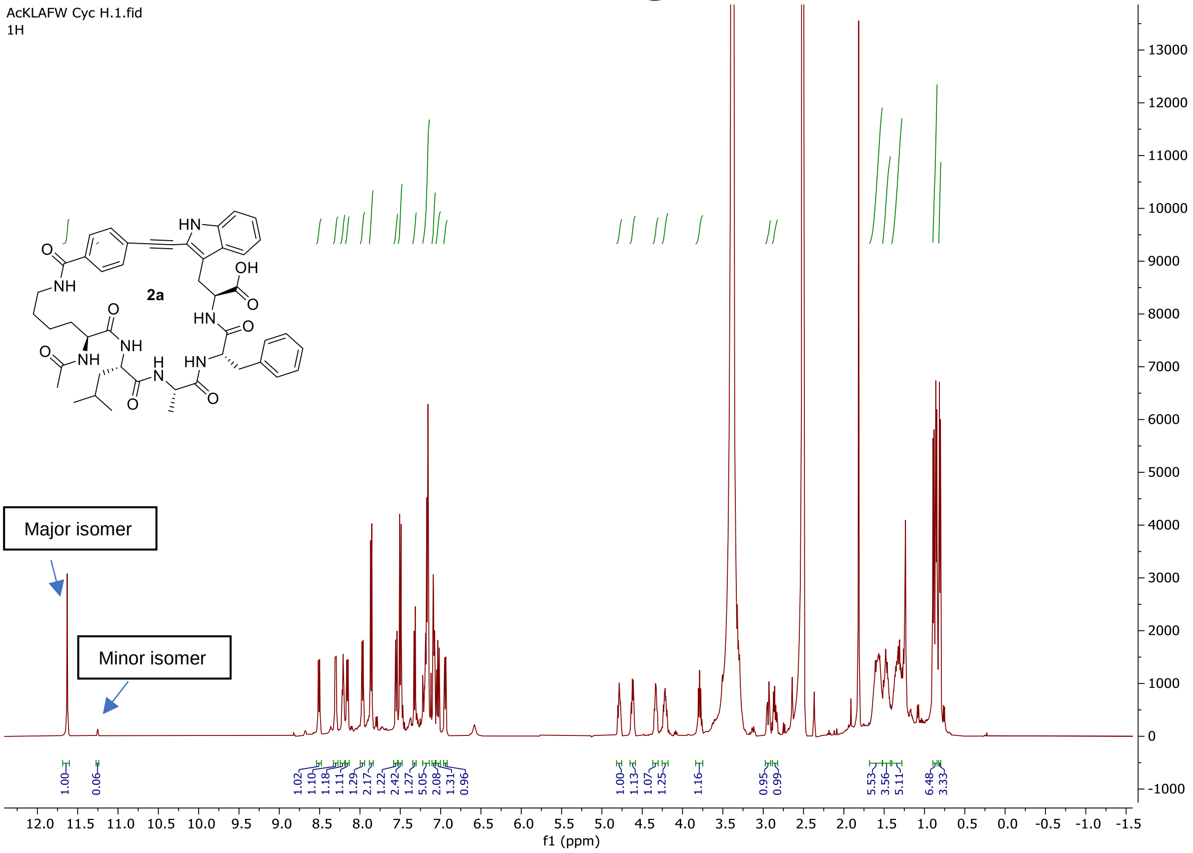

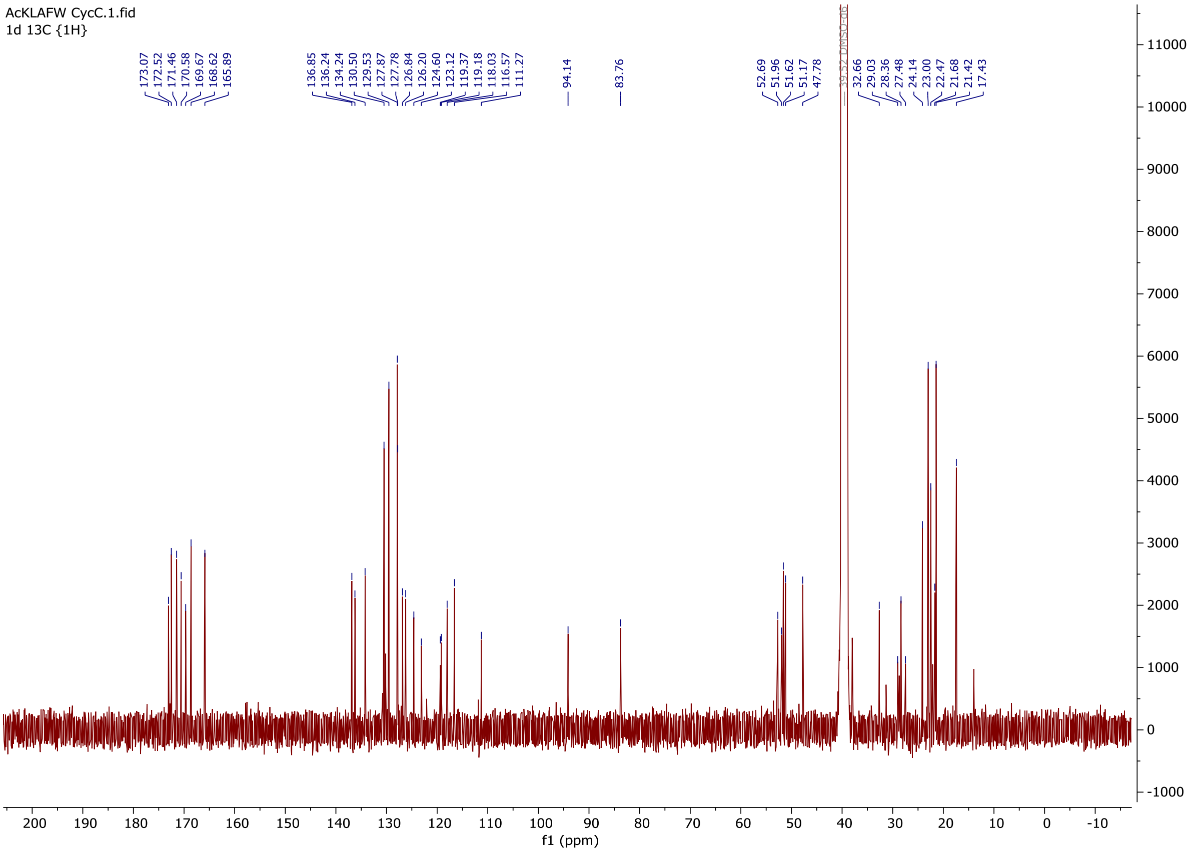

HMBC spectrum shows the weak interaction between alkyne and indole N-H, confirming the major product is Trp C-2 alkynylation product.

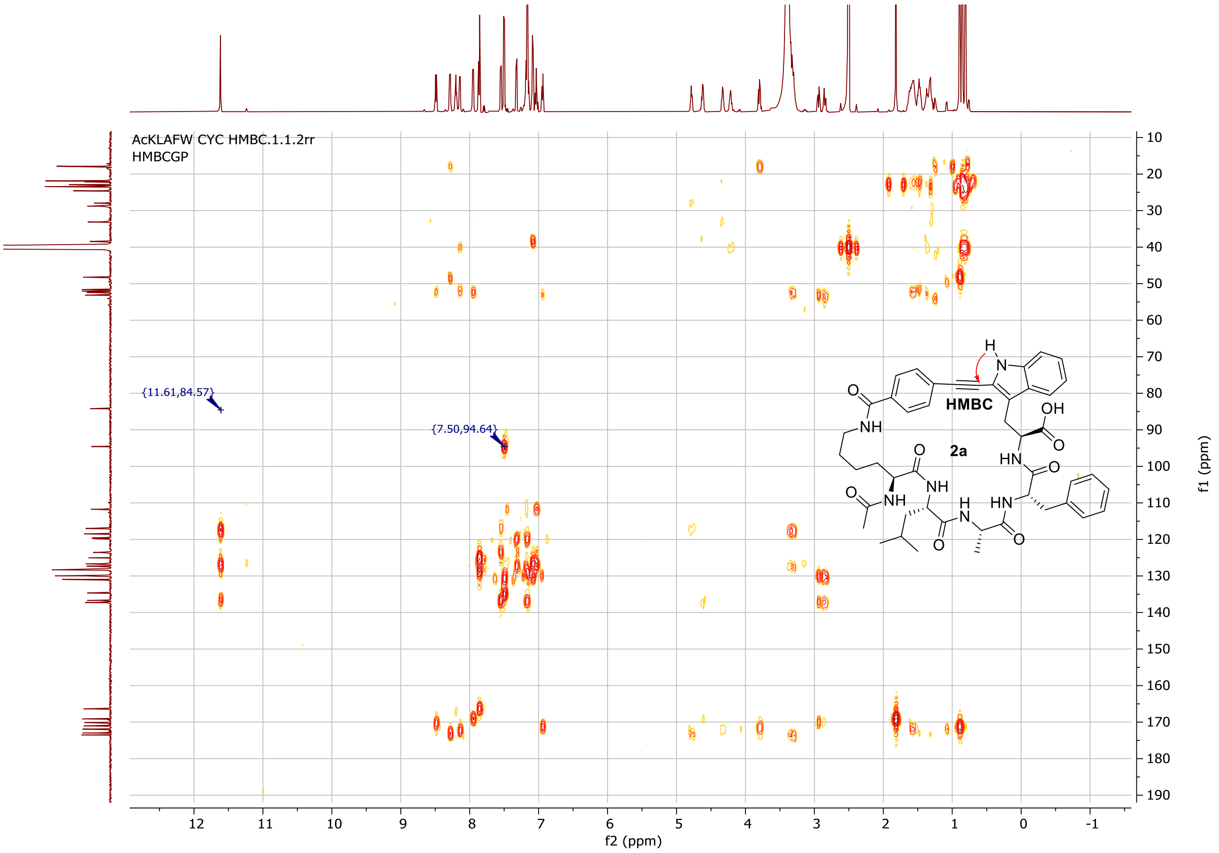

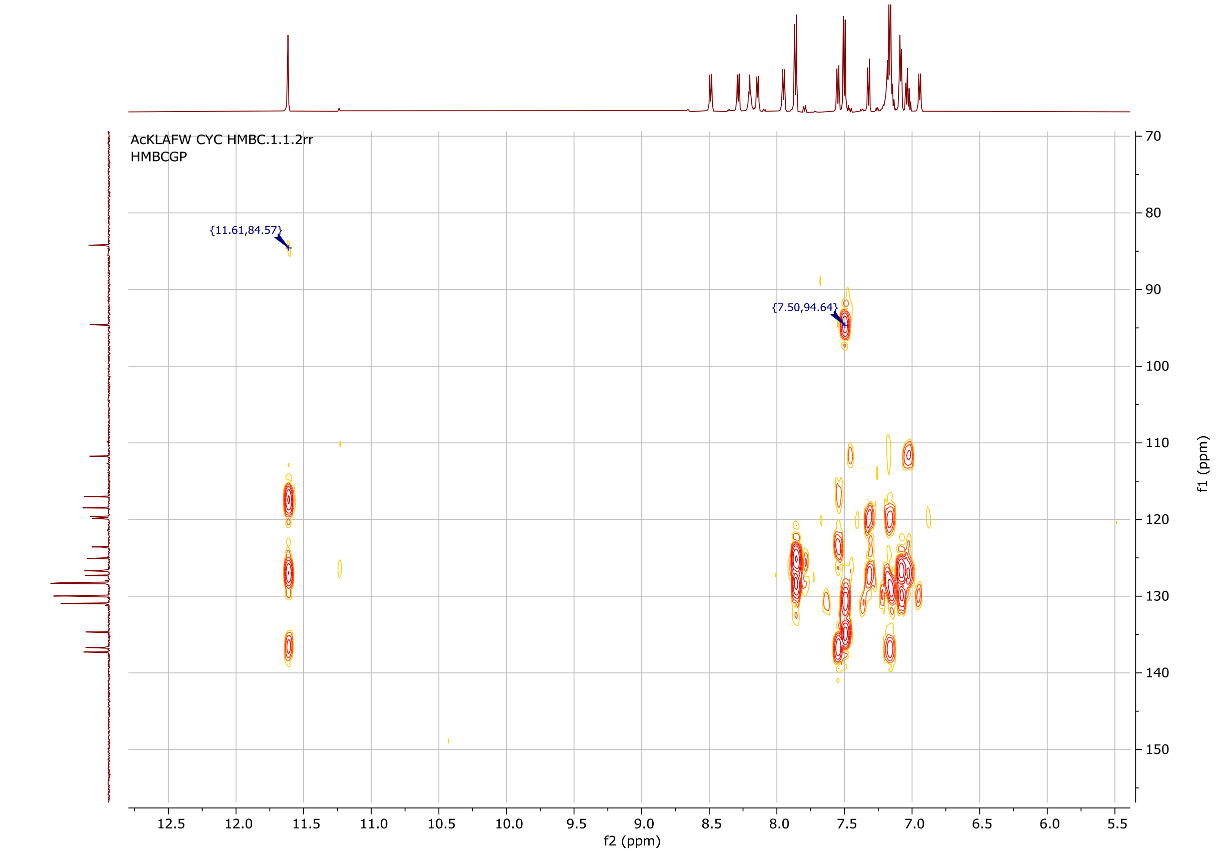

The ratio of two isomers didn't change with increasing temperature in NMR.

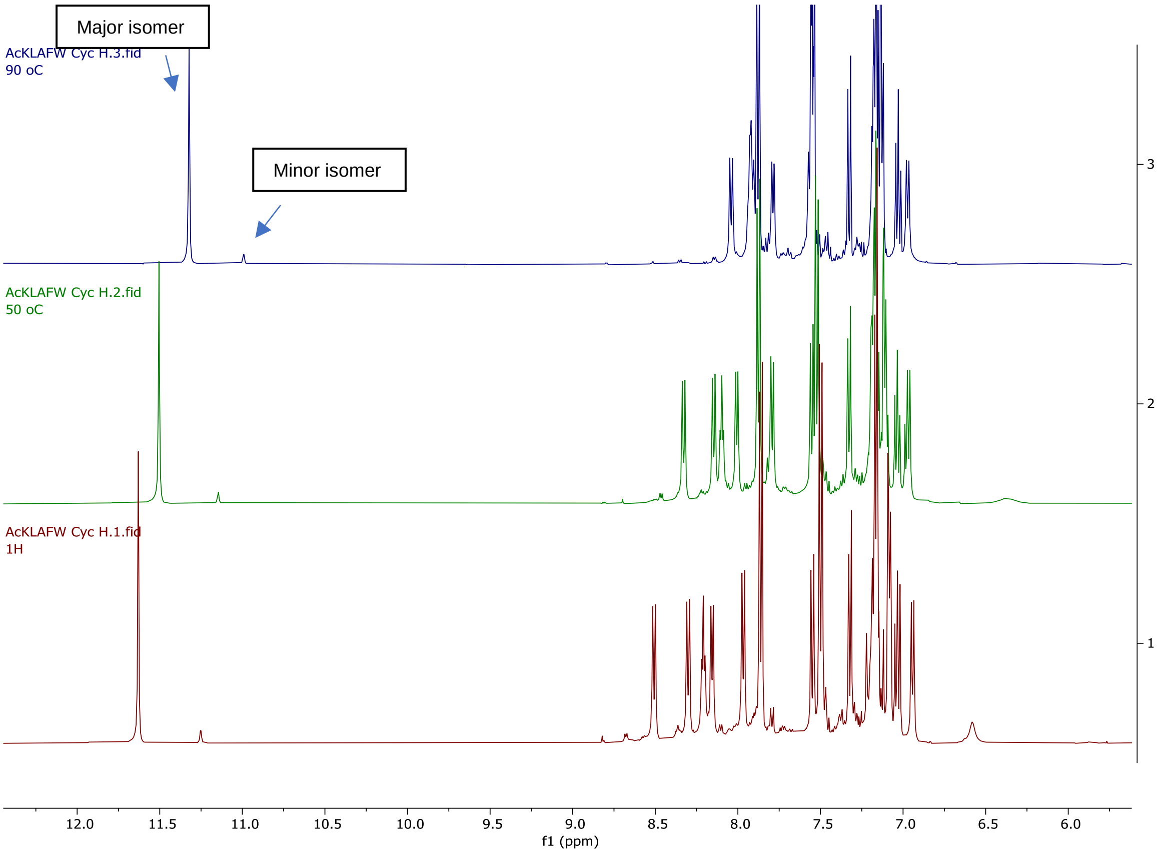

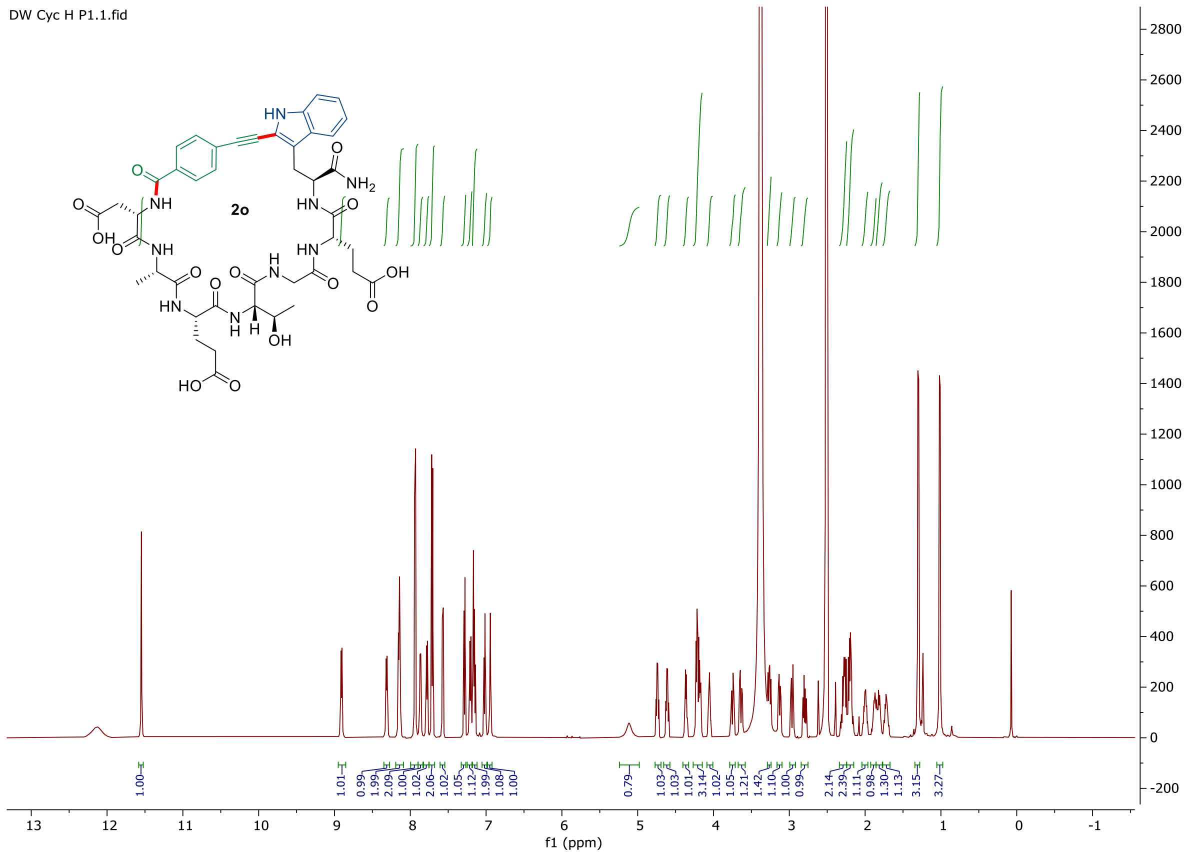
DW Cyc H P1.1.fid
2o
f1 (ppm)

HSQC

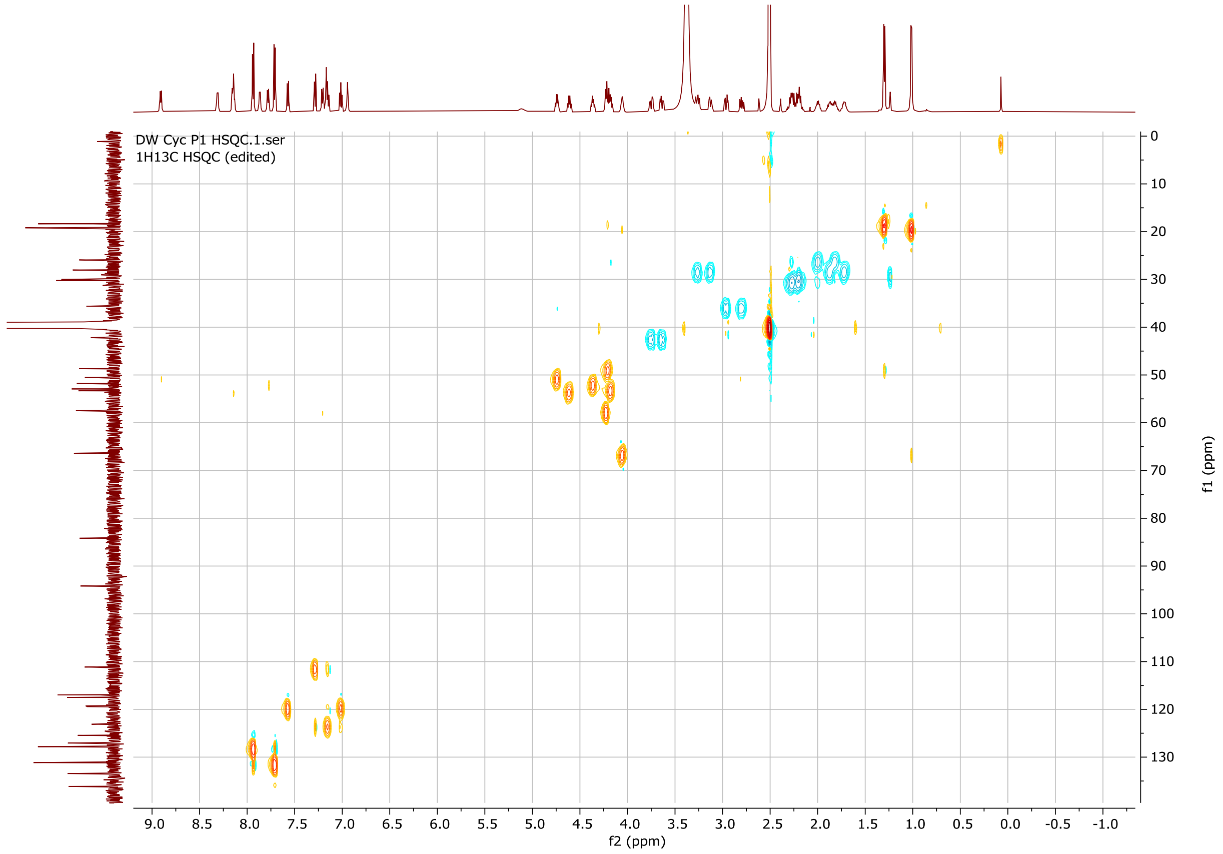
DW Cyc P1 HSQC.1.ser
1H13C HSQC (edited)
f2 (ppm)
f1 (ppm)

HMBC

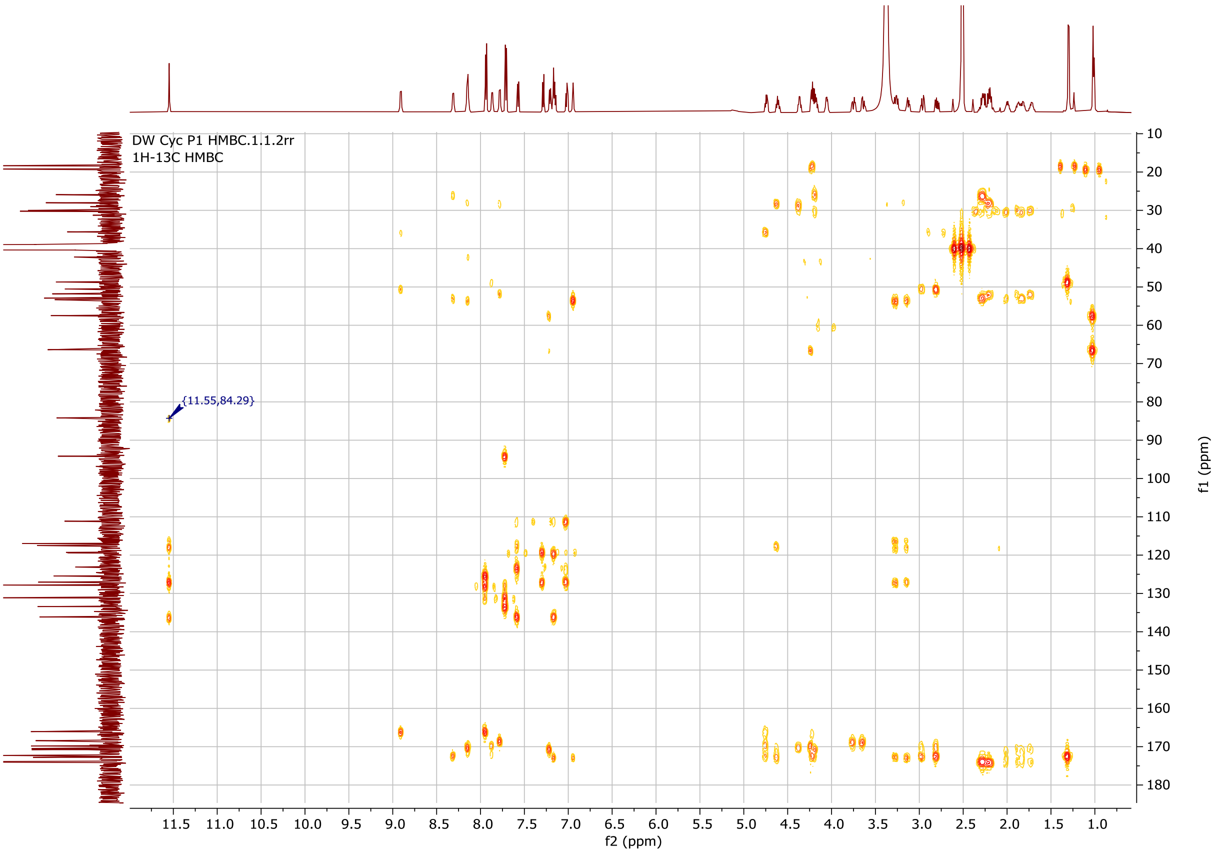
DW Cyc P1 HMBC.1.1.2rr
1H-13C HMBC
{11.55,84.29}
f1 (ppm)
f2 (ppm)

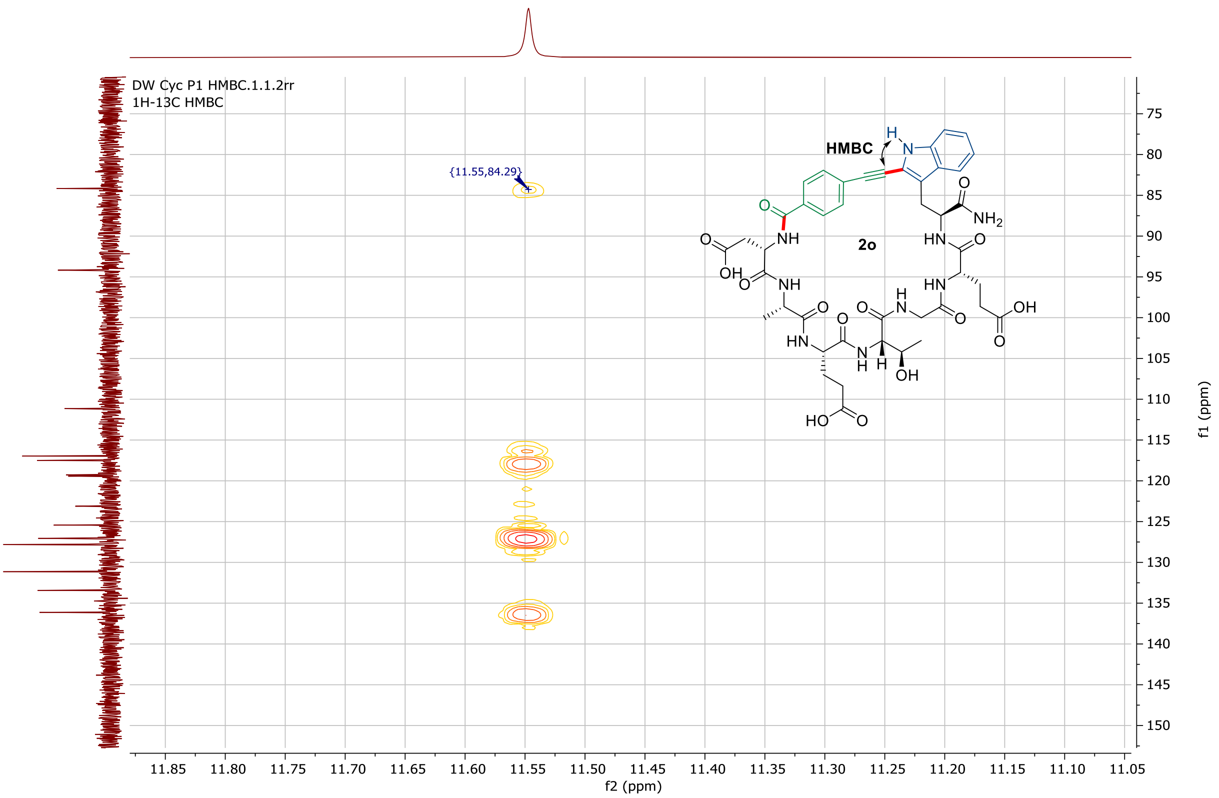
DW Cyc P1 HMBC.1.1.2rr
1H-13C HMBC
{11.55,84.29}
HMBC
2o
f1 (ppm)
f2 (ppm)

HMBC spectrum shows the weak interaction between alkyne and indole N-H, confirming the **2p** is Trp C-2 alkynylation product.

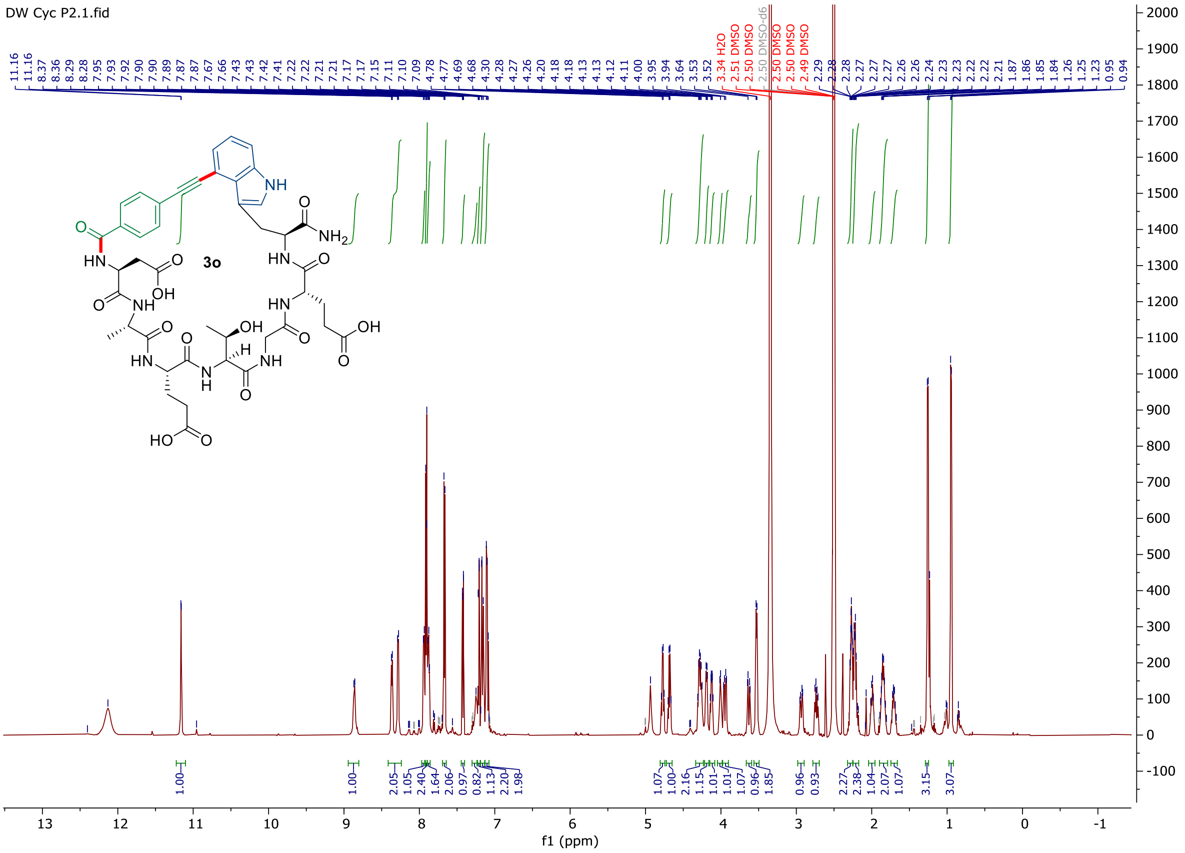

HSQC

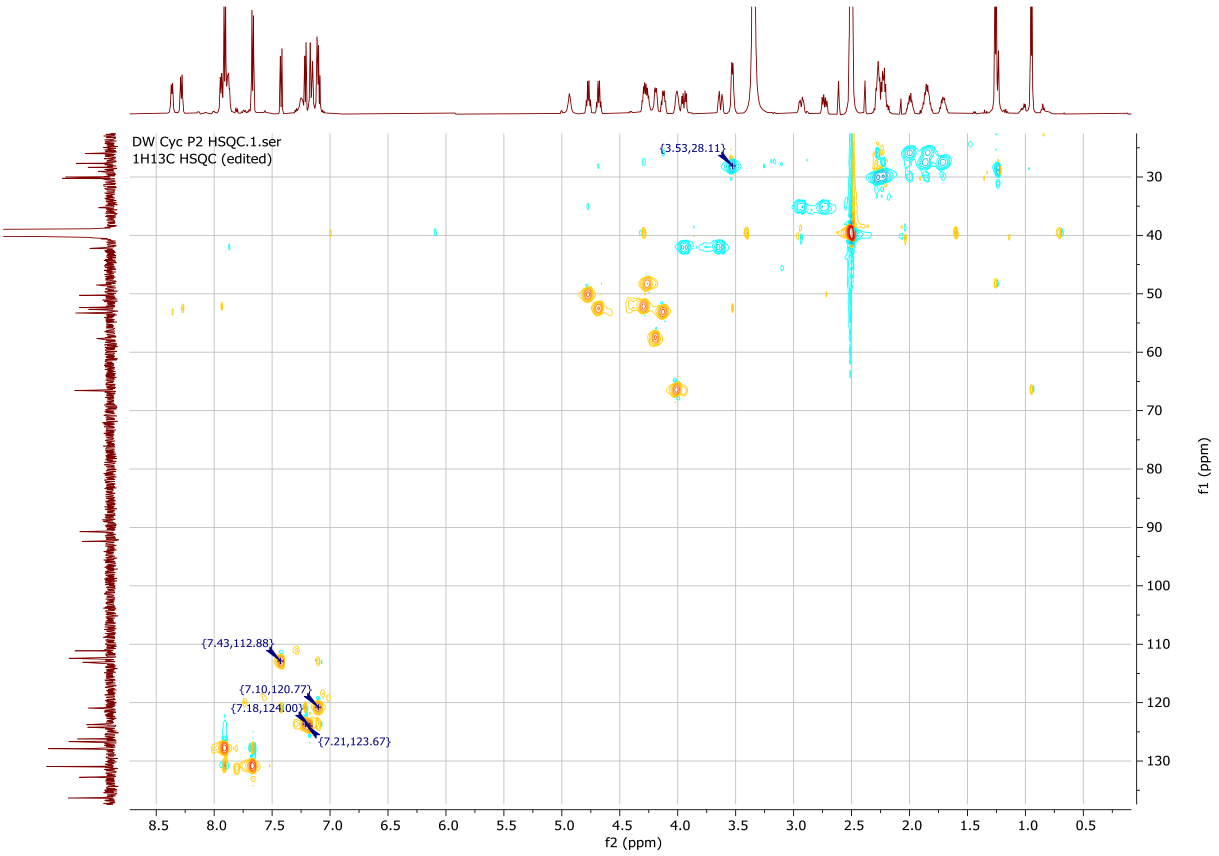
DW Cyc P2 HSQC.1.ser
1H13C HSQC (edited)
{3.53,28.11}
{7.43,112.88}
{7.10,120.77}
{7.18,124.00}
{7.21,123.67}
f2 (ppm)
f1 (ppm)

HMBC

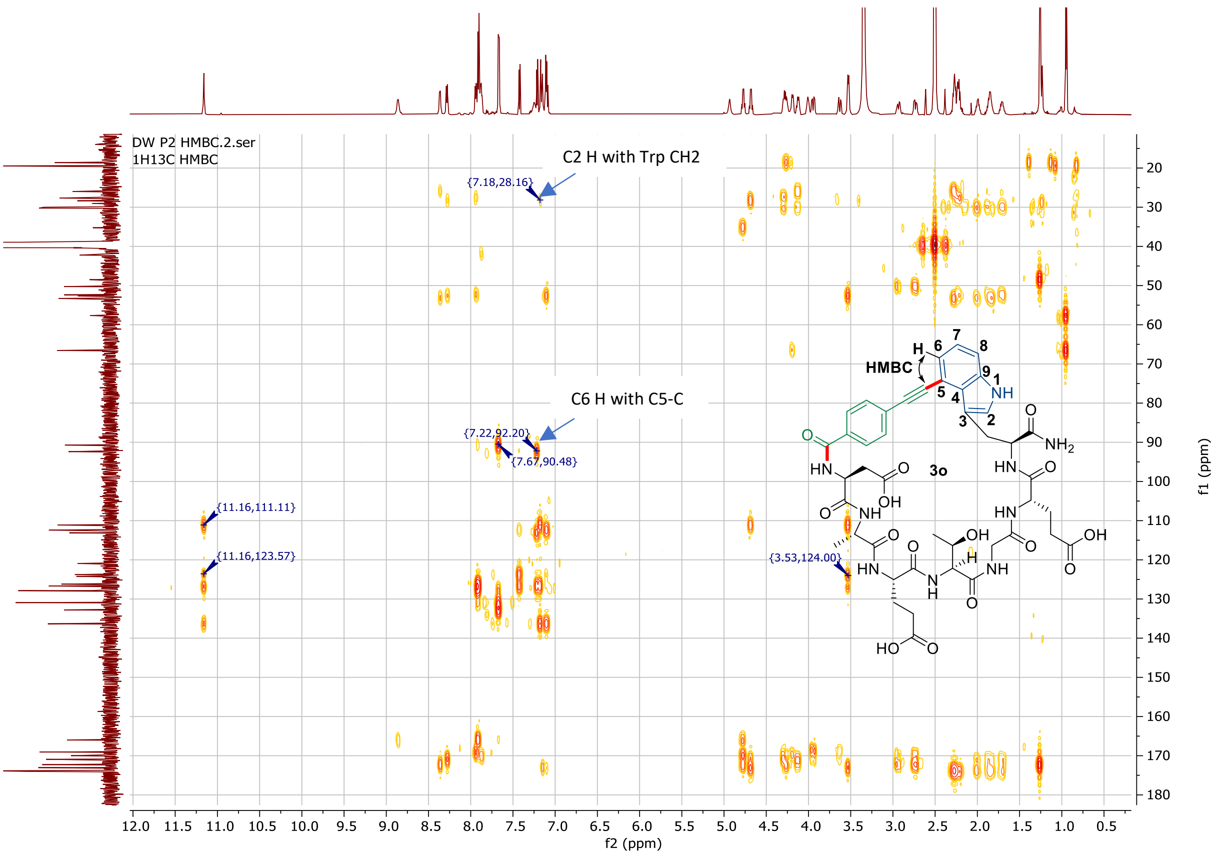
DW P2 HMBC.2.ser
1H13C HMBC
C2 H with Trp CH2
{7.18,28.16}
C6 H with C5-C
{7.22,92.20}
{7.67,90.48}
{11.16,111.11}
{11.16,123.57}
{3.53,124.00}
HMBC
3o
f2 (ppm)
f1 (ppm)

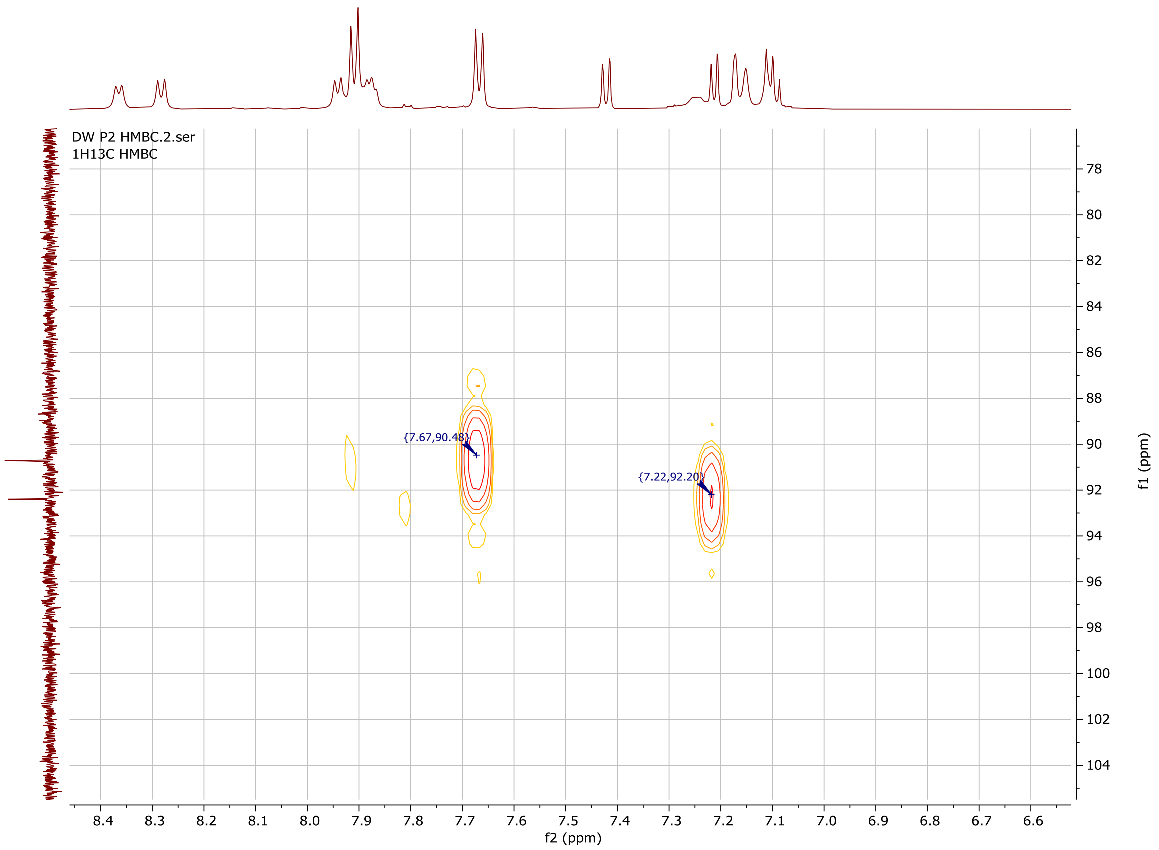

HMBC spectrum shows NO interaction between alkyne and indole N-H. Strong interaction between alkyne C and C6-H.

NOE

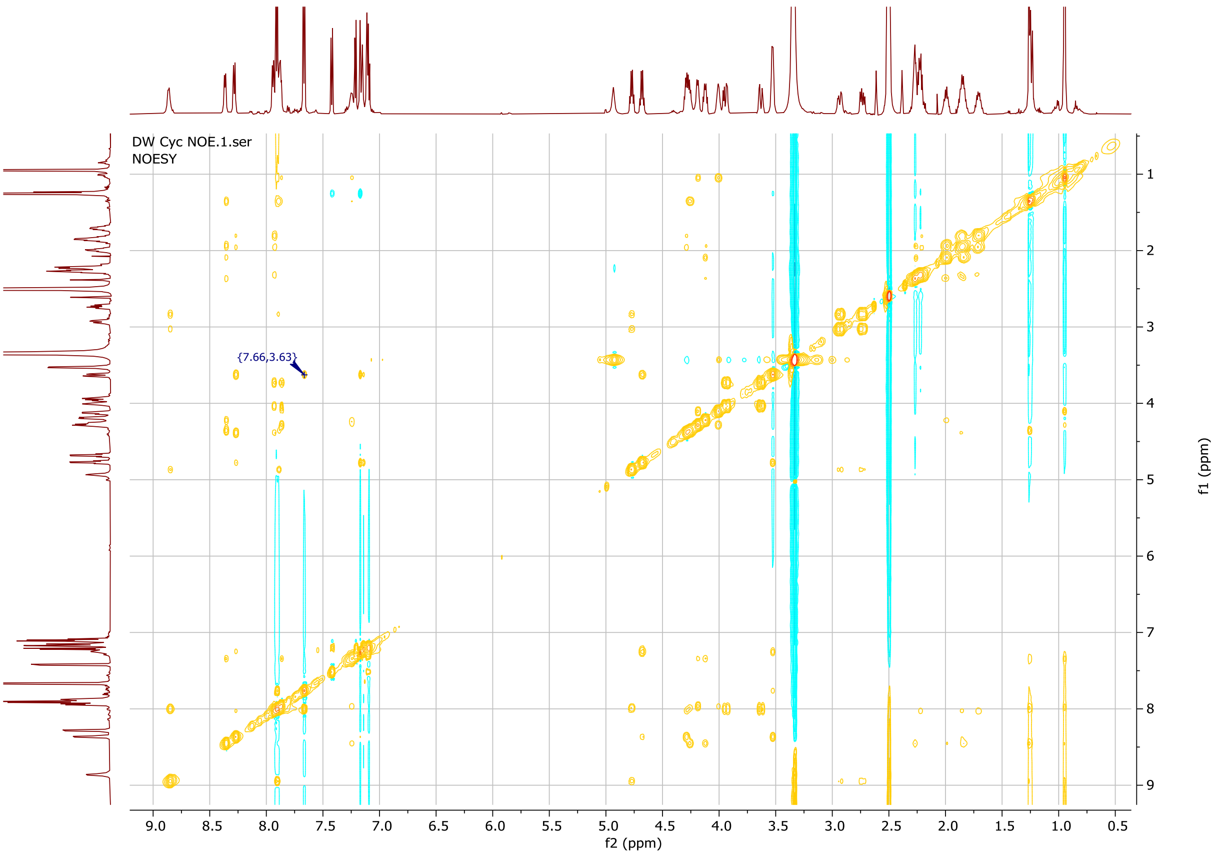

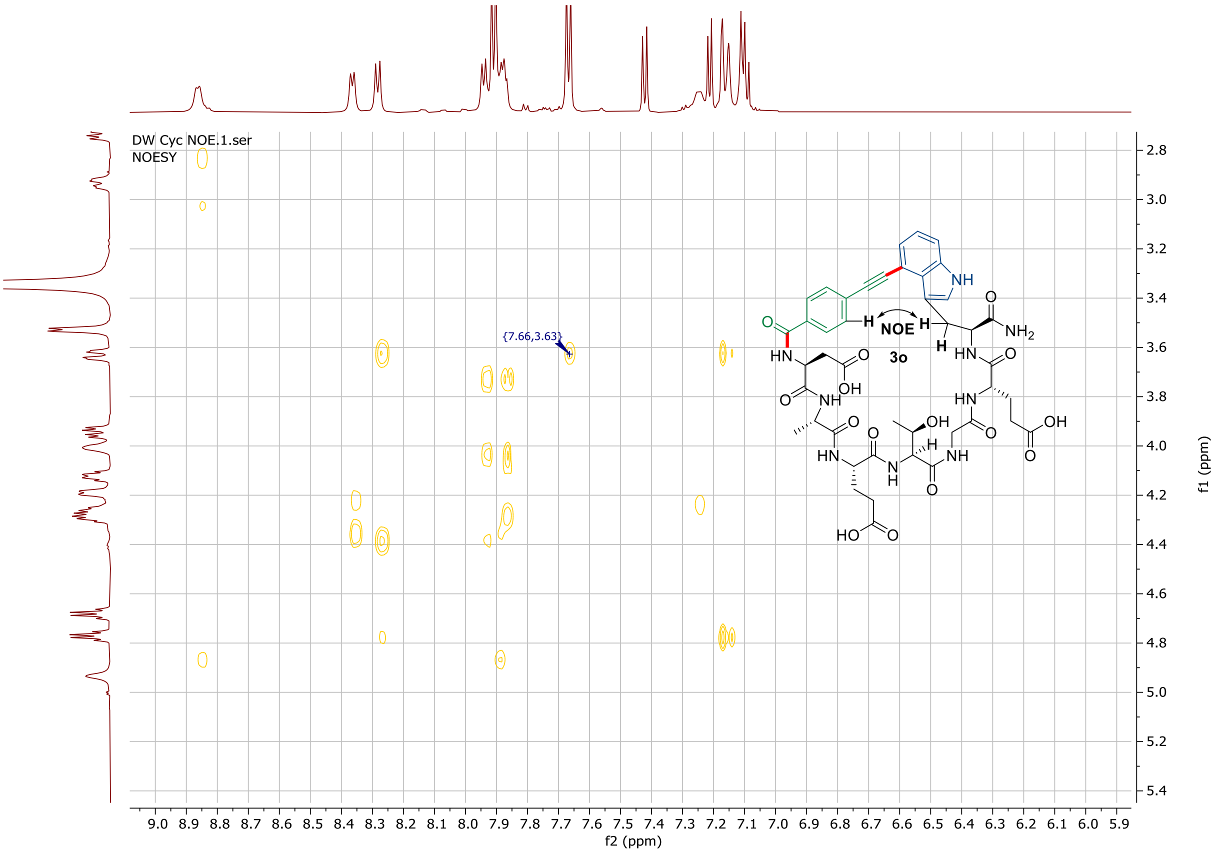

NOE spectrum observed the correlation between proton on phenylacetylene and Trp CH2

7.2 Cyclization under different reaction temperature:

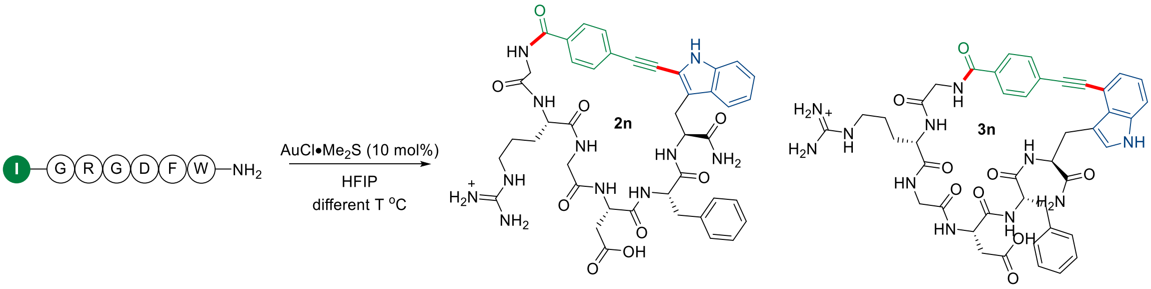

0 ºC: HPLC-UV ratio of **2n**:**3n**:dimer = 7.7: 90.5: 1.7

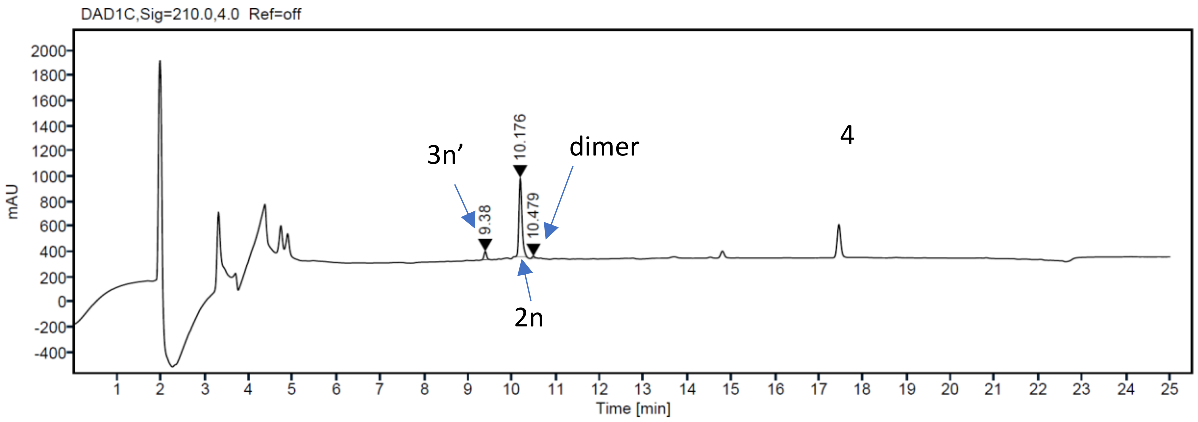

RT: HPLC-UV ratio of **2m**:**3m**:dimer = 6.1: 92.0 : 1.8

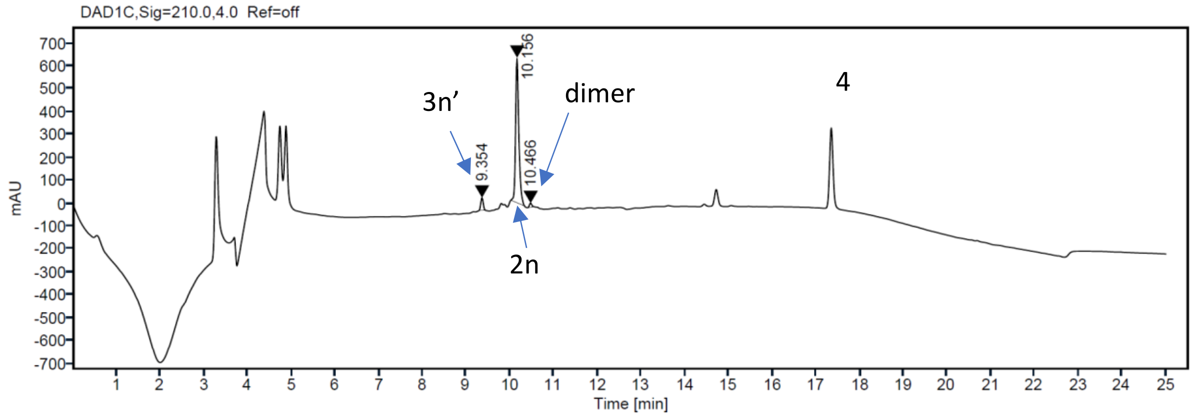

40 ºC: HPLC-UV ratio of **2n**:**3n**:dimer = 6.3: 92.0 : 1.2

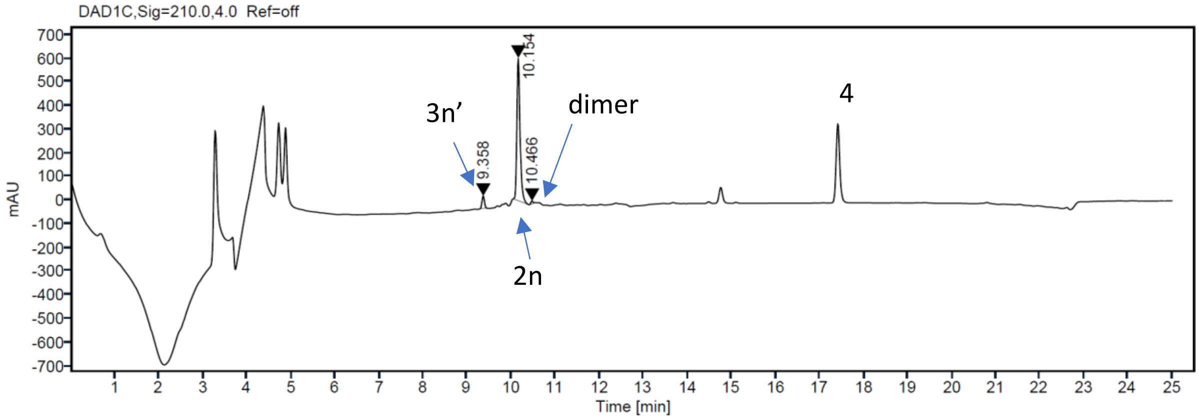

8. Absorption and emission of cyclic peptides

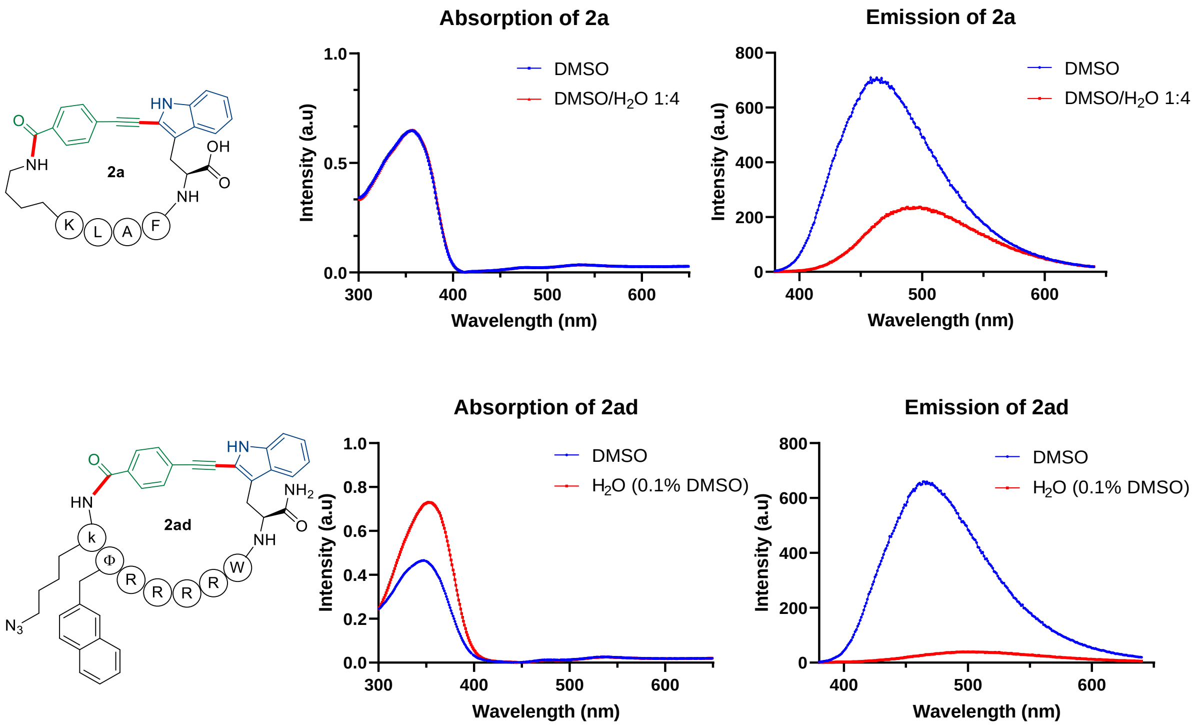

Figure S3 Absorption and emission of cyclic peptide **2a** and **2ad** in DMSO and DMSO/H_2O (20 μM). Emission curve was recorded under the excitation of 350 nm light.

Due to the poor solubility of **2a** in water, DMSO/H_2O 1:4 was used for the absorption and emission measurement.

9. Excited state lifetime of 2ab

Sample preparation: Two glass coverslip (#1.5, 170 µm thick) cleaned in 1% Hellmanex solution, rinsed in deionized water and oxygen plasma-treated (100W, 30 sccm, 90s). A 120 µm thick Secure-Seal imaging spacer with a ~1cm diameter aperture was used to define a chamber between the coverslips, in which 20 µL of 20 mM **2ab** in deionized water/DMSO 9:1 was placed.

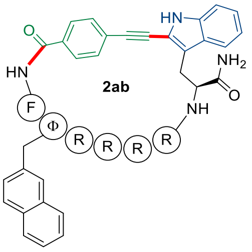

Excitation: SuperK Fianium FIU-15 at 100% power, 38.7 MHz repetition rate, 390-400 nm window filtered by SuperK Varia tunable filter, power ~1.7 mW focused to a ~50µm diameter circle. In these conditions, the pulse FWHM is about 50 ps as specified by the manufacturer.

Optics: Semrock (Laser-Beamsplitter HC R488 1 lambda PV flat, 488 LP Edge Basic Longpass Filter). Olympus UPLANSApo 60X WI 1.20 NA objective mounted on an inverted Olympus IX-71 microscope.

Detection: Pi Imaging SPAD 512^2 placed at the image plane operating voltage 26V, 95.6 ps gate steps, gate width ~8 ns, 50 ms integration time per frame. The signal was integrated from a 128x128 pixel region. Assuming a depth of field of 1 µm for the imaging system, this corresponds to a probing volume of ~30 µm x 30 µm x 1 µm. SPAD cameras, due to imperfect fabrication process, exhibit hot pixels (~5% of the total) which we filtered out. A cutoff dark count rate of 15 cps was used for this hot pixel removal.

Figure S4: The IRF (in blue) was acquired as the reflection of the beam on the sample using a 50:50 beam splitter (BSW10R, Thorlabs) and attenuation with an adjustable ND filter in order not to saturate the camera while still using the laser at full power to preserve the pulse shape. The time reference for this graph is arbitrary. The measured decay with the 488 nm filter set showed a longer tail corresponding to the fluorescence of the compound (in red). In order to simplify the analysis of the red curve which is the convolution of the IRF with the time response of the sample, we use the approach of directly fitting the part of the fluorescence decay where the IRF has decayed to very low values (highlighted in red).[4] We set the cutoff value for this approach as the dotted vertical line, where the IRF has decayed to 4‰.

[4] Liu, X.; Lin, D.; Becker, W.; Niu, J.; Yu, B.; Liu, L.; Qu, J., Fast fluorescence lifetime imaging techniques: A review on challenge and development. *J. Innovat. Opt. Health. Sci.* **2019,** *12* (05), 1930003.

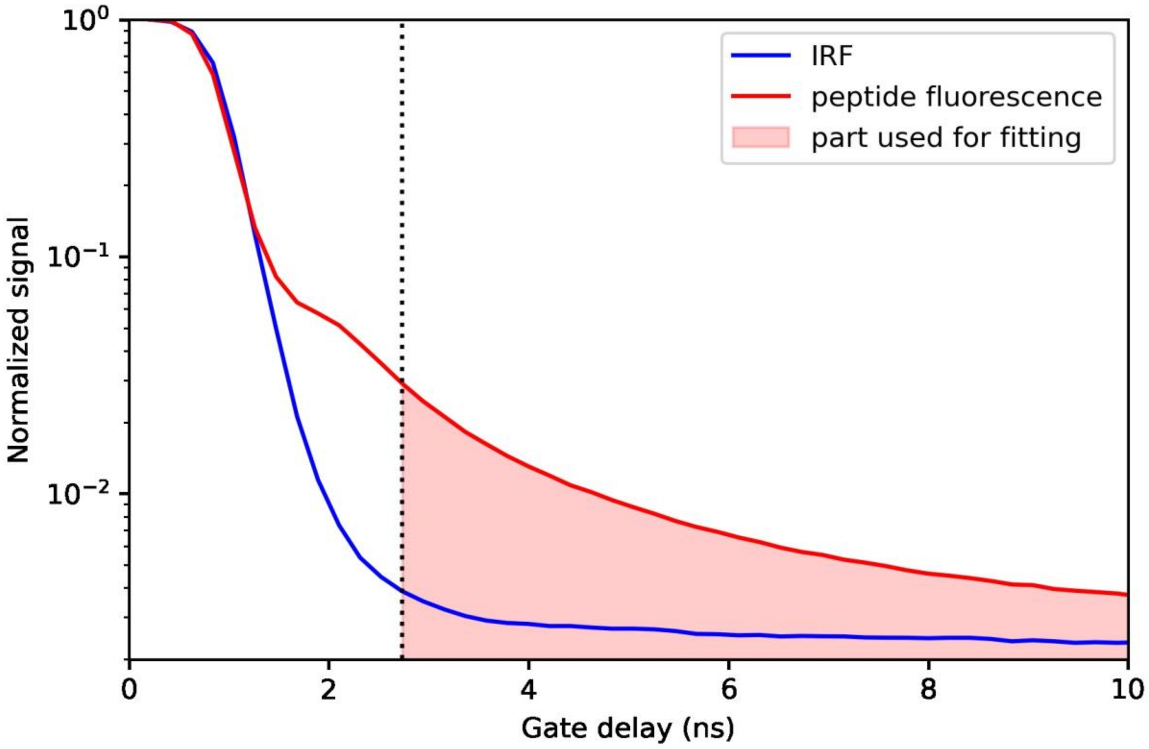

Figure S5: Least-square fitting of the exponential decay (black dots) with a mono (blue) or bi-exponential (red) model with background. The fit is near-perfect for a bi-exponential model, but not for a mono-exponential model.

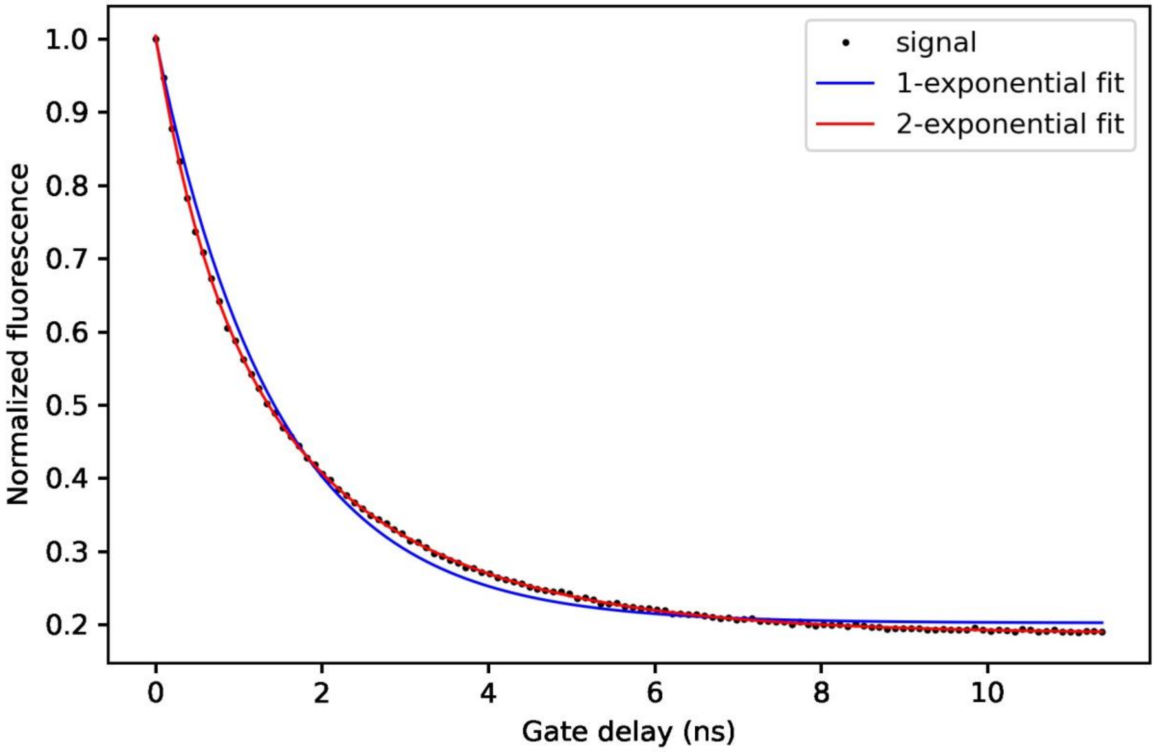

Figure S6: Visualizing the goodness of the fit in the previous figure with a semi-logarithmic plot. The background value obtained from fitting was removed to visualize the exponential decay.

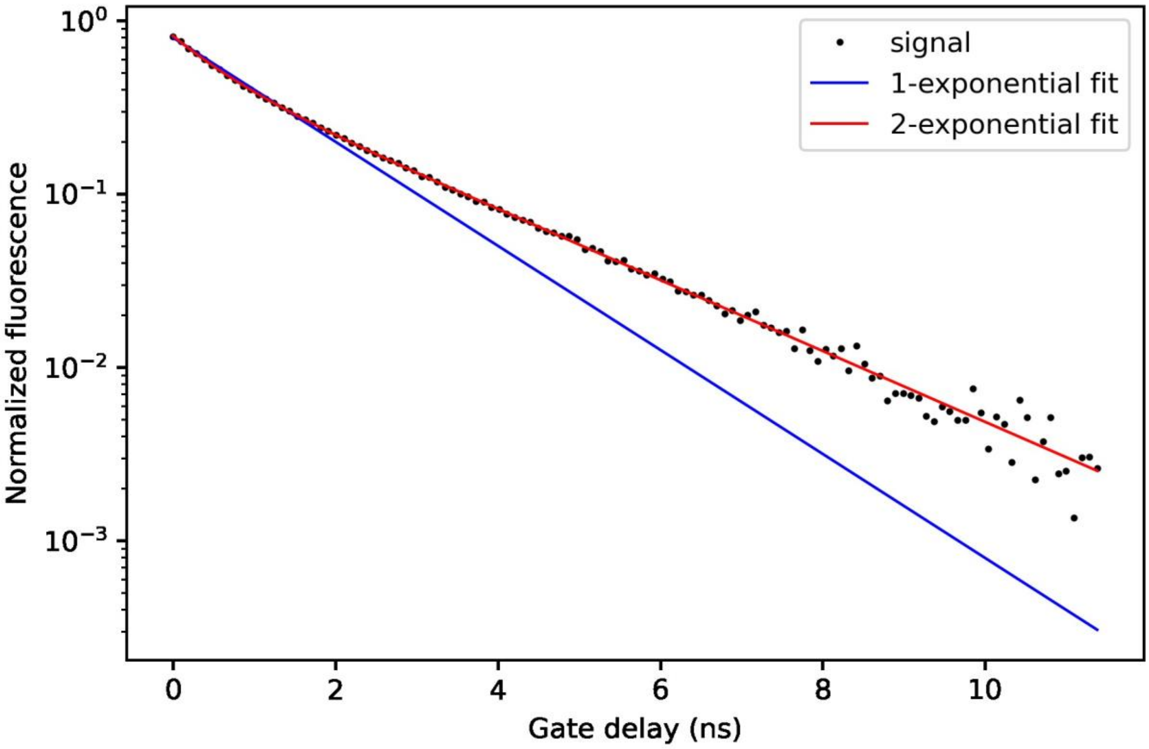

Fit results:

τ_1=2.12 ±0.02 ns with amplitude 0.54±0.01

τ_2=0.60 ±0.02 ns with amplitude 0.28±0.01

background: 0.19

The second lifetime component could be due to the high concentration used.[5,6] This was however necessary for robust lifetime readout, as the extinction coefficient is low, and the excitation is off-resonance.

[5] Chen, W.; Young, L. J.; Lu, M.; Zaccone, A.; Ströhl, F.; Yu, N.; Kaminski Schierle, G. S.; Kaminski, C. F., Fluorescence Self-Quenching from Reporter Dyes Informs on the Structural Properties of Amyloid Clusters Formed in Vitro and in Cells. *Nano Lett.* **2017,** *17* (1), 143-149.

[6] Quinn, S. D.; Dalgarno, P. A.; Cameron, R. T.; Hedley, G. J.; Hacker, C.; Lucocq, J. M.; Baillie, G. S.; Samuel, I. D. W.; Penedo, J. C., Real-time probing of β-amyloid self-assembly and inhibition using fluorescence self-quenching between neighbouring dyes. *Mol. Biosyst.* **2014,** *10* (1), 34-44.

10. Cell experiments

Cell line stocks. Hela cell lines were obtained from the European Collection of Animal Cell Cultures (ECACC, United Kingdom) and they were grown in complete medium (DMEM) supplemented with 10% fetal bovine serum (FBS) and maintained in an incubator at 37 °C in an atmosphere of 5% CO_2. Hela cells were seeded on poly-lysine coated 8-well glass bottom cell culture plates at a density of 10’000 cells/well the day before the experiment. For the experiment, the medium was exchanged to fresh medium containing 10 μM of the indicated peptide (stock solution were prepared to a concentration of 10 mM in DMSO). Cells were incubated for 3 hours. After that, the medium was removed and cells were washed with PBS before imaging. The signal was detected using an Eclipse Ti2 microscope with a Yokogawa CUS W2 confocal spinning disk unit and equipped with a Prime 95B sCMOS camera (Photometrics). We used a 60x oil immersion objective with a N.A. of 1.40 to observe cells at 37 ^{o}C in 5% CO_2. For co-localization studies, solution of LysoTracker Red (0.1 μM) was added to the pre-washed cells in the cell culture medium and cells were incubated at 37 °C for 1 hour. For the nuclear staining, cells were incubated with 1 μM SYTO Deep Red at 37°C for 30 min prior to imaging. Imaging was performed using the following parameters: cyclic peptides (excited with a 405 nm laser and captured with a 447/60 nm bandpass filter), LysoTracker Red (excited with a 561 nm laser and captured with a 542/27 nm bandpass filter) SYTO Deep Red (excited with a 638 nm laser and captured with a 600/52 nm bandpass filter). Image analysis was performed using Fiji/ImageJ.[6] For quantitative image analysis, the following threshold were used: Cyclic peptide channel: 124, LysoTracker channel: 110, an area for analysis was selected with the “create selection” function and fluorescent intensity of the whole image was measured.

MTT assay. Cells were seeded into 96-well tissue culture plates with the cell density around 10000 cells per well, and incubated overnight. Then cells in each well were incubated with complete medium containing different concentrations of cyclic peptides. Control cells were incubated with complete medium containing 0.1% of DMSO. After 16 hours of incubation, 3-(4,5-dimethylthiazol-2-yl)-2,5-diphenyltetrazolium bromide (MTT) was added to each well (1 mg/mL of final concentration), medium containing MTT was discarded after the cells were incubated for 2 hours, the MTT precipitate was dissolved in 200 μL of DMSO for each well and mixed thoroughly using the pipette. Absorbance was measured at 570 nm using the microplate reader. The absorbance of each sample was normalized with its untreated control($A_{\text{treated cells}} / A_{\text{blank}}$) × 100%.

[6] Schindelin, J.; Arganda-Carreras, I.; Frise, E.; Kaynig, V.; Longair, M.; Pietzsch, T.; Preibisch, S.; Rueden, C.; Saalfeld, S.; Schmid, B.; Tinevez, J.-Y.; White, D. J.; Hartenstein, V.; Eliceiri, K.; Tomancak, P.; Cardona, A., Fiji: an open-source platform for biological-image analysis. *Nat. Meth.* **2012,** *9* (7), 676-682.

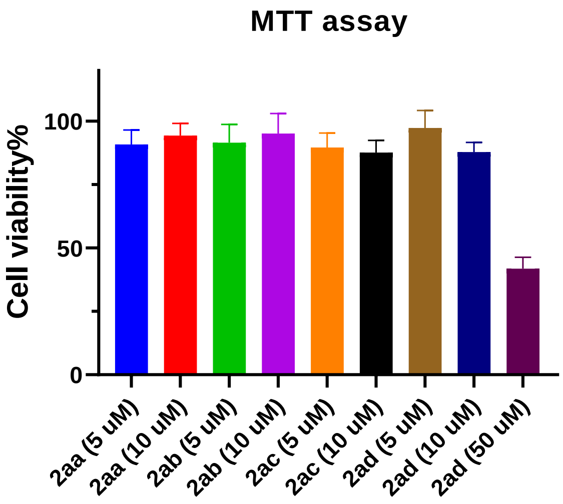

Figure S7 MTT assay**.** Cell viability were assessed by MTT assay. cells were treated with different cyclic peptides at indicated doses for 16 hours. Data shown are representative of 3 separate experiments.

Live-cell imaging:

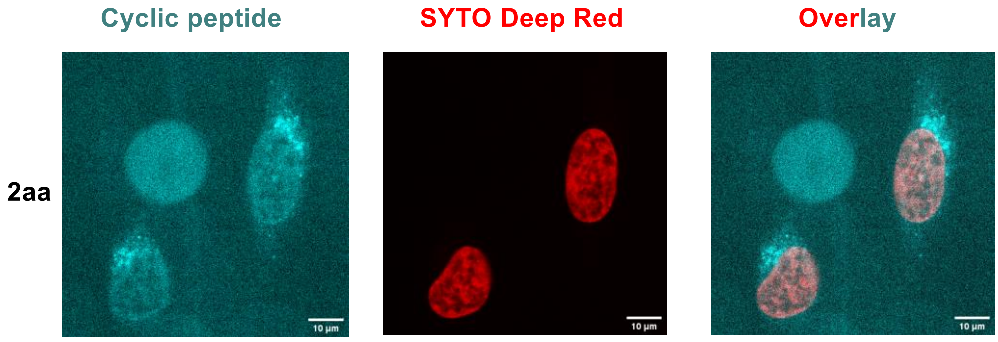

Figure S8: Live-cell images of Hela cells after 3 h incubation with 10 μM **2aa** by using confocal spinning disk microscope. Scale bar: 10 μm.

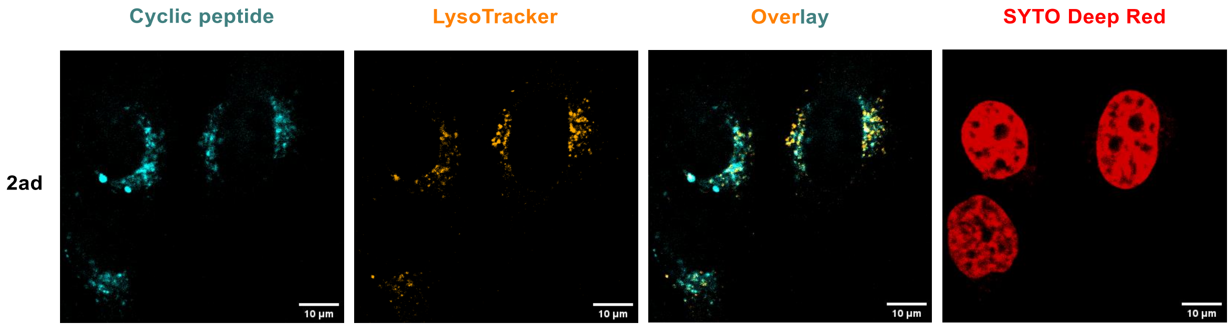

Figure S9: Intracellular distribution of 10 μM **2ad** compared to LysoTracker Red (0.1 μM) in Hela cells. The nucleus was stained by SYTO Deep Red at 1 μM. Scale bar: 10 μm.

Regions of interest (**ROI**): Cyclic peptide, Pearson's correlation coefficient (**PCC**): 0.422 Mander's overlap coefficient (**MOC**): 0.517.

11. NMR spectra

S6 ^{1}H NMR (500 MHz, $CDCl_3$)

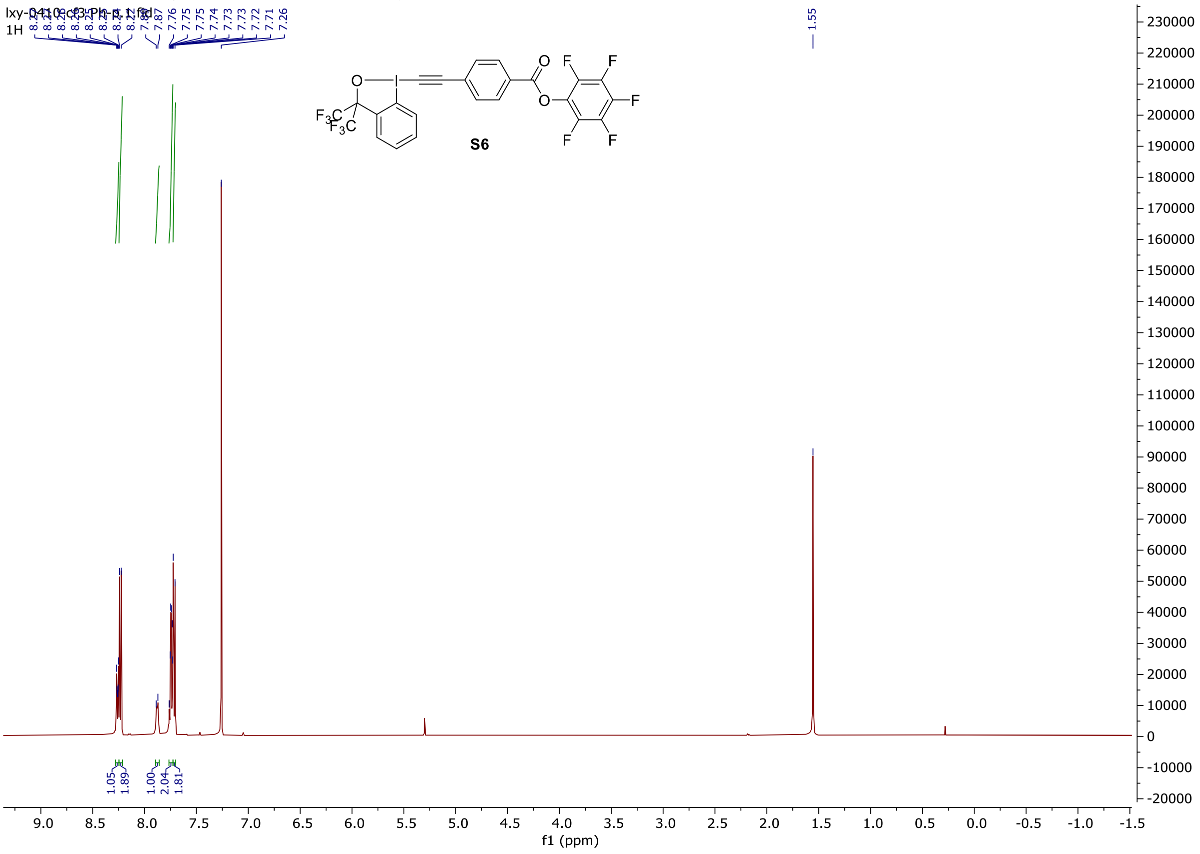

^{13}C NMR (500 MHz, $CDCl_3$)

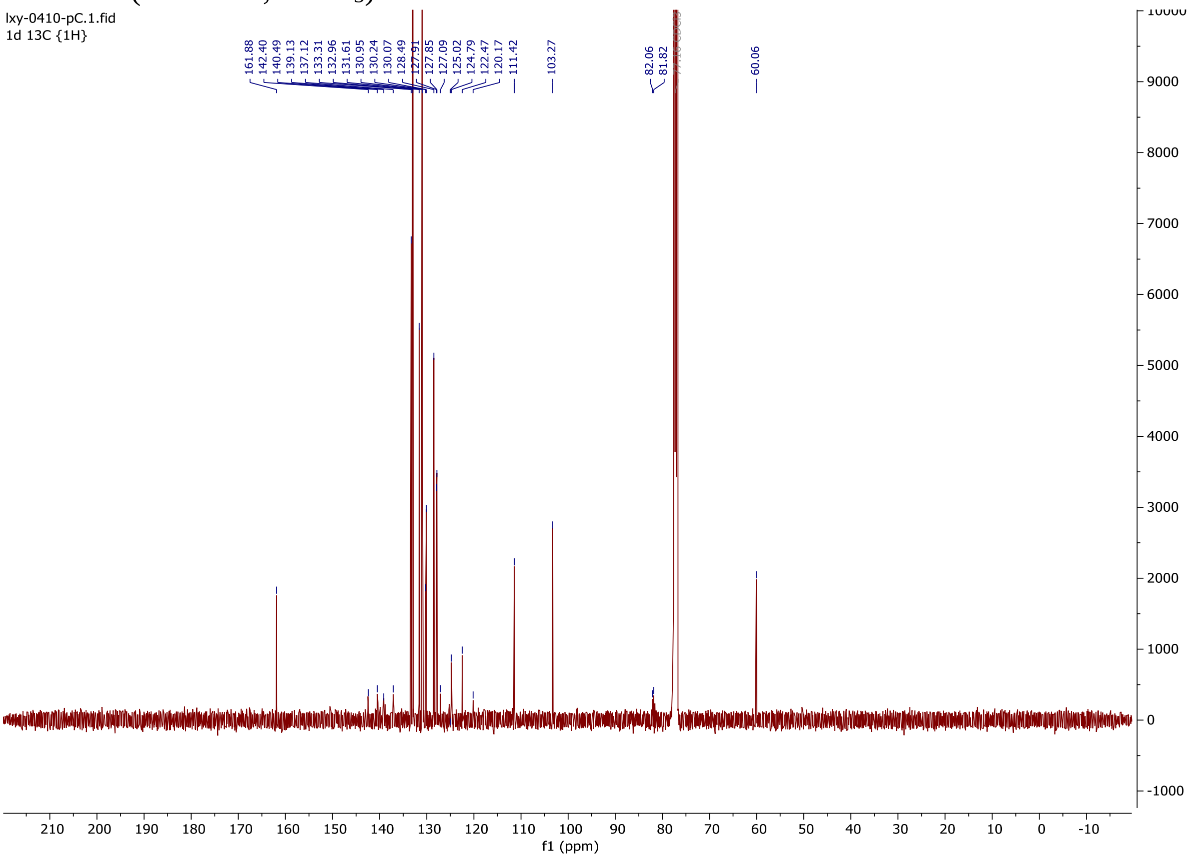

1a ^{1}H NMR (500 MHz, CD_3OD)

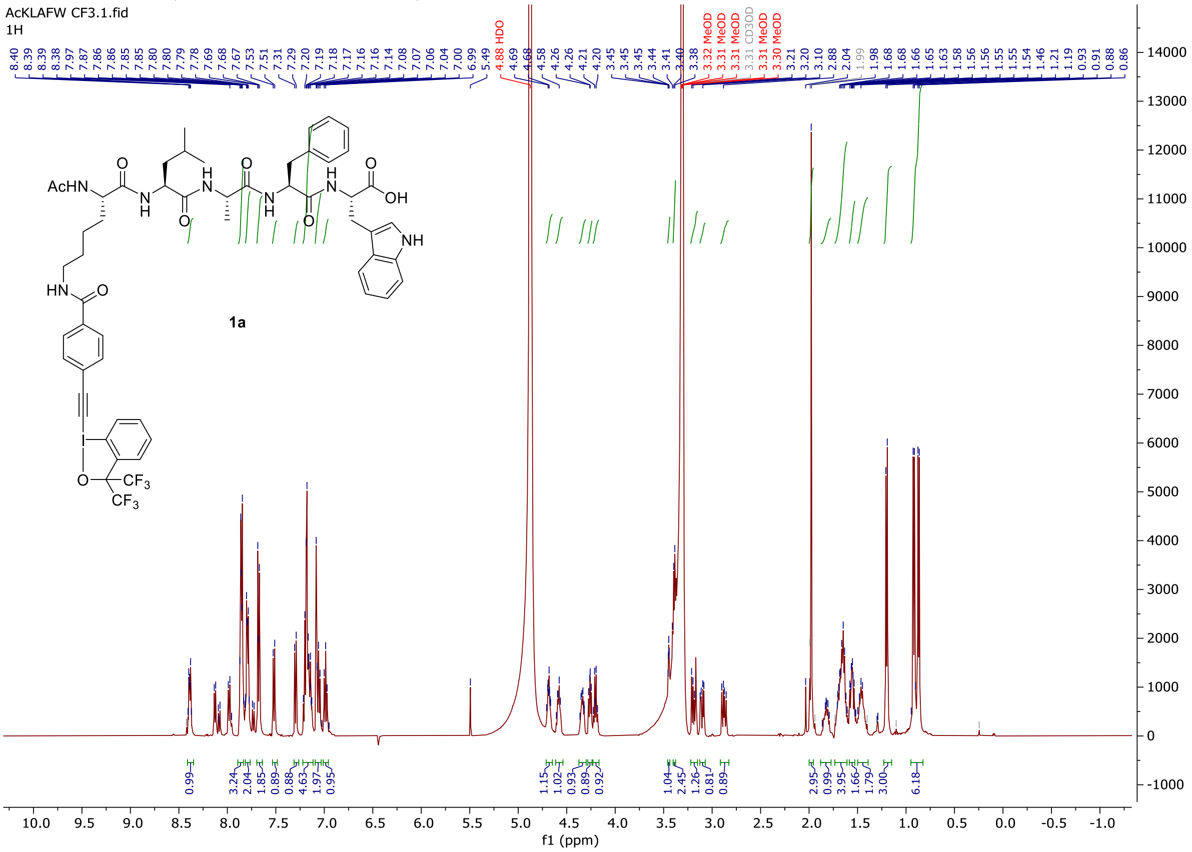

^{13}C NMR (500 MHz, CD_3OD)

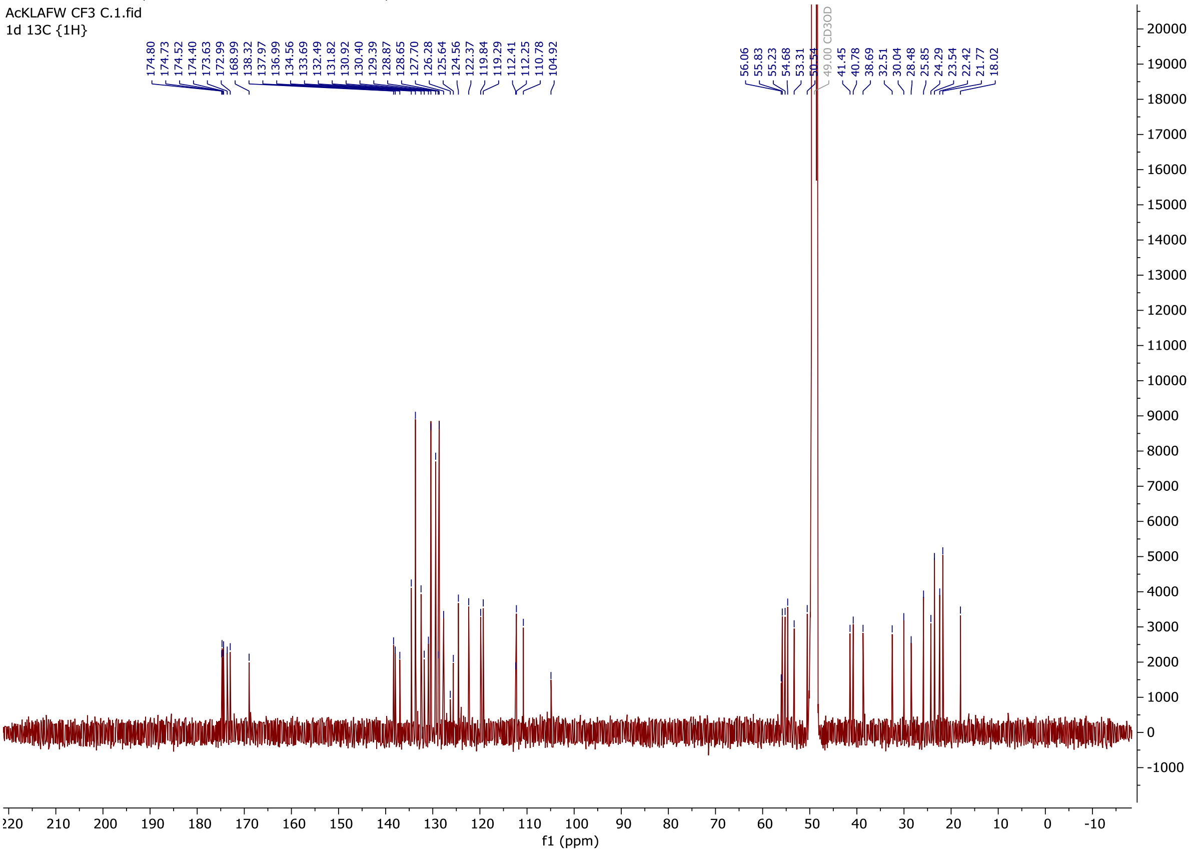

HMBC

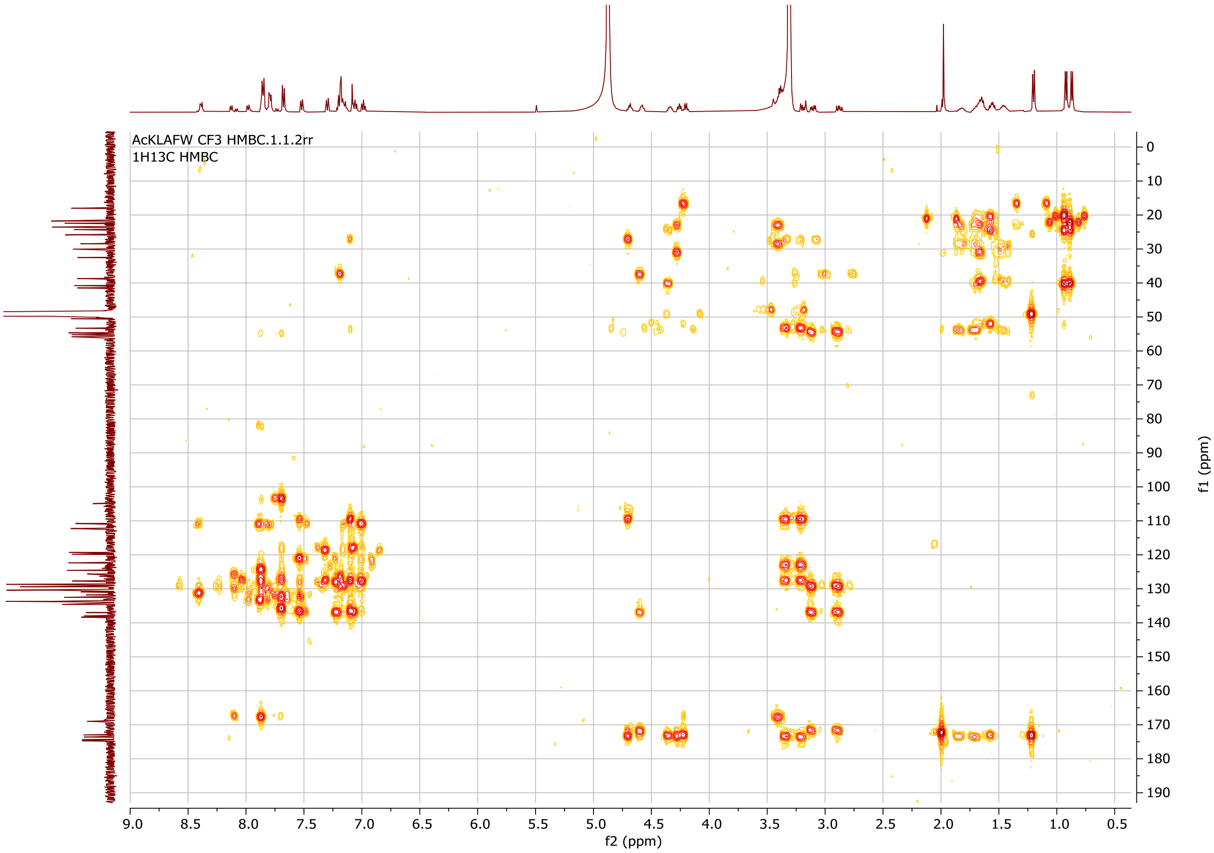

2a ^{1}H NMR (500 MHz, DMSO-d$_6$)

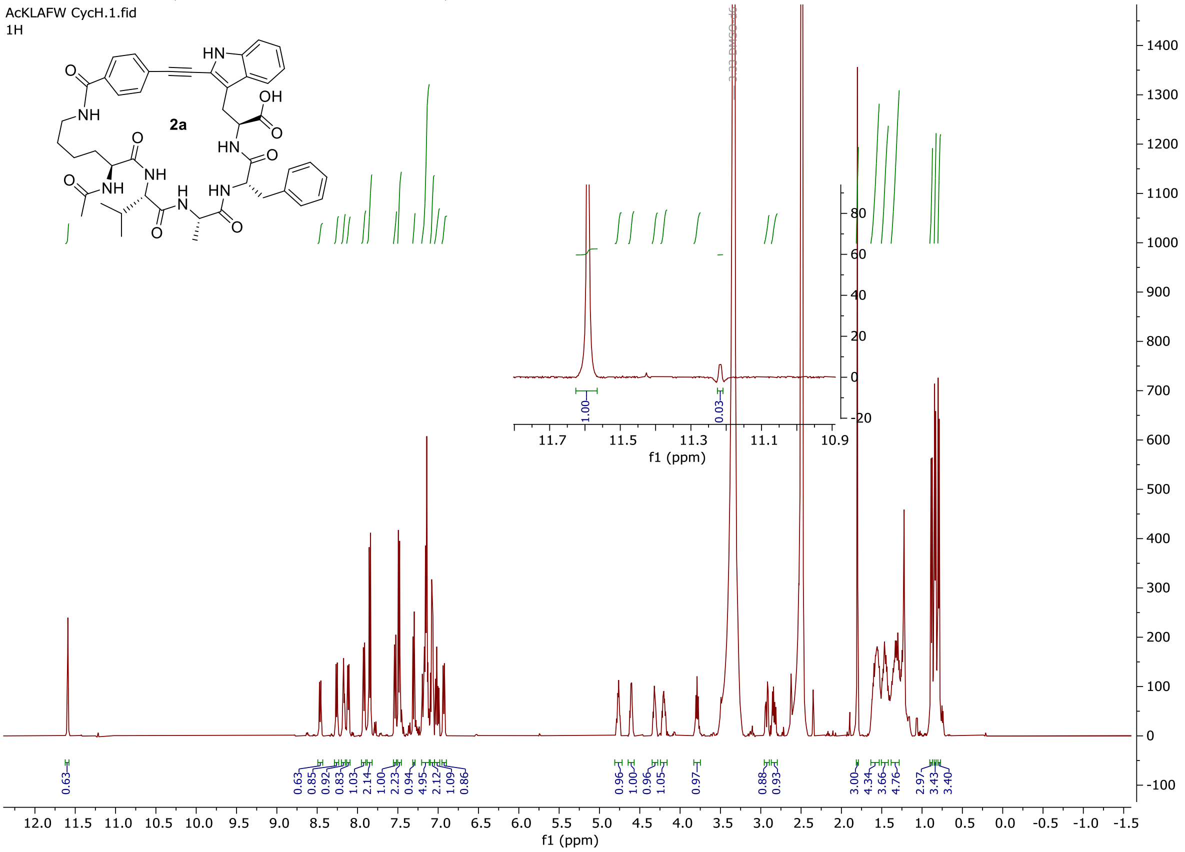

^{13}C NMR (125 MHz, DMSO-d$_6$)

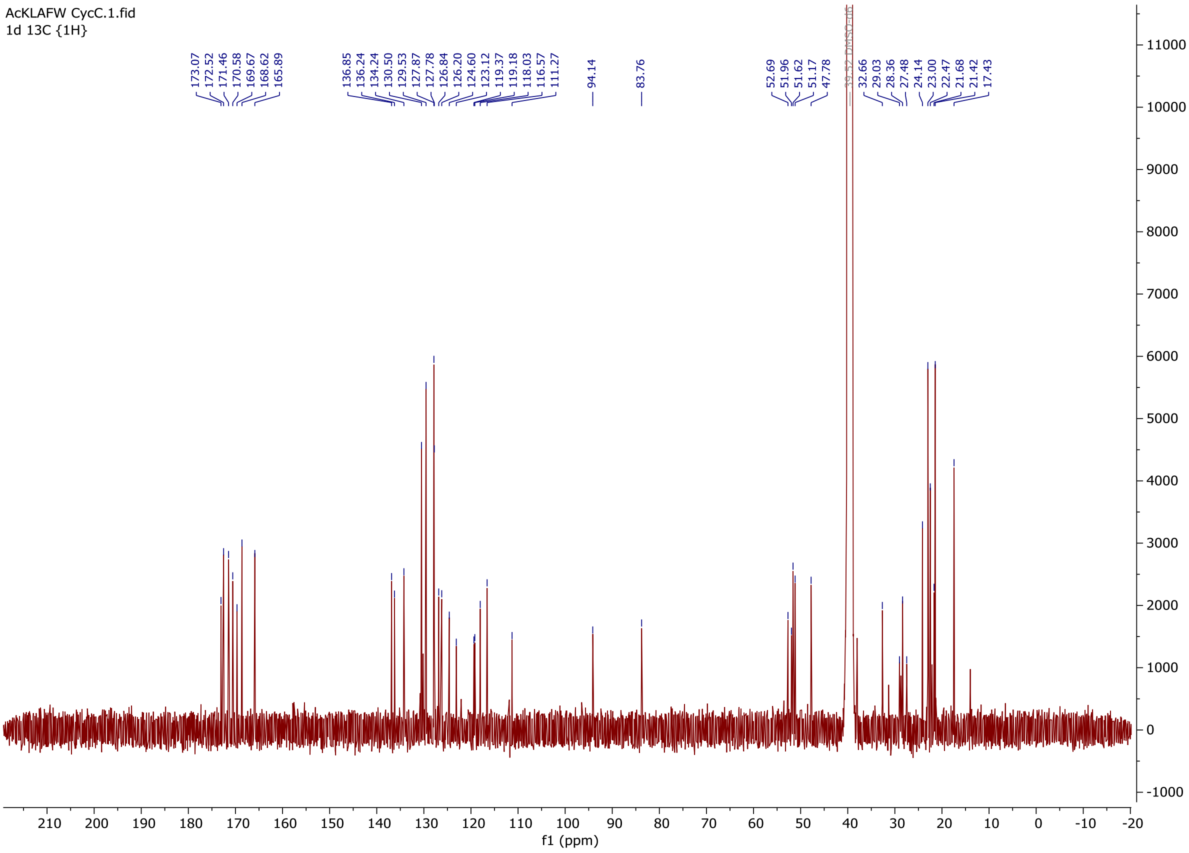

HMBC spectrum shows the weak interaction between alkyne and indole N-H, confirming the major product is Trp C-2 alkynylation product.

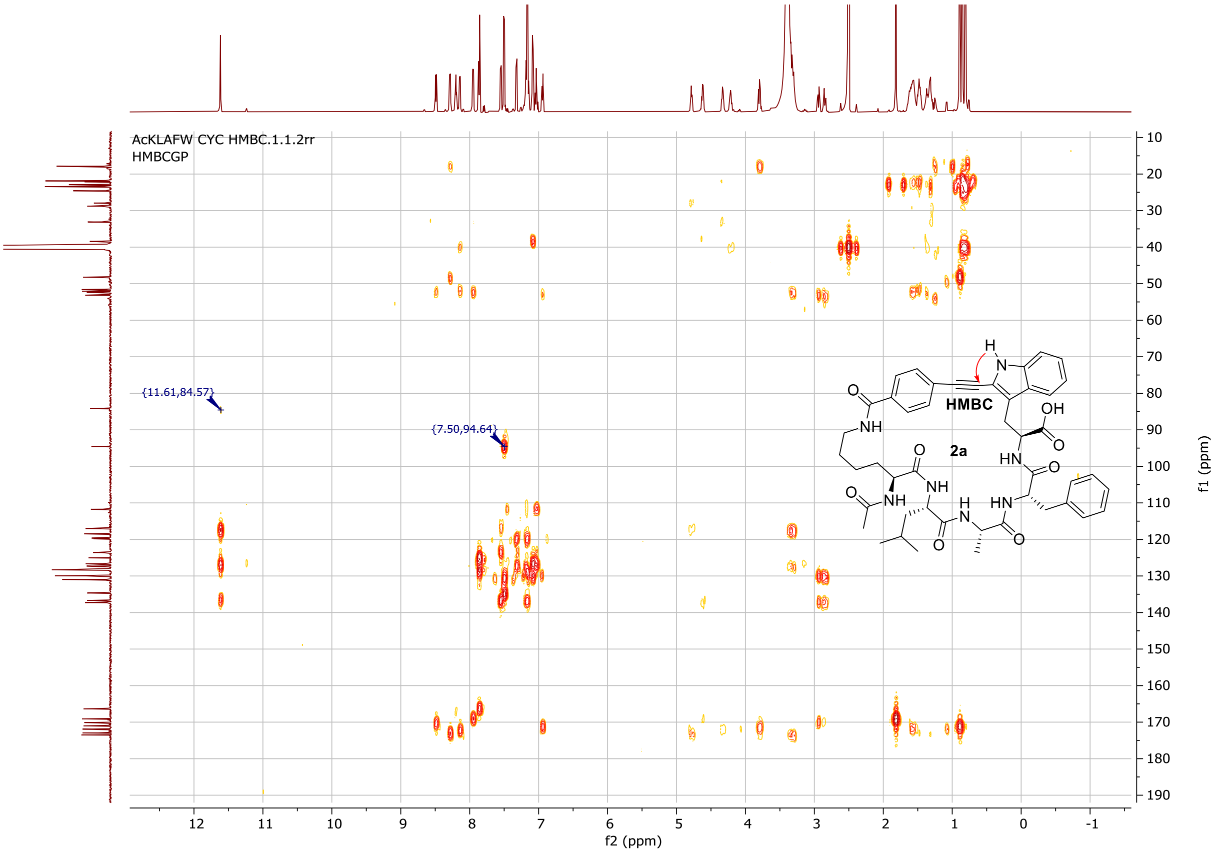

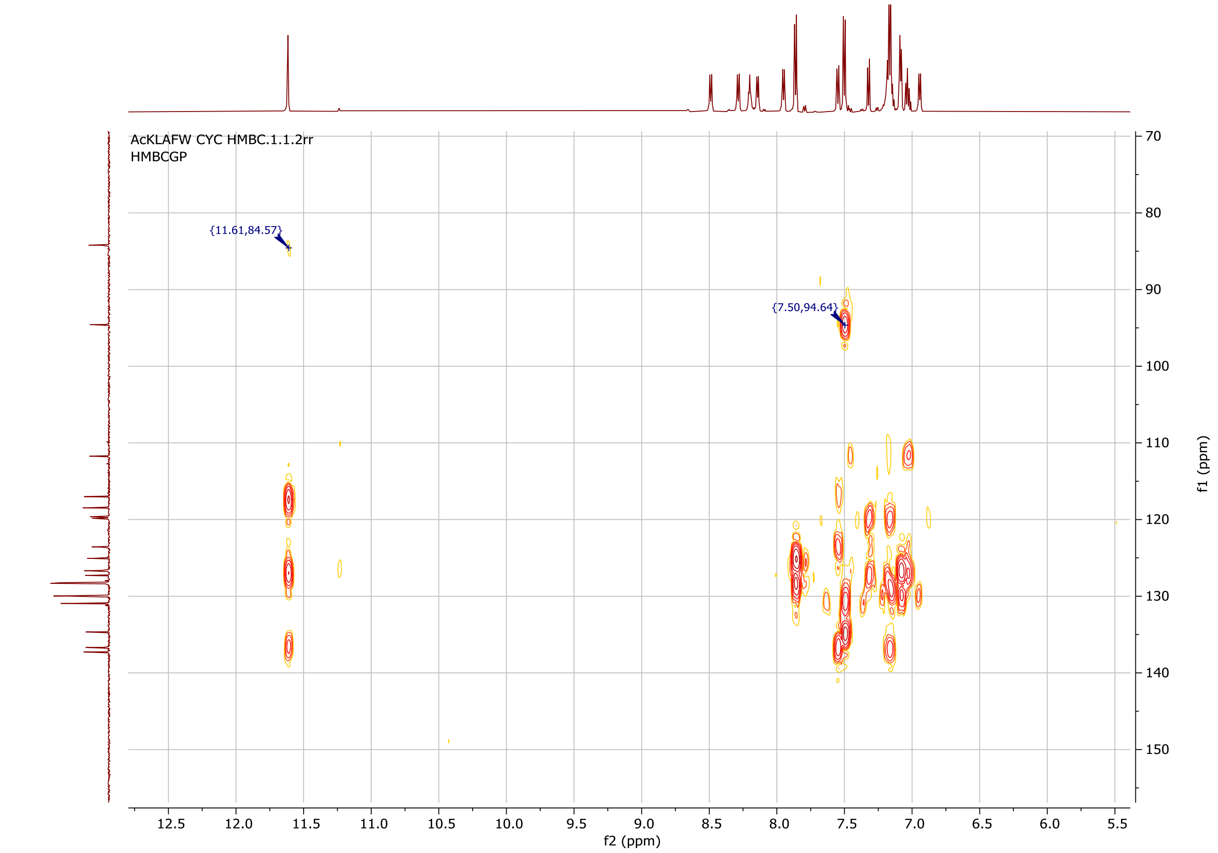

2k ^{1}H NMR (600 MHz, DMSO-d$_6$)

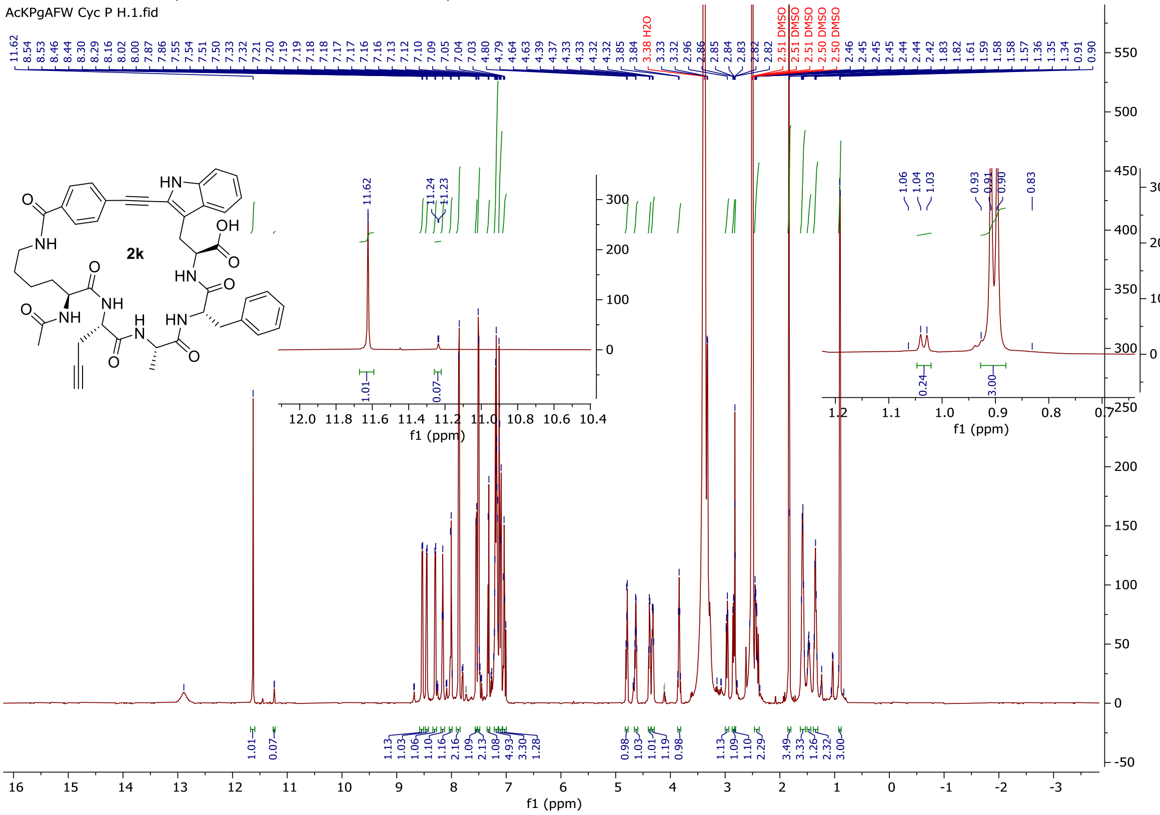

2k ^{13}C NMR (150 MHz, DMSO-d$_6$)

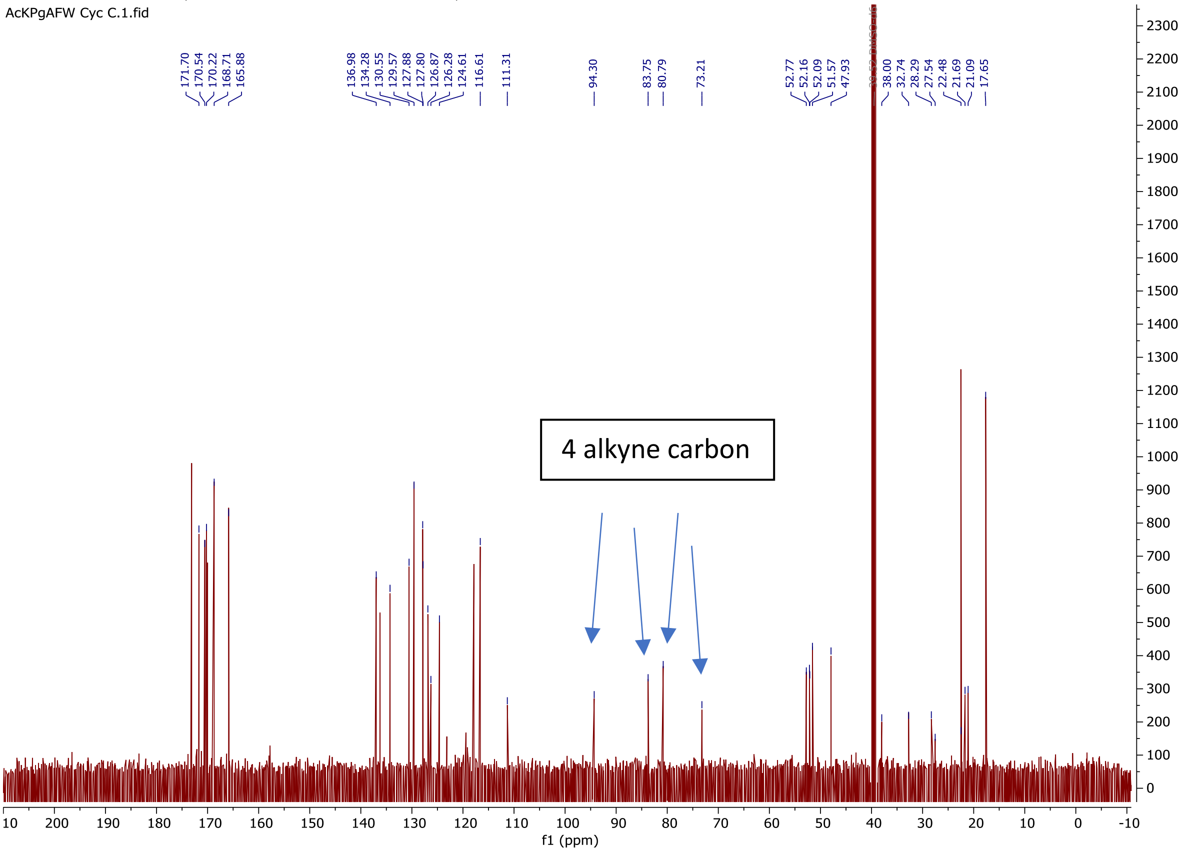

HMBC spectrum of **2k**, terminal alkyne is untouched after reaction

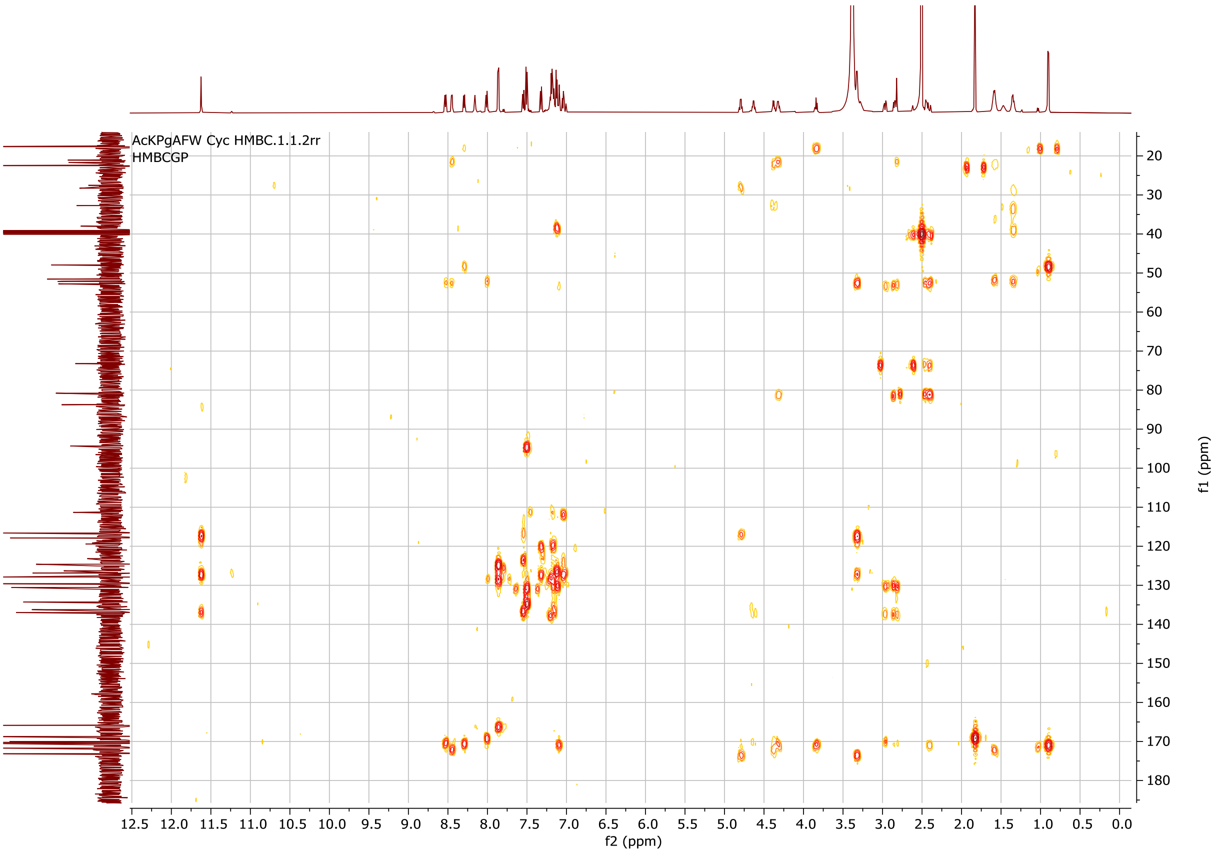

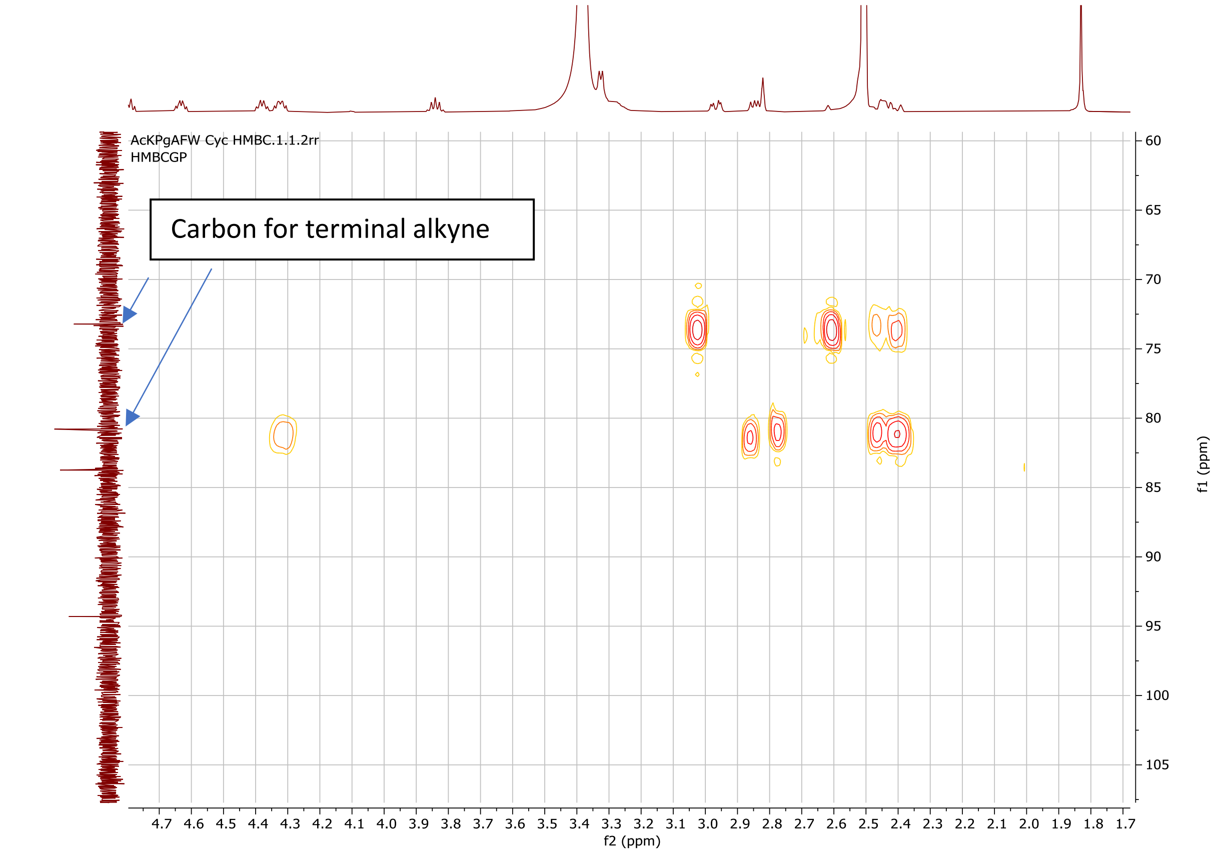

2p ^{1}H NMR (600 MHz, DMSO-d_6)

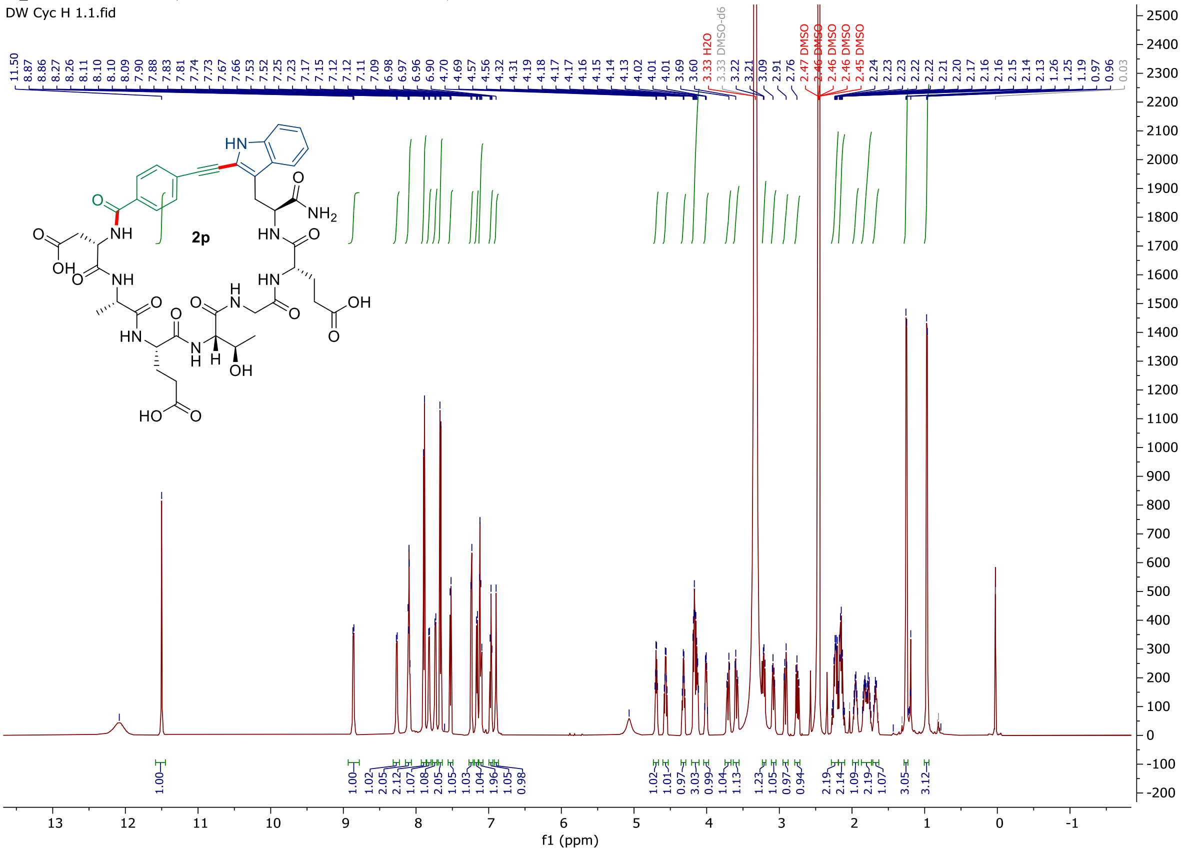

2p ^{13}C NMR (126 MHz, DMSO-d_6)

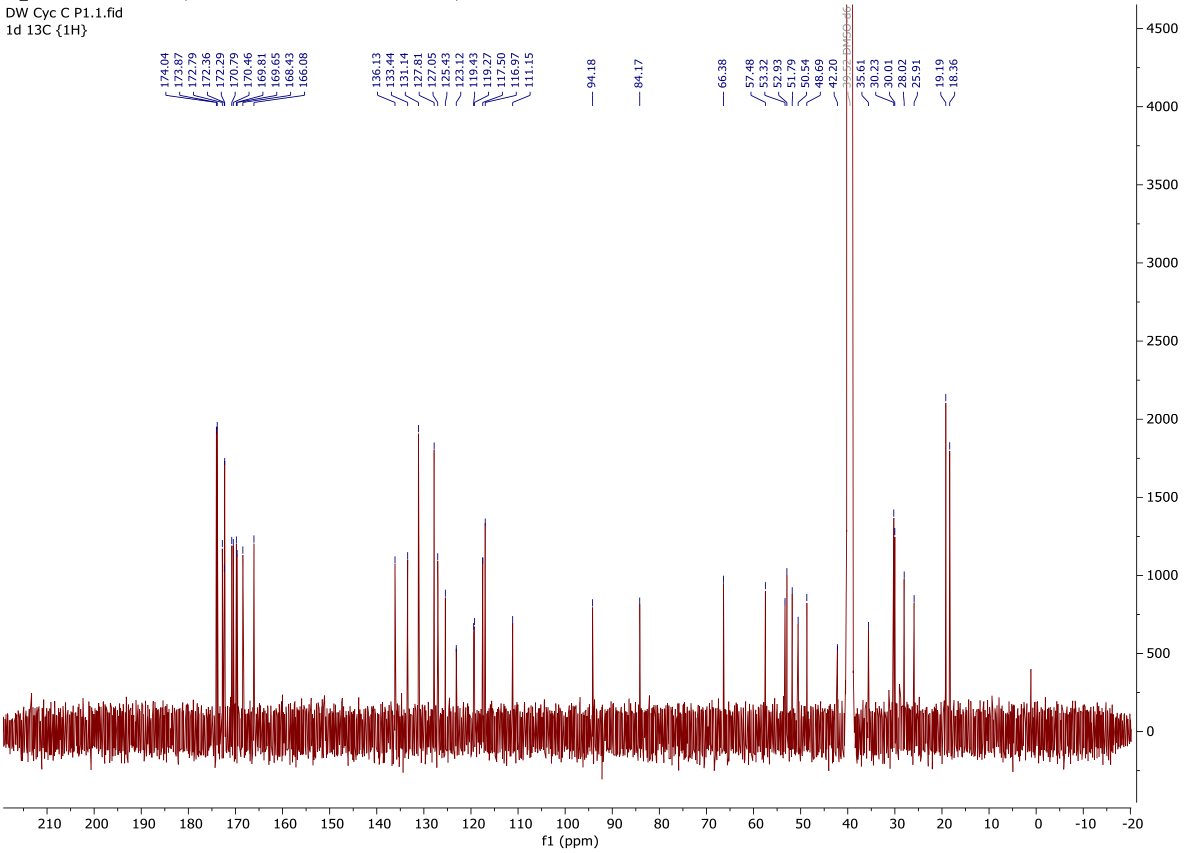

3p ^1H NMR (600 MHz, DMSO-d_6)

DW Cyc P2.1.fid

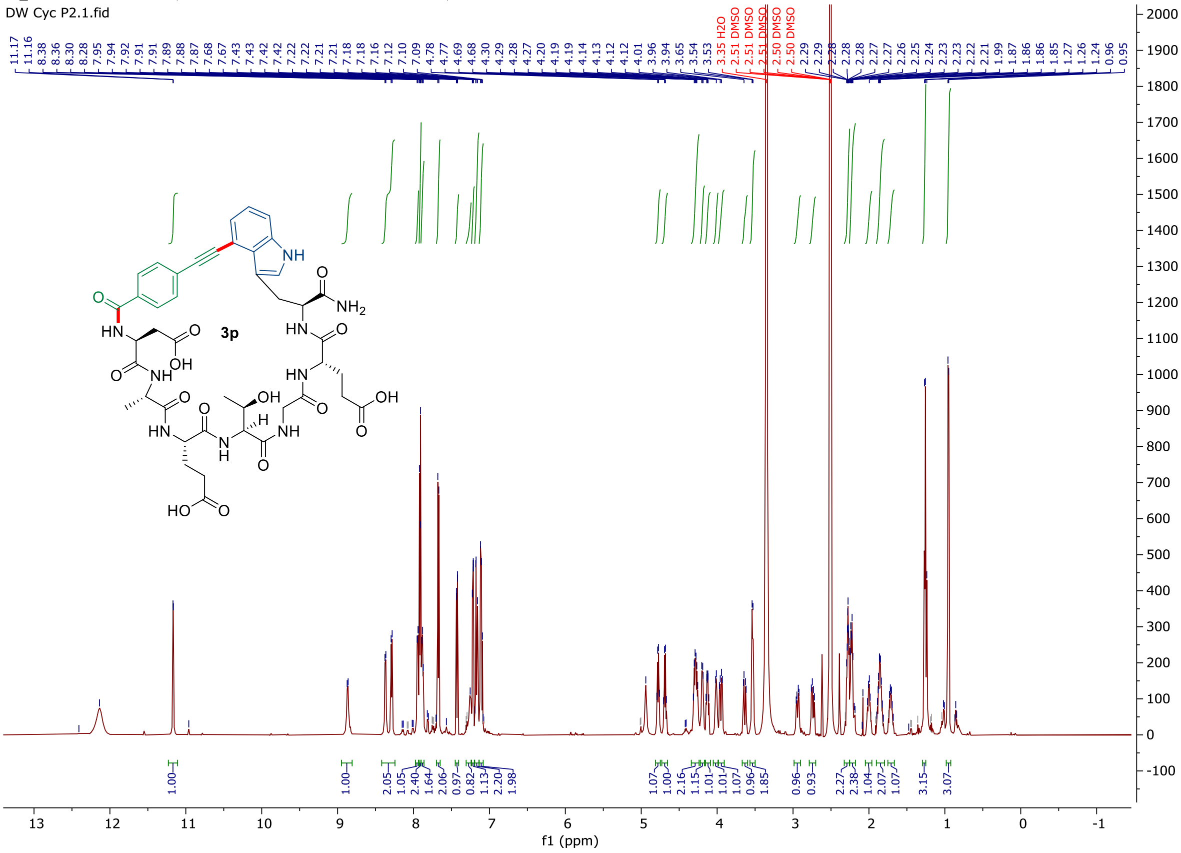

3p ^{13}C NMR (126 MHz, DMSO-d_6)

DW Cyc C P2.1.fid
1d 13C {1H}

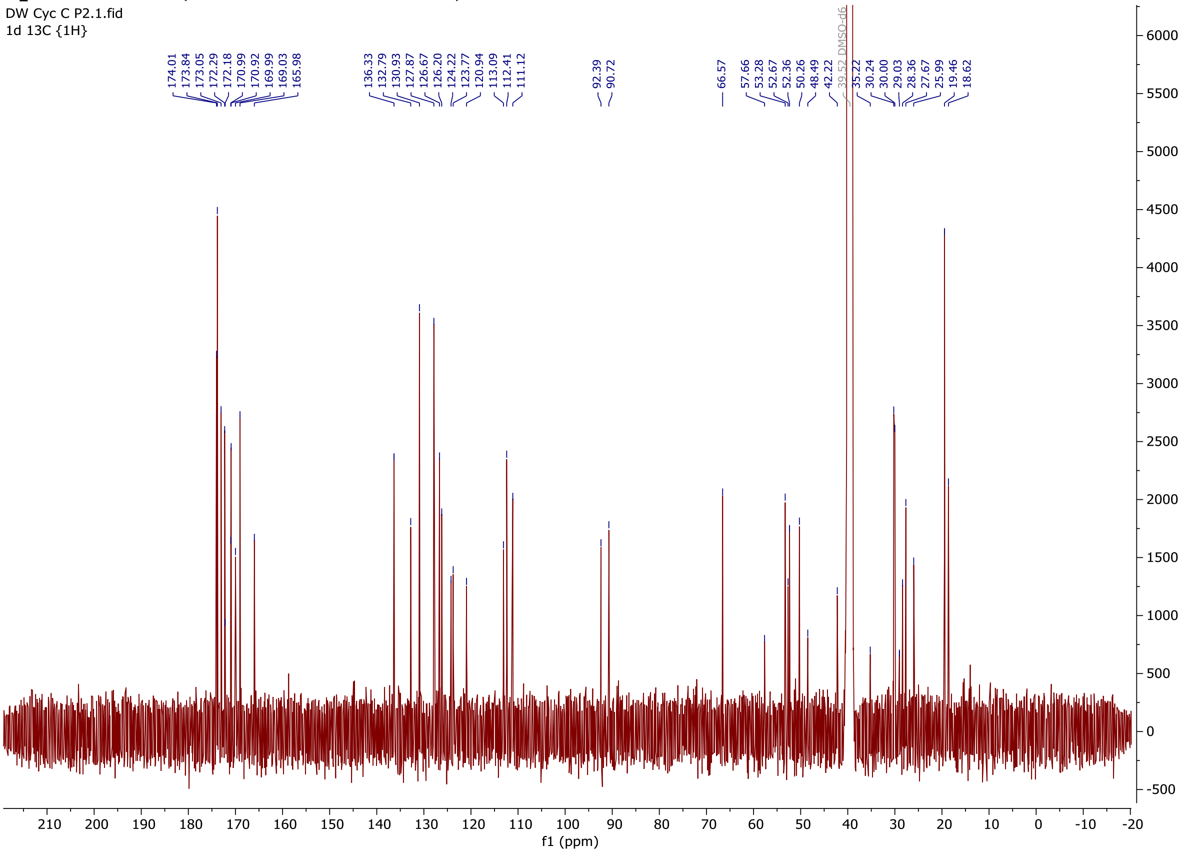

2u ^{1}H NMR (500 MHz, DMSO-d$_6$)

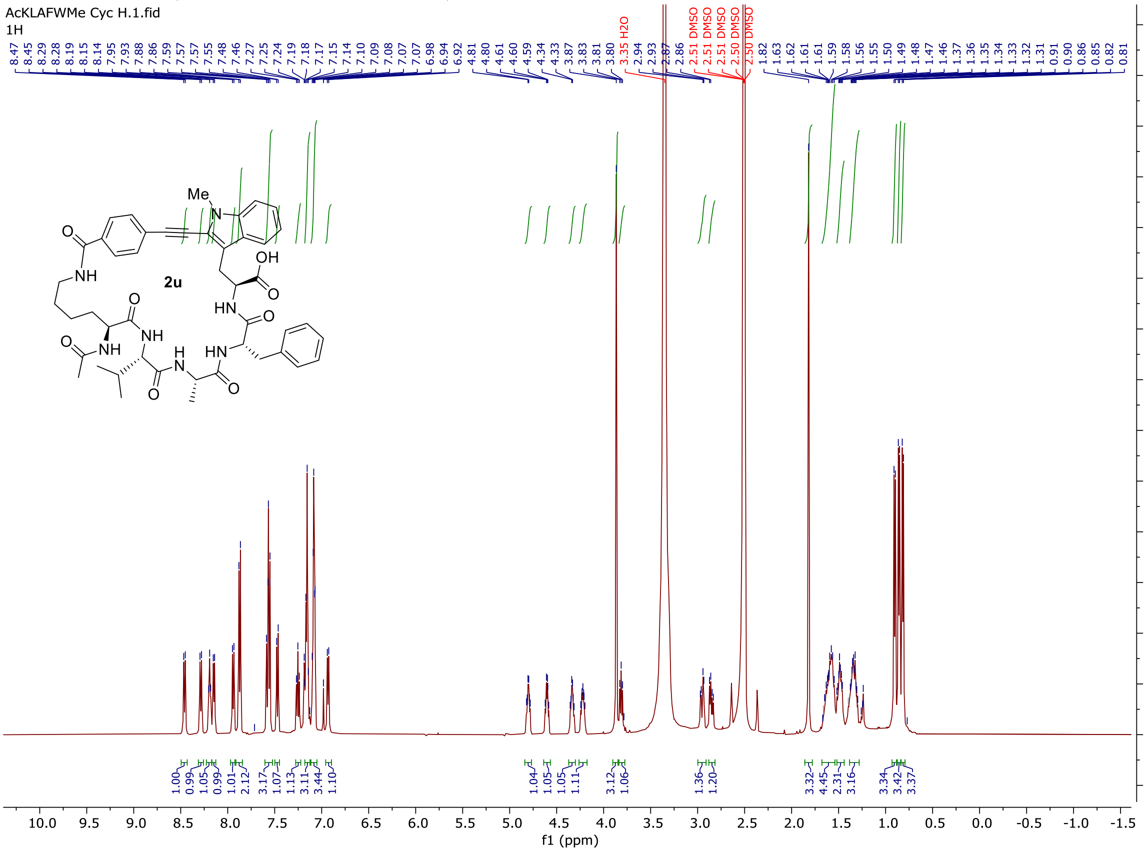

^{13}C NMR (126 MHz, DMSO-d$_6$)

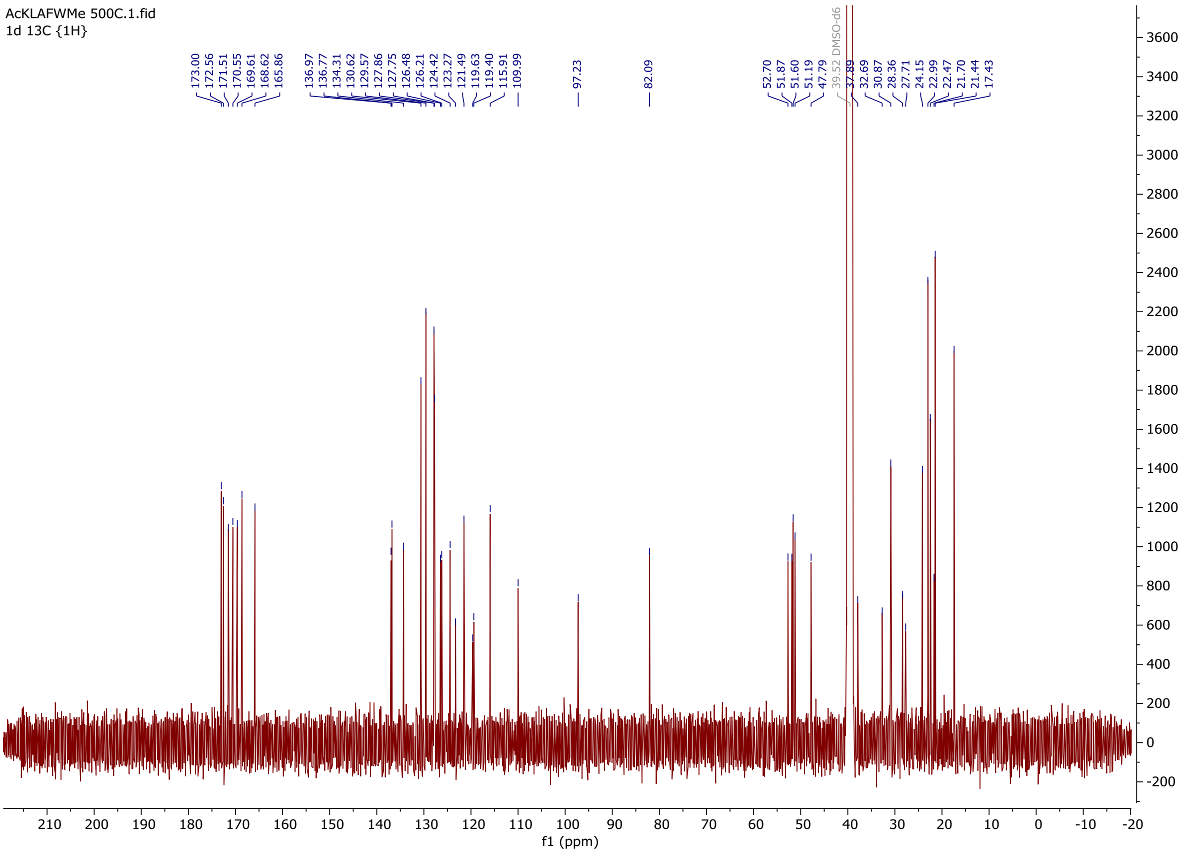

HMBC of **2u**

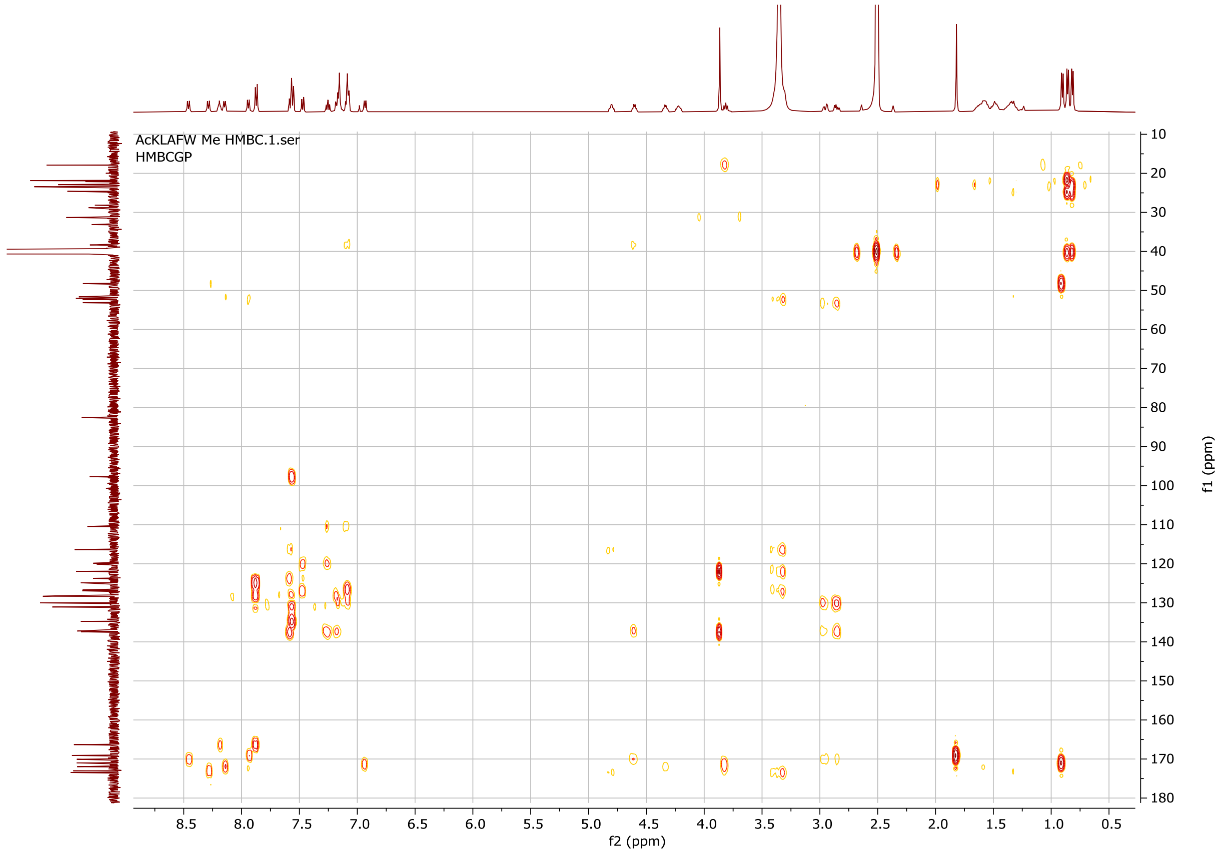

Zoom-in

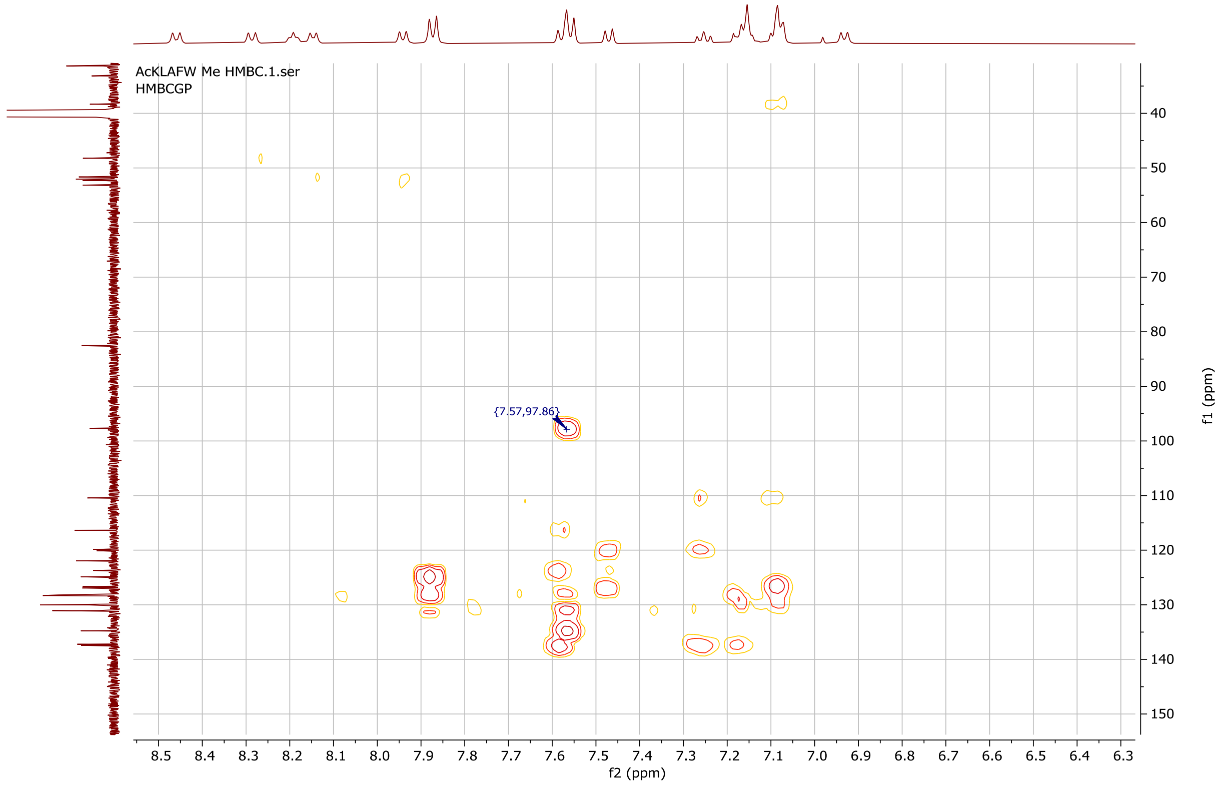

Supplement: Supplementary file 1 — ja3c09261_si_001.pdf [file ja3c09261_si_001.pdf]
